# Supplementary material for: A global perspective of advanced practice nursing research: A review of systematic reviews
Source: PLoS One. 2024 Jul 2;19(7):e0305008. doi: 10.1371/journal.pone.0305008 (PMC11218965; doi:10.1371/journal.pone.0305008)
Supplement: S1 Appendix — (PDF) [file pone.0305008.s002.pdf]

S1 Appendix. Record of review-related decisions.

Excluded for wrong study design:

1. Aginga C. Evidence Summary. Nurse-Led Preoperative Assessment Services. The JBI EBP Database. 2022; JBI-ES-2313-4.
2. Andriopoulou M, Mpellou P, Dalamaga D. [The advanced role of nurses]. Perioper Nurs. 2018;7(2):89–105. Greek.
3. Barnett M, Balkissoon C, Sandhu J. The level of quality care nurse practitioners provide compared with their physician colleagues in the primary care setting: A systematic review. J Am Assoc Nurse Pract. 2022;34(3):457-464.
4. Carney M. Regulation of advanced nurse practice: its existence and regulatory dimensions from an international perspective. J Nurs Manag. 2016;24(1):105-114.
5. Chan SS, Cheung NK, Graham CA, Rainer TH. Strategies and solutions to alleviate access block and overcrowding in emergency departments. Hong Kong Med J. 2015;21(4):345-52.
6. Hsieh PL, Chen CM. [Nurse-led care models in the context of community elders with chronic disease management: A systematic review]. Hu Li Za Zhi. 2016;63:35-49. Chinese.
7. Thompson HJ, Belza B, Baker M, Christianson P, Doorenbos A, Nguyen H. Identifying and evaluating electronic learning resources for use in adult-gerontology nurse practitioner education. J Prof Nurs. 2014;30:155-161.
8. Tej Krnel T. [The advanced nurse practitioner's role in health care of adolescents with nonsuicidal self-injury behavior: literature review]. Obzor Zdrav Neg. 2019;53(1):57-69. Slovenian.
9. Crawford CC. Addition of advanced practice registered nurses to the trauma team: An integrative systematic review of literature. J Trauma Nurs. 2019;26(3):141-146.
10. Fry M. Literature review of the impact of nurse practitioners in critical care services. Nurs Crit Care. 2011;16(2):58-66.
11. Graham K, Stern C. A systematic review of the effectiveness of different nursing staff models on patient, staff and organisational outcomes in Day Surgery Units. JBI Libr Syst Rev. 2007;5(10 Suppl):1-13.
12. Gyi A, Sivapuram M. Evidence Summary. Nurse-led Preoperative Assessment: Elective Surgery. The JBI EBP Database. 2022; JBI-ES-3064-4.
13. Hare N, Hopkins P, Lee G, Vercueil A. Rapid nurse led assessment of critically ill patients by sonography: A systematic review & multidisciplinary team survey. Intensive Care Med. 2014;1:S30.
14. Kyzas P. Nurse-led oral and maxillofacial oncology clinics: a review. Br J Oral Maxillofac Surg. 2021;59:151-155.
15. Lazarus JV, Anstee QM, Hagstrom H, Cusi K, Cortez-Pinto H, Mark HE, et al. Defining comprehensive models of care for NAFLD. Nat Rev Gastroenterol Hepatol. 2021;18:717-729.
16. Lorenzo L. Partnering with patients to promote holistic diabetes management: Changing paradigms. J Am Assoc Nurse Pract. 2013;25(7):351-361.
17. Marin T. Evidence Summary. Nursing Care: Principles of Nurse-Led Patient Education. The JBI EBP Database. 2021; JBI-ES-117-1.
18. Marin T, Travers C. Evidence Summary. Reducing Unnecessary Hospitalization (Older People): Interventions. The JBI EBP Database. 2021; JBI-ES-2472-2.
19. Martelly MT, Squires A. Effect of substituting nurses for doctors in primary care. J Clin Outcomes Manag. 2014;21:398-399.
20. Moola S. Evidence Summary. Nurse-led Cardiac Rehabilitation Clinics: Post Coronary Artery Bypass Graft. The JBI EBP Database. 2021; JBI-ES-649-1.
21. Owaya A. Evidence Summary. Role Substitution: Nursing, Midwifery, Mental Health and Long Term Conditions. The JBI EBP Database. 2021; JBI-ES-3160-2.
22. Shah T, Deswal A. ACP Journal Club. Review: In HF with reduced EF, nurse-led titration of HF drugs reduces hospitalizations and mortality. Ann Intern Med. 2016;164(8):JC41.

23. Travers C. Evidence Summary. Aged Care (Staffing): Nurse Practitioners. The JBI EBP Database. 2021; JBI-ES-1198-1.
24. Villasenor S, Krouse HJ. Can the use of urgent care clinics improve access to care without undermining continuity in primary care? J Am Assoc Nurse Pract. 2016;28(6):335-341.
25. Zwilling JG, Fiandt K. Where are we now? Practice-level utilization of nurse practitioners in comparison with state-level regulations. J Am Assoc Nurse Pract. 2020;32(6):429-437.
26. White T, Kokiousis J, Ensminger S, Shirey M. Supplementing intensivist staffing with nurse practitioners: Literature review. AACN Adv Crit Care. 2017;28(2):111-123.

Excluded for wrong role:

27. Abdel-Aleem H, El-Gibaly OMH, El-Gazzar AFES, Al-Attar GST. Mobile clinics for women's and children's health. Cochrane Database Syst Rev. 2016;2016(8):CD009677.
28. Abu-Qamar MZ, Vafeas C, Ewens B, Ghosh M, Sundin D. Postgraduate nurse education and the implications for nurse and patient outcomes: A systematic review. Nurse Educ Today. 2020;92:104489.
29. Al-Mallah MH, Farah I, Al-Madani W, Bdeir B, Al Habib S, Bigelow ML, et al. The impact of nurse-led clinics on the mortality and morbidity of patients with cardiovascular diseases: A systematic review and meta-analysis. J Cardiovasc Nurs. 2016;31:89-95.
30. Anderson DA, Clemett V. What impact do specialist and advanced-level nurses have on people living with heart failure compared to physician-led care? A literature review. J Res Nurs. 2021;26(3):229-249.
31. Anthony BF, Surgey A, Hiscock J, Williams NH, Charles JM. General medical services by non-medical health professionals: A systematic quantitative review of economic evaluations in primary care. Br J Gen Pract. 2019;69:E304-E313.
32. Bae KR, Cho J. Research trends for nurse-led interventions to decrease fear of cancer recurrence. Asian Oncol Nurs. 2020;20(1):20-27.
33. Baker E, Fatoye F. Patient perceived impact of nurse-led self-management interventions for COPD: A systematic review of qualitative research. Int J Nurs Stud. 2019;91:22-34.
34. Beltran SJ, Hamel MN. Caring for dying infants: A systematic review of healthcare providers' perspectives of neonatal palliative care. Am J Hosp Palliat Care. 2021;38(8):1013-1027.
35. Bradley PM, Lindsay B, Fleeman N. Care delivery and self management strategies for adults with epilepsy. Cochrane Database Syst Rev. 2016;2(2):CD006244.
36. Bridges J, Lucas G, Wiseman T, Griffiths P. Workforce characteristics and interventions associated with high-quality care and support to older people with cancer: A systematic review. BMJ Open. 2017;7(7): e016127.
37. Chan RJ, Teleni L, McDonald S, Kelly J, Mahony J, Ernst K, et al. Breast cancer nursing interventions and clinical effectiveness: a systematic review. BMJ Support Palliat Care. 2020;10:276-286.
38. Chiang CY, Choi KC, Ho KM, Yu SF. Effectiveness of nurse-led patient-centered care behavioral risk modification on secondary prevention of coronary heart disease: A systematic review. Int J Nurs Stud. 2018;84:28-39.
39. Clark CE, Smith LF, Taylor RS, Campbell JL. Nurse-led interventions used to improve control of high blood pressure in people with diabetes: A systematic review and meta-analysis. Diabet Med. 2011;28(3):250-261.
40. Mares MA, McNally S, Fernandez RS. Effectiveness of nurse-led cardiac rehabilitation programs following coronary artery bypass graft surgery: a systematic review. JBI Database Syst Rev Implement Rep. 2018;16(12):2304-2329.
41. Hickman L, Ferguson C, Davidson PM, Allida S, Inglis S, Parker D, et al. Key elements of interventions for heart failure patients with mild cognitive impairment or dementia: A systematic review. Eur J Cardiovasc Nurs. 2020;19(1):8-19.
42. Howell D, Hack TF, Oliver TK, Chulak T, Mayo S, Aubin M, et al. Models of care for post-treatment follow-up of adult cancer survivors: A systematic review and quality appraisal of the evidence. J Cancer Surviv. 2012;6:359-371.

43. Crowe M, Jones V, Stone MA, Coe G. The clinical effectiveness of nursing models of diabetes care: A synthesis of the evidence. Int J Nurs Stud. 2019;93:119-128.
44. Mitosi N, Papadopoulou K, Matziou V. [Can the nurse educator influence the therapeutic result in atopic dermatitis in children?] Hellenic Journal of Nursing. 2012;51(3):269-277. Greek.
45. Stephens M, Hourigan LF, Appleyard M, Ostapowicz G, Schoeman M, Desmond PV, et al. Non-physician endoscopists: A systematic review. World J Gastroenterol. 2015;21(16):5056-5071.
46. Considine J, Shaban RZ, Curtis K, Fry M. Effectiveness of nurse-initiated X-ray for emergency department patients with distal limb injuries: A systematic review. Eur J Emerg Med. 2019;26:314-322.
47. Garry L, Rohan N, O'Connor T, Patton D, Moore Z. Do nurse-led critical care outreach services impact inpatient mortality rates? Nurs Crit Care. 2018;24:40-46.
48. Gutiérrez-Alemán T, Esandi N, Pardavila-Belio MI, Pueyo-Garrigues M, Canga-Armayor N, Alfaro-Díaz C, et al. Effectiveness of educational programs for clinical competence in family nursing: A systematic review. J Fam Nurs. 2021;27(4):255-274.
49. Djerbib A. A qualitative systematic review of the factors that influence prescribing decisions by nurse independent prescribers in primary care. Primary Health Care. 2018;28(3):25-34.
50. Hill H, McMeekin P, Price C. A systematic review of the activity and impact of emergency care practitioners in the NHS. Emerg Med J. 2014;31(10):853-860.
51. Malik M, Moore Z, Patton D, O'Connor T, Nugent LE. The impact of geriatric focused nurse assessment and intervention in the emergency department: A systematic review. Int Emerg Nurs. 2018;37:52-60.
52. Satherley R, Lonergan K, Devakumar D, Cheung R, Cortina-Borja M, Heys M, et al. A systematic review and meta-analysis of out of hospital nursing interventions to reduce emergency department attendances in children and young people. Arch Dis Child. 2018;103(Suppl 1):A173.
53. Clark C, Smith L, Cloutier L, Glynn L, Clark O, Taylor R, et al. Allied health professional-led interventions for improving control of blood pressure in patients with hypertension: A cochrane systematic review and meta-analysis. J Hypertens. 2018;1:e44.
54. Cleary M, Kornhaber R, Sayers J, Gray R. Mental health nurse prescribing: A qualitative, systematic review. Int J Ment Health Nurs. 2017;26(6):541-553.
55. Cook O, McIntyre M, Recoche K. Exploration of the role of specialist nurses in the care of women with gynaecological cancer: a systematic review. J Clin Nurs. 2015;24(5):683-695.
56. Corones-Watkins K, Cooke M, Theobald K, White K, Thompson DR, Ski CF, et al. Effectiveness of nurse-led clinics in the early discharge period after percutaneous coronary intervention: A systematic review. Aust Crit Care. 2021;34(5):510-517.
57. Coughlan D, Kontogiannis V, Lovat P, Ellis R, Bajaw D, Labus M, et al. Surveillance strategies for AJCC Stage 1 Melanoma: The health technology assessment (HTA) approach. Pigment Cell Melanoma Res. 2020;33:164.
58. Crisford P, Winzenberg T, Venn A, Schultz M, Aitken D, Cleland V. Factors associated with physical activity promotion by allied and other non-medical health professionals: A systematic review. Patient Educ Couns. 2018;101(10):1775-1785.
59. Daniele RM, Bova AM, LeGar M, Smith PJ, Shortridge-Baggett LM. Rapid response team composition effects on outcomes for adult hospitalised patients: A systematic review. JBI Libr Syst Rev. 2011;9(31):1297-1340.
60. Davidson JR, Dickson C, Han H. Cognitive behavioural treatment for insomnia in primary care: A systematic review of sleep outcomes. Br J Gen Pract. 2019;69:E657-E664.
61. Day LW, Inadomi JM, Somsouk M. Non-physicians performing endoscopy: Systematic review and meta-analysis. Gastrointest Endosc. 2014;1:AB210.
62. Doebl S, Macfarlane GJ, Hollick RJ. “No one wants to look after the fibro patient”. Understanding models, and patient perspectives, of care for fibromyalgia: reviews of current evidence. PAIN. 2020;161:1716–1725.
63. Efraimsson EO, Lennmalm EA, Nyberg A. COPD care and management at nurse-led COPD-clinics in Swedish primary health care: A literature review. Eur Respir J. 2012;40:1.

64. Ekers D, Murphy R, Archer J, Ebenezer C, Kemp D, Gilbody S. Nurse-delivered collaborative care for depression and long-term physical conditions: A systematic review and meta-analysis. J Affect Disord. 2013;149:14-22.
65. Elliott J, Wiechula R. The acute care nurses' experience of caring for patients who receive nurse initiated interventions: A qualitative systematic review. JBI Libr Syst Rev. 2011;9:1–12.
66. Elliott S, Murrell K, Harper P, Stephens T, Pellowe C. A comprehensive systematic review of the use of simulation in the continuing education and training of qualified medical, nursing and midwifery staff. JBI Libr Syst Rev. 2011;9(17):538-587.
67. Ersser SJ, Cowdell F, Latter S, Gardiner E, Flohr C, Thompson AR, et al. Psychological and educational interventions for atopic eczema in children. Cochrane Database Syst Rev. 2014;2014(1):CD004054.
68. Foster CB, Simone S, Bagdure D, Garber NA, Bhutta A. Optimizing team dynamics: An assessment of physician trainees and advanced practice providers collaborative practice. Pediatr Crit Care Med. 2016;17:e430-e436.
69. Fry MM. A systematic review of the impact of afterhours care models on emergency departments, ambulance and general practice services. Australas Emerg Nurs J. 2011;14(4):217-225.
70. Garrard JW, Cox NJ, Dodds RM, Roberts HC, Sayer AA. Comprehensive geriatric assessment in primary care: A systematic review. Aging Clin Exp Res. 2020;32(2):197-205.
71. Geense WW, Van Den Boogaard M, Van Der Hoeven JG, Vermeulen H, Hannink G, Zegers M. Nonpharmacological interventions to prevent or mitigate adverse long-term outcomes among ICU survivors: A systematic review and meta-analysis. Crit Care Med. 2019;47:1607–1618.
72. Glick SB, Clarke AR, Blanchard A, Whitaker AK. Cervical cancer screening, diagnosis and treatment interventions for racial and ethnic minorities: A systematic review. J Gen Intern Med. 2012;27(8):1016-1032.
73. Gong F, Chen X, Wu Y, Yao D, Xie L, Ouyang Q, et al. Nurse vs. physician-led care for obstructive sleep apnoea: A systematic review and meta-analysis of randomized trials. J Adv Nurs. 2018;74:501-6.
74. Grassley JS, Strohfus PK, Lambe AC. No Longer Expert: A meta-synthesis describing the transition from clinician to academic. J Nurs Educ. 2020;59(7):366-374.
75. Grundy Q, Bero L, Malone R. Interactions between non-physician clinicians and industry: a systematic review. PLoS Med. 2013;10(11):e1001561.
76. Hines S, Munday J, Kynoch K. Effectiveness of nurse-led preoperative assessment services for elective surgery: A systematic review update. JBI Database Syst Rev Implement Rep. 2015;13(6):279-317.
77. Ho JK, Chau JP, Cheung NM. Effectiveness of emergency nurses' use of the Ottawa Ankle Rules to initiate radiographic tests on improving healthcare outcomes for patients with ankle injuries: A systematic review. Int J Nurs Stud. 2016;63:37-47.
78. Hoff T, Carabetta S, Collinson GE. Satisfaction, burnout, and turnover among nurse practitioners and physician assistants: A review of the empirical literature. Med Care Res Rev. 2019;76(1):3-31.
79. Hoff T, Prout K. Comparing retail clinics with other sites of care: A systematic review of cost, quality, and patient satisfaction. Med Care. 2019;57:734-741.
80. Jakimowicz M, Williams D, Stankiewicz G. A systematic review of experiences of advanced practice nursing in general practice. BMC Nurs. 2017;18:1-12.
81. Steen M, Raynor J, Baldwin CD, Jee SH. Child adversity and trauma-informed care teaching interventions: A systematic review. Pediatrics. 2021;149(3):e2021051174.
82. Johal J, Dodd A. Physician extenders on surgical services: a systematic review. Can J Surg. 2017;60(3):172-178.
83. Joyner RL, Strickland SL, Becker EA, Ginier E, Keene S, Rye K, et al. Adequacy of the provider workforce for persons with cardiopulmonary disease. Chest. 2020;157:1221-1229.
84. Keijsers CJPW, van Hensbergen L, Jacobs L, Brouwers JRBJ, de Wildt DJ, ten Cate OTJ, et al. Geriatric pharmacology and pharmacotherapy education for health professionals and students: A systematic review. Br J Clin Pharmacol. 2012;74:762-773.
85. King AJ, Evans M, Moore TH, Paterson C, Sharp D, Persad R, et al. Prostate cancer and supportive care: a

systematic review and qualitative synthesis of men's experiences and unmet needs. Eur J Cancer Care. 2015;24:618-634.
86. Kreeftenberg HG, Pouwels S, Bindels A, de Bie A, van der Voort PHJ. Impact of the advanced practice provider in adult critical care: A systematic review and meta-analysis. Crit Care Med. 2019;47(5):722-730.
87. Lambert CC, Gall B, Enriquez M, Reynolds NR. A systematic review of nurse-led antiretroviral medication adherence intervention trials: How nurses have advanced the science. J Assoc Nurses AIDS Care. 2021;32:347-372.
88. Laserna Jiménez C, López Poyato M, Casado Montañés I, Guix-Comellas EM, Fabrellas N. Paediatric nursing clinical competences in primary healthcare: A systematic review. J Adv Nurs. 2021;77(6):2662-2679.
89. Lee MK. Effects of nurse-led telephone-based supportive interventions for patients with cancer: A meta-analysis. Oncol Nurs Forum. 2017;44(2):1.
90. Lewis SR, Nicholson A, Smith AF, Alderson P. Physician anaesthetists versus non-physician providers of anaesthesia for surgical patients. Cochrane Database Syst Rev. 2014:(7):CD010357.
91. Liu J, Butow P, Beith J. Systematic review of interventions by non-mental health specialists for managing fear of cancer recurrence in adult cancer survivors. Support Care Cancer. 2019;27:4055-4067.
92. Lois P, Lopez Pedraza L, Candelas G, Lajas C, Rodero M, Mulero Lopez T, et al. Benefit of a nurse-led program of care for management of patients with established rheumatoid arthritis: Systematic literature review. Ann Rheum Dis. 2021;80:1475.
93. Mayeux JJ, Ng YC. Pre-exposure prophylaxis in the urgent care setting: A systematic review. J Nurse Pract. 2019;15(8):595-599.
94. McIntosh T, Stewart D, Forbes-McKay K, McCaig D, Cunningham S. Influences on prescribing decision-making among non-medical prescribers in the United Kingdom: Systematic review. Fam Pract. 2016;33:572-579.
95. Nicholson A, Coldwell CH, Lewis SR, Smith AF. Nurse-led versus doctor-led preoperative assessment for elective surgical patients requiring regional or general anaesthesia. Cochrane Database Syst Rev. 2013;11:CD010160.
96. Nocera N, Eckert K, Yoon-Flannery K. The breast cancer survivorship program: A systematic review of literature with recommendations for successful implementation. Ann Surg Oncol. 2020;27:S476-S477.
97. Nowzari S, Finnell DS, Broyles LM. Nurse provision of alcohol-related interventions in the patient-centered medical home. Alcohol Clin Exp Res. 2013;2:205A.
98. Nuttall D. Nurse prescribing in primary care: a metasynthesis of the literature. Prim Health Care Res Dev. 2018;19(1):7-22.
99. O'Neill I, Gale CP, McCallum A, McIntyre H, Squire I, Cherif M. Impact of mode of delivery of disease management programmes on clinical outcomes among patients following hospitalised heart failure: A systematic review and meta-analysis. Eur J Heart Fail. 2017;19:227.
100. Papermaster A, Champion JD. The common practice of 'curbside consultation': A systematic review. J Am Assoc Nurse Pract. 2017;29(10):618-628.
101. Pereira de Paiva MH, Pinheiro Lages L, Cavalcanti de Medeiros Z. Studies on forensic nursing in Brazil: a systematic review of the literature. Int Nurs Rev. 2016;64(2):286-295.
102. Piazza M. Why speaking about continuity of care among cancer patients? How to ensure it? A literary review about organizational strategies. Ann Oncol. 2021;32:S1277.
103. Piot MA, Dechartres A, Attoe C, Romeo M, Jollant F, Billon G, et al. Effectiveness of simulation in psychiatry for nursing students, nurses and nurse practitioners: A systematic review and meta-analysis. J Adv Nurs. 2022;78(2):332-347.
104. Qiu X, Lan C, Xiao X, Li J. The effect of nurse-led interventions on re-admission and mortality for congestive heart failure: A meta-analysis. Medicine. 2021;100:e24599.
105. Rasul A, Subhi Y, Sorensen TL, Munch IC. Non-Physician delivered intravitreal injection service is feasible and safe - A systematic review. Dan Med J. 2016;63(5):A5229.
106. Rega ML, De Vito C, Ruberto M, Grossi V, Damiani G. The Nurse Led of stroke patient after discharge

from hospital. A systematic review and GRADE. Prof Inferm. 2020;73(4):278-287.
107. Reid C, Hall J, Boys J, Lewis S, Chang A. Self management of haemodialysis for End Stage Renal Disease: A systematic review. JBI Libr Syst Rev. 2011;9(3):69-103.
108. Reyes AM, Akanyirige PW, Wishart D, Dahdouh R, Young MR, Estrada A, et al. Interventions addressing social needs in perinatal care: A systematic review. Health Equity. 2021;5(1):100-118.
109. Rocke J, McLaren O, Hardman J, Garas G, Smith ME, Ishii H, et al. The role of allied healthcare professionals in head and neck cancer surveillance: A systematic review. Clin Otolaryngol. 2020;45:83-98.
110. Santomassino M, Costantini GD, McDermott M, Primiano D, Slyer JT, Singleton JK. A systematic review on the effectiveness of continuity of care and its role in patient satisfaction and decreased hospital readmissions in the adult patient receiving home care services. JBI Libr Syst Rev. 2012;10(21):1214-1259.
111. Sezgin MG, Bektas H. The effect of nurse-led care on fatigue in patients with rheumatoid arthritis: A systematic review and meta-analysis of randomised controlled studies. J Clin Nurs. 2022;31(7-8):832-842.
112. Sheehan TO, Davis NW, Peach BC, Ansell M, Cimiotti JP, Yi G, et al. Hospital characteristics and mortality in aneurysmal subarachnoid hemorrhage. J Neurosci Nurs. 2021;53(1):2-4.
113. Spacey A, Hipperson V, Gloster A, Mercer C. The role of the advanced clinical practitioner in breast diagnosis: A systematic review of the literature. Radiography. 2021;27(2):654-662.
114. Spears JA, Craft M, White S. Outcomes of cancer survivorship care provided by Advanced Practice RNs compared to other models of care: A systematic review. Oncol Nurs Forum. 2017;44(1):E34-E41.
115. Sullivan LE, Tetrault JM, Braithwaite RS, Turner BJ, Fiellin DA. A meta-analysis of the efficacy of nonphysician brief interventions for unhealthy alcohol use: Implications for the patient-centered medical home. Am J Addict. 2011;20:343-356.
116. Tabesh M, Magliano DJ, Koye DN, Shaw JE. The effect of nurse prescribers on glycaemic control in type 2 diabetes: A systematic review and meta-analysis. Int J Nurs Stud. 2018;78:37-43.
117. Tan SM, Han E, Quek RYC, Singh SR, Gea-Sanchez M, Legido-Quigley H. A systematic review of community nursing interventions focusing on improving outcomes for individuals exhibiting risk factors of cardiovascular disease. J Adv Nurs. 2020;76:47-61.
118. Van Erp RMA, Van Doorn AL, Van Den Brink GT, Peters JWB, Laurant MGH, Van Vught AJ. Physician assistants and nurse practitioners in Primary Care Plus: A systematic review. Int J Integr Care. 2021;21(1):1-17.
119. Varndell W, Topacio M, Hagness C, Lemon H, Tracy D. Nurse-performed focused ultrasound in the emergency department: A systematic review. Australas Emerg Care. 2018;21:121-130.
120. Walsh JC. A nurse led clinic's contribution to patient education and promoting self-care in heart failure patients: A systematic review. Int J Integr Care. 2017;17:1-2.
121. Whear R, Thompson-Coon J, Rogers M, Abbott RA, Anderson L, Ukoumunne O, et al. Patient-initiated appointment systems for adults with chronic conditions in secondary care. Cochrane Database Syst Rev. 2020;4(4):CD010763.
122. Wood EM, Zani B, Esterhuizen TM, Young T. Nurse led home-based care for people with HIV/AIDS. BMC Health Serv Res. 2018;18(1):219.
123. Yung DE, Fernandez-Urien I, Douglas S, Plevris JN, Sidhu R, McAlindon ME, et al. Systematic review and meta-analysis of the performance of nurses in small bowel capsule endoscopy reading. United European Gastroenterol J. 2017;5:1061-1072.
124. Zusman EZ, Kapanen AI, Klaassen A, Reardon J. Workplace cardiovascular risk reduction by healthcare professionals-a systematic review. Occup Med. 2021;71:270-276.
125. Randall S, Crawford T, Currie J, River J, Betihavas V. Impact of community based nurse-led clinics on patient outcomes, patient satisfaction, patient access and cost effectiveness: A systematic review. Int J Nurs Stud. 2017;73:24-33.
126. Grant KL, Bayley CJ, Premji Z, Lang E, Innes G. Throughput interventions to reduce emergency department crowding: A systematic review. CJEM. 2020;22(6):864–874.
127. Gomez del Pulgar M, Cuevas-Budhart MA, Hernández-Iglesias S, Kappes M, Riquelme Contreras VA, Rodriguez-Lopez E, et al. Best nursing intervention practices to prevent non-communicable disease: A

systematic review. Public Health Rev. 2022;43:1604429.
128. Martins AFM, Batista FIO, Silva C, Pino HN, Nunes I. Self-care Promotion in the Person Undergoing Hip Arthroplasty: Gains Sensitive to Rehabilitation Nursing Care. In: García-Alonso J, Fonseca C, editors. IWoG 2021, LNBE. EVORA: Portugal; 2022. pp. 171–181. doi: 10.1007/978-3-030-97524-1_16
129. Mayo-Smith MF, Robbins RA, Murray M, Weber R, Bagley PJ, Vitale EJ, et al. Analysis of variation in organizational definitions of primary care panels: A systematic review. JAMA Netw Open. 2022;5(4):e227497.
130. McCullough K, Andrew L, Genoni A, Dunham M, Whitehead L, Porock D. An examination of primary health care nursing service evaluation using the Donabedian model: A systematic review. Res Nurs Health. 2023;46(1):159-176.
131. National Guideline Alliance (UK). Effectiveness of a nurse specialist in the management of epilepsy: Epilepsies in children, young people and adults: Evidence review O. London: National Institute for Health and Care Excellence (NICE); 2022 Apr. (NICE Guideline, No. 217.) Available from: https://www.ncbi.nlm.nih.gov/books/NBK581161/
132. Sharma S K, Thakur K, Kant R, Mudgal SK. Impact of nurse-led titration versus physician prescription of hypoglycaemic agents on HbA1c level in type 2 diabetes patients: A systematic review and meta-analysis of randomized controlled trials. Cureus. 2021;13(12): e20436.
133. Watkins AK, Clark AP, Champion JD. Telemedicine practices in adult patients with atrial fibrillation. J Am Assoc Nurse Pract. 2022;34(8):957-962.
134. Zhang J, Zheng X, Ma D, Liu C, Ding Y. Nurse-led care versus usual care on cardiovascular risk factors for patients with type 2 diabetes: a systematic review and meta-analysis. BMJ Open. 2022;12(3):e058533.
135. Kappes M, Espinoza P, Jara V, Hall A. Nurse-led telehealth intervention effectiveness on reducing hypertension: a systematic review. BMC Nurs. 2023;22(1):19.

Excluded for no APN outcome:

136. Braet A, Weltens C, Sermeus W. Effectiveness of discharge interventions from hospital to home on hospital readmissions: A systematic review. JBI Database System Rev Implement Rep. 2016;14(2):106-173.
137. Laurant M, van der Biezen M, Wijers N, Watananirun K, Kontopantelis E, van Vught AJ. Nurses as substitutes for doctors in primary care. Cochrane Database Syst Rev. 2018;7:CD001271.
138. Gonçalves-Bradley D, Khangura JK, Flodgren G, Perera R, Rowe BH, Shepperd S. Primary care professionals providing non-urgent care in hospital emergency departments. Cochrane Database Syst Rev. 2018;2(2):CD002097.
139. Lagisetty P, Klasa K, Bush C, Heisler M, Chopra V, Bohnert A. Primary care models for treating opioid use disorders: What actually works? A systematic review. PLoS ONE. 2017;12(10):e0186315.
140. Linedale EC, Mikocka-Walus A, Gibson PR, Andrews JM. The potential of integrated nurse-led models to improve care for people with functional gastrointestinal disorders: A systematic review. Gastroenterol Nurs. 2020;43(1):53-64.
141. Morse M, Procter N. Review: exploring the role of mental health nurse-practitioner in the treatment of early psychosis. J Clin Nurs. 2011;20(20):2702-2711.
142. Sanders VL, Flanagan J. Radiology physician extenders: A literature review of the history and current roles of physician extenders in medical imaging. J Allied Health. 2015;44(4):219-224.
143. Zheng J, Mednick T, Heidenreich PA, Sandhu AT. Pharmacist- and nurse-led medical optimization in heart failure: A systematic review and meta-analysis. J Card Fail. 2023;29(7):1000-1013.
