# Supplementary material for: A global perspective of advanced practice nursing research: A review of systematic reviews
Source: PLoS One. 2024 Jul 2;19(7):e0305008. doi: 10.1371/journal.pone.0305008 (PMC11218965; doi:10.1371/journal.pone.0305008)
Supplement: S2 Appendix — (PDF) [file pone.0305008.s003.pdf]

S2 Appendix. Search strategies for the published literature.

1. PubMed

**Search strategy concept 1 – Advanced Practice Nursing**, comprised of the following 3 sub-concepts:<br><br>1.1) Advanced Practice Nurse;	#1	("Advanced Practice Nursing"[MH] OR "advanced practice nurse"[tiab] OR "advanced practice nurses"[tiab] OR "advanced practice nursing"[tiab] OR ("APN"[tiab] AND "nurse"[tiab]) OR ("APN"[tiab] AND "nurses"[tiab]) OR ("APN"[tiab] AND "nursing"[tiab]) OR "advanced nursing practice"[tiab] OR "advanced nursing practices"[tiab] OR "Nurse-led"[tiab] OR "Nurses-led"[tiab] OR "Nursing-led"[tiab] OR "Advanced clinical practitioner"[tiab] OR "Advanced clinical practitioners"[tiab] OR "Advanced clinical practice"[tiab] OR "Advanced practice clinician"[tiab] OR "Advanced practice clinicians"[tiab] OR "Advanced practitioner"[tiab] OR "Advanced practitioners"[tiab] OR Certified paediatric nurse[tiab] OR Certified paediatric nurses[tiab] OR "Certified pediatric nurse"[tiab] OR "Certified pediatric nurses"[tiab] OR "Community health nurse"[tiab] OR "Community health nurses"[tiab] OR "Community health nursing"[tiab] OR "Expert nurse"[tiab] OR "Expert nurses"[tiab] OR "Expert nursing"[tiab] OR ("Master"[tiab] AND "advanced practice nursing"[tiab]) OR ("Nurse"[tiab] AND "advanced practice"[tiab]) OR ("Nurses"[tiab] AND "advanced practice"[tiab]) OR "Nurse prescriber"[tiab] OR "Nurse prescribers"[tiab] OR Registered nurse extended class[tiab] OR Registered nurses extended class[tiab] OR "RN(EC)"[tiab] OR Enfermera practica avanzada[tiab] OR Enfermeras practica avanzada[tiab] OR "Enfermera de practica avanzada"[tiab] OR "Enfermeras de practica avanzada"[tiab] OR "Enfermera gestora de casos"[tiab] OR Enfermeras gestoras de casos[tiab] OR "Infirmière de pratique avancée"[tiab] OR Infirmières de pratique avancée[tiab] OR Infirmiere de pratique avancee[tiab] OR Infirmieres de pratique avancee[tiab] OR "Infirmière en pratique avancée"[tiab] OR Infirmières en pratique avancée[tiab] OR Infirmiere en pratique avancee[tiab] OR Infirmieres en pratique avancee[tiab] OR Pflegeexperte APN[tiab] OR "Pflegeexpertin"[tiab])
1.2) Nurse Practitioner;	#2	("Nurse Practitioners"[MH] OR "advanced nurse practitioner"[tiab] OR "advanced nurse practitioners"[tiab] OR ("ANP"[tiab] AND "nurse"[tiab]) OR ("ANP"[tiab] AND "nurses"[tiab]) OR ("ANP"[tiab] AND "nursing"[tiab]) OR "advanced practice registered nurse"[tiab] OR "advanced practice registered nurses"[tiab] OR "advanced practice registered nursing"[tiab] OR ("APRN"[tiab] AND "nurse"[tiab]) OR ("APRN"[tiab] AND "nurses"[tiab]) OR ("APRN"[tiab] AND "nursing"[tiab]) OR "Nurse practitioner"[tiab] OR "Nurse practitioners"[tiab] OR ("NP"[tiab] and "nurse"[tiab]) OR ("NP"[tiab] and "nurses"[tiab]) OR ("NP"[tiab] and "nursing"[tiab]) OR "Advanced registered nurse practitioner"[tiab] OR "Advanced registered nurse practitioners"[tiab] OR "Certified nurse practitioner"[tiab] OR "Certified nurse practitioners"[tiab] OR "Certified registered nurse practitioner"[tiab] OR "Certified registered nurse practitioners"[tiab] OR "Medical nurse practitioner"[tiab] OR Medical nurse practitioners[tiab] OR Registered advanced nurse practitioner[tiab] OR Registered advanced nurse practitioners[tiab] OR Registered nurse extended class nurse practitioner[tiab] OR Registered nurse extended class nurse practitioners[tiab] OR "Registered nurse practitioner"[tiab] OR "Registered nurse practitioners"[tiab] OR Registered nurse-nurse practitioner[tiab] OR Registered nurse-nurse practitioners[tiab] OR "RN(NP)"[tiab] OR "Women's health nurse practitioner"[tiab] OR "Women's health nurse practitioners"[tiab] OR ("WHNP"[tiab] and "nurse"[tiab]) OR ("WHNP"[tiab] and "nurses"[tiab]) OR ("WHNP"[tiab] and "nursing"[tiab]) OR "Infirmière praticienne spécialisée"[tiab] OR Infirmières praticiennes spécialisées[tiab] OR Infirmiere praticienne specialisee[tiab] OR Infirmieres praticiennes

		specialisees[tiab] OR "Family Nurse Practitioners"[MH] OR "Primary healthcare nurse practitioner"[tiab] OR "Primary healthcare nurse practitioners"[tiab] OR "Primary health care nurse practitioner"[tiab] OR "Primary health care nurse practitioners"[tiab] OR "Primary health-care nurse practitioner"[tiab] OR "Primary health-care nurse practitioners"[tiab] OR ("PHCNP"[tiab] AND "Nurse"[tiab]) OR ("PHCNP"[tiab] AND "Nurses"[tiab]) OR ("PHCNP"[tiab] AND "Nursing"[tiab]) OR "Primary care nurse practitioner"[tiab] OR "Primary care nurse practitioners"[tiab] OR ("PCNP"[tiab] AND "Nurse"[tiab]) OR ("PCNP"[tiab] AND "Nurses"[tiab]) OR ("PCNP"[tiab] AND "Nursing"[tiab]) OR "Family nurse practitioner"[tiab] OR "Family nurse practitioners"[tiab] OR ("FNP"[tiab] AND "Nurse"[tiab]) OR ("FNP"[tiab] AND "Nurses"[tiab]) OR ("FNP"[tiab] AND "Nursing"[tiab]) OR "Geriatric nurse practitioner"[tiab] OR "Geriatric nurse practitioners"[tiab] OR "Gerontological nurse practitioner"[tiab] OR "Gerontological nurse practitioners"[tiab] OR ("GNP"[tiab] AND "Nurse"[tiab]) OR ("GNP"[tiab] AND "Nurses"[tiab]) OR ("GNP"[tiab] AND "Nursing"[tiab]) OR "Adult gerontology nurse practitioner"[tiab] OR Adult gerontology nurse practitioners [tiab] OR ("AGNP"[tiab] AND "Nurse"[tiab]) OR ("AGNP"[tiab] AND "Nurses"[tiab]) OR ("AGNP"[tiab] AND "Nursing"[tiab]) OR "Adult gerontology primary care nurse practitioner"[tiab] OR "Adult gerontology primary care nurse practitioners"[tiab] OR ("AGPCNP "[tiab] AND "Nurse"[tiab]) OR ("AGPCNP "[tiab] AND "Nurses"[tiab]) OR ("AGPCNP "[tiab] AND "Nursing"[tiab]) OR "Pediatric Nurse Practitioners"[MH] OR "Adult gerontology acute care nurse practitioner"[tiab] OR "Adult gerontology acute care nurse practitioners"[tiab] OR ("AGACNP"[tiab] AND "Nurse"[tiab]) OR ("AGACNP"[tiab] AND "Nurses"[tiab]) OR ("AGACNP"[tiab] AND "Nursing"[tiab]) OR "Advanced critical care practitioner"[tiab] OR "Advanced critical care practitioners"[tiab] OR ("ACCP"[tiab] AND "Nurse"[tiab]) OR ("ACCP"[tiab] AND "Nurses"[tiab]) OR ("ACCP"[tiab] AND "Nursing"[tiab]) OR "Advanced neonatal nurse practitioner"[tiab] OR "Advanced neonatal nurse practitioners"[tiab] OR ("ANNP"[tiab] AND "Nurse"[tiab]) OR ("ANNP"[tiab] AND "Nurses"[tiab]) OR ("ANNP"[tiab] AND "Nursing"[tiab]) OR "Advanced paediatric nurse practitioner"[tiab] OR Advanced paediatric nurse practitioners[tiab] OR ("cAPNP"[tiab] AND "Nurse"[tiab]) OR ("cAPNP"[tiab] AND "Nurses"[tiab]) OR ("cAPNP"[tiab] AND "Nursing"[tiab]) OR Advanced pediatric nurse practitioner[tiab] OR Advanced pediatric nurse practitioners[tiab] OR Enhanced neonatal nurse practitioner[tiab] OR Enhanced neonatal nurse practitioners[tiab] OR ("ENNP"[tiab] AND "Nurse"[tiab]) OR ("ENNP"[tiab] AND "Nurses"[tiab]) OR ("ENNP"[tiab] AND "Nursing"[tiab]) OR "Oncology nurse practitioner"[tiab] OR "Oncology nurse practitioners"[tiab] OR ("ONP"[tiab] AND "Nurse"[tiab]) OR ("ONP"[tiab] AND "Nurses"[tiab]) OR ("ONP"[tiab] AND "Nursing"[tiab]) OR Pediatric acute care nurse practitioner[tiab] OR Pediatric acute care nurse practitioners [tiab] OR Paediatric acute care nurse practitioner[tiab] OR Paediatric acute care nurse practitioners[tiab] OR ("PNPAC"[tiab] AND "Nurse"[tiab]) OR ("PNPAC"[tiab] AND "Nurses"[tiab]) OR ("PNPAC"[tiab] AND "Nursing"[tiab]) OR "Acute care nurse practitioner"[tiab] OR "Acute care nurse practitioners"[tiab] OR ("ACNP"[tiab] AND "Nurse"[tiab]) OR ("ACNP"[tiab] AND "Nurses"[tiab]) OR ("ACNP"[tiab] AND "Nursing"[tiab]) OR "Adult nurse practitioner"[tiab] OR "Adult nurse practitioners"[tiab] OR "Critical care nurse practitioner"[tiab] OR "Critical care nurse practitioners"[tiab] OR "Emergency nurse practitioner"[tiab] OR "Emergency nurse practitioners"[tiab] OR ("ENP"[tiab] AND "Nursing"[tiab]) OR ("ENP"[tiab] AND "Nurse"[tiab]) OR ("ENP"[tiab] AND "Nurses"[tiab]) OR ("Hospital-based"[tiab] AND "nurse practitioner"[tiab]) OR ("Hospital-based"[tiab] AND "nurse practitioners"[tiab]) OR ("Hospitalised"[tiab] AND "nurse practitioner"[tiab]) OR ("Hospitalised"[tiab] AND "nurse practitioners"[tiab]) OR ("Hospitalized"[tiab] AND "nurse practitioner"[tiab]) OR ("Hospitalized"[tiab] AND "nurse

		practitioners"[tiab]) OR ("Intensive care unit"[tiab] AND "nurse practitioner"[tiab]) OR ("Intensive care unit"[tiab] AND "nurse practitioners"[tiab]) OR ("Intensive care units"[tiab] AND "nurse practitioner"[tiab]) OR ("Intensive care units"[tiab] AND "nurse practitioners"[tiab]) OR ("ICU"[tiab] AND "nurse practitioner"[tiab]) OR ("ICU"[tiab] AND "nurse practitioners"[tiab]) OR ("ICUs"[tiab] AND "nurse practitioner"[tiab]) OR ("ICUs"[tiab] AND "nurse practitioners"[tiab]) OR "Mental Health Nurse Practitioner"[tiab] OR "Mental Health Nurse Practitioners"[tiab] OR ("MHNP"[tiab] AND "Nurse"[tiab]) OR ("MHNP"[tiab] AND "Nurses"[tiab]) OR ("MHNP"[tiab] AND "Nursing"[tiab]) OR "Psychiatric-Mental Health Nurse Practitioner"[tiab] OR "Psychiatric-Mental Health Nurse Practitioners"[tiab] OR "Psychiatric Mental Health Nurse Practitioner"[tiab] OR "Psychiatric Mental Health Nurse Practitioners"[tiab] OR "PMHNP"[tiab] OR "PMHNPs"[tiab] OR "Pediatric nurse practitioner"[tiab] OR "Pediatric nurse practitioners"[tiab] OR "Paediatric nurse practitioner"[tiab] OR "Paediatric nurse practitioners"[tiab] OR ("PNP"[tiab] AND "Nurse"[tiab]) OR ("PNP"[tiab] AND "Nurses"[tiab]) OR ("PNP"[tiab] AND "Nursing"[tiab]) OR "Neonatal nurse practitioner"[tiab] OR "Neonatal nurse practitioners"[tiab] OR ("NNP"[tiab] AND "Nurse"[tiab]) OR ("NNP"[tiab] AND "Nurses"[tiab]) OR ("NNP"[tiab] AND "Nursing"[tiab]))
1.3) Clinical Nurse Specialist	#3	("Nurse Specialists"[MH] OR "Infection Control Practitioners"[MH] OR "Clinical nurse specialist"[tiab] OR "Clinical nurse specialists"[tiab] OR ("CNS"[tiab] AND "nurse"[tiab]) OR ("CNS"[tiab] AND "nurses"[tiab]) OR ("CNS"[tiab] AND "nursing"[tiab]) OR "Clinical nurse consultant"[tiab] OR "Clinical nurse consultants"[tiab] OR "Clinical specialist"[tiab] OR "Clinical specialists"[tiab] OR "Infection control practitioner"[tiab] OR "Infection control practitioners"[tiab] OR "Nurse clinician"[tiab] OR "Nurse clinicians"[tiab] OR "Nurse consultant"[tiab] OR "Nurse consultants"[tiab] OR "Nurse specialist"[tiab] OR "Nurse specialists"[tiab] OR "Specialist nurse"[tiab] OR "Specialist nurses"[tiab] OR Infirmière clinicienne spécialisée[tiab] OR Infirmières cliniciennes spécialisées[tiab] OR Infirmiere clinicienne specialisee[tiab] OR Infirmieres cliniciennes specialisees[tiab] OR "Verpleegkundig specialist"[tiab] OR Verpleegkundig specialist geestelijk gezondheidszorg[tiab] OR Verpleegkundig specialist acute zorg bij somatische aandoeningen[tiab])
	#4	#1 OR #2 OR #3
**Search strategy concept 2 – Search filter** based on the CADTH systematic reviews and meta-analyses search filter [24] and that developed by Lunny et al., 2016 for reviews of systematic reviews [23]	#5	(("systematic"[filter] OR "meta-analysis"[pt] OR "meta-analysis as topic"[mh] OR meta analy*[tw] OR metanaly*[tw] OR metaanaly*[tw] OR met analy*[tw] OR research overview*[tiab] OR "systematic review"[pt] OR "systematic reviews as topic"[mh] OR systematic review*[tiab]) OR ((overview*[ti] OR review[ti] OR synthesis[ti] OR summary[ti] OR cochrane[ti] OR analysis[ti]) AND (reviews[ti] OR meta-analyses[ti] OR articles[ti])) OR (meta-review[tiab] OR meta review[tiab]) OR ((overview*[ti] OR reviews[ti]) AND (systematic[ti] OR cochrane[ti])) OR (reviews[tiab] AND (meta [tiab] OR published[tiab] OR quality[tiab] OR included[tiab] OR summar*[tiab])) OR ("cochrane reviews"[tiab]) OR (evidence[ti] AND (reviews[ti] OR meta-analyses[ti])))
	#6	#4 AND #5
	#7	#6
	#8	#6 Filters: **from 2011/1/1 - 2023/4/3**

2. Ovid Medline

1. exp Advanced Practice Nursing/

2. (advanced practice nurse or advanced practice nurses or advanced practice nursing or (APN and nurse) or (APN and nurses) or (APN and nursing) or advanced nursing practice or advanced nursing practices or Nurse-led or Nurses-led or Nursing-led or Advanced clinical practitioner or Advanced clinical practitioners or Advanced clinical practice or Advanced practice clinician or Advanced practice clinicians or Advanced practitioner or Advanced practitioners or Certified paediatric nurse or Certified paediatric nurses or Certified pediatric nurse or Certified pediatric nurses or Community health nurse or Community health nurses or Community health nursing or Expert nurse or Expert nurses or Expert nursing or (Master and advanced practice nursing) or (Nurse and advanced practice) or (Nurses and advanced practice) or Nurse prescriber or Nurse prescribers or Registered nurse extended class or Registered nurses extended class or Enfermera practica avanzada or Enfermeras practica avanzada or Enfermera de practica avanzada or Enfermeras de practica avanzada or Enfermera gestora de casos or Enfermeras gestoras de casos or Infirmiere de pratique avancee or Infirmieres de pratique avancee or Infirmiere en pratique avancee or Infirmieres en pratique avancee or Pflegeexperte APN or Pflegeexpertin).ti,ab,kf.

3. 1 or 2

4. exp Nurse Practitioners/

5. exp Family Nurse Practitioners/

6. exp Pediatric Nurse Practitioners/

7. (advanced nurse practitioner or advanced nurse practitioners or (ANP and nurse) or (ANP and nurses) or (ANP and nursing) or advanced practice registered nurse or advanced practice registered nurses or advanced practice registered nursing or (APRN and nurse) or (APRN and nurses) or (APRN and nursing) or Nurse practitioner or Nurse practitioners or (NP and nurse) or (NP and nurses) or (NP and nursing) or Advanced registered nurse practitioner or Advanced registered nurse practitioners or Certified nurse practitioner or Certified nurse practitioners or Certified registered nurse practitioner or Certified registered nurse practitioners or Medical nurse practitioner or Medical nurse practitioners or Registered advanced nurse practitioner or Registered advanced nurse practitioners or Registered nurse extended class nurse practitioner or Registered nurse extended class nurse practitioners or Registered nurse practitioner or Registered nurse practitioners or Registered nurse-nurse practitioner or Registered nurse-nurse practitioners or Women's health nurse practitioner or Women's health nurse practitioners or (WHNP and nurse) or (WHNP and nurses) or (WHNP and nursing) or Infirmiere praticienne specialisee or Infirmieres praticiennes specialisees or Family nurse practitioner or Family nurse practitioners or (FNP and Nurse) or (FNP and Nurses) or (FNP and Nursing) or Geriatric nurse practitioner or Geriatric nurse practitioners or Gerontological nurse practitioner or Gerontological nurse practitioners or (GNP and Nurse) or (GNP and Nurses) or (GNP and Nursing) or Adult gerontology nurse practitioner or Adult gerontology nurse practitioners or (AGNP and Nurse) or (AGNP and Nurses) or (AGNP and Nursing) or Adult gerontology primary care nurse practitioner or Adult gerontology primary care nurse practitioners or (AGPCNP and Nurse) or (AGPCNP and Nurses) or (AGPCNP and Nursing) or Primary care nurse practitioner or Primary care nurse practitioners or (PCNP and Nurse) or (PCNP and Nurses) or (PCNP and Nursing) or Primary health care nurse practitioner or Primary health care nurse practitioners or Primary healthcare nurse practitioner or Primary healthcare nurse practitioners or Primary health-care nurse practitioner or Primary health-care nurse practitioners or (PHCNP and Nurse) or (PHCNP and Nurses) or (PHCNP and Nursing) or Adult gerontology acute care nurse practitioner or Adult gerontology acute care nurse

practitioners or (AGACNP and Nurse) or (AGACNP and Nurses) or (AGACNP and Nursing) or Advanced critical care practitioner or Advanced critical care practitioners or (ACCP and Nurse) or (ACCP and Nurses) or (ACCP and Nursing) or Advanced neonatal nurse practitioner or Advanced neonatal nurse practitioners or (ANNP and Nurse) or (ANNP and Nurses) or (ANNP and Nursing) or Advanced paediatric nurse practitioner or Advanced paediatric nurse practitioners or (cAPNP and Nurse) or (cAPNP and Nurses) or (cAPNP and Nursing) or Advanced pediatric nurse practitioner or Advanced pediatric nurse practitioners or Enhanced neonatal nurse practitioner or Enhanced neonatal nurse practitioners or (ENNP and Nurse) or (ENNP and Nurses) or (ENNP and Nursing) or Oncology nurse practitioner or Oncology nurse practitioners or (ONP and Nurse) or (ONP and Nurses) or (ONP and Nursing) or Pediatric acute care nurse practitioner or Pediatric acute care nurse practitioners or Paediatric acute care nurse practitioner or Paediatric acute care nurse practitioners or (PNPAC and Nurse) or (PNPAC and Nurses) or (PNPAC and Nursing) or Acute care nurse practitioner or Acute care nurse practitioners or (ACNP and Nurse) or (ACNP and Nurses) or (ACNP and Nursing) or Adult nurse practitioner or Adult nurse practitioners or Critical care nurse practitioner or Critical care nurse practitioners or Emergency nurse practitioner or Emergency nurse practitioners or (ENP and Nurse) or (ENP and Nurses) or (ENP and Nursing) or (Hospital-based and nurse practitioner) or (Hospital-based and nurse practitioners) or (Hospitalised and nurse practitioner) or (Hospitalised and nurse practitioners) or (Hospitalized and nurse practitioner) or (Hospitalized and nurse practitioners) or (Intensive care unit and nurse practitioner) or (Intensive care unit and nurse practitioners) or (Intensive care units and nurse practitioner) or (Intensive care units and nurse practitioners) or (ICU and nurse practitioner) or (ICU and nurse practitioners) or (ICUs and nurse practitioner) or (ICUs and nurse practitioners) or Mental Health Nurse Practitioner or Mental Health Nurse Practitioners or (MHNP and Nurse) or (MHNP and Nurses) or (MHNP and Nursing) or Psychiatric-Mental Health Nurse Practitioner or Psychiatric-Mental Health Nurse Practitioners or Psychiatric Mental Health Nurse Practitioner or Psychiatric Mental Health Nurse Practitioners or PMHNP or PMHNPs or Pediatric nurse practitioner or Pediatric nurse practitioners or Paediatric nurse practitioner or Paediatric nurse practitioners or (PNP and Nurse) or (PNP and Nurses) or (PNP and Nursing) or Neonatal nurse practitioner or Neonatal nurse practitioners or (NNP and Nurse) or (NNP and Nurses) or (NNP and Nursing)).ti,ab,kf.

8. 4 or 5 or 6 or 7

9. exp Nurse Specialists/

10. exp Infection Control Practitioners/

11. (Clinical nurse specialist or Clinical nurse specialists or (CNS and nurse) or (CNS and nurses) or (CNS and nursing) or Clinical nurse consultant or Clinical nurse consultants or Clinical specialist or Clinical specialists or Infection control practitioner or Infection control practitioners or Nurse clinician or Nurse clinicians or Nurse consultant or Nurse consultants or Nurse specialist or Nurse specialists or Specialist nurse or Specialist nurses or Infirmiere clinicienne specialisee or Infirmieres cliniciennes specialisees or Verpleegkundig specialist or Verpleegkundig specialist geestelijk gezondheidszorg or Verpleegkundig specialist acute zorg bij somatische aandoeningen).ti,ab,kf.

12. 9 or 10 or 11

13. 3 or 8 or 12

14. ((Systematic* adj3 (Review* or Overview*)) or (Methodologic* adj3 (Review* or Overview*))).ti,ab,kf,kw.

15. (systematic review or meta-analysis).pt.

16. meta-analysis/ or systematic review/ or systematic reviews as topic/ or meta-analysis as topic/ or "meta analysis (topic)"/ or "systematic review (topic)"/

17. (Meta Analy* or Metanaly*).ti,ab,kf,kw.

18. (meta-analy* or metaanaly* or systematic review*).mp,hw.

19. (cochrane or evidence report).jw.

20. ((overview$ or review or synthesis or summary or cochrane or analysis) and (reviews or meta-analyses or articles)).ti.

21. (meta-review or metareview).ti,ab.

22. ((overview$ or reviews) and (systematic or cochrane)).ti.

23. (reviews adj2 (meta or published or quality or included or summar$)).ab.

24. cochrane reviews.ab.

25. (evidence and (reviews or meta-analyses)).ti.

26. 14 or 15 or 16 or 17 or 18 or 19 or 20 or 21 or 22 or 23 or 24 or 25

27. 13 and 26

28. limit 27 to yr="2011 -Current"

3. Ovid Healthstar

1. exp Advanced Practice Nursing/

2. (advanced practice nurse or advanced practice nurses or advanced practice nursing or (APN and nurse) or (APN and nurses) or (APN and nursing) or advanced nursing practice or advanced nursing practices or Nurse-led or Nurses-led or Nursing-led or Advanced clinical practitioner or Advanced clinical practitioners or Advanced clinical practice or Advanced practice clinician or Advanced practice clinicians or Advanced practitioner or Advanced practitioners or Certified paediatric nurse or Certified paediatric nurses or Certified pediatric nurse or Certified pediatric nurses or Community health nurse or Community health nurses or Community health nursing or Expert nurse or Expert nurses or Expert nursing or (Master and advanced practice nursing) or (Nurse and advanced practice) or (Nurses and advanced practice) or Nurse prescriber or Nurse prescribers or Registered nurse extended class or Registered nurses extended class or Enfermera practica avanzada or Enfermeras practica avanzada or Enfermera de practica avanzada or Enfermeras de practica avanzada or Enfermera gestora de casos or Enfermeras gestoras de casos or Infirmiere de pratique avancee or Infirmieres de pratique avancee or Infirmiere en pratique avancee or Infirmieres en pratique avancee or Pflegeexperte APN or Pflegeexpertin).ti,ab,kf.

3. 1 or 2

4. exp Nurse Practitioners/

5. exp Family Nurse Practitioners/

6. exp Pediatric Nurse Practitioners/

7. (advanced nurse practitioner or advanced nurse practitioners or (ANP and nurse) or (ANP and nurses) or (ANP and nursing) or advanced practice registered nurse or advanced practice registered nurses or advanced practice registered nursing or (APRN and nurse) or (APRN and nurses) or (APRN and nursing) or Nurse practitioner or Nurse practitioners or (NP and nurse) or (NP and nurses) or (NP and nursing) or Advanced registered nurse practitioner or Advanced registered nurse practitioners or Certified nurse practitioner or Certified nurse practitioners or Certified registered nurse practitioner or Certified registered nurse practitioners or Medical nurse practitioner or Medical nurse practitioners or Registered advanced nurse practitioner or Registered advanced nurse practitioners or Registered nurse extended class nurse practitioner or Registered nurse extended class nurse practitioners or Registered nurse practitioner or Registered nurse practitioners or Registered nurse-nurse practitioner or Registered nurse-nurse practitioners or Women's health nurse practitioner or Women's health nurse practitioners or (WHNP and nurse) or (WHNP and nurses) or (WHNP and nursing) or Infirmiere praticienne specialisee or Infirmieres praticiennes specialisees or Family nurse practitioner or Family nurse practitioners or (FNP and Nurse) or (FNP and Nurses) or (FNP and Nursing) or Geriatric nurse practitioner or Geriatric nurse practitioners or Gerontological nurse practitioner or Gerontological nurse practitioners or (GNP and Nurse) or (GNP and Nurses) or (GNP and Nursing) or Adult gerontology nurse practitioner or Adult gerontology nurse practitioners or (AGNP and Nurse) or (AGNP and Nurses) or (AGNP and Nursing) or Adult gerontology primary care nurse practitioner or Adult gerontology primary care nurse practitioners or (AGPCNP and Nurse) or (AGPCNP and Nurses) or (AGPCNP and Nursing) or Primary care nurse practitioner or Primary care nurse practitioners or (PCNP and Nurse) or (PCNP and Nurses) or (PCNP and Nursing) or Primary health care nurse practitioner or Primary health care nurse practitioners or Primary healthcare nurse practitioner or Primary healthcare nurse practitioners or Primary health-care nurse practitioner or Primary health-care nurse practitioners or (PHCNP and Nurse) or (PHCNP and Nurses) or (PHCNP and Nursing) or Adult gerontology acute care nurse practitioner or Adult gerontology acute care nurse practitioners or (AGACNP and Nurse) or (AGACNP and Nurses) or (AGACNP and Nursing) or Advanced critical

care practitioner or Advanced critical care practitioners or (ACCP and Nurse) or (ACCP and Nurses) or (ACCP and Nursing) or Advanced neonatal nurse practitioner or Advanced neonatal nurse practitioners or (ANNP and Nurse) or (ANNP and Nurses) or (ANNP and Nursing) or Advanced paediatric nurse practitioner or Advanced paediatric nurse practitioners or (cAPNP and Nurse) or (cAPNP and Nurses) or (cAPNP and Nursing) or Advanced pediatric nurse practitioner or Advanced pediatric nurse practitioners or Enhanced neonatal nurse practitioner or Enhanced neonatal nurse practitioners or (ENNP and Nurse) or (ENNP and Nurses) or (ENNP and Nursing) or Oncology nurse practitioner or Oncology nurse practitioners or (ONP and Nurse) or (ONP and Nurses) or (ONP and Nursing) or Pediatric acute care nurse practitioner or Pediatric acute care nurse practitioners or Paediatric acute care nurse practitioner or Paediatric acute care nurse practitioners or (PNPAC and Nurse) or (PNPAC and Nurses) or (PNPAC and Nursing) or Acute care nurse practitioner or Acute care nurse practitioners or (ACNP and Nurse) or (ACNP and Nurses) or (ACNP and Nursing) or Adult nurse practitioner or Adult nurse practitioners or Critical care nurse practitioner or Critical care nurse practitioners or Emergency nurse practitioner or Emergency nurse practitioners or (ENP and Nurse) or (ENP and Nurses) or (ENP and Nursing) or (Hospital-based and nurse practitioner) or (Hospital-based and nurse practitioners) or (Hospitalised and nurse practitioner) or (Hospitalised and nurse practitioners) or (Hospitalized and nurse practitioner) or (Hospitalized and nurse practitioners) or (Intensive care unit and nurse practitioner) or (Intensive care unit and nurse practitioners) or (Intensive care units and nurse practitioner) or (Intensive care units and nurse practitioners) or (ICU and nurse practitioner) or (ICU and nurse practitioners) or (ICUs and nurse practitioner) or (ICUs and nurse practitioners) or Mental Health Nurse Practitioner or Mental Health Nurse Practitioners or (MHNP and Nurse) or (MHNP and Nurses) or (MHNP and Nursing) or Psychiatric-Mental Health Nurse Practitioner or Psychiatric-Mental Health Nurse Practitioners or Psychiatric Mental Health Nurse Practitioner or Psychiatric Mental Health Nurse Practitioners or PMHNP or PMHNPs or Pediatric nurse practitioner or Pediatric nurse practitioners or Paediatric nurse practitioner or Paediatric nurse practitioners or (PNP and Nurse) or (PNP and Nurses) or (PNP and Nursing) or Neonatal nurse practitioner or Neonatal nurse practitioners or (NNP and Nurse) or (NNP and Nurses) or (NNP and Nursing)).ti,ab,kf.

8. 4 or 5 or 6 or 7

9. exp Nurse Specialists/

10. exp Infection Control Practitioners/

11. (Clinical nurse specialist or Clinical nurse specialists or (CNS and nurse) or (CNS and nurses) or (CNS and nursing) or Clinical nurse consultant or Clinical nurse consultants or Clinical specialist or Clinical specialists or Infection control practitioner or Infection control practitioners or Nurse clinician or Nurse clinicians or Nurse consultant or Nurse consultants or Nurse specialist or Nurse specialists or Specialist nurse or Specialist nurses or Infirmiere clinicienne specialisee or Infirmieres cliniciennes specialisees or Verpleegkundig specialist or Verpleegkundig specialist geestelijk gezondheidszorg or Verpleegkundig specialist acute zorg bij somatische aandoeningen).ti,ab,kf.

12. 9 or 10 or 11

13. 3 or 8 or 12

14. ((Systematic* adj3 (Review* or Overview*)) or (Methodologic* adj3 (Review* or Overview*))).ti,ab,kf,kw.

15. (systematic review or meta-analysis).pt.

16. meta-analysis/ or systematic review/ or systematic reviews as topic/ or meta-analysis as topic/ or "meta analysis (topic)"/ or "systematic review (topic)"/

17. (Meta Analy* or Metanaly*).ti,ab,kf,kw.

18. (meta-analy* or metaanaly* or systematic review*).mp,hw.

19. (cochrane or evidence report).jw.

20. ((overview$ or review or synthesis or summary or cochrane or analysis) and (reviews or meta-analyses or articles)).ti.

21. (meta-review or metareview).ti,ab.

22. ((overview$ or reviews) and (systematic or cochrane)).ti.

23. (reviews adj2 (meta or published or quality or included or summar$)).ab.

24. cochrane reviews.ab.

25. (evidence and (reviews or meta-analyses)).ti.

26. 14 or 15 or 16 or 17 or 18 or 19 or 20 or 21 or 22 or 23 or 24 or 25

27. 13 and 26

28. limit 27 to yr="2011 -Current"

4. CINAHL

S36	S35<br>Limiters - Published Date: 20110101-20230431; Exclude MEDLINE records
S35	S18 AND S34
S34	S19 OR S20 OR S21 OR S22 OR S23 OR S24 OR S25 OR S26 OR S27 OR S28 OR S29 OR S30 OR S31 OR S32 OR S33
S33	TI (evidence) AND (TI (reviews OR metaanalyses))
S32	AB (cochrane reviews)
S31	AB (reviews N2 (meta OR published OR quality OR included OR summar*))
S30	(TI (overview* OR reviews)) AND (TI (systematic OR cochrane)))
S29	(TI metareview OR AB metareview)
S28	(TI meta-review OR AB meta-review)
S27	(TI (overview* OR review OR synthesis OR summary OR Cochrane OR analysis)) AND (TI (reviews OR metaanalyses OR articles)))
S26	TX (cochrane OR evidence report)
S25	(SU (meta analy* OR metanaly* OR metaanaly* OR met analy* OR systematic review*))
S24	(TX (meta analy* OR metanaly* OR metaanaly* OR met analy* OR systematic review*))
S23	(MH "Meta Analysis") OR (MH "Systematic Review")
S22	PT (systematic review*) OR PT (meta-analysis) OR PT (meta analysis)
S21	SU ((Systematic* N3 (Review* OR Overview*)) OR (Methodologic* N3 (Review* OR Overview*)))
S20	AB ((Systematic* N3 (Review* OR Overview*)) OR (Methodologic* N3 (Review* OR Overview*)))
S19	TI ((Systematic* N3 (Review* OR Overview*)) OR (Methodologic* N3 (Review* OR Overview*)))
S18	S4 OR S14 OR S17
S17	S15 OR S16
S16	TI (Clinical nurse specialist OR Clinical nurse specialists OR ("CNS" AND nurse) OR ("CNS" AND nurses) OR ("CNS" AND nursing) OR Clinical nurse consultant OR Clinical nurse consultants OR Clinical specialist OR Clinical specialists OR Infection control practitioner OR Infection control practitioners OR Nurse clinician OR Nurse clinicians OR Nurse consultant OR Nurse consultants OR Nurse specialist OR Nurse specialists OR Specialist nurse OR Specialist nurses OR Infirmiere clinicienne specialisee OR Infirmieres cliniciennes specialisees OR Infirmière clinicienne spécialisée OR Infirmières cliniciennes spécialisées OR Verpleegkundig specialist OR Verpleegkundig specialist geestelijk gezondheidszorg OR Verpleegkundig specialist acute zorg bij somatische aandoeningen) OR AB (Clinical nurse specialist OR Clinical nurse specialists OR ("CNS" AND nurse) OR ("CNS" AND nurses) OR ("CNS" AND nursing) OR Clinical nurse consultant OR Clinical nurse consultants OR Clinical specialist OR Clinical specialists OR Infection control practitioner OR Infection control practitioners OR Nurse clinician OR Nurse clinicians OR Nurse consultant OR Nurse consultants OR Nurse specialist OR Nurse specialists OR Specialist nurse OR Specialist nurses OR Infirmiere clinicienne specialisee OR Infirmieres cliniciennes specialisees OR Infirmière clinicienne spécialisée OR Infirmières cliniciennes spécialisées OR Verpleegkundig specialist OR Verpleegkundig specialist geestelijk gezondheidszorg OR Verpleegkundig specialist acute zorg bij somatische aandoeningen)
S15	(MH "Clinical Nurse Specialists") OR (MH "Infection Preventionists")
S14	S5 OR S6 OR S7 OR S8 OR S9 OR S10 OR S11 OR S12 OR S13

S13	TI (advanced nurse practitioner OR advanced nurse practitioners OR ("ANP" AND nurse) OR ("ANP" AND nurses) OR ("ANP" AND nursing) OR advanced practice registered nurse OR advanced practice registered nurses OR advanced practice registered nursing OR ("APRN" AND nurse) OR ("APRN" AND nurses) OR ("APRN" AND nursing) OR Nurse practitioner OR Nurse practitioners OR ("NP" AND nurse) OR ("NP" AND nurses) OR ("NP" AND nursing) OR Advanced registered nurse practitioner OR Advanced registered nurse practitioners OR Certified nurse practitioner OR Certified nurse practitioners OR Certified registered nurse practitioner OR Certified registered nurse practitioners OR Medical nurse practitioner OR Medical nurse practitioners OR Registered advanced nurse practitioner OR Registered advanced nurse practitioners OR Registered nurse extended class nurse practitioner OR Registered nurse extended class nurse practitioners OR "RN(EC)NP" OR Registered nurse practitioner OR Registered nurse practitioners OR Registered nurse-nurse practitioner OR Registered nurse-nurse practitioners OR "RN(NP)" OR Women’s health nurse practitioner OR Women’s health nurse practitioners OR ("WHNP" AND nurse) OR ("WHNP" AND nurses) OR ("WHNP" AND nursing) OR Infirmiere praticienne specialisee OR Infirmieres praticiennes specialisees OR Infirmière praticienne spécialisée OR Infirmières praticiennes spécialisées OR Family nurse practitioner OR Family nurse practitioners OR ("FNP" AND Nurse) OR ("FNP" AND Nurses) OR ("FNP" AND Nursing) OR Geriatric nurse practitioner OR Geriatric nurse practitioners OR Gerontological nurse practitioner OR Gerontological nurse practitioners OR ("GNP" AND Nurse) OR ("GNP" AND Nurses) OR ("GNP" AND Nursing) OR Adult gerontology nurse practitioner OR Adult gerontology nurse practitioners OR ("AGNP" AND Nurse) OR ("AGNP" AND Nurses) OR ("AGNP" AND Nursing) OR Adult gerontology primary care nurse practitioner OR Adult gerontology primary care nurse practitioners OR ("AGPCNP" AND Nurse) OR ("AGPCNP" AND Nurses) OR ("AGPCNP" AND Nursing) OR Primary care nurse practitioner OR Primary care nurse practitioners OR ("PCNP" AND Nurse) OR ("PCNP" AND Nurses) OR ("PCNP" AND Nursing) OR Primary health care nurse practitioner OR Primary health care nurse practitioners OR Primary healthcare nurse practitioner OR Primary healthcare nurse practitioners OR Primary health-care nurse practitioner OR Primary health-care nurse practitioners OR ("PHCNP" AND Nurse) OR ("PHCNP" AND Nurses) OR ("PHCNP" AND Nursing) OR Adult gerontology acute care nurse practitioner OR Adult gerontology acute care nurse practitioners OR ("AGACNP" AND Nurse) OR ("AGACNP" AND Nurses) OR ("AGACNP" AND Nursing) OR Advanced critical care practitioner OR Advanced critical care practitioners OR ("ACCP" AND Nurse) OR ("ACCP" AND Nurses) OR ("ACCP" AND Nursing) OR Advanced neonatal nurse practitioner OR Advanced neonatal nurse practitioners OR ("ANNP" AND Nurse) OR ("ANNP" AND Nurses) OR ("ANNP" AND Nursing) OR Advanced paediatric nurse practitioner OR Advanced paediatric nurse practitioners OR ("cAPNP" AND Nurse) OR ("cAPNP" AND Nurses) OR ("cAPNP" AND Nursing) OR Advanced pediatric nurse practitioner OR Advanced pediatric nurse practitioners OR Enhanced neonatal nurse practitioner OR Enhanced neonatal nurse practitioners OR ("ENNP" AND Nurse) OR ("ENNP" AND Nurses) OR ("ENNP" AND Nursing) OR Oncology nurse practitioner OR Oncology nurse practitioners OR ("ONP" AND Nurse) OR ("ONP" AND Nurses) OR ("ONP" AND Nursing) OR Pediatric acute care nurse practitioner OR Pediatric acute care nurse practitioners OR Paediatric acute care nurse practitioner OR Paediatric acute care nurse practitioners OR ("PNPAC" AND Nurse) OR ("PNPAC" AND Nurses) OR ("PNPAC" AND Nursing) OR Acute care nurse practitioner OR Acute care nurse practitioners OR ("ACNP" AND Nurse) OR ("ACNP" AND Nurses) OR ("ACNP" AND Nursing) OR Adult nurse practitioner OR Adult nurse practitioners OR Critical care nurse practitioner OR Critical care nurse practitioners OR Emergency nurse practitioner OR Emergency nurse practitioners OR ("ENP" AND Nurse) OR ("ENP" AND Nurses) OR ("ENP" AND Nursing) OR (Hospital-based AND nurse practitioner) OR (Hospital-based AND nurse practitioners) OR (Hospitalised AND nurse practitioner) OR (Hospitalised AND nurse practitioners) OR (Hospitalized AND nurse practitioner) OR

	(Hospitalized AND nurse practitioners) OR (Intensive care unit AND nurse practitioner) OR (Intensive care unit AND nurse practitioners) OR (Intensive care units AND nurse practitioner) OR (Intensive care units AND nurse practitioners) OR (ICU AND nurse practitioner) OR (ICU AND nurse practitioners) OR (ICUs AND nurse practitioner) OR (ICUs AND nurse practitioners) OR Mental Health Nurse Practitioner OR Mental Health Nurse Practitioners OR ("MHNP" AND Nurse) OR ("MHNP" AND Nurses) OR ("MHNP" AND Nursing) OR Psychiatric-Mental Health Nurse Practitioner OR Psychiatric-Mental Health Nurse Practitioners OR Psychiatric Mental Health Nurse Practitioner OR Psychiatric Mental Health Nurse Practitioners OR "PMHNP" OR "PMHNPs" OR Pediatric nurse practitioner OR Pediatric nurse practitioners OR Paediatric nurse practitioner OR Paediatric nurse practitioners OR ("PNP" AND Nurse) OR ("PNP" AND Nurses) OR ("PNP" AND Nursing) OR Neonatal nurse practitioner OR Neonatal nurse practitioners OR ("NNP" AND Nurse) OR ("NNP" AND Nurses) OR ("NNP" AND Nursing)) OR AB (advanced nurse practitioner OR advanced nurse practitioners OR ("ANP" AND nurse) OR ("ANP" AND nurses) OR ("ANP" AND nursing) OR advanced practice registered nurse OR advanced practice registered nurses OR advanced practice registered nursing OR ("APRN" AND nurse) OR ("APRN" AND nurses) OR ("APRN" AND nursing) OR Nurse practitioner OR Nurse practitioners OR ("NP" AND nurse) OR ("NP" AND nurses) OR ("NP" AND nursing) OR Advanced registered nurse practitioner OR Advanced registered nurse practitioners OR Certified nurse practitioner OR Certified nurse practitioners OR Certified registered nurse practitioner OR Certified registered nurse practitioners OR Medical nurse practitioner OR Medical nurse practitioners OR Registered advanced nurse practitioner OR Registered advanced nurse practitioners OR Registered nurse extended class nurse practitioner OR Registered nurse extended class nurse practitioners OR "RN(EC)NP" OR Registered nurse practitioner OR Registered nurse practitioners OR Registered nurse-nurse practitioner OR Registered nurse-nurse practitioners OR "RN(NP)" OR Women's health nurse practitioner OR Women's health nurse practitioners OR ("WHNP" AND nurse) OR ("WHNP" AND nurses) OR ("WHNP" AND nursing) OR Infirmiere praticienne specialisee OR Infirmieres praticiennes specialisees OR Infirmière praticienne spécialisée OR Infirmières praticiennes spécialisées OR Family nurse practitioner OR Family nurse practitioners OR ("FNP" AND Nurse) OR ("FNP" AND Nurses) OR ("FNP" AND Nursing) OR Geriatric nurse practitioner OR Geriatric nurse practitioners OR Gerontological nurse practitioner OR Gerontological nurse practitioners OR ("GNP" AND Nurse) OR ("GNP" AND Nurses) OR ("GNP" AND Nursing) OR Adult gerontology nurse practitioner OR Adult gerontology nurse practitioners OR ("AGNP" AND Nurse) OR ("AGNP" AND Nurses) OR ("AGNP" AND Nursing) OR Adult gerontology primary care nurse practitioner OR Adult gerontology primary care nurse practitioners OR ("AGPCNP" AND Nurse) OR ("AGPCNP" AND Nurses) OR ("AGPCNP" AND Nursing) OR Primary care nurse practitioner OR Primary care nurse practitioners OR ("PCNP" AND Nurse) OR ("PCNP" AND Nurses) OR ("PCNP" AND Nursing) OR Primary health care nurse practitioner OR Primary health care nurse practitioners OR Primary healthcare nurse practitioner OR Primary healthcare nurse practitioners OR Primary health-care nurse practitioner OR Primary health-care nurse practitioners OR ("PHCNP" AND Nurse) OR ("PHCNP" AND Nurses) OR ("PHCNP" AND Nursing) OR Adult gerontology acute care nurse practitioner OR Adult gerontology acute care nurse practitioners OR ("AGACNP" AND Nurse) OR ("AGACNP" AND Nurses) OR ("AGACNP" AND Nursing) OR Advanced critical care practitioner OR Advanced critical care practitioners OR ("ACCP" AND Nurse) OR ("ACCP" AND Nurses) OR ("ACCP" AND Nursing) OR Advanced neonatal nurse practitioner OR Advanced neonatal nurse practitioners OR ("ANNP" AND Nurse) OR ("ANNP" AND Nurses) OR ("ANNP" AND Nursing) OR Advanced paediatric nurse practitioner OR Advanced paediatric nurse practitioners OR ("cAPNP" AND Nurse) OR ("cAPNP" AND Nurses) OR ("cAPNP" AND Nursing) OR Advanced pediatric nurse practitioner OR Advanced pediatric nurse practitioners OR Enhanced neonatal nurse practitioner OR Enhanced neonatal nurse practitioners OR ("ENNP" AND Nurse) OR ("ENNP" AND Nurses) OR ("ENNP" AND Nursing) OR

	Oncology nurse practitioner OR Oncology nurse practitioners OR ("ONP" AND Nurse) OR ("ONP" AND Nurses) OR ("ONP" AND Nursing) OR Pediatric acute care nurse practitioner OR Pediatric acute care nurse practitioners OR Paediatric acute care nurse practitioner OR Paediatric acute care nurse practitioners OR ("PNPAC" AND Nurse) OR ("PNPAC" AND Nurses) OR ("PNPAC" AND Nursing) OR Acute care nurse practitioner OR Acute care nurse practitioners OR ("ACNP" AND Nurse) OR ("ACNP" AND Nurses) OR ("ACNP" AND Nursing) OR Adult nurse practitioner OR Adult nurse practitioners OR Critical care nurse practitioner OR Critical care nurse practitioners OR Emergency nurse practitioner OR Emergency nurse practitioners OR ("ENP" AND Nurse) OR ("ENP" AND Nurses) OR ("ENP" AND Nursing) OR (Hospital-based AND nurse practitioner) OR (Hospital-based AND nurse practitioners) OR (Hospitalised AND nurse practitioner) OR (Hospitalised AND nurse practitioners) OR (Hospitalized AND nurse practitioner) OR (Hospitalized AND nurse practitioners) OR (Intensive care unit AND nurse practitioner) OR (Intensive care unit AND nurse practitioners) OR (Intensive care units AND nurse practitioner) OR (Intensive care units AND nurse practitioners) OR (ICU AND nurse practitioner) OR (ICU AND nurse practitioners) OR (ICUs AND nurse practitioner) OR (ICUs AND nurse practitioners) OR Mental Health Nurse Practitioner OR Mental Health Nurse Practitioners OR ("MHNP" AND Nurse) OR ("MHNP" AND Nurses) OR ("MHNP" AND Nursing) OR Psychiatric-Mental Health Nurse Practitioner OR Psychiatric-Mental Health Nurse Practitioners OR Psychiatric Mental Health Nurse Practitioner OR Psychiatric Mental Health Nurse Practitioners OR "PMHNP" OR "PMHNPs" OR Pediatric nurse practitioner OR Pediatric nurse practitioners OR Paediatric nurse practitioner OR Paediatric nurse practitioners OR ("PNP" AND Nurse) OR ("PNP" AND Nurses) OR ("PNP" AND Nursing) OR Neonatal nurse practitioner OR Neonatal nurse practitioners OR ("NNP" AND Nurse) OR ("NNP" AND Nurses) OR ("NNP" AND Nursing))
S12	(MH "Neonatal Nurse Practitioners")
S11	(MH "Emergency Nurse Practitioners")
S10	(MH "Adult Nurse Practitioners")
S9	(MH "Acute Care Nurse Practitioners")
S8	(MH "Pediatric Nurse Practitioners+")
S7	(MH "Gerontologic Nurse Practitioners")
S6	(MH "Family Nurse Practitioners")
S5	(MH "Nurse Practitioners+")
S4	S1 OR S2 OR S3
S3	TI (advanced practice nurse OR advanced practice nurses OR advanced practice nursing OR ("APN" AND nurse) OR ("APN" AND nurses) OR ("APN" AND nursing) OR advanced nursing practice OR advanced nursing practices OR Nurse-led OR Nurses-led OR Nursing-led OR Advanced clinical practitioner OR Advanced clinical practitioners OR Advanced clinical practice OR Advanced practice clinician OR Advanced practice clinicians OR Advanced practitioner OR Advanced practitioners OR Certified paediatric nurse OR Certified paediatric nurses OR Certified pediatric nurse OR Certified pediatric nurses OR Community health nurse OR Community health nurses OR Community health nursing OR Expert nurse OR Expert nurses OR Expert nursing OR Master in advanced practice nursing OR Nurse in advanced practice OR Nurses in advanced practice OR Nurse prescriber OR Nurse prescribers OR Registered nurse extended class OR Registered nurses extended class OR "RN(EC)" OR Enfermera practica avanzada OR Enfermeras practica avanzada OR Enfermera de practica avanzada OR Enfermeras de practica avanzada OR Enfermera gestora de casos OR Enfermeras gestoras de casos OR Infirmière de pratique avancée OR Infirmières de pratique avancée OR Infirmiere de pratique avancee OR Infirmieres de pratique avancee OR Infirmière en pratique avancée OR Infirmières en pratique avancée OR Infirmiere en pratique avancee OR Infirmieres en pratique avancee OR Pflegeexperte APN OR Pflegeexpertin) OR AB (advanced practice nurse OR advanced practice nurses OR advanced practice nursing OR ("APN" AND nurse) OR ("APN" AND nurses) OR ("APN" AND nursing) OR

	advanced nursing practice OR advanced nursing practices OR Nurse-led OR Nurses-led OR Nursing-led OR Advanced clinical practitioner OR Advanced clinical practitioners OR Advanced clinical practice OR Advanced practice clinician OR Advanced practice clinicians OR Advanced practitioner OR Advanced practitioners OR Certified paediatric nurse OR Certified paediatric nurses OR Certified pediatric nurse OR Certified pediatric nurses OR Community health nurse OR Community health nurses OR Community health nursing OR Expert nurse OR Expert nurses OR Expert nursing OR Master in advanced practice nursing OR Nurse in advanced practice OR Nurses in advanced practice OR Nurse prescriber OR Nurse prescribers OR Registered nurse extended class OR Registered nurses extended class OR "RN(EC)" OR Enfermera practica avanzada OR Enfermeras practica avanzada OR Enfermera de practica avanzada OR Enfermeras de practica avanzada OR Enfermera gestora de casos OR Enfermeras gestoras de casos OR Infirmière de pratique avancée OR Infirmières de pratique avancée OR Infirmiere de pratique avancee OR Infirmieres de pratique avancee OR Infirmière en pratique avancée OR Infirmières en pratique avancée OR Infirmiere en pratique avancee OR Infirmieres en pratique avancee OR Pflegeexperte APN OR Pflegeexpertin)
S2	(MH "Advanced Practice Nurses+")
S1	(MH "Advanced Nursing Practice+")

5. Ovid Global Health

1. exp nursing/

2. (advanced practice nurse or advanced practice nurses or advanced practice nursing or (APN and nurse) or (APN and nurses) or (APN and nursing) or advanced nursing practice or advanced nursing practices or Nurse-led or Nurses-led or Nursing-led or Advanced clinical practitioner or Advanced clinical practitioners or Advanced clinical practice or Advanced practice clinician or Advanced practice clinicians or Advanced practitioner or Advanced practitioners or Certified paediatric nurse or Certified paediatric nurses or Certified pediatric nurse or Certified pediatric nurses or Community health nurse or Community health nurses or Community health nursing or Expert nurse or Expert nurses or Expert nursing or (Master and advanced practice nursing) or (Nurse and advanced practice) or (Nurses and advanced practice) or Nurse prescriber or Nurse prescribers or Registered nurse extended class or Registered nurses extended class or Enfermera practica avanzada or Enfermeras practica avanzada or Enfermera de practica avanzada or Enfermeras de practica avanzada or Enfermera gestora de casos or Enfermeras gestoras de casos or Infirmiere de pratique avancee or Infirmieres de pratique avancee or Infirmiere en pratique avancee or Infirmieres en pratique avancee or Pflegeexperte APN or Pflegeexpertin).ti,ab.

3. 1 or 2

4. exp nurses/

5. exp careproviders/

6. (advanced nurse practitioner or advanced nurse practitioners or (ANP and nurse) or (ANP and nurses) or (ANP and nursing) or advanced practice registered nurse or advanced practice registered nurses or advanced practice registered nursing or (APRN and nurse) or (APRN and nurses) or (APRN and nursing) or Nurse practitioner or Nurse practitioners or (NP and nurse) or (NP and nurses) or (NP and nursing) or Advanced registered nurse practitioner or Advanced registered nurse practitioners or Certified nurse practitioner or Certified nurse practitioners or Certified registered nurse practitioner or Certified registered nurse practitioners or Medical nurse practitioner or Medical nurse practitioners or Registered advanced nurse practitioner or Registered advanced nurse practitioners or Registered nurse extended class nurse practitioner or Registered nurse extended class nurse practitioners or Registered nurse practitioner or Registered nurse practitioners or Registered nurse-nurse practitioner or Registered nurse-nurse practitioners or Women's health nurse practitioner or Women's health nurse practitioners or (WHNP and nurse) or (WHNP and nurses) or (WHNP and nursing) or Infirmiere praticienne specialisee or Infirmieres praticiennes specialisees or Family nurse practitioner or Family nurse practitioners or (FNP and Nurse) or (FNP and Nurses) or (FNP and Nursing) or Geriatric nurse practitioner or Geriatric nurse practitioners or Gerontological nurse practitioner or Gerontological nurse practitioners or (GNP and Nurse) or (GNP and Nurses) or (GNP and Nursing) or Adult gerontology nurse practitioner or Adult gerontology nurse practitioners or (AGNP and Nurse) or (AGNP and Nurses) or (AGNP and Nursing) or Adult gerontology primary care nurse practitioner or Adult gerontology primary care nurse practitioners or (AGPCNP and Nurse) or (AGPCNP and Nurses) or (AGPCNP and Nursing) or Primary care nurse practitioner or Primary care nurse practitioners or (PCNP and Nurse) or (PCNP and Nurses) or (PCNP and Nursing) or Primary health care nurse practitioner or Primary health care nurse practitioners or Primary healthcare nurse practitioner or Primary healthcare nurse practitioners or Primary health-care nurse practitioner or Primary health-care nurse practitioners or (PHCNP and Nurse) or (PHCNP and Nurses) or (PHCNP and Nursing) or Adult gerontology acute care nurse practitioner or Adult gerontology acute care nurse practitioners or (AGACNP and Nurse) or (AGACNP and Nurses) or (AGACNP and Nursing) or Advanced critical care practitioner or Advanced critical care practitioners or (ACCP and Nurse) or (ACCP and Nurses) or (ACCP and Nursing) or Advanced neonatal nurse practitioner or Advanced neonatal nurse practitioners or (ANNP and

Nurse) or (ANNP and Nurses) or (ANNP and Nursing) or Advanced paediatric nurse practitioner or Advanced paediatric nurse practitioners or (cAPNP and Nurse) or (cAPNP and Nurses) or (cAPNP and Nursing) or Advanced pediatric nurse practitioner or Advanced pediatric nurse practitioners or Enhanced neonatal nurse practitioner or Enhanced neonatal nurse practitioners or (ENNP and Nurse) or (ENNP and Nurses) or (ENNP and Nursing) or Oncology nurse practitioner or Oncology nurse practitioners or (ONP and Nurse) or (ONP and Nurses) or (ONP and Nursing) or Pediatric acute care nurse practitioner or Pediatric acute care nurse practitioners or Paediatric acute care nurse practitioner or Paediatric acute care nurse practitioners or (PNPAC and Nurse) or (PNPAC and Nurses) or (PNPAC and Nursing) or Acute care nurse practitioner or Acute care nurse practitioners or (ACNP and Nurse) or (ACNP and Nurses) or (ACNP and Nursing) or Adult nurse practitioner or Adult nurse practitioners or Critical care nurse practitioner or Critical care nurse practitioners or Emergency nurse practitioner or Emergency nurse practitioners or (ENP and Nurse) or (ENP and Nurses) or (ENP and Nursing) or (Hospital-based and nurse practitioner) or (Hospital-based and nurse practitioners) or (Hospitalised and nurse practitioner) or (Hospitalised and nurse practitioners) or (Hospitalized and nurse practitioner) or (Hospitalized and nurse practitioners) or (Intensive care unit and nurse practitioner) or (Intensive care unit and nurse practitioners) or (Intensive care units and nurse practitioner) or (Intensive care units and nurse practitioners) or (ICU and nurse practitioner) or (ICU and nurse practitioners) or (ICUs and nurse practitioner) or (ICUs and nurse practitioners) or Mental Health Nurse Practitioner or Mental Health Nurse Practitioners or (MHNP and Nurse) or (MHNP and Nurses) or (MHNP and Nursing) or Psychiatric-Mental Health Nurse Practitioner or Psychiatric-Mental Health Nurse Practitioners or Psychiatric Mental Health Nurse Practitioner or Psychiatric Mental Health Nurse Practitioners or PMHNP or PMHNPs or Pediatric nurse practitioner or Pediatric nurse practitioners or Paediatric nurse practitioner or Paediatric nurse practitioners or (PNP and Nurse) or (PNP and Nurses) or (PNP and Nursing) or Neonatal nurse practitioner or Neonatal nurse practitioners or (NNP and Nurse) or (NNP and Nurses) or (NNP and Nursing)).ti,ab.

7. 4 or 5 or 6

8. (Clinical nurse specialist or Clinical nurse specialists or (CNS and nurse) or (CNS and nurses) or (CNS and nursing) or Clinical nurse consultant or Clinical nurse consultants or Clinical specialist or Clinical specialists or Infection control practitioner or Infection control practitioners or Nurse clinician or Nurse clinicians or Nurse consultant or Nurse consultants or Nurse specialist or Nurse specialists or Specialist nurse or Specialist nurses or Infirmiere clinicienne specialisee or Infirmieres cliniciennes specialisees or Verpleegkundig specialist or Verpleegkundig specialist geestelijk gezondheidszorg or Verpleegkundig specialist acute zorg bij somatische aandoeningen).ti,ab.

9. 3 or 7 or 8

10. ((Systematic* adj3 (Review* or Overview*)) or (Methodologic* adj3 (Review* or Overview*))).af.

11. (systematic review or meta-analysis).af.

12. meta-analysis/ or systematic review/ or systematic reviews as topic/ or meta-analysis as topic/ or "meta analysis (topic)"/ or "systematic review (topic)"/

13. (Meta Analy* or Metanaly*).af.

14. (meta-analy* or metaanaly* or systematic review*).mp,hw.

15. (cochrane or evidence report).jx.

16. ((overview$ or review or synthesis or summary or cochrane or analysis) and (reviews or meta-analyses or articles)).ti.

17. (meta-review or metareview).ti,ab.

18. ((overview$ or reviews) and (systematic or cochrane)).ti.

19. (reviews adj2 (meta or published or quality or included or summar$)).ab.

20. cochrane reviews.ab.

21. (evidence and (reviews or meta-analyses)).ti.

22. 10 or 11 or 12 or 13 or 14 or 15 or 16 or 17 or 18 or 19 or 20 or 21

23. 9 and 22

24. limit 23 to yr="2011 -Current"

6. Web of Science Core Collection

#15 #4 AND #14
Indexes=SCI-EXPANDED, SSCI, A&HCI, CPCI-S, SPCI-SSH Timespan=2011-01-01 to 2023-04-03

#14 #5 OR #6 OR #7 OR #8 OR #9 OR #10 OR #11 OR #12 OR #13
Indexes=SCI-EXPANDED, SSCI, A&HCI, CPCI-S, SPCI-SSH Timespan=2011-01-01 to 2023-04-03

#13 TI=evidence AND (TI=(reviews OR meta-analyses))
Indexes=SCI-EXPANDED, SSCI, A&HCI, CPCI-S, SPCI-SSH Timespan=2011-01-01 to 2023-04-03

#12 AB=cochrane reviews
Indexes=SCI-EXPANDED, SSCI, A&HCI, CPCI-S, SPCI-SSH Timespan=2011-01-01 to 2023-04-03

#11 AB=(reviews NEAR/2 (meta OR published OR quality OR included OR summar*))
Indexes=SCI-EXPANDED, SSCI, A&HCI, CPCI-S, SPCI-SSH Timespan=2011-01-01 to 2023-04-03

#10 (TI=(overview* OR reviews)) AND (TI=(systematic OR cochrane))
Indexes=SCI-EXPANDED, SSCI, A&HCI, CPCI-S, SPCI-SSH Timespan=2011-01-01 to 2023-04-03

#9 (TI=meta-review OR AB=meta-review) OR (TI=metareview OR AB=metareview)
Indexes=SCI-EXPANDED, SSCI, A&HCI, CPCI-S, SPCI-SSH Timespan=2011-01-01 to 2023-04-03

#8 (TI=(overview* OR review OR synthesis OR summary OR cochrane OR analysis) AND TI=(reviews OR meta-analyses OR articles))
Indexes=SCI-EXPANDED, SSCI, A&HCI, CPCI-S, SPCI-SSH Timespan=2011-01-01 to 2023-04-03

#7 TS=(meta analy* OR metanaly* OR metaanaly* OR met analy* OR cochrane OR “evidence report”)
Indexes=SCI-EXPANDED, SSCI, A&HCI, CPCI-S, SPCI-SSH Timespan=2011-01-01 to 2023-04-03

#6 TS=(systematic review* OR meta-analysis)
Indexes=SCI-EXPANDED, SSCI, A&HCI, CPCI-S, SPCI-SSH Timespan=2011-01-01 to 2023-04-03

#5 TS=((Systematic* NEAR/3 (Review* OR Overview*)) OR (Methodologic* NEAR/3 (Review* OR Overview*)))
Indexes=SCI-EXPANDED, SSCI, A&HCI, CPCI-S, SPCI-SSH Timespan=2011-01-01 to 2023-04-03

#4 #1 OR #2 OR #3
Indexes=SCI-EXPANDED, SSCI, A&HCI, CPCI-S, SPCI-SSH Timespan=2011-01-01 to 2023-04-03

#3 TS=("Clinical nurse specialist" OR "Clinical nurse specialists" OR ("CNS" AND "nurse") OR ("CNS" AND "nurses") OR ("CNS" AND "nursing") OR "Clinical nurse consultant" OR "Clinical nurse consultants" OR "Clinical specialist" OR "Clinical specialists" OR "Infection control practitioners" OR "Infection control practitioners" OR "Nurse clinician" OR "Nurse clinicians" OR "Nurse consultant" OR "Nurse consultants" OR "Nurse specialist" OR "Nurse specialists" OR "Specialist nurse" OR "Specialist nurses" OR "Infirmière clinicienne spécialisée" OR "Infirmières cliniciennes spécialisées" OR "Infirmiere clinicienne specialisee" OR "Infirmieres cliniciennes specialisees" OR "Verpleegkundig specialist" OR "Verpleegkundig specialist geestelijk gezondheidszorg" OR "Verpleegkundig specialist acute zorg bij somatische aandoeningen")
Indexes=SCI-EXPANDED, SSCI, A&HCI, CPCI-S, SPCI-SSH Timespan=2011-01-01 to 2023-04-03

#2 TS=("advanced nurse practitioner" OR "advanced nurse practitioners" OR ("ANP" AND "nurse") OR ("ANP" AND "nurses") OR ("ANP" AND "nursing") OR "advanced practice registered nurse" OR "advanced practice registered nurses" OR "advanced practice registered nursing" OR ("APRN" AND "nurse") OR ("APRN" AND "nurses") OR ("APRN" AND "nursing") OR "Nurse practitioner" OR "Nurse practitioners" OR ("NP" AND "nurse") OR ("NP" AND "nurses") OR ("NP" AND "nursing") OR "Advanced registered nurse practitioner" OR "Advanced registered nurse practitioners" OR "Certified nurse practitioner" OR "Certified nurse practitioners" OR "Certified registered nurse practitioner" OR "Certified registered nurse practitioners" OR "Medical nurse practitioner" OR "Medical nurse practitioners" OR "Registered advanced nurse practitioner" OR "Registered advanced nurse practitioners" OR "Registered nurse extended class nurse practitioner" OR "Registered nurse extended class nurse practitioners" OR "Registered nurse practitioner" OR "Registered nurse practitioners" OR "Registered nurse-nurse practitioner" OR "Registered nurse-nurse practitioners" OR "RN(NP)" OR "Women's health nurse practitioner" OR "Women's health nurse practitioners" OR ("WHNP" AND "nurse") OR ("WHNP" AND "nurses") OR ("WHNP" AND "nursing") OR "Infirmière praticienne spécialisée" OR "Infirmières praticiennes spécialisées" OR "Infirmiere praticienne specialisee" OR "Infirmieres praticiennes specialisees" OR "Primary healthcare nurse practitioner" OR "Primary healthcare nurse practitioners" OR "Primary health care nurse practitioner" OR "Primary health care nurse practitioners" OR "Primary health-care nurse practitioner" OR "Primary health-care nurse practitioners" OR ("PHCNP" AND "Nurse") OR ("PHCNP" AND "Nurses") OR ("PHCNP" AND "Nursing") OR "Primary care nurse practitioner" OR "Primary care nurse practitioners" OR ("PCNP" AND "Nurse") OR ("PCNP" AND"Nurses") OR ("PCNP" AND "Nursing") OR "Family nurse practitioner" OR "Family nurse practitioners" OR ("FNP" AND "Nurse") OR ("FNP" AND "Nurses") OR ("FNP" AND "Nursing") OR "Geriatric nurse practitioner" OR "Geriatric nurse practitioners" OR "Gerontological nurse practitioner" OR "Gerontological nurse practitioners" OR ("GNP" AND "Nurse") OR ("GNP" AND "Nurses") OR ("GNP" AND "Nursing") OR "Adult gerontology nurse practitioner" OR "Adult gerontology nurse practitioners " OR ("AGNP" AND "Nurse") OR ("AGNP" AND "Nurses") OR ("AGNP" AND "Nursing") OR "Adult gerontology primary care nurse practitioner" OR "Adult gerontology primary care nurse practitioners" OR ("AGPCNP" AND "Nurse") OR ("AGPCNP" AND "Nurses") OR ("AGPCNP" AND "Nursing") OR "Adult gerontology acute care nurse practitioner" OR "Adult gerontology acute care nurse practitioners" OR ("AGACNP" AND" Nurse") OR ("AGACNP" AND "Nurses") OR ("AGACNP" AND "Nursing") OR "Advanced critical care practitioner" OR "Advanced critical care practitioners" OR ("ACCP" AND "Nurse") OR ("ACCP" AND "Nurses") OR ("ACCP" AND "Nursing") OR "Advanced neonatal nurse practitioner" OR "Advanced neonatal nurse practitioners" OR ("ANNP" AND "Nurse") OR ("ANNP" AND "Nurses") OR ("ANNP" AND "Nursing") OR "Advanced paediatric nurse practitioner" OR "Advanced paediatric nurse practitioners" OR ("cAPNP" AND "Nurse") OR ("cAPNP" AND "Nurses") OR ("cAPNP" AND "Nursing") OR "Advanced pediatric nurse practitioner" OR "Advanced pediatric nurse practitioners" OR "Enhanced neonatal nurse practitioner" OR "Enhanced neonatal nurse practitioners" OR ("ENNP" AND "Nurse") OR ("ENNP" AND "Nurses") OR ("ENNP" AND "Nursing") OR "Oncology nurse practitioner" OR "Oncology nurse practitioners" OR ("ONP" AND "Nurse") OR ("ONP" AND "Nurses") OR ("ONP" AND "Nursing") OR "Pediatric acute care nurse practitioner" OR "Pediatric acute care nurse practitioners" OR "Paediatric acute care nurse practitioner" OR "Paediatric acute care nurse practitioners" OR ("PNPAC" AND "Nurse") OR ("PNPAC" AND "Nurses") OR ("PNPAC" AND "Nursing") OR "Acute care nurse practitioner" OR "Acute care nurse practitioners" OR ("ACNP" AND "Nurse") OR ("ACNP" AND "Nurses") OR ("ACNP" AND "Nursing") OR "Adult nurse practitioner" OR "Adult nurse practitioners" OR "Critical care nurse practitioner" OR "Critical care nurse practitioners" OR "Emergency nurse practitioner" OR "Emergency nurse practitioners" OR ("ENP" AND "Nursing") OR ("ENP" AND "Nurse") OR ("ENP" AND "Nurses") OR ("Hospital-based" AND "nurse practitioner") OR ("Hospital-based" AND "nurse practitioners") OR ("Hospitalised" AND "nurse practitioner") OR ("Hospitalised" AND "nurse practitioners") OR ("Hospitalized" AND "nurse practitioner") OR ("Hospitalized" AND "nurse practitioners") OR ("Intensive care unit" AND "nurse practitioner") OR ("Intensive care unit" AND

"nurse practitioners") OR ("Intensive care units" AND "nurse practitioner") OR ("Intensive care units" AND "nurse practitioners") OR ("ICU" AND "nurse practitioner") OR ("ICU" AND "nurse practitioners") OR ("ICUs" AND "nurse practitioner") OR ("ICUs" AND "nurse practitioners") OR "Mental Health Nurse Practitioner" OR "Mental Health Nurse Practitioners" OR ("MHNP" AND "Nurse") OR ("MHNP" AND "Nurses") OR ("MHNP" AND "Nursing") OR "Psychiatric-Mental Health Nurse Practitioner" OR "Psychiatric-Mental Health Nurse Practitioners" OR "Psychiatric Mental Health Nurse Practitioner" OR "Psychiatric Mental Health Nurse Practitioners" OR "PMHNP" OR "PMHNPs" OR "Pediatric nurse practitioner" OR "Pediatric nurse practitioners" OR "Paediatric nurse practitioner" OR "Paediatric nurse practitioners" OR ("PNP" AND "Nurse") OR ("PNP" AND "Nurses") OR ("PNP" AND"Nursing") OR "Neonatal nurse practitioner" OR "Neonatal nurse practitioners" OR ("NNP" AND "Nurse") OR ("NNP" AND "Nurses") OR ("NNP" AND "Nursing"))
Indexes=SCI-EXPANDED, SSCI, A&HCI, CPCI-S, SPCI-SSH Timespan=2011-01-01 to 2023-04-03

#1 TS=("advanced practice nurse" OR "advanced practice nurses" OR "advanced practice nursing" OR ("APN" AND "nurse") OR ("APN" AND "nurses") OR ("APN" AND "nursing") OR "advanced nursing practice" OR "advanced nursing practices" OR "Nurse-led" OR "Nurses-led" OR "Nursing-led" OR "Advanced clinical practitioner" OR "Advanced clinical practitioners" OR "Advanced clinical practice" OR "Advanced practice clinician" OR "Advanced practice clinicians" OR "Advanced practitioner" OR "Advanced practitioners" OR "Certified paediatric nurse" OR "Certified paediatric nurses" OR "Certified pediatric nurse" OR "Certified pediatric nurses" OR "Community health nurse" OR "Community health nurses" OR "Community health nursing" OR "Expert nurse" OR "Expert nurses" OR "Expert nursing" OR ("Master" AND "advanced practice nursing") OR ("Nurse" AND "advanced practice") OR ("Nurses" AND "advanced practice") OR "Nurse prescriber" OR "Nurse prescribers" OR "Registered nurse extended class" OR "Registered nurses extended class" OR "RN(EC)" OR "Enfermera practica avanzada" OR "Enfermeras practica avanzada" OR "Enfermera de practica avanzada" OR "Enfermeras de practica avanzada" OR "Enfermera gestora de casos" OR "Enfermeras gestoras de casos" OR "Infirmière de pratique avancée" OR "Infirmières de pratique avancée" OR "Infirmiere de pratique avancee" OR "Infirmieres de pratique avancee" OR "Infirmière en pratique avancée" OR "Infirmières en pratique avancée" OR "Infirmiere en pratique avancee" OR "Infirmieres en pratique avancee" OR "Pflegeexperte APN" OR "Pflegeexpertin")
Indexes=SCI-EXPANDED, SSCI, A&HCI, CPCI-S, SPCI-SSH Timespan=2011-01-01 to 2023-04-03

7. OVID Joanna Briggs Institute (JBI) EBP

1. exp Advanced Practice Nursing/

2. Advanced Practice Nursing.af.

3. (advanced practice nurse or advanced practice nurses or advanced practice nursing or (APN and nurse) or (APN and nurses) or (APN and nursing) or advanced nursing practice or advanced nursing practices or Nurse-led or Nurses-led or Nursing-led or Advanced clinical practitioner or Advanced clinical practitioners or Advanced clinical practice or Advanced practice clinician or Advanced practice clinicians or Advanced practitioner or Advanced practitioners or Certified paediatric nurse or Certified paediatric nurses or Certified pediatric nurse or Certified pediatric nurses or Community health nurse or Community health nurses or Community health nursing or Expert nurse or Expert nurses or Expert nursing or (Master and advanced practice nursing) or (Nurse and advanced practice) or (Nurses and advanced practice) or Nurse prescriber or Nurse prescribers or Registered nurse extended class or Registered nurses extended class or Enfermera practica avanzada or Enfermeras practica avanzada or Enfermera de practica avanzada or Enfermeras de practica avanzada or Enfermera gestora de casos or Enfermeras gestoras de casos or Infirmiere de pratique avancee or Infirmieres de pratique avancee or Infirmiere en pratique avancee or Infirmieres en pratique avancee or Pflegeexperte APN or Pflegeexpertin).ti,ab.

4. 1 or 2 or 3

5. exp Nurse Practitioners/

6. Nurse Practitioners.af.

7. exp Family Nurse Practitioners/

8. Family Nurse Practitioners.af.

9. exp Pediatric Nurse Practitioners/

10. Pediatric Nurse Practitioners.af.

11. (advanced nurse practitioner or advanced nurse practitioners or (ANP and nurse) or (ANP and nurses) or (ANP and nursing) or advanced practice registered nurse or advanced practice registered nurses or advanced practice registered nursing or (APRN and nurse) or (APRN and nurses) or (APRN and nursing) or Nurse practitioner or Nurse practitioners or (NP and nurse) or (NP and nurses) or (NP and nursing) or Advanced registered nurse practitioner or Advanced registered nurse practitioners or Certified nurse practitioner or Certified nurse practitioners or Certified registered nurse practitioner or Certified registered nurse practitioners or Medical nurse practitioner or Medical nurse practitioners or Registered advanced nurse practitioner or Registered advanced nurse practitioners or Registered nurse extended class nurse practitioner or Registered nurse extended class nurse practitioners or Registered nurse practitioner or Registered nurse practitioners or Registered nurse-nurse practitioner or Registered nurse-nurse practitioners or Women's health nurse practitioner or Women's health nurse practitioners or (WHNP and nurse) or (WHNP and nurses) or (WHNP and nursing) or Infirmiere praticienne specialisee or Infirmieres praticiennes specialisees or Family nurse practitioner or Family nurse practitioners or (FNP and Nurse) or (FNP and Nurses) or (FNP and Nursing) or Geriatric nurse practitioner or Geriatric nurse practitioners or Gerontological nurse practitioner or Gerontological nurse practitioners or (GNP and Nurse) or (GNP and Nurses) or (GNP and Nursing) or Adult gerontology nurse practitioner or Adult gerontology nurse practitioners or (AGNP and Nurse) or (AGNP and

Nurses) or (AGNP and Nursing) or Adult gerontology primary care nurse practitioner or Adult gerontology primary care nurse practitioners or (AGPCNP and Nurse) or (AGPCNP and Nurses) or (AGPCNP and Nursing) or Primary care nurse practitioner or Primary care nurse practitioners or (PCNP and Nurse) or (PCNP and Nurses) or (PCNP and Nursing) or Primary health care nurse practitioner or Primary health care nurse practitioners or Primary healthcare nurse practitioner or Primary healthcare nurse practitioners or Primary health-care nurse practitioner or Primary health-care nurse practitioners or (PHCNP and Nurse) or (PHCNP and Nurses) or (PHCNP and Nursing) or Adult gerontology acute care nurse practitioner or Adult gerontology acute care nurse practitioners or (AGACNP and Nurse) or (AGACNP and Nurses) or (AGACNP and Nursing) or Advanced critical care practitioner or Advanced critical care practitioners or (ACCP and Nurse) or (ACCP and Nurses) or (ACCP and Nursing) or Advanced neonatal nurse practitioner or Advanced neonatal nurse practitioners or (ANNP and Nurse) or (ANNP and Nurses) or (ANNP and Nursing) or Advanced paediatric nurse practitioner or Advanced paediatric nurse practitioners or (cAPNP and Nurse) or (cAPNP and Nurses) or (cAPNP and Nursing) or Advanced pediatric nurse practitioner or Advanced pediatric nurse practitioners or Enhanced neonatal nurse practitioner or Enhanced neonatal nurse practitioners or (ENNP and Nurse) or (ENNP and Nurses) or (ENNP and Nursing) or Oncology nurse practitioner or Oncology nurse practitioners or (ONP and Nurse) or (ONP and Nurses) or (ONP and Nursing) or Pediatric acute care nurse practitioner or Pediatric acute care nurse practitioners or Paediatric acute care nurse practitioner or Paediatric acute care nurse practitioners or (PNPAC and Nurse) or (PNPAC and Nurses) or (PNPAC and Nursing) or Acute care nurse practitioner or Acute care nurse practitioners or (ACNP and Nurse) or (ACNP and Nurses) or (ACNP and Nursing) or Adult nurse practitioner or Adult nurse practitioners or Critical care nurse practitioner or Critical care nurse practitioners or Emergency nurse practitioner or Emergency nurse practitioners or (ENP and Nurse) or (ENP and Nurses) or (ENP and Nursing) or (Hospital-based and nurse practitioner) or (Hospital-based and nurse practitioners) or (Hospitalised and nurse practitioner) or (Hospitalised and nurse practitioners) or (Hospitalized and nurse practitioner) or (Hospitalized and nurse practitioners) or (Intensive care unit and nurse practitioner) or (Intensive care unit and nurse practitioners) or (Intensive care units and nurse practitioner) or (Intensive care units and nurse practitioners) or (ICU and nurse practitioner) or (ICU and nurse practitioners) or (ICUs and nurse practitioner) or (ICUs and nurse practitioners) or Mental Health Nurse Practitioner or Mental Health Nurse Practitioners or (MHNP and Nurse) or (MHNP and Nurses) or (MHNP and Nursing) or Psychiatric-Mental Health Nurse Practitioner or Psychiatric-Mental Health Nurse Practitioners or Psychiatric Mental Health Nurse Practitioner or Psychiatric Mental Health Nurse Practitioners or PMHNP or PMHNPs or Pediatric nurse practitioner or Pediatric nurse practitioners or Paediatric nurse practitioner or Paediatric nurse practitioners or (PNP and Nurse) or (PNP and Nurses) or (PNP and Nursing) or Neonatal nurse practitioner or Neonatal nurse practitioners or (NNP and Nurse) or (NNP and Nurses) or (NNP and Nursing)).ti,ab.

12. 5 or 6 or 7 or 8 or 9 or 10 or 11

13. exp Nurse Specialists/

14. Nurse Specialists.af.

15. exp Infection Control Practitioners/

16. Infection Control Practitioners.af.

17. (Clinical nurse specialist or Clinical nurse specialists or (CNS and nurse) or (CNS and nurses) or (CNS and nursing) or Clinical nurse consultant or Clinical nurse consultants or Clinical specialist or Clinical specialists or Infection control practitioner or Infection control practitioners or Nurse clinician or Nurse clinicians or Nurse consultant or Nurse consultants or Nurse specialist or Nurse specialists or Specialist nurse or Specialist nurses or Infirmiere clinicienne specialisee or Infirmieres cliniciennes specialisees or Verpleegkundig specialist or

Verpleegkundig specialist geestelijk gezondheidszorg or Verpleegkundig specialist acute zorg bij somatische aandoeningen).ti,ab.

18. 13 or 14 or 15 or 16 or 17

19. 4 or 12 or 18

20. ((Systematic* adj3 (Review* or Overview*)) or (Methodologic* adj3 (Review* or Overview*))).af.

21. (systematic review or meta-analysis).pt.

22. meta-analysis/ or systematic review/ or systematic reviews as topic/ or meta-analysis as topic/ or "meta analysis (topic)"/ or "systematic review (topic)"/

23. (Meta Analy* or Metanaly*).af.

24. (meta-analy* or metaanaly* or systematic review*).mp,hw.

25. (cochrane or evidence report).jw.

26. ((overview$ or review or synthesis or summary or cochrane or analysis) and (reviews or meta-analyses or articles)).ti.

27. (meta-review or metareview).ti,ab.

28. ((overview$ or reviews) and (systematic or cochrane)).ti.

29. (reviews adj2 (meta or published or quality or included or summar$)).ab.

30. cochrane reviews.ab.

31. (evidence and (reviews or meta-analyses)).ti.

32. 20 or 21 or 22 or 23 or 24 or 25 or 26 or 27 or 28 or 29 or 30 or 31

33. 19 and 32

34. limit 33 to yr="2011 -Current"

8. Ovid Embase

1. exp advanced practice nursing/

2. exp advanced practice nurse/

3. (advanced practice nurse or advanced practice nurses or advanced practice nursing or (APN and nurse) or (APN and nurses) or (APN and nursing) or advanced nursing practice or advanced nursing practices or Nurse-led or Nurses-led or Nursing-led or Advanced clinical practitioner or Advanced clinical practitioners or Advanced clinical practice or Advanced practice clinician or Advanced practice clinicians or Advanced practitioner or Advanced practitioners or Certified paediatric nurse or Certified paediatric nurses or Certified pediatric nurse or Certified pediatric nurses or Community health nurse or Community health nurses or Community health nursing or Expert nurse or Expert nurses or Expert nursing or (Master and advanced practice nursing) or (Nurse and advanced practice) or (Nurses and advanced practice) or Nurse prescriber or Nurse prescribers or Registered nurse extended class or Registered nurses extended class or Enfermera practica avanzada or Enfermeras practica avanzada or Enfermera de practica avanzada or Enfermeras de practica avanzada or Enfermera gestora de casos or Enfermeras gestoras de casos or Infirmiere de pratique avancee or Infirmieres de pratique avancee or Infirmiere en pratique avancee or Infirmieres en pratique avancee or Pflegeexperte APN or Pflegeexpertin).ti,ab,kf.

4. 1 or 2 or 3

5. exp nurse practitioner/

6. exp family nurse practitioner/

7. exp gerontologic nurse practitioner/

8. exp acute care nurse practitioner/

9. exp adult nurse practitioner/

10. exp emergency nurse practitioner/

11. exp neonatal nurse practitioner/

12. exp pediatric nurse practitioner/

13. (advanced nurse practitioner or advanced nurse practitioners or (ANP and nurse) or (ANP and nurses) or (ANP and nursing) or advanced practice registered nurse or advanced practice registered nurses or advanced practice registered nursing or (APRN and nurse) or (APRN and nurses) or (APRN and nursing) or Nurse practitioner or Nurse practitioners or (NP and nurse) or (NP and nurses) or (NP and nursing) or Advanced registered nurse practitioner or Advanced registered nurse practitioners or Certified nurse practitioner or Certified nurse practitioners or Certified registered nurse practitioner or Certified registered nurse practitioners or Medical nurse practitioner or Medical nurse practitioners or Registered advanced nurse practitioner or Registered advanced nurse practitioners or Registered nurse extended class nurse practitioner or Registered nurse extended class nurse practitioners or Registered nurse practitioner or Registered nurse practitioners or Registered nurse-nurse practitioner or Registered nurse-nurse practitioners or Women's health nurse practitioner or Women's health nurse practitioners or (WHNP and nurse) or (WHNP and nurses) or (WHNP and nursing) or Infirmiere praticienne specialisee or Infirmieres praticiennes specialisees or Family

nurse practitioner or Family nurse practitioners or (FNP and Nurse) or (FNP and Nurses) or (FNP and Nursing) or Geriatric nurse practitioner or Geriatric nurse practitioners or Gerontological nurse practitioner or Gerontological nurse practitioners or (GNP and Nurse) or (GNP and Nurses) or (GNP and Nursing) or Adult gerontology nurse practitioner or Adult gerontology nurse practitioners or (AGNP and Nurse) or (AGNP and Nurses) or (AGNP and Nursing) or Adult gerontology primary care nurse practitioner or Adult gerontology primary care nurse practitioners or (AGPCNP and Nurse) or (AGPCNP and Nurses) or (AGPCNP and Nursing) or Primary care nurse practitioner or Primary care nurse practitioners or (PCNP and Nurse) or (PCNP and Nurses) or (PCNP and Nursing) or Primary health care nurse practitioner or Primary health care nurse practitioners or Primary healthcare nurse practitioner or Primary healthcare nurse practitioners or Primary health-care nurse practitioner or Primary health-care nurse practitioners or (PHCNP and Nurse) or (PHCNP and Nurses) or (PHCNP and Nursing) or Adult gerontology acute care nurse practitioner or Adult gerontology acute care nurse practitioners or (AGACNP and Nurse) or (AGACNP and Nurses) or (AGACNP and Nursing) or Advanced critical care practitioner or Advanced critical care practitioners or (ACCP and Nurse) or (ACCP and Nurses) or (ACCP and Nursing) or Advanced neonatal nurse practitioner or Advanced neonatal nurse practitioners or (ANNP and Nurse) or (ANNP and Nurses) or (ANNP and Nursing) or Advanced paediatric nurse practitioner or Advanced paediatric nurse practitioners or (cAPNP and Nurse) or (cAPNP and Nurses) or (cAPNP and Nursing) or Advanced pediatric nurse practitioner or Advanced pediatric nurse practitioners or Enhanced neonatal nurse practitioner or Enhanced neonatal nurse practitioners or (ENNP and Nurse) or (ENNP and Nurses) or (ENNP and Nursing) or Oncology nurse practitioner or Oncology nurse practitioners or (ONP and Nurse) or (ONP and Nurses) or (ONP and Nursing) or Pediatric acute care nurse practitioner or Pediatric acute care nurse practitioners or Paediatric acute care nurse practitioner or Paediatric acute care nurse practitioners or (PNPAC and Nurse) or (PNPAC and Nurses) or (PNPAC and Nursing) or Acute care nurse practitioner or Acute care nurse practitioners or (ACNP and Nurse) or (ACNP and Nurses) or (ACNP and Nursing) or Adult nurse practitioner or Adult nurse practitioners or Critical care nurse practitioner or Critical care nurse practitioners or Emergency nurse practitioner or Emergency nurse practitioners or (ENP and Nurse) or (ENP and Nurses) or (ENP and Nursing) or (Hospital-based and nurse practitioner) or (Hospital-based and nurse practitioners) or (Hospitalised and nurse practitioner) or (Hospitalised and nurse practitioners) or (Hospitalized and nurse practitioner) or (Hospitalized and nurse practitioners) or (Intensive care unit and nurse practitioner) or (Intensive care unit and nurse practitioners) or (Intensive care units and nurse practitioner) or (Intensive care units and nurse practitioners) or (ICU and nurse practitioner) or (ICU and nurse practitioners) or (ICUs and nurse practitioner) or (ICUs and nurse practitioners) or Mental Health Nurse Practitioner or Mental Health Nurse Practitioners or (MHNP and Nurse) or (MHNP and Nurses) or (MHNP and Nursing) or Psychiatric-Mental Health Nurse Practitioner or Psychiatric-Mental Health Nurse Practitioners or Psychiatric Mental Health Nurse Practitioner or Psychiatric Mental Health Nurse Practitioners or PMHNP or PMHNPs or Pediatric nurse practitioner or Pediatric nurse practitioners or Paediatric nurse practitioner or Paediatric nurse practitioners or (PNP and Nurse) or (PNP and Nurses) or (PNP and Nursing) or Neonatal nurse practitioner or Neonatal nurse practitioners or (NNP and Nurse) or (NNP and Nurses) or (NNP and Nursing)).ti,ab,kf.

14. 5 or 6 or 7 or 8 or 9 or 10 or 11 or 12 or 13

15. exp clinical nurse specialist/

16. exp nurse specialist/

17. exp infection control practitioner/

18. (Clinical nurse specialist or Clinical nurse specialists or (CNS and nurse) or (CNS and nurses) or (CNS and nursing) or Clinical nurse consultant or Clinical nurse consultants or Clinical specialist or Clinical specialists or Infection control practitioner or Infection control practitioners or Nurse clinician or Nurse clinicians or Nurse consultant or Nurse consultants or Nurse specialist or Nurse specialists or Specialist nurse or Specialist nurses

or Infirmiere clinicienne specialisee or Infirmieres cliniciennes specialisees or Verpleegkundig specialist or Verpleegkundig specialist geestelijk gezondheidszorg or Verpleegkundig specialist acute zorg bij somatische aandoeningen).ti,ab,kf.

19. 15 or 16 or 17 or 18

20. 4 or 14 or 19

21. ((Systematic* adj3 (Review* or Overview*)) or (Methodologic* adj3 (Review* or Overview*))).ti,ab,kf,kw.

22. (systematic review or meta-analysis).mp.

23. meta-analysis/ or systematic review/ or systematic reviews as topic/ or meta-analysis as topic/ or "meta analysis (topic)"/ or "systematic review (topic)"/

24. (Meta Analy* or Metanaly*).ti,ab,kf,kw.

25. (meta-analy* or metaanaly* or systematic review*).mp,hw.

26. (cochrane or evidence report).jw.

27. ((overview$ or review or synthesis or summary or cochrane or analysis) and (reviews or meta-analyses or articles)).ti.

28. (meta-review or metareview).ti,ab.

29. ((overview$ or reviews) and (systematic or cochrane)).ti.

30. (reviews adj2 (meta or published or quality or included or summar$)).ab.

31. cochrane reviews.ab.

32. (evidence and (reviews or meta-analyses)).ti.

33. 21 or 22 or 23 or 24 or 25 or 26 or 27 or 28 or 29 or 30 or 31 or 32

34. 20 and 33

35. limit 34 to yr="2011 -Current"

9. Cochrane Library

#1 MeSH descriptor: [Advanced Practice Nursing] explode all trees

#2 ("advanced practice nurse" OR "advanced practice nurses" OR "advanced practice nursing" OR ("APN" AND "nurse") OR ("APN" AND "nurses") OR ("APN" AND "nursing") OR "advanced nursing practice" OR "advanced nursing practices" OR "Nurse-led" OR "Nurses-led" OR "Nursing-led" OR "Advanced clinical practitioner" OR "Advanced clinical practitioners" OR "Advanced clinical practice" OR "Advanced practice clinician" OR "Advanced practice clinicians" OR "Advanced practitioner" OR "Advanced practitioners" OR "Certified paediatric nurse" OR "Certified paediatric nurses" OR "Certified pediatric nurse" OR "Certified pediatric nurses" OR "Community health nurse" OR "Community health nurses" OR "Community health nursing" OR "Expert nurse" OR "Expert nurses" OR "Expert nursing" OR ("Master" AND "advanced practice nursing") OR ("Nurse" AND "advanced practice") OR ("Nurses" AND "advanced practice") OR "Nurse prescriber" OR "Nurse prescribers" OR "Registered nurse extended class" OR "Registered nurses extended class" OR "Enfermera practica avanzada" OR "Enfermeras practica avanzada" OR "Enfermera de practica avanzada" OR "Enfermeras de practica avanzada" OR "Enfermera gestora de casos" OR "Enfermeras gestoras de casos" OR "Infirmière de pratique avancée" OR "Infirmières de pratique avancée" OR "Infirmiere de pratique avancee" OR "Infirmieres de pratique avancee" OR "Infirmière en pratique avancée" OR "Infirmières en pratique avancée" OR "Infirmiere en pratique avancee" OR "Infirmieres en pratique avancee" OR "Pflegeexperte APN" OR "Pflegeexpertin"):ti,ab,kw

#3 #1 or #2

#4 MeSH descriptor: [Nurse Practitioners] explode all trees

#5 MeSH descriptor: [Family Nurse Practitioners] explode all trees

#6 MeSH descriptor: [Pediatric Nurse Practitioners] explode all trees 3

#7 ("advanced nurse practitioner" OR "advanced nurse practitioners" OR ("ANP" AND "nurse") OR ("ANP" AND "nurses") OR ("ANP" AND "nursing") OR "advanced practice registered nurse" OR "advanced practice registered nurses" OR "advanced practice registered nursing" OR ("APRN" AND "nurse") OR ("APRN" AND "nurses") OR ("APRN" AND "nursing") OR "Nurse practitioner" OR "Nurse practitioners" OR ("NP" AND "nurse") OR ("NP" AND "nurses") OR ("NP" AND "nursing") OR "Advanced registered nurse practitioner" OR "Advanced registered nurse practitioners" OR "Certified nurse practitioner" OR "Certified nurse practitioners" OR "Certified registered nurse practitioner" OR "Certified registered nurse practitioners" OR "Medical nurse practitioner" OR "Medical nurse practitioners" OR "Registered advanced nurse practitioner" OR "Registered advanced nurse practitioners" OR "Registered nurse extended class nurse practitioner" OR "Registered nurse extended class nurse practitioners" OR "Registered nurse practitioner" OR "Registered nurse practitioners" OR "Registered nurse-nurse practitioner" OR "Registered nurse-nurse practitioners" OR "Women’s health nurse practitioner" OR "Women’s health nurse practitioners" OR ("WHNP" AND "nurse") OR ("WHNP" AND "nurses") OR ("WHNP" AND "nursing") OR "Infirmière praticienne spécialisée" OR "Infirmières praticiennes spécialisées" OR "Infirmiere praticienne specialisee" OR "Infirmieres praticiennes specialisees" OR "Primary healthcare nurse practitioner" OR "Primary healthcare nurse practitioners" OR "Primary health care nurse practitioner" OR "Primary health care nurse practitioners" OR "Primary health-care nurse practitioner" OR "Primary health-care nurse practitioners" OR ("PHCNP" AND "Nurse") OR ("PHCNP" AND "Nurses") OR ("PHCNP" AND "Nursing") OR "Primary care nurse practitioner" OR "Primary care nurse practitioners" OR "Family nurse practitioner" OR "Family nurse practitioners" OR

("FNP" AND "Nurse") OR ("FNP" AND "Nurses") OR ("FNP" AND "Nursing") OR "Geriatric nurse practitioner" OR "Geriatric nurse practitioners" OR "Gerontological nurse practitioner" OR "Gerontological nurse practitioners" OR ("GNP" AND "Nurse") OR ("GNP" AND "Nurses") OR ("GNP" AND "Nursing") OR "Adult gerontology nurse practitioner" OR "Adult gerontology nurse practitioners " OR ("AGNP" AND "Nurse") OR ("AGNP" AND "Nurses") OR ("AGNP" AND "Nursing") OR "Adult gerontology primary care nurse practitioner" OR "Adult gerontology primary care nurse practitioners" OR ("AGPCNP" AND "Nurse") OR ("AGPCNP" AND "Nurses") OR ("AGPCNP" AND "Nursing") OR "Adult gerontology acute care nurse practitioner" OR "Adult gerontology acute care nurse practitioners" OR "Advanced critical care practitioner" OR "Advanced critical care practitioners" OR ("ACCP" AND "Nurse") OR ("ACCP" AND "Nurses") OR ("ACCP" AND "Nursing") OR "Advanced neonatal nurse practitioner" OR "Advanced neonatal nurse practitioners" OR ("ANNP" AND "Nurse") OR ("ANNP" AND "Nurses") OR ("ANNP" AND "Nursing") OR "Advanced paediatric nurse practitioner" OR "Advanced paediatric nurse practitioners" OR ("cAPNP" AND "Nurse") OR ("cAPNP" AND "Nurses") OR ("cAPNP" AND "Nursing") OR "Advanced pediatric nurse practitioner" OR "Advanced pediatric nurse practitioners" OR "Enhanced neonatal nurse practitioner" OR "Enhanced neonatal nurse practitioners" OR ("ENNP" AND "Nurse") OR ("ENNP" AND "Nurses") OR ("ENNP" AND "Nursing") OR "Oncology nurse practitioner" OR "Oncology nurse practitioners" OR ("ONP" AND "Nurse") OR ("ONP" AND "Nurses") OR ("ONP" AND "Nursing") OR "Pediatric acute care nurse practitioner" OR "Pediatric acute care nurse practitioners" OR "Paediatric acute care nurse practitioner" OR "Paediatric acute care nurse practitioners" OR ("PNPAC" AND "Nurse") OR ("PNPAC" AND "Nurses") OR ("PNPAC" AND "Nursing") OR "Acute care nurse practitioner" OR "Acute care nurse practitioners" OR ("ACNP" AND "Nurse") OR ("ACNP" AND "Nurses") OR ("ACNP" AND "Nursing") OR "Adult nurse practitioner" OR "Adult nurse practitioners" OR "Critical care nurse practitioner" OR "Critical care nurse practitioners" OR "Emergency nurse practitioner" OR "Emergency nurse practitioners" OR ("ENP" AND "Nursing") OR ("ENP" AND "Nurse") OR ("ENP" AND "Nurses") OR ("Hospital-based" AND "nurse practitioner") OR ("Hospital-based" AND "nurse practitioners") OR ("Hospitalised" AND "nurse practitioner") OR ("Hospitalised" AND "nurse practitioners") OR ("Hospitalized" AND "nurse practitioner") OR ("Hospitalized" AND "nurse practitioners") OR ("Intensive care unit" AND "nurse practitioner") OR ("Intensive care unit" AND "nurse practitioners") OR ("Intensive care units" AND "nurse practitioner") OR ("Intensive care units" AND "nurse practitioners") OR ("ICU" AND "nurse practitioner") OR ("ICU" AND "nurse practitioners") OR ("ICUs" AND "nurse practitioner") OR ("ICUs" AND "nurse practitioners") OR "Mental Health Nurse Practitioner" OR "Mental Health Nurse Practitioners" OR ("MHNP" AND "Nurse") OR ("MHNP" AND "Nurses") OR ("MHNP" AND "Nursing") OR "Psychiatric-Mental Health Nurse Practitioner" OR "Psychiatric-Mental Health Nurse Practitioners" OR "Psychiatric Mental Health Nurse Practitioner" OR "Psychiatric Mental Health Nurse Practitioners" OR "PMHNP" OR "PMHNPs" OR "Pediatric nurse practitioner" OR "Pediatric nurse practitioners" OR "Paediatric nurse practitioner" OR "Paediatric nurse practitioners" OR "Neonatal nurse practitioner" OR "Neonatal nurse practitioners" OR ("NNP" AND "Nurse") OR ("NNP" AND "Nurses") OR ("NNP" AND "Nursing")):ti,ab,kw

#8 #4 or #5 or #6 or #7

#9 MeSH descriptor: [Nurse Specialists] explode all trees

#10 MeSH descriptor: [Infection Control Practitioners] explode all trees

#11 ("Clinical nurse specialist" OR "Clinical nurse specialists" OR ("CNS" AND "nurse") OR ("CNS" AND "nurses") OR ("CNS" AND "nursing") OR "Clinical nurse consultant" OR "Clinical nurse consultants" OR "Clinical specialist" OR "Clinical specialists" OR "Infection control practitioners" OR "Infection control

practitioners" OR "Nurse clinician" OR "Nurse clinicians" OR "Nurse consultant" OR "Nurse consultants" OR "Nurse specialist" OR "Nurse specialists" OR "Specialist nurse" OR "Specialist nurses" OR "Infirmière clinicienne spécialisée" OR "Infirmières cliniciennes spécialisées" OR "Infirmiere clinicienne specialisee" OR "Infirmieres cliniciennes specialisees" OR "Verpleegkundig specialist" OR "Verpleegkundig specialist geestelijk gezondheidszorg" OR "Verpleegkundig specialist acute zorg bij somatische aandoeningen"):ti,ab,kw

#12 #9 or #10 or #11

#13 #3 or #8 or #12

#14 ((Systematic* NEAR/3 (Review* or Overview*)) or (Methodologic* NEAR/3 (Review* or Overview*))):ti,ab,kw

#15 (systematic review or meta-analysis):pt

#16 MeSH descriptor: [Meta-Analysis as Topic] explode all trees

#17 MeSH descriptor: [Systematic Reviews as Topic] explode all trees

#18 (Meta Analy* or Metanaly*):ti,ab,kw

#19 (meta-analy* or metaanaly* or systematic review*):ti,ab,kw

#20 (evidence report):ti,ab,kw

#21 ((overview* or review or synthesis or summary or analysis) and (reviews or meta-analyses or articles)):ti

#22 (meta-review or metareview):ti,ab

#23 ((overview* or reviews) and (systematic)):ti

#24 (reviews NEAR/2 (meta or published or quality or included or summar*)):ab

#25 (evidence and (reviews or meta-analyses)):ti

#26 #14 or #15 or #16 or #17 or #18 or #19 or #20 or #21 or #22 or #23 or #24 or #25

#27 #13 and #26

#28 #27 with Cochrane Library publication date Between Jan 2011 and Apr 2023, in Cochrane Reviews, Cochrane Protocols, Special Collections

10. DARE

1. MeSH DESCRIPTOR Advanced Practice Nursing EXPLODE ALL TREES IN DARE

2. ((advanced practice nurse or advanced practice nurses or advanced practice nursing or (APN and nurse) or (APN and nurses) or (APN and nursing) or advanced nursing practice or advanced nursing practices or Nurse-led or Nurses-led or Nursing-led or Advanced clinical practitioner or Advanced clinical practitioners or Advanced clinical practice or Advanced practice clinician or Advanced practice clinicians or Advanced practitioner or Advanced practitioners or Certified paediatric nurse or Certified paediatric nurses or Certified pediatric nurse or Certified pediatric nurses or Community health nurse or Community health nurses or Community health nursing or Expert nurse or Expert nurses or Expert nursing or (Master and advanced practice nursing) or (Nurse and advanced practice) or (Nurses and advanced practice) or Nurse prescriber or Nurse prescribers or Registered nurse extended class or Registered nurses extended class or Enfermera practica avanzada or Enfermeras practica avanzada or Enfermera de practica avanzada or Enfermeras de practica avanzada or Enfermera gestora de casos or Enfermeras gestoras de casos or Infirmiere de pratique avancee or Infirmieres de pratique avancee or Infirmiere en pratique avancee or Infirmieres en pratique avancee or Pflegeexperte APN or Pflegeexpertin)) IN DARE

3. #1 OR #2

4. MeSH DESCRIPTOR Nurse Practitioners EXPLODE ALL TREES IN DARE

5. MeSH DESCRIPTOR Family Nurse Practitioners EXPLODE ALL TREES IN DARE

6. MeSH DESCRIPTOR Pediatric Nurse Practitioners EXPLODE ALL TREES IN DARE

7. ((advanced nurse practitioner or advanced nurse practitioners or (ANP and nurse) or (ANP and nurses) or (ANP and nursing) or advanced practice registered nurse or advanced practice registered nurses or advanced practice registered nursing or (APRN and nurse) or (APRN and nurses) or (APRN and nursing) or Nurse practitioner or Nurse practitioners or (NP and nurse) or (NP and nurses) or (NP and nursing) or Advanced registered nurse practitioner or Advanced registered nurse practitioners or Certified nurse practitioner or Certified nurse practitioners or Certified registered nurse practitioner or Certified registered nurse practitioners or Medical nurse practitioner or Medical nurse practitioners or Registered advanced nurse practitioner or Registered advanced nurse practitioners or Registered nurse extended class nurse practitioner or Registered nurse extended class nurse practitioners or Registered nurse practitioner or Registered nurse practitioners or Registered nurse-nurse practitioner or Registered nurse nurse practitioners or Women's health nurse practitioner or Women's health nurse practitioners or (WHNP and nurse) or (WHNP and nurses) or (WHNP and nursing) or Infirmiere praticienne specialisee or Infirmieres praticiennes specialisees or Family nurse practitioner or Family nurse practitioners or (FNP and Nurse) or (FNP and Nurses) or (FNP and Nursing) or Geriatric nurse practitioner or Geriatric nurse practitioners or Gerontological nurse practitioner or Gerontological nurse practitioners or (GNP and Nurse) or (GNP and Nurses) or (GNP and Nursing) or Adult gerontology nurse practitioner or Adult gerontology nurse practitioners or (AGNP and Nurse) or (AGNP and Nurses) or (AGNP and Nursing) or Adult gerontology primary care nurse practitioner or Adult gerontology primary care nurse practitioners or (AGPCNP and Nurse) or (AGPCNP and Nurses) or (AGPCNP and Nursing) or Primary care nurse practitioner or Primary care nurse practitioners or (PCNP and Nurse) or (PCNP and Nurses) or (PCNP and Nursing) or Primary health care nurse practitioner or Primary health care nurse practitioners or Primary healthcare nurse practitioner or Primary healthcare nurse practitioners or Primary health-care nurse practitioner or Primary health-care nurse practitioners or (PHCNP and Nurse) or (PHCNP and Nurses) or (PHCNP and Nursing) or Adult gerontology acute care nurse practitioner or Adult gerontology acute care nurse practitioners or (AGACNP and Nurse) or (AGACNP and Nurses) or (AGACNP and Nursing) or Advanced critical

care practitioner or Advanced critical care practitioners or (ACCP and Nurse) or (ACCP and Nurses) or (ACCP and Nursing) or Advanced neonatal nurse practitioner or Advanced neonatal nurse practitioners or (ANNP and Nurse) or (ANNP and Nurses) or (ANNP and Nursing) or Advanced paediatric nurse practitioner or Advanced paediatric nurse practitioners or (cAPNP and Nurse) or (cAPNP and Nurses) or (cAPNP and Nursing) or Advanced pediatric nurse practitioner or Advanced pediatric nurse practitioners or Enhanced neonatal nurse practitioner or Enhanced neonatal nurse practitioners or (ENNP and Nurse) or (ENNP and Nurses) or (ENNP and Nursing) or Oncology nurse practitioner or Oncology nurse practitioners or (ONP and Nurse) or (ONP and Nurses) or (ONP and Nursing) or Pediatric acute care nurse practitioner or Pediatric acute care nurse practitioners or Paediatric acute care nurse practitioner or Paediatric acute care nurse practitioners or (PNPAC and Nurse) or (PNPAC and Nurses) or (PNPAC and Nursing) or Acute care nurse practitioner or Acute care nurse practitioners or (ACNP and Nurse) or (ACNP and Nurses) or (ACNP and Nursing) or Adult nurse practitioner or Adult nurse practitioners or Critical care nurse practitioner or Critical care nurse practitioners or Emergency nurse practitioner or Emergency nurse practitioners or (ENP and Nurse) or (ENP and Nurses) or (ENP and Nursing) or (Hospital-based and nurse practitioner) or (Hospital-based and nurse practitioners) or (Hospitalised and nurse practitioner) or (Hospitalised and nurse practitioners) or (Hospitalized and nurse practitioner) or (Hospitalized and nurse practitioners) or (Intensive care unit and nurse practitioner) or (Intensive care unit and nurse practitioners) or (Intensive care units and nurse practitioner) or (Intensive care units and nurse practitioners) or (ICU and nurse practitioner) or (ICU and nurse practitioners) or (ICUs and nurse practitioner) or (ICUs and nurse practitioners) or Mental Health Nurse Practitioner or Mental Health Nurse Practitioners or (MHNP and Nurse) or (MHNP and Nurses) or (MHNP and Nursing) or Psychiatric-Mental Health Nurse Practitioner or Psychiatric-Mental Health Nurse Practitioners or Psychiatric Mental Health Nurse Practitioner or Psychiatric Mental Health Nurse Practitioners or PMHNP or PMHNPs or Pediatric nurse practitioner or Pediatric nurse practitioners or Paediatric nurse practitioner or Paediatric nurse practitioners or (PNP and Nurse) or (PNP and Nurses) or (PNP and Nursing) or Neonatal nurse practitioner or Neonatal nurse practitioners or (NNP and Nurse) or (NNP and Nurses) or (NNP and Nursing))) IN DARE

8. #4 OR #5 OR #6 OR #7

9. MeSH DESCRIPTOR Nurse Specialists EXPLODE ALL TREES IN DARE

10. MeSH DESCRIPTOR Infection Control Practitioners EXPLODE ALL TREES IN DARE

11. ((Clinical nurse specialist or Clinical nurse specialists or (CNS and nurse) or (CNS and nurses) or (CNS and nursing) or Clinical nurse consultant or Clinical nurse consultants or Clinical specialist or Clinical specialists or Infection control practitioner or Infection control practitioners or Nurse clinician or Nurse clinicians or Nurse consultant or Nurse consultants or Nurse specialist or Nurse specialists or Specialist nurse or Specialist nurses or Infirmiere clinicienne specialisee or Infirmieres cliniciennes specialisees or Verpleegkundig specialist or Verpleegkundig specialist geestelijk gezondheidszorg or Verpleegkundig specialist acute zorg bij somatische aandoeningen)) IN DARE

12. #9 OR #10 OR #11

13. (((Systematic* adj3 (Review* or Overview*)) or (Methodologic* adj3 (Review* or Overview*)))) IN DARE

14. ((systematic review or meta-analysis)) IN DARE

15. MeSH DESCRIPTOR Meta-Analysis EXPLODE ALL TREES IN DARE

16. MeSH DESCRIPTOR Meta-Analysis as Topic EXPLODE ALL TREES IN DARE

17. MeSH DESCRIPTOR Systematic Review EXPLODE ALL TREES IN DARE

18. MeSH DESCRIPTOR Systematic Reviews as Topic EXPLODE ALL TREES IN DARE

19. ((Meta Analy* or Metanaly*)) IN DARE

20. ((meta-analy* or metaanaly* or systematic review*)) IN DARE

21. ((cochrane or evidence report)) IN DARE

22. (((overview* or review or synthesis or summary or cochrane or analysis) and (reviews or meta-analyses or articles))):TI IN DARE

23. ((meta-review or metareview)) IN DARE

24. (((overview* or reviews) and (systematic or cochrane))):TI IN DARE

25. ((reviews adj2 (meta or published or quality or included or summar*))) IN DARE

26. (cochrane reviews) IN DARE

27. ((evidence and (reviews or meta-analyses))):TI IN DARE

28. #13 OR #14 OR #15 OR #16 OR #17 OR #18 OR #19 OR #20 OR #21 OR #22 OR #23 OR #24 OR #25 OR #26 OR #27

29. #3 OR #8 OR #12

30. #28 AND #29

31. (#30) IN DARE FROM 2011 TO 2023
