# Supplementary material for: A global perspective of advanced practice nursing research: A review of systematic reviews
Source: PLoS One. 2024 Jul 2;19(7):e0305008. doi: 10.1371/journal.pone.0305008 (PMC11218965; doi:10.1371/journal.pone.0305008)
Supplement: S3 Appendix — (PDF) [file pone.0305008.s004.pdf]

S3 Appendix. Search strategies for the grey literature.

Sample grey literature search terms:
(Advanced practice nurs* OR Nurse practitioner* OR Clinical nurse specialist*) AND (Primary care OR Acute care) AND Systematic review*

CADTH Information Services, Grey Matters: a practical tool for searching health-related grey literature
https://www.cadth.ca/resources/finding-evidence/grey-matters

Organization for Economic Co-operation and Development (OECD)
https://www.oecd.org/

ProQuest Dissertation and Theses
https://about.proquest.com/products-services/pqdtglobal.html

World Health Organization
https://www.who.int/

International Council of Nurses
https://www.icn.ch/

00 - GENERAL, MULTIDISCIPLINARY
East View Information Services
https://www.eastview.com

GreyNet International, Grey literature Network Service
http://www.greynet.org

Grijze Literatuur in Nederland, GLIN
http://www.publiekwijzer.nl/bestanden.php?id=zoeknaar&db=3.2

Italian Grey Literature Database
http://polarcnr.area.ge.cnr.it/cataloghi/bice/index.php?type=Grigia

National Repository of Grey Literature, NRGL
http://www.nusl.cz/?lang=en

OpenGrey Repository, System for Information on Grey Literature in Europe
http://www.opengrey.eu

06 - BIOLOGICAL & MEDICAL SCIENCES

Cochrane Reviews
https://www.cochranelibrary.com/

Doctor of Nursing Practice, DNP
https://libguides.rutgers.edu/c.php?g=1248282

Duke University Medical Center Library
http://guides.mclibrary.duke.edu/greyliterature

Gray Literature in Health Research
http://researchguides.dml.georgetown.edu/content.php?pid=352972&sid=2887419

Grey Horizon, A Grey Literature Current Awareness Tool in Cancer Care
http://grey-horizon.blogspot.nl/

Grey Literature for Dentistry
https://guides.library.utoronto.ca/dentistrygreylit-statistics

Grey Literature in the Health Sciences
https://guides.library.upenn.edu/healthgreylit

Grey Literature Report - New York Academy of Medicine
http://www.greylit.org

HealthKnowledge
http://www.healthknowledge.org.uk/public-health-textbook/research-methods/1a-epidemiology/grey-literature

Lister Hill Library of the Health Sciences
http://libguides.lhl.uab.edu/GreyLit

Mesothelioma Guide
https://www.mesotheliomaguide.com/mesothelioma/causes/asbestos-and-cancer/

Mesothelioma.net
https://www.mesothelioma.net

Norris Medical Library - Grey Literature Resource Guide
http://norris.usc.libguides.com/grey_literature

Nursing and Allied Health Resource Section, NAHRS
http://sites.google.com/site/nahrsnursingresources/Home/grey-literature-1

Social Policy and Practice
http://bathhealthnews.blogspot.com/2009/11/new-database-social-policy-practice.html

Health Sciences Library and Informatics Center
http://libguides.health.unm.edu/content.php?pid=200149

University of Waterloo
https://subjectguides.uwaterloo.ca/c.php?g=695484&p=4932747
