# Supplementary material for: A global perspective of advanced practice nursing research: A review of systematic reviews
Source: PLoS One. 2024 Jul 2;19(7):e0305008. doi: 10.1371/journal.pone.0305008 (PMC11218965; doi:10.1371/journal.pone.0305008)
Supplement: S1 Table — (PDF) [file pone.0305008.s005.pdf]

S1 Table. Risk of bias assessment for the included systematic reviews (n=117).

First author (year) [ref]	1. Did the review address a clearly focused question?	2. Did the authors look for the right type of papers?	3. Do you think all the important, relevant studies were included?	4. Did the review's authors do enough to assess quality of the included studies?	5. If the results of the review have been combined, was it reasonable to do so?	6. What are the overall results of the review?	7. How precise are the results?	8. Can the results be applied to the local population?	9. Were all important outcomes considered?	10. Are the benefits worth the harms and costs?
Abraham (2019) [131]	1	1	1	1	1	1	1	1	1	1
Allsop (2021) [10]	1	1	0	1	1	1	1	1	1	1
Alotaibi (2020) [75]	1	1	1	1	1	1	1	1	1	1
Andregard (2015) [137]	1	1	0	1	1	1	1	1	1	1
Ansell (2017) [100]	1	1	0	0	1	1	1	1	1	1
Arian (2017) [88]	1	1	0	1	1	1	1	1	1	1
Audet (2021) [7]	1	1	1	1	1	1	1	1	1	1
Baker (2017) [41]	1	1	0	1	1	1	1	1	1	1
Barker (2018) [89]	1	1	0	1	1	1	1	1	1	1
Barrott (2023) [124]	1	0	0	1	1	1	1	1	1	1
Belun-Vieira (2016) [76]	1	0	0	1	1	1	1	1	1	1
Bohner (2012) [98]	1	1	1	0	1	1	1	1	1	1
Borum (2018) [145]	1	1	0	0	0	1	0	1	1	1
Bryant-Lukosius (2015) [70]	1	1	1	1	1	1	1	1	1	1
Bryant-Lukosius (2015)* [83]	1	1	1	1	1	1	1	1	1	1

Carranza (2021) [61]	1	1	0	1	0	1	0	1	0	0
Chan (2018) [42]	1	1	0	1	1	1	1	1	1	1
Cheng (2018) [77]	1	1	0	1	1	1	1	1	1	1
Chua (2023) [116]	1	1	1	1	1	1	1	1	1	1
Clavo-Hall (2018) [138]	1	1	0	0	1	1	1	1	0	1
Cook (2017) [71]	1	1	0	1	1	1	1	1	1	1
Cooper (2019) [136]	1	1	0	1	1	1	1	1	1	1
Curr (2015) [134]	1	1	0	1	1	1	1	1	1	1
Dawson (2015) [101]	1	1	0	1	1	1	1	1	1	1
De Thurah (2017) [43]	1	1	1	1	1	1	1	1	1	1
Donald (2013) [44]	1	1	1	0	1	1	1	1	1	1
Donald (2015)* [33]	1	1	1	1	1	1	1	1	1	1
Driscoll (2015) [90]	1	1	1	1	1	1	1	1	1	1
Edkins (2014) [91]	0	1	0	0	1	1	1	1	1	1
Elder (2015) [86]	1	0	0	0	1	1	0	0	0	0
Emrich-Mills (2019) [114]	1	1	0	0	1	1	1	1	1	1
Fichadiya (2021) [57]	1	1	0	0	1	1	1	1	1	1
Fraser (2018) [132]	1	1	0	0	0	1	0	0	0	0
Fung (2014) [45]	1	1	0	1	1	1	1	1	1	1
Galiana-Camacho (2018) [87]	1	0	0	1	1	1	1	1	1	1

Garner (2017) [46]	1	1	0	1	1	1	1	1	1	1
Geese (2022) [128]	1	1	0	1	1	1	1	1	1	1
Gielen (2014) [48]	1	1	1	1	1	1	1	1	1	1
Hako (2023) [144]	1	1	0	1	1	1	1	1	1	1
Han (2018) [129]	1	1	0	0	0	1	0	0	0	0
Harkless (2018) [139]	1	1	0	0	0	1	1	1	1	1
Hourahane (2012) [140]	1	1	0	1	1	1	1	1	1	1
HQO (2013) [62]	1	1	0	1	1	1	1	1	1	1
Hutchinson (2014) [141]	1	1	1	1	1	1	1	1	1	1
Hyde (2020) [115]	1	1	0	1	1	1	1	1	1	1
Hyer (2019) [117]	1	1	1	1	1	1	0	0	0	0
Ismail (2013) [102]	0	0	1	0	0	0	0	0	0	0
Jennings (2015) [103]	1	1	1	1	1	1	1	1	1	1
Jeyaraman (2022) [104]	1	1	0	1	1	1	1	1	1	1
Johnson (2015) [105]	1	0	0	0	1	1	1	1	0	0
Jokiniemi (2012) [146]	1	1	1	0	1	1	1	1	1	1
Joseph (2015) [106]	1	1	0	0	1	1	0	1	1	1
Kennedy (2012) [92]	1	1	1	1	1	1	1	1	1	1
Kilpatrick (2014)* [55]	1	1	1	1	1	1	1	1	1	1
Kilpatrick (2015)* [56]	1	1	1	1	1	1	1	1	1	1
Kobleder (2017) [80]	1	1	0	1	1	1	1	1	1	1

Kuethe (2013) [85]	1	1	1	1	1	1	1	1	1	1
Kwok (2022) [99]	1	1	0	1	1	1	1	1	1	1
Lawton (2018) [84]	1	1	0	1	1	1	1	1	1	1
Leduc (2021) [93]	1	1	0	1	1	1	1	1	1	1
Lempp (2020) [82]	1	1	0	1	1	1	1	1	1	1
Loescher (2018) [72]	1	1	0	1	0	1	0	1	0	0
Lovink (2017) [34]	1	1	1	1	1	1	1	1	1	1
Lyness (2021) [130]	1	1	0	1	0	1	1	1	1	1
Manoj (2019) [63]	1	1	0	0	1	1	1	1	1	1
Martinez-Gonzalez (2014) [65]	1	1	0	1	1	1	1	1	1	1
Martin-Misener (2015)* [69]	1	1	1	1	1	1	1	1	1	1
Massimi (2017) [64]	1	1	0	1	1	1	1	1	1	1
McCrory (2018) [58]	1	1	0	1	1	1	1	1	1	1
McMenamin (2023) [47]	1	1	0	1	1	1	1	1	1	1
McParland (2022) [35]	1	1	0	1	1	1	1	1	1	1
McQuilkin (2020) [59]	1	1	0	0	0	1	0	1	0	1
Medeiros (2011) [94]	1	1	0	0	0	1	1	1	1	1
Mileski (2020) [49]	1	1	0	0	1	1	0	0	0	0
Monterosso (2019) [60]	1	1	0	1	1	1	1	1	1	1

Morilla-Herrera (2016) [36]	1	1	0	1	1	1	1	1	1	1
Ness (2016) [125]	1	0	0	1	1	1	1	1	1	1
Newhouse (2011)/ Stanik-Hutt (2013) [39-40]	1	1	0	1	1	1	1	1	1	1
Niezen (2014) [107]	1	1	0	1	1	1	1	1	1	1
Nikpour (2022) [112]	1	1	1	1	1	1	1	1	1	1
Norful (2019) [66]	1	1	0	1	1	1	1	1	1	1
Ordonez-Piedra (2021) [95]	1	1	0	1	0	1	1	1	1	1
Osakwe (2020) [37]	1	0	0	1	1	1	1	1	1	1
Patel (2019) [108]	1	1	0	1	1	1	1	1	1	1
Ramis (2013) [145]	1	1	0	1	1	1	0	1	1	1
Raymond (2022) [148]	1	1	1	0	1	1	1	1	1	1
Rutherford-Hemming (2016) [118]	1	1	0	1	1	1	1	1	1	1
Salamanca-Balen (2018) [126]	1	1	0	1	1	1	1	1	1	1
Schadewaldt (2011) [67]	1	1	0	1	1	1	1	1	1	1
Schallmo (2019) [119]	1	1	0	1	0	1	0	1	0	0
Scheydt (2021) [79]	1	1	0	0	1	1	1	1	1	1
Schneider (2021) [80]	1	1	1	1	1	1	1	1	1	1

Schoch (2014) [135]	1	1	0	1	1	1	1	1	1	1
Scott (2011) [68]	1	1	1	1	1	1	1	1	1	1
Searle (2023) [133]	1	0	1	1	1	1	1	1	1	1
Smigorowsky (2020) [50]	1	1	0	1	1	1	1	1	1	1
Smith (2014) [73]	1	0	0	0	1	1	1	1	1	1
Speight (2019) [120]	1	1	0	1	1	1	1	1	1	1
Stratton (2020) [121]	1	1	0	0	0	1	0	0	0	0
Sun (2022) [38]	1	1	0	1	1	1	1	1	1	1
Swan (2015) [51]	1	0	0	1	1	1	1	1	1	1
Thamm (2019) [109]	1	1	0	1	1	1	1	1	1	1
Thomas (2019) [110]	1	1	1	1	1	1	1	1	1	1
Tsiachristas (2015) [52]	1	1	0	1	1	1	1	1	1	1
Turi (2023) [81]	1	1	0	1	1	1	1	1	1	1
Van Camp (2013) [97]	1	1	1	0	1	1	1	1	1	1
van Vliet (2020) [53]	1	1	1	1	1	1	1	1	1	1
Veenema (2021) [122]	1	1	0	1	1	1	1	1	1	1
Wang (2019) [74]	1	1	1	1	1	1	1	1	1	1
Wang-Romjue (2018) [142]	1	1	0	1	1	1	1	1	1	1
Warren (2016) [123]	1	1	0	1	1	1	1	1	1	1
Whiteford (2016) [111]	1	1	0	1	1	1	1	1	1	1

Woo (2017) [96]	1	1	0	1	1	1	1	1	1	1
Wu (2020) [113]	1	1	0	1	1	1	1	1	1	1
Xue (2016) [143]	1	1	0	1	1	1	1	1	1	1
Yang (2021) [54]	1	1	0	1	1	1	1	1	1	1
Zhang (2022) [127]	1	1	0	1	1	1	1	1	1	1

* Systematic review published as constellation papers, with additional methodological data extracted from: 154. Marshall D, Donald F, Lacny S, Reid K, Bryant-Lukosius D, Carter N, et al. Assessing the quality of economic evaluations of clinical nurse specialists and nurse practitioners: A systematic review of cost-effectiveness. NursingPlus Open. 2015;1(2015):11-7.doi:10.1016/j.npls.2015.07.001; and 155. Donald F, Kilpatrick K, Reid K, Carter N, Martin-Misener R, Bryant-Lukosius D, et al. A systematic review of the cost-effectiveness of nurse practitioners and clinical nurse specialists: What is the quality of the evidence? Nurs Res Pract. 2014;2014. doi:10.1155/2014/896587.
