# Supplementary material for: A global perspective of advanced practice nursing research: A review of systematic reviews
Source: PLoS One. 2024 Jul 2;19(7):e0305008. doi: 10.1371/journal.pone.0305008 (PMC11218965; doi:10.1371/journal.pone.0305008)
Supplement: S2 Table — (PDF) [file pone.0305008.s006.pdf]

S2 Table. Description of included systematic reviews (n=117).

Author (year)	Systematic Review Objectives	Databases (N)	Countries	Included Studies (N) (date range)	APN Role and Population	Intervention/Comparator	Data Synthesis	Funded Study	APNs involved in research team
Abraham (2019) [131]	To determine the cost-effectiveness of chronic disease care management delivered by APNs compared to physicians.	N=9	N = 5 USA, Netherlands, UK, England, Whales	N=11 (2000-2016)	**APN role:** APNs are registered nurses with advanced training at the master's or doctoral level and can diagnose and manage patient conditions by performing assessments, ordering and interpreting diagnostic tests, prescribing medications and treatments, and providing patient education **Population:** Patients in primary care settings with chronic disease followed by APNs	**Intervention:** APN providing care or prescribing blood tests, echocardiograms, remote cardiac monitoring (e.g., Holter monitoring), chest x-rays, consultations, electrocardiography, emergency care **Comparato**r: Physician care	NR	Yes	NR
Allsop (2021) [10]	To explore the role and impact of APNs in care of patients following fragility hip fracture	N=5	N=10 Australia, Canada, Hong Kong, Ireland, Netherlands, Singapore, Spain, Taiwan, UK, USA	N=19 (After 2003)	**APN role:** Six characteristics of APNs were identified: (1) coordination; (2) collaboration; (3) education; (4) assessment, investigation and treatment recommendations; (5) discharge planning, support and follow up; and (6) documentation. **Population:** Patients post fragility hip fracture (n=43,218).	**Intervention:** Service delivery models focussed on managing orthopedic clinical pathway: evaluating patients' needs, designing individualized education and care plans, supporting multi-disciplinary team, interpreting test results, addressing barriers to discharge, developing professional rapport with patients and families, initiating referral services, completing discharge summaries. **Comparator:** Usual care	Narrative synthesis	No	NR
Alotaibi (2020) [75]	To identify how Advanced NPs can contribute to oncology care.	N=4	NR	N=5 (2009-2015)	**APN role:** NP in oncology **Population:** Elder patients in oncology undergoing treatment, patients at risk of cancer return	**Intervention:** NPs' care includes clinic visits, phone calls, mental health interventions, palliative care, quality of life assessments, hospice education, and symptom management. They monitor to treat side effects from treatments. They work with nurses to provide	Narrative synthesis	Yes	NR

						care to those patients who are receiving treatment.<br>**Comparator:** Usual care			
Andregard (2015) [137]	To explore obstacles and opportunities to achieve optimal interprofessional team collaboration with the NP's introduction.	N=2	N=7<br>Not all listed, Canada, Taiwan, UK, USA	N=26 (1995-2012)	**APN role:** NP<br>**Population:** Inpatient and Outpatient settings	**Intervention:** Introduction of NP into team<br>**Comparator:** N/A	Meta-synthesis	No	Yes
Ansell (2017) [100]	To identify interventions designed to reduce wait times for primary care appointments.	N=6	N=3<br>Canada, USA, UK	N=11 (2004-2010)	**APN role:** Primary healthcare NP<br>**Population:** Patients in primary care, all health conditions	**Intervention:** Activities aimed at reducing wait times for primary care appointments<br>**Comparator:** No intervention	Descriptive approach	No	No
Arian (2017) [88]	To describe what nurses prescribe, and the challenges and consequences of nurse prescribing	N=10	N=4<br>Australia, England, Sweden, UK	N=72 (1999-2017)	**APN role:** Independent and supplementary nurse prescribers with at least three years of clinical experience in addition to education and participation in training courses.<br>**Population:** Patients with diabetes, mental health issues, rheumatological and dermatological conditions primary care, elder care and geriatrics, epilepsy, pain, asthma, hypertension, constipation, Emergency, hemodialysis, glaucoma.	**Intervention**: Implementation of independent nurse prescribers.<br>**Comparator**: Physicians	Narrative synthesis	NR	NR
Audet (2021) [7]	1) Identify patient and organizational outcomes of advanced practice nursing roles in post-operative cardiac surgery<br>2) Synthesize the evidence of current roles of	N=6	N=3<br>USA, Canada, Korea	N=13 (1999-2019)	**APN role:** NPs and CNSs<br>**Population:** Patients with coronary artery bypass graft with/ without valve repairs, percutaneous coronary intervention, myocardial infarction, and several cardiac interventions	**Intervention:** NP care included health assessment, symptom management, management of pharmacotherapy and diagnostic tests, lifestyle modification, counselling, telephone and follow-up appointments after discharge. CNS care included: evaluation of the patient's smoking status, level of motivation to quit, patient morale, self-efficacy, coping	Narrative synthesis	Yes	No

	APNs in post-operative cardiac surgery to provide the best quality of care for patients.					strategies, support, symptom management, and healthy lifestyle. In acute care, CNSs assessed patient hemodynamic stability, clinical symptoms, chest- tube drainage, surgical wound healing, laboratory tests, based on a standardized protocol.<br>**Comparator:** Usual care, physician			
Baker (2017) [41]	To examine the clinical and cost effectiveness of nurse-led self-management interventions for patients with chronic obstructive pulmonary disease in primary care.	N=7	$N_{(Total)}$=14<br><br>N=3 in studies with Advanced NPs: USA, UK, Taiwan	$N_{(Total)}$= 20<br><br>N=4 studies with Advanced NPs (2012-2014)	**APN role:** Advanced NPs<br>**Population:** At least 80% of participants were community dwelling adults with chronic obstructive pulmonary disease	**Intervention**: Nurse-led self-management approach including at least two of the following components: smoking cessation, self-recognition and self-treatment of exacerbations, exercise or physical activity component, advice about diet, advice about medication or coping with breathlessness.<br>**Comparator:** usual care	Narrative	No	NR
Barker (2018) [89]	Investigate how health outcomes of older adults in long-term care vary according to which professional group(s) provides first-line medical care.	N=4	N=9 Australia, Austria, Canada, Germany, Netherlands, New Zealand, Spain, UK, USA	N=24 (After 2000)	**APN role**: Primary healthcare NP<br>**Population:** Residents in long-term care	**Intervention**: Assessment of residents, prescription and monitoring of medication, pro-active outreach, education, clinical coaching, care coordination for high-risk patients (including comprehensive geriatric assessment, liaison with specialists), minor procedures, follow-up with families<br>**Comparator:** Usual care, care provided by family physician	Narrative synthesis	No	No
Barrott (2023) [124]	To review how nurse and pharmacist roles have been incorporated into the management of patients undergoing	N=7	N=10 USA, UK (includes England, Scotland), Netherlands, Czech	$N_{(Total)}$= 15 (2010-20121)<br>N=4 focussed on nurse	**APN role:** Multi-professional, (some nurses), advanced clinical practice- involves experienced professionals from various healthcare backgrounds, including nursing and pharmacy, working autonomously and utilizing advanced knowledge, decision making,	**Intervention:** Skills include physical assessment and prescribing; four levels of systemic anti-cancer therapy nurse practice are identified, ranging from highly protocolled, checklist-led pre-treatment toxicity reviews to complete nurse led clinical	Thematic analysis	Yes	NR

	systemic anti-cancer therapy services and their impact on patient experience and care provision		Republic, Germany, Italy, Norway, Poland, Turkey	prescribing	leadership skills and research to improve patient care<br>**Population:** Patients undergoing systemic anti-cancer therapy	management for a systemic anti-cancer therapy regimen. Advanced practitioners support treatment side effect management.<br>**Comparator:** Medical prescribers			
Belun-Vieira (2016) [76]	To compare medical with CNS-led follow-up by assessing the patient experience, quality of life and emotional wellbeing.	N=3	N=4<br>UK, Netherlands, Sweden, Australia	N=10 (1996-2011)	**APN role:** CNS, Cancer Nurse Specialist<br>**Population**: Patients with cancer, irrespective of the type of cancer	**Intervention:** Nurse-led follow-up, with a focus of consultation from physical examination to a more holistic patient-centred model of care resulting in high patient satisfaction. Nurse-led services could be face to face or by phone.<br>**Comparator:** Medical follow-up, usual care	Meta-analysis	NR	Yes
Bohner (2012) [98]	To examine what impact do APNs have on outcome of patients with heart failure	N=4	N=1<br>USA	N=5	**APN role:** APNs include nursing roles with master's level qualifications such as CNS or NP. APNs exhibit specialized knowledge and expertise, clinical judgment, highly skilled, self-initiated care, and research.<br>**Population:** Patients with heart failure	**Intervention:** APNs provide comprehensive transitional care, additional emails about medication management by CNS, telehealth and telephone interventions<br>**Comparator:** Home visits by nurses, usual discharge, information email, no comparator	Narrative synthesis	NR	NR
Borum (2018) [147]	To identify what are the barriers for NPs utilizing clinical decision support in hospital settings?	N=9	NR	N=9 (2011-2017)	**APN role:** NPs, mid-levels, APNs, advanced practice registered nurses, or advanced practice professionals working in hospitals and acute care settings<br>**Population:** Hospital based NPs	**Intervention:** The utilization of clinical decision support by APNs in hospitals and acute care settings. Clinical decision support is "a process for enhancing health-related decisions and actions with pertinent, organized clinical knowledge and patient information to improve health and healthcare delivery using technology.<br>**Comparator:** None	Narrative synthesis	No	Yes
Bryant-Lukosius (2015) [70]	To make evidence-based recommendations about APN roles for optimizing patient, provider, and health system	N=4	N=4<br>Canada, USA, Sweden, UK	N=29 published in 31 papers (1988-2012)	**APN role:** CNSs and NPs across the cancer care continuum. Adopted the definition of the Canadian Nurses Association<br>**Population**: Patients receiving cancer care (prevention, diagnosis, treatment,	**Intervention**: Cancer care provided by APNs<br>**Comparator:** Usual care	Narrative synthesis	Yes	Yes

	outcomes across the cancer journey				survivorship/post-treatment follow-up, palliative, and end-of-life care				
Bryant-Lukosius (2015)* [83]	To summarize systematic review results specific to the clinical effectiveness and cost-effectiveness of master's prepared CNSs providing transitional care	N=10	N=2 USA, UK	N=13 (1986-2011)	**APN role:** CNSs delivering transitional care where a central feature was continuity of care from the hospital to the home.<br>**Population:** The studies focused on four populations at risk for poor health including patients with post-cancer surgery, patients with heart failure, elderly patients, and high-risk pregnant women and low birthweight infants	**Interventions:** included discharge planning, care coordination and patient education, medication review, referrals to other providers and services, counselling, emotional support, teaching self-care skills, caregiver support, patient safety, wound care, symptom management, disease monitoring and treatment adherence<br>**Comparator:** to usual care alone	Narrative synthesis	Yes	Yes
Carranza (2021) [61]	Determine how care provided by NPs compares with that of physicians in patient satisfaction and clinical outcomes, for patients seeking specialized care in ambulatory settings.	N=3	N=4 USA, UK, South Africa, Netherlands	N=11 (1995-2016)	**APN role**: Primary healthcare NP<br>**Population:** Patients from different specialties (chronic lung disease, hypertension, diabetes, rheumatology, cardiology, HIV, gastroenterology, pediatric cardiology, psychology and dermatology)<br>NP: n=1, others not reported<br>Physicians: n =239<br>Patients: n =4529	**Intervention:** Care offered by NPs to patients or for specific disease processes (i.e., HIV, cardiovascular, or diabetes management within primary care), and conducted in an outpatient setting<br>**Control**: Usual care, care provided by physicians	Narrative synthesis	No	Yes
Chan (2018) [42]	To assess the clinical effectiveness of nurse-led services in the ambulatory or community care setting,	N=4	N=9 Sweden, UK, Taiwan, Denmark, Netherlands, Hong Kong, USA, Australia, Spain	N=25 (2005-2016)	**APN role:** NP-type services "nurse-led services" as a model of care where the registered nurse has primary responsibility for patient management and care for a cohort of patients located in an ambulatory or community care setting<br>**Population:** People who received nurse-led services in an ambulatory or community care setting	**Intervention:** Nurse-led services as a model of care where the registered nurse has primary responsibility for patient management and care for a cohort of patients located in an ambulatory or community care setting.<br>**Comparator:** Usual care or any other alternative model of care (e.g. physician-led services)	Qualitative synthesis	Yes	NR

Cheng (2018) [77]	To compare quality of life between nurse-led and non-nurse-led interventions for patients with cancer	N=3	N=6 Korea, England, Sweden, China, Australia, Netherlands	N=7 (2009-2017)	**APN role:** Nurse-led disease management program for patients with cancer provides symptom management, psychological and/or social support, lifestyle changes, health education, support, tailored-up, and coaching **Population:** Patients with cancer	**Intervention:** Education, behavioural training, stress management, cognitive therapy, emotional support, telephone supportive care, Nurse-led (in-hospital care, follow-up support, and relatives and caregivers received training), Survivor care (telephone follow-up, face-to-face end-of-treatment session. Nurse-led follow-up and home visits **Comparator:** Routine discharge, routine care, hospital follow-up, standard follow-up	Meta-analysis	No	NR
Chua (2023) [116]	To consolidate and synthesize evidence of using standardized patients on APN students' learning and assessment experiences.	N=7	N=3 Singapore, Taiwan, USA	N=20 (2002-2022)	**APN role:** NP students **Population:** NP students using standardized patients for education	**Intervention**: Use of standardized patients (individual, paired, group) **Comparator**: Traditional education experiences without standardized patients	Narrative synthesis	No	NR
Clavo-Hall (2018) [138]	To better understand what roles and activities certified Clinical Nurse Leaders are enacting when not hired into formally designated Clinical Nurse Leader roles	N=2	NR	N=69	**APN role:** Clinical Nurse Leader assumes accountability for healthcare outcomes for a specific group of clients within a unit or setting through the assimilation and application of research-based information to design, implement, and evaluate client plans of care. The Clinical Nurse Leader is trained to identify the clinical and cost outcomes that will improve safety, timeliness, efficiency, effectiveness, quality and patient-centeredness. Roles included: faculty, clinical management/executive, specialty clinician, and staff nurse	**Intervention:** Certified Clinical Nurse Leaders are involved in developing and implementing academic curriculum, including Clinical Nurse Leader immersion experiences and accelerated nursing programs. Those not hired as Clinical Nurse Leaders were involved in research related to Clinical Nurse Leader practice, dyspnea management for hospice patients, chronic obstructive pulmonary disease and ventilator associated pneumonia, and the impact of depression on quality **Comparator:** None identified	NR	Not indicated	NR

Cook (2017) [71]	To explore patient experience of care by a specialist nurse in gynecological oncology.	N=6	N=4 Denmark, UK, Australia, Switzerland	N=7 (2009-2016)	**APN role:** CNS-type role in gynecological oncology included: CNSs, cancer nurse consultants, cancer care coordinators, specialist cancer nurses, liaison nurses, nurse navigators or APNs.<br>**Population:** Patients who received care from specialist nurses in gynecological oncology in acute hospitals, outpatient/ ambulatory clinics, chemotherapy or radiotherapy units, support groups, palliative care units or the patient's home.	**Intervention:** Tailored, accessible and expert care to women with gynecological cancer. Specialist nurses provide information to patients on their disease and treatment in the form preferred by the patient and ensure that information is understood.<br>**Comparator**: Not identified	Meta-synthesis of themes	NR	Not clearly stated
Cooper (2019) [136]	To systematically analyse the research literature on Advanced NPs and CNSs to compare the similarities and differences between the two roles	N=3	N=7 Australia, Canada, Europe, UK, Finland, Ireland, USA	N=12 (2006-2015)	**APN role:** NP roles defined in generalist areas such as general practice, Emergency rooms, or broad specialist areas such as sexual health, oncology or cardiology. The goals of the Advanced NP were broader and medically based. CNS defined by a specific disease, medical sub-specialty or type of care areas such as diabetes, stroke and heart failure<br>**Population**: All	**Intervention**: None-descriptive comparison of NP and CNS roles	Narrative	NR	Yes
Curr (2015) [134]	To examine the effectiveness of the Ottawa ankle rule, compared with standard care, in reducing length of stay within Emergency Care settings	N=5	NR	$N_{(Total)}$=4 (2000-2014)<br><br>N=1 NP study (2000)	**APN role:** NP<br>**Population:** Adults in Emergency room with ankle injury	**Intervention:** Use of the Ottawa ankle rule in triage<br>**Comparator:** using Emergency room protocol prior to implementation of Ottawa ankle rule, a registered nurse, not trained in Ottawa ankle rule	Narrative synthesis	Yes	NR
Dawson (2015) [101]	To identify nursing and midwifery workforce interventions that have led to an increase in the number of well-	N=8	N=2 NP studies: Canada, US	N=36<br>N=3 studies specific to NPs (2008-2014)	**APN role:** NP in primary care<br>**Population:** Women in rural areas, Medicare and Medicaid beneficiaries, rural workers 18–70 years of age who were injured in Washington state	**Intervention:** Introduction of NP roles in rural settings and expansion of scope of practice regulations<br>**Comparator**: rates of hospitalization and full practice authority, pre-implementation rates of claim filings	Content analysis	Yes	NR

	trained nurses and midwives								
De Thurah (2017) [43]	To compare the efficacy of embedded nurse-led versus conventional physician-led follow-up on disease activity in patients with rheumatoid arthritis	N=7	NR	N=7 (2003-2014)	**APN role**: Rheumatology nurses, CNSs or experienced rheumatology nurses trained in joint assessment. **Population**: Patients with rheumatoid arthritis, ankylosing spondylitis, psoriatic arthritis, juvenile idiopathic arthritis or undifferentiated polyarthritis.	**Intervention:** Nurse-led follow-up on disease control in patients with rheumatoid arthritis. Nurses performed assessment of tender and swollen joints, evaluated blood samples and monitored the medical treatment to assess the patients' disease activity. **Comparator:** Physician-led follow-up care	Meta-analysis	NR	NR
Donald (2013) [44]	Examine the effectiveness of APNs in meeting the healthcare needs of older adults living in long-term care.	N=12	N=1 USA	N=4 reported in 15 papers (1966-2010)	**APN roles:** NPs and CNSs in LTC **Population:** Adults aged 60 years and older living in LTC residential settings, their families or LTC staff	**Intervention:** NP/MD team provide care to nursing home residents; CNSs provide a 6-month educational intervention, facilitated application of evidence-based protocols, staff consultation, and direct care to residents for 6 months postadmission **Comparator**: physicians, usual care	Narrative synthesis	Yes	Yes
Donald (2015)* [33]	Determine the cost-effectiveness of NPs delivering transitional care in alternative or complementary roles	N=10	N=3 UK, Canada, USA	N=5	**APN role:** Primary healthcare NP **Population:** N= 1171 adults including n = 154 acute asthma patients (>16 years) discharged from hospital. NPs: n=7	**Intervention**: NP-delivered transitional interventions (follow-up care for asthma) **Comparator:** Usual care	Meta-analysis	Yes	Yes
Driscoll (2015) [90]	To assess the effects of nurse-led titration of medications in patients with heart failure with reduced ejection fraction in terms of safety and patient outcomes.	N=3	NR	$N_{(Total)}$=7 (2003-2015) **N=1 NP study** (Ansari 2003)	**APN role:** Primary healthcare NP **Population in NP study**: Patients diagnosed with heart failure that met the Framingham criteria and a left ventricular ejection fraction ≤45% or moderate or severe left ventricular systolic dysfunction on their last visit. Other Professionals: n =74; Patients : n =105	**Intervention:** Nurse-led titration of beta-blockers by study NP under cardiologist supervision **Comparator:** Health professionals were provided education about titration of beta-blockers	Meta-analysis	Yes	Yes

Edkins (2014) [91]	To examine provider models in intensive care units that include physicians, NPs, physician assistants	N=4	N=1 USA only	N=18<br><br>(2002 to 2012)	**APN role:** Advanced practice providers including NPs and physician assistants in critical care who function with and without residents, reporting to either an intensivist or an attending physician.<br>**Population**: Patients in neonatal, pediatric and adult intensive care units	**Interventions:** Insertion of intra-cranial monitors, thoracotomy tube insertion, ventilator management, identification of deep vein thromboses, patient follow-up post-trauma.<br>**Comparator:** Physicians and physician resident/fellow	NR	Yes	Yes
Elder (2015) [86]	To explore three key strategies designed to promote patient throughput in the Emergency room (increased nursing scope of practice, physician-assisted triage and medical assessment units.	N=5	N=7 Australia, UK, Canada, USA, Ireland, Singapore, Sweden.	$N_{(Total)}$= 21 (1980-2014)<br><br>N=11 studies for NPs	**APN role:** Primary healthcare NP<br>N total= 26,774<br>**Population:** Patients presenting to the Emergency room	**Intervention**: Clinical initiatives nurses in the Emergency room utilizing advanced practice nursing including nurse-initiated activities, such as analgesia and X-rays. An Emergency room NP is an independent practitioner who is able to assess, diagnose, treat, prescribe and refer to other health specialties.	Narra tive	No	Yes
Emrich-Mills (2019) [114]	To inform the development of Continued Professional Development workshops for mental health nurse prescribers working in memory services.	N=7	N=1 UK	N=9 (2005-2015)	**APN role:** Specialist practice for mental health nurse prescribers.<br>**Population:** Patient followed in memory services, dedicated to the diagnosis and treatment of Alzheimer's disease and other types of dementia.	**Intervention:** Medication management, de-prescription and drug interactions, psychotropic medications, assessment and diagnosis, referral for non-pharmacological interventions, pre-/post-diagnostic counselling and support, signposting to/from other healthcare professionals and supervising junior colleagues<br>**Comparator:** Physician, usual care	Narra tive revie w	Yes	NR
Fichadiya (2021) [57]	To review the existing evidence related to the role and effectiveness of NP-led heart failure management on outcomes of care for adult outpatients.	N=2	N=2 USA, Australia	N=5 (2003-2018)	**APN role:** NPs were responsible for the assessment and monitoring of heart failure patients' symptoms and their medication management either through NP-led telehealth or NP-led heart failure outpatient clinic<br>**Population:** Adults with heart failure enrolled in NP-led heart failure home management program or NP-led heart	**Intervention:** NP-led outpatient heart failure management either through telehealth or outpatient clinic visit<br>**Comparator**: Nurse home health, physician, usual care	Narra tive synth esis	NR	Yes

					failure outpatient clinic or physician outpatient usual care				
Fraser (2018) [132]	Examine methodologies employed in cost analyses of increasing scope of practice for APNs	N=3	N=1 USA	N=6 (1985-2015)	**APN role:** Primary healthcare NP **Population:** Patients and other stakeholders in primary care	**Intervention** : Economic analysis of expanded scope of practice regulations **Comparator:** benefit cost analyses, cost minimization analyses; economic impact analyses	Economic impact analysis	No	No
Fung (2014) [45]	Review reported aspects of the role and performance of psychiatric APNs.	N=11	N=3 USA, Australia, Ireland	N=14 (1997-2012) English only	**APN role:** Primary healthcare NP **Population:** Community and homebound individuals with serious mental Illness (schizophrenia); after surgery for ovarian cancer, depressed low-income mothers, and adolescents exposed to catastrophic stress Other Professionals: n =206 Patients: n=1893	**Intervention:** Providing psychiatric nursing consultation service, psycho-social interventions, transitional care prior to discharge **Comparator:** Usual care, skilled nursing, social work, physical and occupational therapy	Narrative synthesis	No	Yes
Galiana-Camacho (2018) [87]	To show evidence on the results of APN models in the Emergency room including professional competencies, cost-effectiveness results, patient safety and patient satisfaction for future implementation in Spain.	N=8	N=3 UK, Australia, USA	$N_{(Total)}$= 14 (2006-2017)	**APN role:** Primary healthcare NP **Population:** Patients qualified as non-urgent, using the Australasian Triage Scale with scores of 4-5 presenting to the Emergency room or primary care. Most common diagnoses: soft tissue (trauma, loss of skin integrity) (35%) and bone fractures (11%) Patients: n =6839	**Intervention:** Advanced practice nursing in emergency services, including different models of nurse prescribing and levels of autonomy, fast-track APN care, clinical initiative APN care, intervention and treatment by APNs **Comparator:** Care provided by the physician, other APNs. No comparison group in some studies	Narrative synthesis	No	NR
Garner (2017) [46]	To assess the effect of nurse-led care for patients with rheumatoid arthritis on care quality	N=3	N=4 Denmark, UK, Sweden, Netherlands	N=17 (1994-2015)	**APN role:** Primary healthcare NP **Population:** Patients with rheumatoid arthritis Patients: n =1569	**Intervention:** APN-led care in rheumatoid arthritis **Comparator:** Rheumatologist, junior doctor undergoing training in rheumatology, general practitioner, staff nurse working with rheumatologist, patients previous experience with other care model,	Narrative synthesis	No	Yes

						inpatient care and day-patient team care			
Geese (2022) [128]	To identify, evaluate, and summarize evidence about APNs' job satisfaction in cancer care	N=3	N=3 USA, Canada, Japan	N=4 (2007-2020)	**APN role:** APNs', NPs', CNSs' and physician assistants' job satisfaction in cancer care, in different settings including primary care, secondary care, inpatient care, and outpatient care **Population**: Patients with any type of cancer diagnosis.	Survey methodology **No intervention** **No comparator**	Narrative synthesis	None stated	Yes
Gielen (2014) [48]	To identify, appraise and synthesize the evidence presented in the literature on the effectiveness of nurse prescribing compared to physician prescribing.	N=11	N=6 USA, UK, Netherlands, Canada, Norway, Colombia	N=35 (1974-2011)	**APN role:** Nurse prescribing models involved independent nurse prescribing, supplementary nurse prescribing, a mix of independent and supplementary prescribing, and prescribing based on group directions in primary and secondary care **Population:** Mixed patient populations, and specified patient groups only (e.g. patients with diabetes, mental health patients or patients with acute minor illnesses), cardiology patients with hypertension, mental health care, sore throats or upper respiratory throat infections, birth control pills children with moderate asthma, management of constipation, and acute minor illnesses, and radiotherapy and oncology.	**Intervention**: Nurse prescribing of medicines as defined in one of the three general models of nurse prescribing, i.e. independent prescribing, supplementary prescribing or prescribing by patient group directions. **Comparison:** Physician prescribing	Narrative and tabular methods	Yes	NR
Hako (2023) [144]	To summarize and compare published international literature on APN capabilities.	N=3	N=6 Canada, USA, Australia, New Zealand, Ireland, UK	N=11 (2006-2020)	**APN role:** Seven APN titles were used: CNS and NP, advanced practice registered nurse, APN, advanced NP, advanced practitioner, and clinical specialist. **Population:** No specific population targeted	**Interventions:** None specified. Article addressed the profession, daily work, and dimensions of the capability of APNs. **Comparator:** None specified	Narrative synthesis	No	Yes
Han (2018) [129]	Explore relationships between advanced practice registered nurses' job satisfaction	N=3	N=1 USA	N=10 (1991-2017)	**APN role:** Advanced Practice Registered Nurses in single practice, single and multiple US states, a US administrative agency (the Veterans Health Administration). N=3,594 APRNs	**No intervention** **No comparison**<br><br>Examination of factors associated with job satisfaction and intent to leave	Narrative synthesis	Not indicated	Yes

	and intent to leave				**Population:** Advanced Practice Registered Nurses				
Harkless (2018) [139]	To investigate and synthesise literature around NP reimbursement policy from a state, payer and NP perspectives	N=8	N=1 USA only	N=17 (2006-2017)	**APN role:** NPs identified as primary care providers: credentialing and contracting; reimbursement parity; and "incident to" billing **Population:** Patients in primary care, long-term care, Medicare claim data	**Intervention:** Determine the influence of state-level Medicaid reimbursement and scope of practice legislation on NP clinical practice. **Comparator:** not specified, several cross-sectional surveys	NR	NR	Yes
Health Quality Ontario (2013) [62]	Determine effectiveness of specialized nurses with a clinical role in patient care to optimize chronic disease management for adults in the primary care.	N=6	N=3 US, UK, Netherlands	N=6 (Published before May 3rd 2012)	**APN role:** Primary healthcare NP **Population:** Patients with a chronic disease including diabetes, hypertension, coronary artery disease, congestive heart failure Physicians: n = 48 Patients: n =5088	**Intervention:** Model 1 (nurse alone) and Model 2 (nurse and physician versus physician alone). **Comparator:** Comparable outcomes between Model 1 (physician alone), or improved outcomes or efficiency in Model 2.	Narrative synthesis	No	No
Hourahane (2012) [140]	To synthesize the evidence on the experiences of UK consultant nurses in implementing a new role to identify inhibitors and facilitators of role development.	N=14	N=1 UK	N=11 (2002-2008)	**APN role:** Consultant nurses **Population:** Population included a mix of nurse consultants, a nurse midwife consultant, stakeholders, directors of nursing, and one health visitor.	**Intervention:** Consultant nurse role implementation **Comparator:** None	Meta-synthesis	NR	Yes
Hutchinson (2014) [141]	To derive an integrative description of the defining characteristics of advanced practice nursing	N=8	N=3 Australia, USA, UK	N=50 (1986-2012)	**APN role:** All advanced practice nurses including APNs, advanced NP, consultant nurse, higher level or advanced practitioner, CNS. **Population:** None specified	**Intervention**: APNs and domains of care unique to advanced practice. **Comparator**: None specified	Qualitative meta-summary	NR	NR

Hyde (2020) [115]	To critically appraise and synthesise the current evidence related to APN in children and young people's healthcare setting.	N=6	N=2 USA, Netherlands	N=9 (1998-2018)	**APN role:** APN in pediatrics in acute and primary care settings **Population:** Children and young people followed in cardiology, inpatient, trauma care, primary care, pediatric intensive care unit, eczema management, respiratory infections, surgical settings	**Intervention:** APN-managed inpatients with asthma, in the cardiology clinic, APN trauma care, primary care, pediatric intensive care unit 24/7, APN model in pediatric surgical setting, children with eczema and children with respiratory tract infections **Comparator**: Physician care	Narrative synthesis	Not mentioned	Yes
Hyer (2019) [117]	Examine the practice patterns of NPs related to weight management in primary care	N=6	N=4 North America, UK, Netherlands	N=13 (2010-2017)	**APN role:** Primary healthcare NP **Population:** Patient in weight management (obesity/overweight) practice patterns of NPs with primary care adult patients. Excluded: children, adolescents, or pregnancy-related care. Patients : n =3047	**Intervention:** practice patterns of NPs related to weight management in primary care **Comparator:** Usual care (General Practitioner). No comparator in several studies which was consistent with the design.	Narrative synthesis	No	NR
Ismail (2013) [102]	To review the evidence on primary care service interventions to reduce inappropriate accident and emergency attendances	N=4	N=1 Australia for NP study	$N_{(Total)}$= 34 (1986-2011)<br><br>N=1 NP study (2010)	**APN role:** Emergency NP **Population:** Residents in long-term care.	**Intervention:** Emergency NP providing first-line medical care to long-term care residents	Economic analysis	No	No
Jennings (2015) [103]	Determine the impact of emergency NP services on cost, quality of care, satisfaction and waiting times in Emergency rooms	N=4	N=6 UK, Australia, New Zealand, USA, Netherlands, Canada	N=12, excluded 2 previous reviews	**APN role:** Primary healthcare NP **Population:** Patient presenting to minor injuries clinic, walk in centres, Emergency room, accident and emergency, casualty, primary care clinics. Patients: n =36267	**Intervention:** NP services conducted on site in the Emergency room **Comparator:** Traditional Emergency room clinical services that do not include nurse led care, only medical lead services	Narrative synthesis	No	NR
Jeyaraman (2022) [104]	To identify, critically appraise and summarize evidence on the impact of primary healthcare	N=4	N=3 Australia, England, USA	$N_{(Total)}$= 40 studies reported in 44 papers.	**APN role:** Primary healthcare NP **Population:** Level 4 and Level 5 triage scale adult and pediatric patients seen in the Emergency room Patients: n = 4361	**Intervention:** NP triage team: NP located at the Emergency room triage area working alongside a triage nurse, either ordering investigations at triage before	Meta-analysis	Yes	NR

	professionals in the Emergency room triage, on patient flow.			N=14 NP studies		streaming to Emergency room physician.<br>**Comparator:** Traditional nurse-led triage model			
Johnson (2015) [105]	To review the metrics used to assess oncology NP navigation programs	N=9	N=2<br>USA, Netherlands	N=7<br>(2007-2013)	**APN role:** Oncology nurse navigation defined as: individualized assistance offered to patients, families, and caregivers to help overcome healthcare system barriers and facilitate timely access to quality health and psychosocial care<br>**Population:** Patients in oncology regardless of the type of cancer	**Intervention:** NP navigation in oncology use the navigation process to care for patients at any point along the cancer care continuum.<br>**Comparator:** Current standards of care.	Methodologic matrix was created	NR	Yes
Jokiniemi (2012) [146]	To analyze and synthesize the literature on one specialized APN role in three different countries to: (i) describe prerequisites, role domains, challenges affecting role implementation, and outcomes of advance practice nursing roles; (ii) determine if roles are consistent in the USA, UK, and Australia.	N=2	N=3<br><br>Australia, UK, USA	N=42<br>(1990-2011)	**APN role:** Nurse consultant in the UK, CNS in the USA, and clinical nurse consultant in Australia<br>**Settings:** Primary care, diabetes, palliative and end-of-live care, in early discharge and very low birth weight infant, stroke consultant, emergency room, psychiatric and mental health, health consultant, critical care, generalist community nurse, older adults.<br>**Population:** No restriction; population served by APN in UK, USA, and Australia	**Intervention:** APN roles in UK, USA, and Australia<br>**Comparator**: None	Qualitative content analysis	Not mentioned	NR
Joseph (2015) [106]	To gather and synthesize evidence on the safety and effectiveness of nurse-performed endoscopy in the	N=11	N=2<br>USA, Canada	N=6<br>(1994-2011)	**APN role:** Nurse endoscopists<br>**Population**: Males and females aged 45 years and older	**Intervention:** Nurse-performed endoscopy for colorectal cancer screening purposes.<br>**Comparator:** Medical endoscopists (physicians)	Thematic analysis	Yes	Yes

	bowel cancer screening.								
Kennedy (2012) [92]	To examine the impact of nurse consultant roles in adult care and identify patient and professional indicators	N=12	N=1 UK	N=36 (2002-2009)	**APN role:** Nurse consultant, CNS and NPs in adult acute or primary care settings. **Population**: Patients, staff and/or nurse consultants perceptions of the impact of nurse consultant-led care.	**Intervention:** Introduction of nurse consultant-led services or the addition/substitution of nurse consultants to existing services. **Comparator:** Usual care, another healthcare professional	Mixed method synthesis	NR	NR
Kilpatrick (2014)* [55]	To evaluate the cost-effectiveness of CNSs delivering outpatient care in alternative or complementary provider roles.	N=10	N=4 USA, Netherlands, UK, China	N=11 (1988-2012)	**APN role: CNS**: CNSs in **alternative roles** provide similar services to those for whom they substitute, usually doctors. CNSs in **complementary roles** augment existing services. **Population:** Care of children with asthma, patients with diabetes, men with bladder or prostate cancer undergoing radical radiotherapy, modes of interprofessional hospital-based care for adults with rheumatoid arthritis, mental health issues, heart failure, breast cancer, high-risk pregnancy, and residents in nursing homes	**Intervention**: CNSs in outpatient settings manage care of complex and vulnerable populations, the education and support of interprofessional staff, and facilitation of change within health care systems. **Comparator**: Usual care, physicians, hospital-based care	Narrative synthesis	Yes	Yes
Kilpatrick (2015)* [56]	To synthesize the evidence of effectiveness and cost-effectiveness of CNSs and NPs working in alternative or complementary roles in inpatient settings	N=10	N=2 USA, Canada	N=3 (1990-2001)	**APN role:** CNSs and NPs in alternative and complementary roles. **Population:** Patients in inpatient roles in tertiary-level neonatal intensive care unit, patients at risk of self-harm or harming others, and care on general medical wards	**Intervention:** CNS provided individualized consultations, investigated sitter request, assessed patient and intervention. CNS interventions targeted nursing staff, patients and sitters. NPs assumed responsibilities for documentation, assessment, identifying medical problems, revising management plans, implementing diagnostic and treatment regimens, and developing discharge plans. **Comparator:** Physicians, physician residents, standard care.	Narrative synthesis	Yes	Yes .

Koblede r (2017) [78]	To examine the effectiveness of APN interventions in gynecologic oncology in terms of improving quality of care and costs	N=9	N=3 Austria, England, USA	N=4 (2001-2017)	**APN role:** APNs were aligned with core competencies, such as those defined by Hamric et al., and according to the definition of the German, Austrian and Swiss professional associations. **Population**: Women 18 years and older, diagnosed with ovarian, uterine, cervical, endometrial, vaginal, vulvar carcinoma who were cared for in the inpatient/ outpatient/home setting	**Intervention**: Gynecology-specialized APN doing counseling interventions, telephone interventions or consultations or in association with a psychiatry-specialized APN **Comparator:** Standard care, counseling and referral to a specialist, symptom management of side effects, toolkit, standardized written information	Narrative	Yes	NR
Kuethe (2013) [85]	Review the effectiveness of nurse-led asthma care	N=6	N=3 Netherlands, UK, Australia	N=5 (2003-2011)	**APN role:** Primary healthcare NP **Population:** Asthma nurses, specialist nurses, respiratory nurses Primary care NP Patients with asthma (n =570)	**Intervention:** Any aspect of asthma management, led by an allied health professional (specialized asthma nurse, NP, physician assistant or specifically trained nursing professional), supervised by a physician **Control**: The same aspect of asthma management provided by a physician	Meta-analysis	Yes	NR
Kwok (2022) [99]	To determine the effectiveness of nurse-led telehealth symptom management interventions for patients with cancer receiving systemic or radiation therapy compared to usual care on health service use, quality of life, and symptom severity.	N=6	N=6 USA, Canada, France, Italy, Hong Kong, Turkey	N=10 (2006-2021)	**APN role:** Nurse-led telehealth interventions with patient-initiated contact and scheduled telehealth interventions initiated by nurses. **Population:** Patients with cancer, including all tumor types and stages of disease, receiving systemic (chemotherapy, targeted therapy, immunotherapy, hormonal therapy) or radiation therapy within 4 weeks of study enrollment.	**Intervention**: Nurses-led telehealth for cancer patients receiving systemic or radiation therapy. **Comparator:** Usual care (typically patient-initiated calls)	Meta-analysis	No	NR

Lawton (2018) [84]	To compare the effectiveness of nurse-led care versus doctor-led care in the management of stable bronchiectasis	N=6	N=1<br>UK	N=1<br>(2002)	**APN role:** Nurse specialist-led care for stable bronchiectasis: specialist care in an outpatient setting, managed or delivered by a nurse who provides chronic disease management for bronchiectasis through a minimum of two contacts over separate days over 12 months. Trials included only stable patients with an established management plan.<br>**Population**: Participants (n=80) with a mean age of 58	**Intervention:** Nurse specialist-led care in the management of stable bronchiectasis<br>**Comparator:** Doctor-led care	Descr iptive	No	Not clear
Leduc (2021) [93]	Determine the effectiveness and safety of interventions to evaluate and treat patients in long-term care to avoid un-scheduled Emergency room transport.	N=3	N=4<br>USA, Canada, Scotland, Norway	N=22<br>(2013-2018)	**APN role:** Primary healthcare NP<br>**Population:** Adult patients in long-term care<br>N for intervention and control groups not reported. Patient numbers not calculated as authors report number of patients and number of beds, depending on the study	**Intervention:** Intervention included a set of tools called Interventions to Reduce Acute Care Transfers (INTERACT).<br>**Comparator:** Standard care or transportation to the Emergency room	Narra tive revie w	Yes	NR
Lempp (2020) [82]	Examine the value, impact and nurses' professional roles in rheumatoid arthritis management	N=1	NR	$N_{(Total)}$= 20<br><br>N=6 APN studies	**APN role:** Care provided by specialist nurses for care provided conventionally by doctors or routine clinic nurses.<br>**Population:** Patients with rheumatoid arthritis	**Intervention:** Holistic care provided by nurses, the benefits of education and emotional support and the ability of nurses to facilitate patient-centred care and shared decision-making<br>**Comparator:** Physicians	Narra tive synth esis	Yes	NR
Loesche r (2018) [72]	To examine APNs' skin cancer knowledge, performance, attitudes, and barriers to clinical skin examination, recognition of skin lesions, and related training activities.	N=10	N=2<br>USA<br>England<br>NR	N=12<br>(2011-2016)<br>Update of a systemat ic review (2000-2010)	**APN role:** Primary healthcare NP<br>**Population:** NPs: N = 783<br>Patients/cases: N = 344<br>Control: N= 1255<br>Four studies reported participants' age, which ranged from 27 to 64 years and averaged 41 years.<br>Three studies reported years of practice ranging between two and 16+ years.<br>One study included NP students.	**Intervention**: Educational activities and training program lasting between 15 minutes to several months. Some studies included direct feedback from physicians on referrals and assessments.<br>**Comparator:** Usual care or no comparator depending on the design	Descr iptive	No	Yes

Lovink (2017) [34]	**Aim 1:** To evaluate the effects of substituting NPs, physician assistants or nurses for physicians in long-term care facilities and primary healthcare for the ageing population.<br>**Aim 2**: To describe what influences the implementation of these roles	N=6	N=4<br>Canada,<br>Japan,<br>Sweden,<br>USA	$N_{(Total)}$= 12 (1997-2015)	**APN role:** Primary healthcare NP<br>**Population:** Mean age of the older adults varied from 72 years to 86.3 years. Sample size varied from 114-2575. All patients ≥65 years old, or with a mean age of ≥70 years; Long-term care facilities and primary healthcare.<br>NP: n =49<br>Physicians: n = 273<br>Other Professional: n =27<br>Patients: n =4487	**Intervention:** Medical or preventive care for older patients provided by NPs, physician assistants, or nurses.<br>**Comparator:** Care as usual provided by a physician.	Narrative summary	No	NR
Lyness (2021) [130]	To understand how task-shifting can be implemented to optimize service effectiveness and patient care.	N=6	N=3<br>UK,<br>Norway,<br>Netherlands	N=6 (2011-2021)	**APN role:** All were involved in delivery of urgent care in out of office hours care.<br>**Population**: Study of non-medical providers (General Practitioners, medical assistants, receptionists)	**Intervention**: Implementation of out of office hours urgent care<br>**Comparators:** Physicians, medical assistants, receptionists	Thematic synthesis	Yes	No
Manoj (2019) [63]	To evaluate nurse-Led cardioversion procedures, outcomes include patient satisfaction, waiting time and cost-effectiveness	N=9	N=1<br>USA	$N_{(Total\ )}$=7<br><br>N=3 APN studies (2016-2017)	**APN role:** The delivery of synchronized direct current cardioversion is a short general anesthetic procedure delivered via paddles by NP and advanced practice provider (including APN)<br>**Population:** Patients with arrythmias requiring elective direct current cardioversion.	**Intervention:** APN-led procedures with medical-led procedures.<br>**Comparator**: Physician-led direct current cardioversion	Narrative synthesis	NR	Yes
Martin-Misener (2015)* [69]	Determine the cost-effectiveness of NPs delivering primary and specialized ambulatory care.	N=10	N=4<br>Netherlands,<br>UK,<br>USA,<br>Wales	N=11 (1991-2011)	**APN role:** Primary healthcare NP<br>**Population:** Patients all ages in the Emergency room, dermatitis, post-discharge lipid management, diabetes management, coordinate and manage	**Intervention**: NPs see patients at first point of contact, same day appointment, initial visits, colonoscopy, telephone follow-up, post-discharge lipid management, diabetes management, manage	Meta-analysis	Yes	Yes

					medically unexplained symptoms and high use of primary care.<br>NP: n=61<br>Physicians: n =98<br>Patients: n =7497	medically unexplained symptoms and high use of primary care.<br>**Comparator**: Physicians, usual care, care from dermatologist, gastroenterologists.			
Martinez-Gonzale (2014) [65]	To assess the impact of physician-nurse substitution in primary care on clinical parameters.	N=4	N=5<br>UK, Netherlands, USA, South Africa, Russia	$N_{(Total)}$= 11 (2000-2011)<br><br>N=8 NP studies	**APN role:** Primary healthcare NP<br>**Population:** Nurses provided care for complex conditions including HIV, hypertension, heart failure, cerebrovascular diseases, diabetes, asthma, Parkinson's disease and incontinence.<br>Excluding non-NP studies n= 4361	**Intervention:** No clear definition of NP roles. Included nurse roles where nurses had no advanced education or decision-making autonomy as NP roles.<br>**Comparator:** Physician care	Meta-analysis	Yes	NR
Massimi (2017) [64]	To assess the efficacy of the nurse-led self-management support versus usual care evaluating patient outcomes in chronic care community programs.	N=4	N=3<br>USA, UK, Netherlands	N=29 (2000-2013)	**APN role:** Any qualified nurse working as a substitute to a primary care physician focused on self-management support for chronic disease. This could include: NPs, CNSs, APNs, practice nurses, registered nurse.<br>**Population:** Patients >18 years old with a diagnosis of chronic disease or multiple morbidity including diabetes, multichronic conditions, and chronic obstructive pulmonary disease	**Intervention:** Nurse-led self-management support performed with any method of communication exchange or Nurse-led self-management support interventions in chronic patients education in a community setting<br>**Comparator:** Usual care, medical primary care provider (General Practitioner, primary care physician) without any structured educational intervention.	Meta-analysis	No	NR
McCrory (2018) [58]	To determine the impact of the addition of an Advanced NP on patient outcomes in adults with chronic kidney disease	N=4	N=2<br>Netherlands, Canada	N=4 (2011-2013)	**APN role:** Advanced NP in chronic kidney disease management<br>**Population**: Adults aged 18 years and over, with chronic kidney disease stages 2–4	**Intervention:** Input of an Advanced NP in addition to the provision of usual care<br>**Comparator:** Physicians, usual care.	Meta-analysis	NR	Yes
McMenamin (2023) [47]	To identify the effect of NP primary care models with and without NP involvement, on cost, quality, and service utilization	N=6	N=1<br>US only	N=15 (2021-2003)	**APN role:** NPs providing primary care<br>**Population**: Patients were adults (age ≥18) with ≥2 chronic conditions (including behavioral health or substance use disorders) in primary care	**Intervention:** NP care could be delivered either as a solo NP provider or as part of an interdisciplinary team.<br>**Comparator:** Physician, physician assistant delivered care without NP involvement, usual care	Narrative synthesis	Yes	NR

	by patients with multiple chronic conditions.					.			
McParland (2022) [35]	To identify types of nurse-led interventions for multimorbidity and which outcomes are positively affected by them.	N=5	N=8 Canada, Hong Kong, Israel, Portugal, Spain, Sweden, USA, UK	N=20 reported in 28 publications	**APN role:** Primary healthcare NP<br>**Population:** Patients with multi-morbidity defined as the coexistence of ≥2 chronic conditions<br>Average age of participants reported in 13 studies, range: 38.0 to 83.1 (median: 75.6, IQR: 73.9–76.5).<br>Patients: n = 43 899<br>Providers: n = 19	**Interventions:** Nurses in advanced practice develop care plans in partnership with patients, to simplify and improve the quality of care both in the long and short-term using case-management, transitional care interventions, support to self-manage conditions, and an emphasis on continuity of care.<br>**Comparator:** Physician-led care, usual care	Narrative synthesis	Yes	NR
McQuilkin (2020) [59]	To identify key program components of successful transition from NP to new faculty	N=6	N=6 USA, Canada, Australia, Ireland, UK, Iran	N=31 (2008-2017)	**APN role:** NP moving into faculty positions<br>**Population**: NPs, clinicians and new NP faculty	**Intervention:** Programs to facilitate the role transition from clinician to NP clinical faculty<br>**Comparator:** Universities with general programs to facilitate the transition for new faculty	Narrative synthesis	NR	Yes
Medeiros (2011) [94]	To evaluate midlevel practitioner practice on intensive care unit outcomes: patient satisfaction, length of stay, mortality and resource utilization/cost	N=2	N=1 USA only	N=5 (1990-2005)	**APN role:** NPs and CNSs<br>**Population:** Only those in adult intensive care units	**Intervention:** Care provided by physician assistants, NPs, or CNSs in intensive care units<br>**Comparator**: Physician residents, attending team	Narrative synthesis	NR	Yes
Mileski (2020) [49]	To increase the understanding of the role an NP has in reducing the risk of hospitalizations and improving quality outcomes	N=3	NR	N=14 (2004-2019)	**APN role:** Primary healthcare NP<br>**Population:** Patients in long-term care, aged care, skilled nursing facilities, Long-term care in hospitals, inpatient rehabilitation<br>Patients: n =1562	**Intervention: NR**<br><br>**Comparison: NR**<br><br>The review **did not include comparisons** of physicians to NPs in relation to hospital readmissions	Rate of occurrence	No	NR

	among nursing facility residents.								
Monterosso (2019) [60]	To determine the effectiveness of nurse-led cancer survivorship care, compared with existing models of care, on patient reported outcomes for cancer survivors.	N=12	NR	N=21 (2007-2017)	**APN role:** Nurse-led cancer survivorship care APNs. In most models of care, the nurse's role included at least two of the following elements: patient assessment, general management of a problem, and patient education or advice<br>**Population:** Adults (age ≥18 years) <2 years post treatment completion, all malignancies included	**Interventions:** Cancer nurse-delivered cancer care, the type of care included: monitoring; treatment and/or referral for recurrence; or assessment and treatment for side effects of treatment or quality of life needs including physical, psychosocial, functional, financial, insurance, occupational, fertility, sexual function, sexuality and spiritual needs.<br>**Comparator:** Physician-led follow-up, conventional nursing care, usual multi-disciplinary hospital care, no intervention, or usual care	Meta-analysis<br><br>Narrative synthesis	Yes	NR
Morilla-Herrera (2016) [36]	To identify, assess and summarize the evidence of the effect of APN interventions deployed when providing care to older people in different care settings, and to describe the roles and components of the interventions developed by these professionals.	N=16	N=7 Sweden, USA, New Zealand, UK, Denmark, China, Switzerland	$N_{(Total)}$= 15 (1990-2014)<br><br>N=14 were extracted	**APN role:** Primary healthcare NP<br>**Population:** Patients' age was over 65 years old in all the studies. Health conditions more frequently reported: dementia, hip fractures, chronic heart disease and multimorbidity.<br>**Settings:** Transitional care, ambulatory care, home care, hospital care.<br>Patients: n =4749	**Intervention:** NPs completed health screening; consultations; case management; discharge planning; telephone follow-up; program development; referral; guidance and support through the health system for patients and caregivers; support for patients and caregivers; health education<br>**Comparison**: Usual care or control group	Narrative synthesis	No	NR
Ness (2016) [125]	To explore the influence on antimicrobial prescribing behaviour of	N=8	N=3 UK, USA, Lesoto	N=7 (2001-2013)	**APN role:** Primary healthcare NP<br>**Population:** Study participants were nurse prescribers who could independently prescribe.<br>NP: n=2022	**Intervention:** Nurse prescriber's decision to prescribe an antimicrobial or not.<br>**Comparator:** No comparator	Narrative synthesis	No	NR

	independent nurse prescribers.								
Newhouse (2011)/ Stanik-Hutt (2013) [39-40]	Compared to other providers, are Advanced Practice Registered Nurse patient outcomes of care similar?	N=3	N=1<br>USA	$N_{(Total)}$ =69<br>NP studies: N=37<br>CNS studies: N=7<br>(1990-2008)	**APN role:** NPs and CNSs<br>**Population:** Patients in the community or nursing homes, with coronary artery disease, diabetes, chronic conditions, hypertension, HIV/AIDS, post partum, end-of-life care, undergoing radical prostatectomy, post total knee replacement	**Intervention:** NP or CNS care provided alone or in a team<br>**Comparator:** physicians or teams without Advanced Practice Registered Nurses	Narrative synthesis	Yes	Yes
Niezen (2014) [107]	To identify facilitators and barriers to reallocating tasks from the traditional domains of medicine to nursing.	N=2	N=7<br>Australia, Canada, Israel, Netherlands, Sweden, UK, USA	N=13 (2000-2012)	**APN role:** NPs<br>**Population:** Considered a broad spectrum of task reallocation (delegation, substitution, complementary care) from various healthcare contexts including long-term care; geriatrics, acute care hospitals, community clinics, general practice	**Intervention**: N/A<br>**Comparator**: N/A	Thematic analysis	Yes	NR
Nikpour (2022) [112]	To examine opioid and non-opioid prescribing patterns of physicians, NPs, and physician assistants in primary care	N=4	N=1<br>USA	N=17 (1991-2020)	**APN role:** NP role<br>**Population:** Adults in primary care settings being prescribed opioids	**Intervention:** Pain management<br>**Comparator:** Physician and physicians assistant prescribing patterns for opioids	Narrative synthesis	NR	Yes
Norful (2019) [66]	To synthesize available studies that compare the effects of NP/physician co-management to physician management in primary care.	N=5	N=1<br>USA only	N=6 Studies less than 20 years old	**APN role:** Primary healthcare NP<br>**Population:** Patients in primary care Most common diagnoses included Alzheimer's dementia, diabetes, hyperlipidemia, and hypertension. Incomplete reporting of n for intervention and control groups	**Intervention**: NP-physician co-management teams<br>**Comparator:** Individual physician-led care	Narrative synthesis	No	Yes

Ordonez-Piedra (2021) [95]	To update literature on the effectiveness of Advanced Practice Nursing interventions for patients with heart failure.	N=4	N=7 USA, China, Australia, Brazil, Sweden, Philippines, France	N=11 (2012-2017)	**APN role:** APN is a specialist who has acquired clinical skills to make complex decisions for a better professional practice<br>**Population:** Patients with heart failure	**Interventions:** Education for patients to detect signs and symptoms of the disease, sign of exacerbations, resources to use in different situations, information about social and personal consequences of the disease, lifestyle management.<br>**Comparator**: Usual care, physicians, other healthcare professionals	Narrative synthesis	NR	NR
Osakwe (2020) [37]	Examine the current evidence on health and healthcare utilization outcomes associated with NP-home visits.	N=5	N=3 Canada, UK, USA	N=7 Published before April 2019	**APN role:** Primary healthcare NP<br>**Population:** Patients receiving NP home visits, assessment of medical, psychological and functional abilities of older adults, care coordination, education, and medication management<br>Patients: n =1757	**Intervention:** Patients receiving home visits from NPs<br>**Comparator:** Usual care	Narrative synthesis	No	Yes
Patel (2019) [108]	To systematically examine the empirical evidence that links NP scope of practice and its impact on access to care.	N=4	N=1 USA only	N=13 (2013-2017)	**APN role:** Primary healthcare NP<br>**Population:** Characteristics of study participants not reported.<br>National sample used in nine studies, primary care services, community health centers, facilities with mammography services<br>NP: n =156 851<br>Physicians: n =149 784<br>Other Professionals: n =95 545<br>Patients: n =4 528 200	**Intervention:** Scope of NP practice and access to care.<br>**Comparator:** Physicians, certified nurse midwives, physician assistants	Narrative synthesis; content analysis	No	NR
Ramis (2013) [145]	To examine the field of advanced practice within Australian acute care settings and to uncover APN experience of practice, as reported by APNs themselves.	N=8	N=1 Australia	N=4 (1996-2008)	**APN role:** Advanced practice registered nurses working in roles within acute, hospital, or tertiary care centres, intensive care and critical care units as well as hospital Emergency rooms.<br>**Population:** APNs	NR<br><br>This was a qualitative study and that was not part of the process.	Meta-syntheses	Yes	NR

Raymond (2022) [148]	To characterize NPs' involvement and experience with artificial intelligence-based health technology	N=4	NR	N=11 (2018-2022)	**APN role:** NPs and NP students in hospital (acute), emergency and primary care<br>**Population:** Patients in specialized tertiary care, Emergency room, pediatric settings, post-operative, acute and post-acute care, primary care	**Intervention**: NP involvement and experience in the development and use of artificial intelligence- based technologies, clinical decisions support systems, and their influence on clinical care<br>**Comparator**: Physicians, physician assistants and/or nurses. NPs and physicians become own comparison for clinical decisions and diagnostic accuracy.	Narrative synthesis	No	NR
Rutherford-Hemming (2016) [118]	Examine the use of simulation in NP education to determine if enough empirical evidence is available to support the use of simulation in lieu of traditional direct patient care clinical hours.	N=2	N=1 USA	N=15 (2010-2015)	**APN role:** NP students<br>**Population:** Simulation scenarios primarily in critical care	**Intervention**: High-fidelity simulators, intermediate-fidelity manikin, role-playing with faculty and/or student simulations using standardized patients.<br>**Comparato**r: Usual education method, usual debriefing, no comparator	Narrative synthesis	NR	Yes
Salamanca-Balen (2018) [126]	To review international evidence on the costs, resource use and cost-effectiveness of CNS–led interventions for patients with palliative care needs.	N=4	N=13 USA, UK, Austria, Australia, Canada, Denmark, Netherlands, New Zealand, Spain, Sweden, Taiwan, Thailand	N=79 (2000-2015)	**APN role:** Advanced NP/APN, CNS, NP, Practice Nurse, Specialist nurse, nurse specialist<br>**Population:** Patients aged 60 years and older with a concomitant chronic health condition, cancer, advanced heart, lung, renal or liver disease, dementia, epilepsy and neuro-degenerative diseases, HIV/AIDS, arthritis, diabetes and leg ulcers, frail elderly (e.g. living in residential care), patients in the intensive care unit who were chronically and critically ill.	**Intervention:** CNS-led interventions in which the clinical component was predominant included activities directed to the diagnosis, treatment and control of the disease and were performed in hospitals, outpatient clinics or patients' homes.<br>Supportive interventions to increase patients' knowledge about their disease and its management, and at the same time aimed to give psychological/spiritual support, care coordination.<br>**Comparator**: Usual care, physicians	Narrative synthesis	Yes	NR

Schadewaldt (2011) [67]	To identify effective interventions in nurse-led cardiac clinics including patient education, risk factor assessment and continuity of care.	N=30	N=5 England, Scotland, Australia, China, Canada	N=7 (2002-2008)	**APN role:** Primary healthcare NP **Population:** Adults (aged >18 years) admitted to a hospital or a general practice with newly diagnosed or existing coronary heart disease, that is angina pectoris symptoms and myocardial infarctions. Patients: n =3246	**Intervention:** Assessment, monitoring and consultation on risk factors, education sessions and promotion of regular intake of medication, and adherence to a healthy lifestyle. **Comparator:** Usual care provided by a physician	Narrative synthesis and meta-analysis	No	NR
Schallmo (2019) [119]	To document if current program curriculum align with current procedures and skills in the clinical setting for primary healthcare NPs	N=5	N=1 USA	N=9 (2000-2016)	**APN role:** APNs in primary care (family NP, adult gerontology NP, pediatric NP, or women health NP) where procedural skills were acquired in a master or doctoral program. Procedural skills were performed in primary care, ambulatory care, or Emergency room setting. **Population:** Care to individuals and families across the lifespan	**Intervention:** Educational programs for procedures in NP education. **Comparator:** N/A	Thematic analysis	NR	Yes
Scheydt (2021) [77]	To describe advanced psychiatric nursing roles, and to distinguish them from the other roles of psychiatric and general nursing care.	N=3	N=7 Great Britain, USA, China, Switzerland, Germany, Austria, Hong Kong	N=20 (1989-2019)	**APN role:** Psychiatric-Mental Health CNS, Psychiatric-Mental Health NP, and blended role (CNS/NP) were examined. **Population**: Nor reported	**Intervention:** No specific intervention for advanced psychiatric nursing roles **Comparator:** Nurses in psychiatric and general care roles	Qualitative thematic analysis	No	NR
Schneider (2021) [78]	To search for evidence of training of APNs, through clinical practice and nursing care.	N=6	N=5 USA, Canada, England, Netherlands, South Korea	N=12 (2000-2017)	**APN role:** APN, CNS, NP **Population:** Patients with cancer receiving chemotherapy, palliative care, treatments for gynecological cancer, breast cancer, lung cancer, pediatric oncology, cancer survivors and home care.	**Interventions:** Educational guide-lines, telephone counseling, care coordination, symptom management and control, clinical evaluation, assistance clinical decision-making. **Comparator:** Usual care	Narrative	Yes	NR
Schoch (2014) [135]	To assess the effects of renal access coordinators on dialysis patient	N=11	N=4 Australia, Netherlands, USA,	N=5 (2004-2012)	**APN role:** CNS, Renal access coordinator provides a communication pathway between nephrology and vascular teams, pre dialysis access coordination,	**Intervention:** The introduction of renal access coordinators and its role in the haemodialysis unit. **Comparator:** Often not reported, sometimes pre-post or units	Narrative	NR	NR

	outcomes and associated service delivery, assess the level of evidence available and identify gaps in the literature.		Canada		access surveillance, patient education and nurse education<br>**Population:** Adults over the age of 18 years who were currently receiving hemodialysis within a renal unit.	without renal access coordinator role.			
Scott (2011) [68]	To determine the extent to which the Emergency room can be used to screen patients for undiagnosed hypertension and estimate the incidence of undiagnosed hypertension in this population	N=7	N=2 US, UK	N=4 (2003-2008)	**APN role:** Primary healthcare NP<br>**Population:** Participants age ≥18 years; no prior history of hypertension; minor Emergency room setting; minimum of two Emergency room blood pressure measurements; systolic blood pressure ≥140 mmHg and/or diastolic blood pressure ≥90 mmHg; and post-discharge follow-up of blood pressure.<br>Patients: n =2767	**Intervention:** Hypertension screening in the Emergency room<br>**Comparator:** Usual care	Meta-analysis was planned but not conducted	No	Yes
Searle (2023) [133]	To synthesize evidence on interventions that influence unplanned hospital admissions or attendances by long-term care facility residents.	N=6	N=2 Australia, New Zealand	$N_{(Total)}$= 43<br><br>N=3 NP/CNS studies (2012-2022)	**APN role:** NPs and CNSs in gerontology<br>**Population:** Residents living in nursing homes	**Intervention**: NPs provided autonomous with independent diagnosis and prescribing. Gerontology CNSs provided clinical support, education and coaching.<br>**Comparator**: Usual care	Narrative synthesis	NR	NR
Smigorowsky (2020) [50]	To assess randomized controlled trials evaluating the impact of NP-led cardiovascular care.	N=7	N=3 Canada, USA, Netherlands	N=5 (2007-2017)	**APN role:** Primary healthcare NP<br>**Population:** Over 18 years of age, outpatient heart failure care, postoperative cardiac surgery, outpatient risk reduction clinic, diagnosed with cardiovascular disease (e.g. coronary artery disease, arrhythmias)<br>Patients : n =887	**Intervention:** NP-led care included completed assessments, diagnosed new findings, ordered and monitored medications/diagnostic tests. The NP could also consult other healthcare providers to give specialty services.<br>**Control:** Usual care	Narrative synthesis, meta analysis	No	Yes

Smith (2014) [73]	Review current literature on participation and roles of Advanced Practice Registered Nurses/Physician Assistants in providing cancer screening and prevention recommendations in primary care settings in the United States	N=2	N=1 USA	$N_{(Total)}$= 15 (1990-2011)<br><br>N=13 (excluded 2 studies reporting on registered nurse midwives)	**APN role:** Primary healthcare NP<br>**Population:** Patients in primary care receiving cancer screening and illness prevention care<br>NP: n=1698 (reported intervention group only)<br>Physicians: n =3758<br>Other Professionals: n =2197 (Intervention group only)<br>Patients: n =94611	**Intervention:** Advanced Practice Registered Nurses/Physician Assistants, cervical, breast, or colorectal cancer screening, smoking cessation, diet and physical activity.<br>**Comparator:** Physicians, other provider, and no comparison group.	NR	No	NR
Speight (2019) [120]	To synthesize evidence regarding the effectiveness of interventions and strategies to promote professional transition of new graduate NPs	N=3	N=1 USA	N=4 studies reported in 5 articles (2014-2019)<br><br>Only published in English	**APN role:** New graduate NPs (i.e., graduation within the past 5 years) transitioning into professional practice<br>**Population:** Practice environments included six Veterans Administration primary care sites and one primary care federally qualified health care center.	**Intervention:** Two main interventions were reported: fellowship programs up to 12 months and a two-hour webinar.<br>**Comparator:** None reported as several studies used before and after measurements of participants.	NR	NR	Yes
Stratton (2020) [121]	Evaluate current interventions targeting clinical skin education education for primary care NPs	N=4	N=3 Australia, UK, USA	N=10 (1998-2016)	**APN role:** Primary healthcare NP<br>**Population:** NP: n=5<br>Physicians: n =502<br>Other Professionals: n =1340<br>Patients: n =628<br>Only 2 studies provided the sample gender and age range	**Intervention:** Didactic portion to review of epidemiology or skin lesion management using feedback from the NP's prior dermatology referrals to guide her education.<br>**Comparator:** None identified	Narrative	No	Yes
Sun (2022) [38]	To synthesize research evidence of NP visits in home-based primary care.	N=6	N=1 USA	N=14 studies published in 17 papers	**APN role:** Primary healthcare NP<br>**Population:** Mainly homebound adults and older adults receiving home care services for heart failure, intellectual or developmental disability, poverty,	**Intervention:** NPs provided health assessments, education, care planning and coordination primarily by face-to-face home visits. Most studies included adults but two	NR	Yes	Yes

					uncontrolled diabetes, children with special healthcare needs, asthma<br>Patients: n = 4957	studies included children with special healthcare needs or asthma<br>**Comparator:** No comparator in half the studies, physicians and/or social workers			
Swan (2015) [51]	To determine the safety and effectiveness of primary care provided by APNs and evaluate their potential to alleviate primary care shortages.	N=3	N=5<br>Canada, England, Wales, Netherlands,<br>USA	N=10 (1974-2011)	**APN role:** Primary healthcare NP<br>**Population:** Patients in primary care. Follow up single consultation, 2 to 4 weeks, or over 12 months.<br>NP: n=33<br>Physicians: n =71<br>Patients: n =9852	**Intervention:** Patients presented for a general or condition focused primary care (blood pressure and lipid management), same-day consultation for any reason, or a pre-defined list of conditions including asthma, diabetes mellitus and hypertension<br>**Comparative:** General practitioner or physician	Narrative synthesis	No	NR
Thamm (2019) [109]	The primary aim was to evaluate the effectiveness of nurse-led interventions on Emergency room length of stay. Secondary aims were to evaluate the effectiveness of nurse-led interventions on mortality, readmission to Emergency room or re-examination, cost utility, and patient and staff satisfaction.	N=3	N=3<br>Australia, England, Scotland	N=6 (1999-2013)	**APN role:** NP role in the Emergency room<br>**Population:** Patients (18 years of age or more) presenting to the Emergency room irrespective of presentation	**Interventions**: Nurse-led interventions addressing the physical, education and/or psychosocial needs of patients<br>**Comparator:** Physicians.	NR	Yes	NR
Thomas (2019) [110]	To assess the effects of interventions for treating urinary incontinence after stroke in adults at	N=8	NR	$N_{(Total)}$= 20 (1996-2017)	**APN role:** Primary healthcare NP<br>**Population:** Adults at least one-month post-stroke<br>Patients: n =232	**Intervention:** Structured assessment and management by continence NPs<br>**Comparator:** Control groups were 'usual care' or no treatment.	Narrative synthesis	Yes	NR

	least one-month post-stroke.			N=1 NP study (2000)					
Tsiachristas (2015) [52]	Investigate the impact of new professional roles on a wide range of health service outcomes and costs	N=3	N=7 Australia, Canada, Norway, Sweden, Netherlands, UK, USA	$N_{(Total)}$= 41<br><br>N=16 NP studies (1994-2012)	**APN role:** Primary healthcare NP<br>**Population** with different disease including cancer, diabetes, rheumatoid arthritis, and cardiovascular risk. Studies evaluated skill-mix change in chronic diseases such as multiple sclerosis, chronic respiratory disease, Parkinson's disease. A few studies focused on the general population or acute care.<br>Patients: n =11 334	**Intervention:** Interventions include consultations and follow-up by NPs, first contact point, follow-up, and case management.<br>**Comparator:** Usual care	Narrative synthesis	No	NR
Turi (2023) [81]	To synthesize evidence of effectiveness of NP-delivered care to patients with mental health conditions (anxiety, depression, substance use disorders) in primary care	N=9	N=1 USA only	N=17 (2015-2022)	**APN role:** Primary healthcare NP<br>**Population:** Adult patients (18+ years of age) with diagnoses or symptoms of anxiety, depression, or substance use disorders, multiple drug use, or alcohol.<br>Patients: n_{total}= 8 975 685	**Intervention:** NP-delivered care (any NP specialty) practicing in a U.S. primary care setting (self-identified as occurring in the primary care setting)<br>**Comparator:** All existing alternative interventions (e.g., physician or physician assistant-delivered care), usual care	Narrative synthesis	Yes	No
Van Camp (2013) [97]	To synthesize the effect of nurse-led interventions on adherence to medication	N=2	N=6 Canada, USA, Niger, UK, Spain, Kenya	N=10 (2006-2011)	**APN role:** Nurse-led individual counselling was most frequently investigated.<br>Experiments with electronic messages and Nurse-led group sessions were reported.<br>**Patient populations** included HIV-positive individuals, patients with depression, arthritis and hypertension	**Intervention:** 3-6 month counselling where the majority of interventions constituted of multifaceted strategies, tailored interventions, combining counselling with education, social support and practical help (provision of alarm devices)<br>**Comparator:** Usual care	Meta-analysis	NR	NR
van Vliet (2020) [53]	To describe the activities of NPs and physician assistants working in ambulance care, and the effect of these	N=6	N=3 UK, Netherlands, USA	N=4 (2001-2019)	**APN role:** Primary healthcare NP<br>**Population:** Professionals with a master's degree in ambulance care.<br>NP: n =26<br>Other Professionals: n =3<br>Patients: n =2281	**Intervention:** NPs in ambulance care<br>**Comparator:** Paramedics, nurses, usual care, no comparator	Tabular form.	No	NR

	activities on patient, processes of care, costs, and provider outcomes								
Veenema (2021) [122]	To seek evidence to support that NP education and training align with current practices in the Emergency room.	N=4	N=1 Only studies USA	N=30 (2010-2020)	**APN role:** APNs primarily Family NPs providing care in Emergency rooms. NPs are nationally certified to address urgent and non-urgent types of patient conditions, similar to primary or urgent care providers treating patient conditions in nonhospital settings. **Populations:** Patients classified as high-acuity and low-acuity in the Emergency room.	**Intervention:** Education to prepare APNs for Emergency room practice **Comparator:** No comparator, physician, self-report	Them atic analy sis	No	Yes
Wang (2019) [74]	To evaluate the impacts of nurse-led clinic and nurse-led prescription on hemoglobin A1c control in type 2 diabetes.	N=8	N=4 America, Ireland, China, Netherlan ds	N=17 (1998-2015)	**APN role:** Similar to NP role **Population:** Patients with type 2 diabetes	**Intervention**: Nurse-led prescription was prescribed by nurse independently and compared with that of doctor **Comparator**: Physician care, standard care	Meta - analy sis	NR	NR
Wang-Romjue (2018) [142]	To describe and understand the experiences of NPs regarding their practice autonomy and roles in various ambulatory settings.	N=6	N=1 USA	N=9 (2000-2017)	**APN role:** NPs' practices and the ability of NPs to freely exercise professional judgment in patient care delivery in the ambulatory care. **Population:** Staff, interprofessional team, employers, patients in primary care	**Intervention:** Contextual characteristics, patients' experiences with NPs' roles and NPs' experiences with their practice autonomy and roles **Comparator:** N/A	Quali tative meta - synth esis	N/A	NR
Warren (2016) [123]	To synthesize evidence about the effectiveness of high fidelity simulation in NP education programs worldwide.	N=10	n=1 Only conducted in the USA	N=10 (2007-2014) English language only	**APN role:** NP education programs: NP students in acute and primary care. **Population:** NP students	**Intervention:** Adult scenarios: acute coronary syndrome, acute abdominal pain, respiratory distress, mechanical intubation, cardiac arrest, pneumonia, sepsis, and rapid atrial fibrillation. Pediatric scenarios: sepsis, respiratory distress, asthma exacerbation, a mock "code".	Narra tive synth esis	NR	Yes

						**Comparator**: On-line learning, traditional classroom learning, seminars, students served as their own controls			
Whiteford (2016) [111]	To examine the effectiveness of nurse-led Ears-Nose-Throat clinics for patients with chronic Ears-Nose-Throat complaints.	N=7	N=1<br>UK	N=3 (1997-2011)	**APN role:** Ears-Nose-Throat NP and nurses with advanced skills leading care (e.g., decision-making autonomy)<br>**Population:** Adult patients, aged 18 years and older, attending Ears-Nose-Throat clinics, regardless of the complaint, duration or type.	**Intervention:** Nurse-led care in general practice and acute care where nurse was identified as taking a lead role in the care of patients with chronic ear, nose and throat complaints.<br>**Comparator**: General practitioner-led care or Ears-Nose-Throat consultant-led care	Narrative	NR	NR
Woo (2017) [96]	To synthesize the best available evidence on the impact of advanced practice nursing on quality of care, clinical outcomes, patient satisfaction, and cost in emergency and critical care.	N=9	N=5<br>UK, USA, Canada, Australia, New Zealand	N=15 (2006-2017)	**APN role:** APNs or NPs working collaboratively or as an independent care provider<br>**Population**: >16 years of age, presenting in Emergency rooms, trauma centers, intensive care unit , or high dependency units, requiring emergency or critical care	**Intervention**: APN-/NP-directed emergency or critical care services provided in a wide range of circumstances. Some examples include minor injuries and fast-track patients in the Emergency room, discharge planning, care coordination, patient education on disease process and self-care, and post-discharge telephone follow-ups in the intensive care unit.<br>**Comparison:** Physicians, intensivists, physician residents, medical officers, hospitalists, or house officers in the Emergency room or intensive care unit or high dependency units	Narrative	Yes	Yes
Wu (2020) [113]	Explore the effectiveness of nurse-led interventions to prevent urinary tract infections in older adults living in residential aged care.	N=8	N=1<br>USA	$N_{(Total)}$=4<br>N=1 for NPs (2015)	**APN role:** Primary healthcare NP<br>**Population:** Long-term care residents<br>Mean age: 79.6 ± 8.07 (range 66–90) years<br>Patients : n =87	**Intervention:** Nurse-led interventions to prevent or manage urinary tract infection: (a) nursing education interventions to prevent urinary tract infections, (b) complementary/alternative therapies to prevent urinary tract infections, such as using cranberry products, (c) early detection of urinary tract infections, and (d) urinary catheter care. NP provided	Narrative synthesis	Yes	NR

						evidence-based supportive management for residents with asymptomatic urinary tract infections<br>**Comparator:** Usual care			
Xue (2016) [143]	To examine the impact of state scope of practice regulations on (a) NP workforce, (b) access to care and health care utilization, and (c) health care costs.	N=4	N=1<br>USA only	N=15<br>(1997-2015)	**APN role:** NP Scope of practice Regulation<br>Included studies did not clarify the NP or Advanced Practice Registered Nurse specialty. Possible that some studies included acute care NPs [or other specialties] or other types of Advanced Practice Registered Nurses (CNS, certified nurse-midwife, etc.)	**Intervention**: Scope of practice defined as full scope of practice regulation (independent practice and prescriptive authority), (b) reduced scope of practice regulation (requires a collaborative agreement with physicians for at least one practice component such as prescription), and restrictive scope of practice regulation (requires supervision, delegation, or team management by physicians<br>**Comparator**: Physicians	Them atic sum mari es	Yes	NR
Yang (2021) [54]	Synthesize evidence of the impact of state NP practice regulations on U.S. health care delivery outcomes	N=4	N=1<br>USA only	N=33<br>(2000-2019)	**APN role:** Primary healthcare NP<br>**Population:** US Census data, administrative claims insurers, Medicare/ Medicaid patients, medical expenditure Panel Survey (1996-2008), retail clinics, American Diabetes Association, National Ambulatory Medical Care Survey, AMA, 2013, AANP, 2012 Census Block<br>NP: n =4453<br>Patients: n =16 003	**Intervention:** Scope of practice regulation for care provided by NPs<br>**Comparator:** Care provided by physicians, physician assistants	Narra tive synth esis	No	NR
Zhang (2020) [127]	To understand current status of non-physician providers in Pre-exposure prophylaxis care implementation in the US	N=4	N=1<br>USA only	N=26<br>No date specified	**APN role:** Primary healthcare NP<br>**Population:** Pre-exposure prophylaxis care for patients with HIV/AIDS<br>NP: n=2842<br>Physicians: n =9789<br>Other Professionals: n =5052	**Intervention**: Pre-exposure prophylaxis care provided by NPs<br>**Comparator:** Usual care provided by physicians	Meta - analy sis	No	NR

APN, advanced practice nurse; CNS, clinical nurse specialist; HIV/AIDS, human immunodeficiency virus/acquired immunodeficiency syndrome; NP, nurse practitioner.

* Systematic review published as constellation papers, with additional methodological data extracted from: **154.** Marshall D, Donald F, Lacny S, Reid K, Bryant-Lukosius D, Carter N, et al. Assessing the quality of economic evaluations of clinical nurse specialists and nurse practitioners: A systematic review of cost-effectiveness. NursingPlus Open. 2015;1(2015):11-7.doi:10.1016/j.npls.2015.07.001; and **155.** Donald F, Kilpatrick K, Reid K, Carter N, Martin-Misener R, Bryant-Lukosius D, et al. A systematic review of the cost-effectiveness of nurse practitioners and clinical nurse specialists: What is the quality of the evidence? Nurs Res Pract. 2014;2014. doi:10.1155/2014/896587.
