# Supplementary material for: A global perspective of advanced practice nursing research: A review of systematic reviews
Source: PLoS One. 2024 Jul 2;19(7):e0305008. doi: 10.1371/journal.pone.0305008 (PMC11218965; doi:10.1371/journal.pone.0305008)
Supplement: S3 Table — (PDF) [file pone.0305008.s007.pdf]

S3 Table. Extractions of review results by indicator category at the Patient level.

Role	Author (year)	Ref	Results
			**Activities of Daily Living (ADLs) (9 reviews)**
APN Acute	Allsop (2021)	[10]	Function—activities of daily living (ADL) was reported in 5 studies. Two studies found no significant differences. Two studies found that patients had reduced functioning at discharge but had regained almost all their pre-morbid level at 12 months. One study found a significant improvement following the implementation of a discharge planning intervention
APN Acute	Audet (2021)	[7]	Functional status: No significant difference was identified between groups
NP Primary care	Lovink (2017)	[34]	Activities of daily living in LTC: significant improvement in 1/1 study (p = 0.04) Health status and functional ability 14 other outcomes, no significant effects were found in 1/1 study in LTC
NP Primary care	McParland (2022)	[35]	Physical Functioning (1 study): Trend towards improved function in the intervention group across the three time points while control declined (no p value reported)
NP Primary care	Morilla-Herrera (2016)	[36]	Functional status: 2/5 studies found significantly better functional status based on the independence to develop IADL [OR (95%CI):0.6(0.3–1.0); P = 0.04]; [OR(95%CI):3.0 (0.6–5.4); P: 0.02] and combined BADL and IADL[OR (95% CI):2.9(4.0–5.4); P: 0.03]; 2/5 found no differences and 1/5 found a trend in improvement (no p-value reported)
APN Primary Care	Newhouse/ Stanik-Hutt (2013)	[39-40]	Functional status: Eight studies (five RCTs); 2/8 studies reported findings favoring the NP group and 6/8 reported no differences between groups (no p-values reported).
NP Primary Care	Osakwe (2020)	[37]	Dependency in basic activity of daily living: The odds were significantly lower in the intervention group compared to the control group (adjusted odds ratio, 0.4; 95% CI, 0.2 – 0.8; p = 0.02). Nine people in the intervention group (4%) and 20 people in the control group (10%) were permanently admitted to nursing homes (P = 0.02). (1/1 study)
NP Primary Care	Sun (2022)	[38]	School attendance of children with special needs (1 study): Providing NP home visits to children with special needs significantly improved school and work attendance for children and parents. Parents missed less work (pre vs post 26.3% s 14.1%, p <.05); missed schools >20 days (pre vs post 10.4% vs 11.7%, p >.05).
NP Primary Care	Donald (2015)* (transition)	[33]	Functional independence: Estimates favoured nurse practitioner care
			**Adaptation to Health Conditions (9 reviews)**
APN Primary Care	Chan (2018)	[42]	Self-management and behavioural outcomes were reported in 5 studies and showed equal to statistically significant improvements for Medicaid knowledge and skills competence, managing osteoarthritis, reductions in active smoking, overall self-management score, medication knowledge, knowledge of condition, healthy lifestyle, diet and nutrition and medication adherence.
NP Primary Care	Baker (2017)	[41]	Self-Efficacy was reported in 3 studies with trends toward improvement noted in 1/3 studies and statistically significant improvements noted in 2/3 studies
APN Acute	De Thurah (2017)	[43]	Adaptation to health conditions was measured using self-efficacy after 1year, statistically significant difference was seen in favour of nurse-led follow-up (SMD: 0.30 (95% CI 0.07 to 0.53)) for patients with rheumatoid arthritis
NP Primary Care	McMenamin (2023)	[47]	Adaptation to health conditions was assessed in two studies with equal to improved abilities of patients t manage their health conditions in 2/2 studies.
APN Primary Care	Donald (2013)	[44]	Adaptation-related goals (1 study) Differed significantly for the nursing home subgroup in favour of the NP group, (P <0.05) Overall mean goal attainment scores did not differ between groups (1/1) Advance directive measures: no significant differences between the groups (1/1)

NP Primary Care	Fung (2014)	[45]	Disempowerment in treatment: significant reduction (p< 0.001). Disempowerment in the early (M1 = 13.073, SD = 7.73) and later in treatment (M2 = 9.14, SD = 6.04): significant reduction (p value not reported). Life review therapy conducted by psychiatric APNs could lead to a decrease in negative themes and might be an effective therapy for the homebound elderly with depression. A significant decline in depression among the treatment group was noted as measured by both the Hamilton Depression Scale (decrease of 6.3, 95% confidence interval (CI) = 4.2–8.3] and the Geriatric Depression Scale (a decline of 4.4, 95% CI = 2.5–6.4). Uncertainty in the ambiguity: When comparing the depressed women with cancer in high distress (n=32) with those in the placebo group, significantly less subscale of the Mishel Uncertainty in Illness Scale (P = 0.0181) (1/1) Distress: When comparing depressed women with cancer in high distress with those in the placebo group, less symptom in the Symptom Distress Scale (P < 0.0001) (1/1) Distress in adolescents at baseline and at 6, 12, 18, 24, 30, and 36 months post-intervention. At the first two time points (N = 1030), a significant effect was seen (P = 0.0001). Taking the first four time points into consideration (N = 810; 21% attrition), a significant intervention effect was noted from the SCSI (P = 0.006). When all six time points were used (N = 669; 17% attrition), a marginally significant intervention effect was seen (P = 0.06). Adolescents experienced less mental distress in the first 2 years, but this difference dissipated by 30 and 36 months.
NP Primary Care	Garner (2017)	[46]	Arthritis self-efficacy and empowerment: 2 studies. In the AMBRA study, nurse-led care was found to be superior to rheumatologist-led care regarding self-efficacy at 12 months, but this was not statistically significant at 24 months (1/1). Arvidsson, et al's phenomenographic study noted that nurse-led care patients were empowered to solve their own problems (1/1).
NP Primary Care	McParland (2022)	[35]	Caregiver Burden (3 studies): No significant differences in caregiver strain or burden in 3/3 studies
NP Primary Care	Morilla-Herrera (2016)	[36]	Care planning 1/1 study showed higher perceived quality of care planning (p = 0.005). Caregiver Distress ½ studies found positive results for mental health [IG: 19(13.48) vs. CG(28 (21.54); P: 0.05]; ½ studies showed significant improvements in distress as measured by the caregiver NPI at 12 months (no p-values reported)
			**Clinical/Health (12 reviews)**
APN Primary Care	Chan (2018)	[42]	Condition related clinical indicators were reported in nine studies with equal to statistically significant improvements noted in all studies. The studies examined health status, compliance of clinical practice guidelines, asthma monitoring with lung function test outcomes (FVC1 and PEF); type 2 diabetes mellitus: HbA1c, blood pressure and lipid profile, chronic inflammatory arthritis, post-surgery for colorectal cancer, HIV and mental illness, chronic depression, blood pressure control, lipid monitoring, women with gestational diabetes, and people with chronic kidney disease.
APN Acute AND Primary	Gielen (2014)	[48]	Most of the 13 studies reporting on clinical outcomes found no differences between nurse prescribing and physician prescribing in this regard (see Table 3).
NP Primary Care	Mileski (2020)	[49]	NPs improved health outcomes in 19.85% of theme occurences
APN Primary Care	Newhouse/ Stanik-Hutt (2013)	[39-40]	Self-reported perceived health (5 studies): 1/5studies reported findings in favour of the NP group; 4/5 studies reported no differences between groups (no p-values reported).
NP Primary Care	Smigorowsky (2020)	[50]	Effect of NP-led care on SF 36 physical composite score (2 studies): Mean difference ([MD] = 0.17, 95% CI: −0.89, 1.23; Z = 0.32), p = .75, inference no significant difference. I2 = 0% indicating low risk for heterogeneity
NP Primary Care	Sun (2022)	[38]	Health Risk reduction for community dwelling adults with intellectual and developmental disability (1 study): Health risk reduction (pre vs post 4.7 vs 3.5, p <.05)

NP Primary Care	Swan (2015)	[51]	Physiologic measures (3 studies): Between-group differences were generally not significant, with the exception of the cholesterol/ high-density lipoprotein (HDL) ratio and the diastolic blood pressure at 6 months with both favoring the APN group. Additional physiologic measures were investigated in single studies with no differences between APN and physician groups for mortality, change in body mass index (BMI), change in LDL or peak expiratory flow rate.
NP Primary Care	Tsiachristas (2015)	[52]	Clinical outcomes (5 studies): 5/5 studies reported improvements in clinical outcomes
NP Primary Care	Van Vliet (2020)	[53]	Clinical outcomes: Little is known about the effects of NPs in ambulance care on patient outcomes or care provider outcomes
NP Primary Care	Yang (2021)	[54]	Health outcomes: 5/5 studies reported positive health outcomes and significant improvements in health status
CNS Acute	Kilpatrick (2014)*	[55]	Patient outcomes for CNSs in ALTERNATIVE Provider Roles were examined in three studies with equal to statistically significant improvements in care outcomes for patients with complex type 1 or 2 diabetes, men with prostate or bladder cancer receiving radical radiotherapy, patients with rheumatoid arthritis. Patient outcomes for CNSs in Complementary provider outpatient roles were assessed in seven studies with equal to statistically significant improvements in 7/7 studies for adults with first-episode psychiatric symptoms, depressed veterans, adults with heart failure, women with newly diagnosed with breast cancer, patients with rheumatoid arthritis, low-income, black, single pregnant women at high risk of delivering low birth weight infants to their usual prenatal and post-natal care, and residents in nursing homes significantly fewer CNS patients received home help than inpatient controls.
APN Acute	Kilpatrick (2015)-Inpatients*	[56]	CNS group: Patient outcomes: no significant differences noted related to charted nursing observations per sitter day in the CNS group<br>NP group: Mortality, neonatal complications, overall long-term delays: No significant differences noted
			**Clinical/Cardiovascular (18 reviews)**
APN Acute	Audet (2021)	[7]	Symptoms inventory: Statistically significant reductions in symptoms at 2 weeks in the intervention group in one study and no difference at 6 weeks post discharge from hospital found in 2/2 studies.
NP Primary Care	Fichadiya (2021)	[57]	Nurse practitioners were responsible for the assessment and monitoring of HF patients' symptoms and their medication management either through NP led telehealth or NP led HF outpatient clinic
APN Acute	Manoj (2019)	[63]	Direct current cardioversion (DCCV): Successful cardioversion rates in restoration to sinus rhythm in atrial fibrillation following DCCV was measured in seven studies. (See Figure 2). No significant difference in success rate or complications was found between APN and Cardiologist performed DCCV procedures. Norton et al. (2016) outcome of this study provided an overall success rate of 93.4% with NP alone (92.7%) and the MD group (93.9%) with no major difference among the groups.

APN Primary Care	Massimi (2017)	[64]	Meta-analyses on systolic (SBP) and diastolic (DBP) blood pressure reduction (10 studies 3,881 patients) and HbA1c reduction (7 studies 2,669 patients) were carried-out. The pooled MD were: SBP -3.04 (95% CI -5.01Ð-1.06), DBP -1.42 (95% CI -1.42Ð-0.49) and HbA1c -0.15 (95% CI -0.32±0.01) in favor of the experimental groups.<br>A meta-analysis on SBP reduction was carried out on 10 studies [24, 39, 44, 48, 50±55], involving a total of 3,881 patients. The pooled MD was -3.04 (95% CI -5.01 to -1.06) in favour of the interventions, with significant heterogeneity between studies (I2 = 55%, p = 0.02) (Fig 2).<br><br>Meta-analyses of subgroups showed a statistically significant effect if the interventions were delivered to diabetic patients (MD -2.56, 95% CI -4.82 to -0.31), if an APN was employed (MD -3.57, 95% CI -6.36 to -0.78), if the nurses were specially trained (MD -2.81, 95% CI - 4.30 to - 1.32), if the studies had a sample size greater than 200 patients (MD -0.13, 95% CI -0.25 to -0.01) and if the allocation concealment was not clearly defined (MD -2.54, 95% CI -5.04 to -0.56). Stratification by type of intervention failed to show a significant effect of any specific intervention. Neither length of intervention nor attrition rate influenced the results, which remained significant in favour of intervention (Table 4). The same 12 studies [24, 25, 39, 43, 44, 48, 50±55] explored the effect on diastolic blood pressure (DBP) levels in a total of 5,671 patients with strong evidence (Table 3). Ten studies with 3,881 patients in total were included in the meta-analysis on the reduction in DBP [24, 39, 44, 48, 50±55]. A statistically significant reduction in DBP was found for the whole group (MD -1.42, 95% CI -1.42 to -0.49) with no statistically significant heterogeneity between studies (I2 = 34%, p = 0.14) (Fig 2). An attempt was made to identify possible influencing factors using stratified meta-analyses.<br><br>A statistically significant effect was shown for interventions on patients with CVD (MD -2.09, 95% CI -4.11 to -0.07), specific training of nurses (MD -1.56, 95% CI 2.63±0.48), face-to-face interventions (MD -2.41, 95% -3.54 to -1.28), attrition rate lower than 20% (MD -1.68, 95% CI -2.93 to -0.43) and unclear presence of allocation concealment (-1.71, 95% CI -2.86 to -0.56).<br><br>Stratification by type of nurse employed, by sample size and by duration of intervention did not influence the results, which remained significant in all subgroups (Table 4).
APN Acute	McCrory (2018)	[58]	Systolic blood pressure: In favour of the ANP group (MD: 3.13, 95% CI 1.83, 4.43, p ¼ 0.00001). However, the I2 was 56%.<br>Diastolic blood pressure: statistically significant difference in systolic BP reduction between the groups, in favour of the ANP group (MD: 2.53, 95% CI 1.78, 3.27; p ¼ 0.00001). However, the I2 was 79%.
NP Education	McQuilkin (2020)	[59]	favor of the experimental groups.
APN Acute	Monterosso (2019)	[60]	A meta-analysis on SBP reduction was carried out on 10 studies, involving a total of 3,881 patients. The pooled MD was -3.04 (95% CI -5.01 to -1.06) in favour of the interventions, with significant heterogeneity between studies (I2 = 55%, p = 0.02) (Fig 2).
NP Primary Care	McMenamin (2023)	[47]	None of the studies found any association between NP care and changes to cholesterol levels, body mass index
NP Primary Care	Carranza (2021)	[61]	Overall improvement in metabolic outcomes, but no p-values reported. NP vs. MD, within-group decrease (between- group difference): -HA1C: 2.5% vs 0.2% (2.3%) -BP: No difference within and between groups -Weight loss: 8.3 lbs. vs. 7.4 lbs. (0.9 lbs.) - Glucose: 83.7 mg/dl vs. 27.4 mg/dl (56.3 mg/dl)
NP Primary Care	HQO (2013)	[62]	Blood pressure: Model 1: 139 mmHg vs 139 mm Hg (p= 0.82) (1/1) Model 2: effect not assessed (4/4)<br>Clinical examination: Model 2: Patients in the specialized nursing group received significantly more measures of blood pressure (P < 0.001) among CAD patients (3/3). Clinical examination: Model 2: Among CAD patients (3/3), no significant difference between groups in the proportion of individuals with cholesterol measured (P = 0.48).

NP Primary Care	Lovink (2017)	[34]	Composite score for heart failure (1 study) intervention improved the composite endpoint of heart failure patients from -37 to 25 (P = 0.01). Ejection Fraction (EF) (1 study) difference in favour of the intervention: 33 patients compared with 45 in the control group (P = 0.03) had an EF <40%. (1/1) N-terminal pro brain natriuretic peptide (NTproBNP) levels (1 study) change in NT-proBNP level before and after the intervention was significant in the intervention group; it decreased from 1091 to 895 ng/L (P = 0.01). There was no significant before/after difference in the control group [588 vs. 671 ng/L (P = 0.5)].
NP Primary Care	Martinez-Gonzalez (2014)	[65]	Meta-analysis: Blood Pressure (5 RCTs) Compared to physician-led care, the pooled weighted mean differences (WMD) revealed a significant SBP-reducing effect of nurse led care interventions (SBP, mmHg: WMD -4.27, 95%CI -6.31 to -2.23; p <0.0001). The pooled weighted mean differences (WMD) also favoured a DBP-reducing effect of nurse-led care interventions but the confidence intervals crossed the line of no effect (DBP, mmHg: WMD -1.48, 95%CI -3.05 to -0.09; p = 0.06). There was no significant heterogeneity between trials (SBP: I2= 0%, p= 0.53; DBP: I2 = 38%, p = 0.19). Meta-analysis Nurse-led care and physician-led care in reducing the mean levels of total cholesterol (TC) at follow up (4 studies) No significant heterogeneity between trials (TC, mmol/l: weighted mean differences (WMD -0.08, 95%CI -0.22 to 0.07, p = 0.29; I2 = 0%). Individual trial estimates showed significantly more patients with nurse-led care had a positive decrease or regression in TC and low density lipoprotein (LDL) levels than did patients in the group of physicians [22]. 3/5 trial estimates showed no significant differences between groups in the reduction of LDL, high density lipoprotein (HDL), TC/HDL ratio or triglycerides. Meta-analysis Cardiac function (1 study) Compared to physician-led care, there were significantly more patients with nurse-led care who had a decrease or regression in the levels of functional exercise capacity (p = 0.001), N-terminal pro-brain natriuretic peptide (p= 0.004) or in the left ventricular end-diastolic volume index (p = 0.040). There were no significant differences between groups in the levels of C-reactive protein, left atrial size index, and left ventricular mass index or in the ratio of early to late mitral valve flow velocity.
APN Primary Care	Newhouse/ Stanik-Hutt (2013)	[39-40]	Lipid control. Three studies; 3/3 studies reported findings favouring the NP group (no p-values reported).<br>Blood pressure. (4 studies); 2/4 reported findings on blood pressure control favouring the NP group and 2/4 no difference between groups (no p-values reported
NP Primary Care	Norful (2019)	[66]	Hyperlipidemia: significantly more patients monitored in the co-management group (p = .007). Blood pressure monitoring: no sig differences in the number of patients monitored (p = .63). High-density lipoproteins: beneficial increase of HDLin 1/1 (p = 0.02)
NP Primary Care	Schadewaldt (2011)	[67]	Blood pressure 1 study Blood pressure reduction is possible for the short term (3 months). Effect not maintained 12 weeks after intense nurse-led support stopped. Random effect meta-analysis not sig at 6 to 8 months from the patient's attendance to a nurse-led clinic (P = 0.26 systolic blood pressure and P = 0.25 diastolic blood pressure. Significant heterogeneity (P = 0.002, I2 = 90% for systolic blood pressure and P = 0.00001, I2 = 96% for diastolic blood pressure). Long-term blood pressure outcomes (up to 1 year) (4 studies) Three studies found significant improvements in blood pressure less than 140/85 mmHg after 1 year participating in a disease management program (P < 0.001). and target 160/95, P < 0.001). Blood lipid outcomes Reduction but no sig difference between groups for total cholesterol (TC) and high-density lipoprotein (HDL) at 6, 8, 12 and 24 months. When examining the target value of less than 5 mmol/L: patients benefited from the nurse-led clinics after 1 year (P < 0.00001) 2/2 studies HDL: no sig difference in the meta-analysis in 2/2 studies at 3, 6, 12, and 24 months LDL-C and Triglyceride: sig results at 3 and 6 months in 2/3 studies (p = 0.001, p = 0.008) and no sig results at 12 and 18 months in 3/3 studies
NP Primary Care	Scott (2011)	[68]	Estimated incidence rates of previously undetected hypertension ranged from 14.3% to 63.1% no sig. specified. (4 studies)
NP Primary Care	Smigorowsky (2020)	[50]	Effect of NP-led care on vascular risk reduction (1 study) The NP-led group had a higher Framingham risk score at baseline. Therefore, the baseline Framingham risk score was adjusted, to produce a relative change of −12% (−22% to −3%) versus usual care −8% (−18% to 2%). Patients in the NP-led group had a 12% decrease in risk of developing coronary heart disease over the next 10 years. Secondary endpoints were absolute changes in the levels of risk factors. In the NP-led group 18.4% of patients reached low-density lipoprotein cholesterol (LDL) targets and 19% stopped smoking.

NP Primary Care	Martin-Misener (2015)*	[69]	Hypertension (1 study) the drop in diastolic blood pressure at 6 months was larger in the nurse practitioner group (356 patients) (mean difference: −3.0 mm Hg (95% CI −5.54 to −0.46); p=0.04).
			**Clinical/Cancer Care (4 reviews)**
APN Acute	Bryant Lukosius (2015)	[70]	Cancer screening was examined in 6 studies. Statistically significant results noted for screening in the intervention group with similar results between groups for technical competence between NPs and physicians.<br>Diagnosis:<br>NPs were significantly more likely to take more than one biopsy compared to the other healthcare providers (p<0.01). significant difference between gynecologists and NPs with respect to agreement between coloscopic and cone biopsy (p=0.2867). NPs had significantly fewer discrepancies between the Pap smear test and the biopsy (5.9% versus 12.3%, p=0.002), performed more endocervical curettages (98.1% versus 77.1%, p<0.001), had fewer patients lost to follow up significant differences in the rates of insufficient biopsies/endocervical curettages, unsatisfactory colposcopy or missed invasive cancers.<br>Treatment:<br>APN roles in cancer control are associated with no differences or improved outcomes. Across 29 studies reported in 31 papers, a total of 150 outcomes were measured. Of these outcomes, 88 were assessed as not different, 53 were found to be superior in the APN group, and nine were found to be superior in the control group (See Table on page 58 in report). In breast cancer care, statistically significant reductions noted levels of uncertainty at one, three and six months but not at 12 months in 1/1 study. Women with high distress scores in the intervention group who received CNS plus psychiatric consultation-liaison nurse care had less uncertainty (MUIS) (p=0.0181), less symptom distress (SDS) (p<0.0001), and better HRQL (SF-12) related to mental (p=0.0001) and physical (p<0.001) well-being compared with the total attention control group. Women with high distress scores in the intervention group who received CNS plus psychiatric consultation-liaison nurse care had less uncertainty (MUIS) (p=0.0181), less symptom distress (SDS) (p<0.0001), and better HRQL (SF-12) related to mental (p=0.0001) and physical (p<0.001) well-being compared with the total attention control group.

CNS Acute	Cook (2017)	[71]	Tailored care: specialist nurses play a role in understanding and meeting the individual needs of women with gynecological cancer.
NP Primary Care	Loescher (2018)	[72]	Clinical examination Six studies had some description of NPs' performance of clinical skin examination. In one study, NP-led skin cancer clinic over a 4- year period provided approximately a 14% increase in clinical skin examination. Over the four years, the authors found that the number of patients reviewed by the dermatologist dropped from 53% to 31%, and the number of patients requiring biopsy or surgery decreased from 22% to 6.5%.
NP Primary Care	Smith (2014)	[73]	Post-treatment survivorship care. No study of post-treatment survivorship care identified.
			**Clinical/Diabetes (11 reviews)**
APN Primary Care	Massimi (2017)	[64]	HbA1c: 29 studies investigated HbA1c levels as a primary outcome in diabetic patients, resulting in strong evidence of the efficacy of intervention. These studies included 4,207 patients. The levels of HbA1c were significantly lower in the experimental groups than in the control groups in four studies (Table 3). The two studies with statistically significant results and high methodological quality
APN Acute	McCrory (2018)	[58]	HBA1C: No difference between both groups statistically.
APN Primary Care	Wang (2019)	[74]	Effect of nurse-led prescription on HbA1c control Nurse-led prescription was prescribed by nurse independently and compared with that of physicians in 2 RCTs. There was no significant difference between nurse-led prescription and physician prescription in controlling HbA1c levels (SMD=0.203; 95% CI: 0.434, 0.029; P=.086; I 2=48.1%) (Fig. 2D)
NP Primary Care	McMenamin (2023)	[47]	Diabetes measures (i.e., HbA1c and blood glucose) were examined in two studies with equal to statistically significant improvements noted in the intervention group. The RCT and a quasi-experimental study found that NP care was associated with modest improvements in diabetes measures (i.e., HbA1c and blood glucose) and blood pressure compared with physician-only teams or pre-intervention measures. However, the RCT found that NP care was associated with no effect on these outcomes compared with usual care.
NP Primary Care	Carranza (2021)	[61]	Metabolic outcomes: Overall improvement in metabolic outcomes, but no p-values reported. NP vs. MD, within-group decrease (between- group difference): -HA1C: 2.5% vs 0.2% (2.3%) -BP: No difference within and between groups -Weight loss: 8.3 lbs. vs. 7.4 lbs. (0.9 lbs.) - Glucose: 83.7 mg/dl vs. 27.4 mg/dl (56.3 mg/dl)
NP Primary Care	HQO (2013)	[62]	HbA1C: Model 1: mean 9.72% intervention vs 9.84%: no difference (p = 0.82) (1/1) Model 2: significant decrease after 12 months favouring specialized nurse-MD team (p value not reported) (1/2); non-sig difference in HbA1C (MD, -0.12; 95% CI -0.44 to 0.20) (2/2) Process indicator: HbA1C Model 1: (p <0.05) Model 2: (no data) Model 1: Process indicator: no sig difference in patient history, weight, blood pressure, foot health, blood glucose level, creatinine level, and referrals to ophthalmologist. Model 2: (no data) Clinical examination: Model 2: Patients in the specialized nursing group received significantly more foot exams (p<0.05) among CAD patients (3/3). Clinical examination: Model 2: Patients in the specialized nursing group received significantly more retinal exam >24 months (p = 0.01) among CAD patients (3/3).
NP Primary Care	Lovink (2017)	[34]	Mean HbA1c of patients with diabetes: no significant difference in 1/1 study Two or more HbA1C tests: no significant differences in 1/1 study
NP Primary Care	Martinez-Gonzalez (2014)	[65]	Meta-analysis Glycosylated Haemoglobin Concentration (4 studies) No significant differences between nurse-led care and physician-led care in reducing glycosylated haemoglobin concentrations (HbA1c) at follow up. No significant heterogeneity between trials (HbA1c, %: weighted mean difference (WMD) 0.12, 95%CI -0.13 to 0.37, p =0.33; I2 = 0%). One trial/4 estimates showed no significant differences in the number of patients with a positive decrease or regression in blood glucose levels.
APN Primary Care	Newhouse/ Stanik-Hutt (2013)	[39-40]	Blood glucose control: 4 studies; ¼ studies reported findings favouring the NP group; ¾ reported no difference between groups (no p-values reported)

NP Primary Care	Norful (2019)	[66]	Annual eye exams: no sig difference in annual exams by ophthalmologists Diabetic control: sig more in the co-management group (<.001) HbA1C: 1/2 sig findings (p=0.02); ½ non sig findings
NP Primary Care	Sun (2022)	[38]	HbA1C (1 study): Greater decrease in A1C at 3 months (intervention vs control 1.7% vs. 0.7%, p <.001), 6 months (1.7% vs 0.8%, p <.001) in 1/1 study.
			**Clinical/Mental Health (18 reviews)**
NP Acute	Alotaibi (2020)	[75]	Greater reduction in anxiety in one study, no reduction of anxiety of symptom burden in another
APN Acute	Audet (2021)	[7]	Symptoms of depression equal to statistically significant reductions in depressive symptoms for patients following cardiac surgery
CNS Acute	Belun- Vieira (2016)	[76]	Anxiety and Depression was examined in five studies with non significant findings in 5/5 studies. A non-significant trend towards increased anxiety and depression noted at 12 and 24 months in the intervention group.<br>Emotional Wellbeing was reported in six studies and examined using role functioning, social functioning and emotional functioning. No differences noted in role functioning at 6 and 12 months, and social functioning at 12 months, statistically significant improvements favouring the intervention group noted in emotional functioning at 3 months, 6 months, and 12 months.
APN Primary Care	Cheng (2018)	[77]	The combined results implied that the nurse led management strategy had no impact on the cognitive (SMD= 0.07, 95% CI=0.28 to 0.42; P=.700), emotional (SMD= 0.15, 95% CI=0.09 to 0.39; P=.217), role (SMD=0.03, 95% CI=0.20 to 0.26; P=.797), social (SMD=0.16, 95% CI= 0.19 to 0.52; P=.360), and physical (SMD=0.12, 95% CI= 0.26 to 0.02; P=.086) functions compared with the control.
APN Acute	Kobleder (2017)	[78]	Psychological morbidity had similar scores for APN-based telephone follow-up in women with endometrial cancer in 1/1 study
APN Acute	Monterosso (2019)	[60]	Distress scores assessed in 4/4 studies with equal to statistically significant improvements noted . One study demonstrated significantly higher depressive symptoms in patients who received the nurse-led intervention during the intervention (6 months) Cognitive functioning was significantly higher by 4.04 units on average (MD = 4.04, 95%CI [0.59, 7.50], 463 participants, p = 0.02; I2= 24%; Fig. 3) and social functioning was significantly higher by 3.06 units on average (MD = 3.06, 95%CI [0.14, 5.97], 463 participants, p = 0.04; I2 = 0%; Fig. 4).
CNS Acute	Scheydt (2021)	[79]	A total of 46 tasks and activities of the Psychiatric Mental Health APN could be extracted (referred to below as practice dimensions). These practice dimensions can in turn be structured in six categories (referred to below as practice domains): (1)Direct (clinical) nursing and care practice (including «Relationship building and partnership», «Assessment and diagnostics», «Treatment planning and documentation» as well as «Treatment, therapy and disease management»); (2) Care coordination and case management; (3) Psychosocial health promotion and prevention; (4) Consulting, education and coaching (subdivided in «Education, counseling and coaching of patients, their relatives and families» as well as «education, consulting and coaching of decision-making organs and employees»); (5) Leadership and Public Relations; and (6) Research and practice development
APN Acute	Schneider (2021)	[80]	Mental health (e.g., anxiety, mood) reported in two studies with trends towards improved to statistically significant reductions in symptoms noted in 2/2 studies
NP Primary Care	McMenamin (2023)	[47]	Depressive symptoms no association between NP care and symptom changes or anxiety (Talley et al., 2021).<br>Mental health was examined in 2 studies in relation to patient confidence, empowerment with trends towards improvement to statistically significant improvements noted in 2/2 studies.
NP Primary Care	Fung (2014)	[45]	Depression symptoms: Reduced in primary care and home care (3/4) Psychological stress: Significant reduction for individuals (women, students, HIV) 5/5 Efficacy self-esteem with women with depression: Increase in the mean efficacy self-esteem score post-intervention and a significant difference between pre- and post-intervention BDI scores (t = 8.765, d.f. = 29, P = 0.0005) in a paired samples t-test. (1/1) Uncertainty for depressed women with cancer including those in high distress: significantly less than those in the placebo group at 6 months after surgery. (1/1 study)

NP Primary Care	McParland (2022)	[35]	Mental health (2 studies): Reductions in cognitive impairment [Pfeiffer Score] over 12 months with telephone follow-up in 1/1 study (p value not reported) Reductions in severe depression at 12 months [Geriatric Depression Scale] and loneliness [UCLA Loneliness Scale] in 1/1 study Significant improvement in cognitive impairment [Orientation Memory Cognition Tool] in 1/1 study
NP Primary Care	Morilla-Herrera (2016)	[36]	Depression 1/1 study showed no significant group differences in depression
NP Primary Care	Schadewaldt (2011)	[67]	Depression and anxiety No sig differences in 1/1 study at 1- and 4-years. Suspected cross-over effect for patients in the control group at 4 years with decreased odds for depression for clinic attendees (P = 0.001).
NP Primary Care	Smigorowsky (2020)	[50]	Effect of NP-led care on SF 36 mental composite score (2 studies) Mean difference for SF36 mental composite score (mean difference [MD] = −1.11, 95% CI: −4.19, 1.98; Z = 0.70, p = .48; suggests no statistical difference. I2 Statistic is 80%, therefore, there is a high risk of heterogeneity. These results must be interpreted with care because there is considerable variation in the combined or pooled results
NP Primary Care	Swan (2015)	[51]	Subjective health status (e.g., SF-36, burden of illness): with no differences between groups
NP Primary Care	Turi (2023)	[81]	Depressive Symptoms and Self-Management: Eight studies evaluated depressive symptoms as outcomes of NP care for depression. Two studies found that when PMHNPs provided measurement-based care, depression symptoms decreased at or near statistical significance (p = 0.001 and 0.08. PMHNP-delivered cognitive behavioral and problem-solving therapy was associated with no significant difference in Hamilton Rating Depression Scale scores compared to usual care (F-statistic = 1.07, p = 0.3;). PMHNP-delivered motivational interviewing was associated with a significant increase in patient depression self- management (pretest = 49.23, SD = 10.71; posttest = 70.65, SD = 13.74; t = -8.84; p <0.001). 1/1 study<br>SUD Symptoms (2 studies) PMHNP delivered measurement-based care was not associated with changes in Alcohol Use Disorders Identification Test scores (3.17 to 2.79, p = 0.211). t NP-led collaborative care was associated with decreased illicit drug use symptoms among patients who were actively engaged and those that declined services in one clinic (p = 0.029, p = 0.046) (1/1 study)<br>Anxiety Symptoms (4 studies) 2/2 studies found that PMHNP delivered measurement-based care was associated with decreases in Generalized Anxiety Disorder (GAD-7) scores that achieved or neared statistical significance (p = 0.001 and 0.08) 2/2 studies found that NP-led collaborative care was associated with decreased GAD-7-scores (p < 0.001 and p = 0.028, GAD-7 scores dropped from 10.0 to 8.1, no p-value reported, 47% of patients had a drop below 5 or 50% of original score) in 1/1 study
NP Primary Care	Donald (2015)* (transition)	[33]	Anxiety (STAI) Rehabilitation (mean difference: -15.7(95%CI: -20.73 to -10.67) <0.0001
APN Acute	Bryant-Lukosius (2015)	[70]	Women with high distress scores in the intervention group who received CNS plus psychiatric consultation-liaison nurse care had less uncertainty (MUIS) (p=0.0181), less symptom distress (SDS) (p<0.0001).
			**Clinical/Musculoskeletal (6 reviews)**
APN Acute	Allsop (2021)	[10]	Refracture: Rates unchanged in 2/3 studies. Non significant reduction at 3 months in 1/3 studies
CNS Primary	Lempp (2020)	[82]	Disease activity was assessed in six studies including 4 RCTs with no differences noted between the groups in 6/6 studies
APN Acute	De Thurah (2017)	[43]	Disease activity for rheumatoid arthritis was assessed in four studies with no differences noted between the groups after 1-year follow-up (MD −0.07 (95% CI −0.23 to 0.09)), trends toward improvements noted in the intervention group at the 2-year follow up.
NP Primary Care	Carranza (2021)	[61]	Disease progression: 1/1 no sig difference between NP and physician groups Disease severity: 1/1 no sig difference between NP and physician groups Disease progression: Sig reduced with NP group (no p-values reported) (1/1)

NP Primary Care	Garner (2017)	[46]	Disease activity in rheumatoid arthritis assessed in 5/5 studies. Results include equal to superior care. Significant reduction (p < 0.05). Morning stiffness assessed in 2 studies with significant increase in ½ studies, rheumatologist-led care change 6.67 vs nurse-Led care – 5.98, p= 0.01)
CNS Acute	Bryant-Lukosius (2015)*	[76]	Significantly fewer caregiver emotional and depression symptoms between 2 and 4 weeks post-discharge in the intervention group and fewer infections in surgical patients at 2 weeks post-discharge in the usual care group noted in studies of frail elderly.
			**Clinical/Renal (3 reviews)**
NP Primary Care	HQO (2013)	[62]	Process indicator: urinalysis Model 1: (p < 0.01) Model 2: (no data)
NP Primary Care	Martinez-Gonzalez (2014)	[65]	Meta-analysis Kidney Function (3 studies) No significant differences between groups in the parameters of kidney function including the levels of urine sodium excretion and serum creatinine at six months. The reported median (IQR) levels of urinary albumin excretion tested to detect renal complications were higher in the nurse-led care group [UAER, mmol/day: nurse-led care, median 39.2 (IQR 16.0 to 200.0) vs. physician-led care, median 30.5 (IQR 14.5 to 147.2)].
APN Acute	McCrory (2018)	[58]	In favour of the ANP group (MD: 13.93, 95% CI 8.82, 19.04; p¼ 0.00001). However, the I2was 42%,
			**Clinical/Respiratory (8 reviews)**
NP Primary Care	Baker (2017)	[41]	Respiratory Exacerbation frequency was examined in two studies. No significant between group differences in frequency of exacerbation were reported in any of these studies
APN Primary Care	Lawton (2018)	[84]	Respiratory exacerbation requiring treatment with antibiotics: no significant differences between nurse-led and doctor-led care in terms of lung function, infective flareups (exacerbations) in 1/1 study.
NP Primary Care	HQO (2013)	[62]	Disease specific measure: peak flow Model 1: no difference (p = 0.82) (1/1) Model 2: (no data)
NP Primary Care	Kuethe (2013)	[85]	Frequency of exacerbations: No sig difference between the groups in 4/4 studies rate ratio 1.23; 95% CI 0.91 to 1.66; P =0.368) No sig difference at 6-month follow up (OR 0.86; 95% CI 0.44 to 1.71;p = 0.674).<br>Absence from school/work due to asthma (3 studies): Median of 0 days school absence in nurse group 20.6% of patients (in an adult population) in the physician group had more than one day of absence in the physician group (p = 0,04). Forced expiratory volume in 1 second (FEV1) (3 studies). No difference found (MD -0.54% predicted; 95% CI -4.20 to 3.12). Sensitivity analysis using a fixed-effect model showed the same results (MD -0.54% predicted; 95% CI -4.20 to 3.12). Peak expiratory flow rate (PEF) (2 studies, a decrease of PEF over time). Mean drop was 2.53% (SD 11.5) in the physician-led group and 3.92% (SD 12.4) in the nurse-led group. No sig difference in the change in PEF between the two groups (P = 0.122) An increase over time (5.3% in the nurse-led group and 3.94% in the physician-led group) in 1/1 study, but no statistically significant between group differences were found (P = 0.66). Airway hyper-reactivity (including PD/PC20 methacholine/histamine) (2 studies: No statistically sig differences between groups
NP Primary Care	Martinez-Gonzalez (2014)	[65]	Meta-analysis Lung Function (3 studies): Individual trial estimates showed no significant differences between groups in various parameters of lung function including measurements of peak flow at six months, and PD20, lung function (%FEV1) or FENO either at 12 or 24 months.
APN Primary Care	Newhouse/ Stanik-Hutt (2013)	[39-40]	Peak flow (1 study). No difference between groups, no p-value reported.

NP Primary Care	Sun (2022)	[38]	Asthma control in children (1 study) Children with asthma had fewer nighttime symptoms (t test 3.966 df 19 p <.05), fewer course of oral steroids (t test 3.750 df 18, p <.05), better adherence to therapy (z score -3.272, p < 05), level of control (z score -4.132, p <.001). School attendance, asthma control in children (1 study) A trend towards 4 fewer missed school days was experienced but not significant (t test 1.928 df 15 p >.05).
NP Primary Care	Donald (2015)* (transition)	[33]	Experience of asthma exacerbation: (relative risk (RR): 0.93, 95%CI: 0.65–1.33, p = 0.67) Change in maximal peak flow in asthma (mean difference (MD): 1.39, 95%CI: 6.63 to 3.85, p = 0.12), Emergency nebulisations (RR: 1.60, 95%CI: 0.79–3.24, p = 0.19)
			**Diagnosis (3 reviews)**
NP Primary care	Carranza (2021)	[61]	Diagnostic accuracy: Sig improved with NP group (1/1). Adenoma detection NP vs. MD: 42% vs. 17% (p = .0001)
NP Primary care	Elder (2015)	[86]	Nurse-initiated X-Rays reduced time to diagnosis and treatment (from 102.7 min to 65.5 min, P <0.001). 1/3
NP Primary care	Galiana-Camacho (2018)	[87]	Most common diagnoses: soft tissue (both trauma and loss of skin integrity) (around 35%), and bone fractures in second place (around 11%) (6/6)
			**Education-Patient (10 reviews)**
NP Acute AND Primary	Arian (2017)	[88]	Education-Patient: Patients and society are not always aware of independent nurse prescribing and the positive contributions.
NP Primary Care	Carranza (2021)	[61]	Patient education: 2/3 studies favour NP group (no p-values reported), 1/3 no sig differences with care provided by physicians
NP Primary Care	Galiana-Camacho (2018)	[87]	Health education: 98.6% of patients with pharmacological treatment at discharge received information on correct intake; 69.8% received health education; in 41.3% of cases they were given advice on their health problems in written form, and 90.1% received information on who to turn to if they needed help (1/1)
APN Acute AND Primary	Gielen (2014)	[48]	Provision of information: Nurses generally appear to give more or the same amount of information to patients.
NP Primary Care	HQO (2013)	[62]	Patient education Model 1: more likely in NP group (p < 0.001) Model 2: (no data)
APN Primary Care	Massimi (2017)	[64]	Educational interventions were based on face-to-face visits carried out with hospital [61] with telephone follow-up [60, 61].
NP Primary Care	Morilla-Herrera (2016)	[36]	Increased knowledge 1/1 study showed increased knowledge of services and who to contact at 3 and 12 months (p <0.03)
NP Primary Care	Norful (2019)	[66]	Patient education: more likely to be completed for dietary and activity recommendations that included sodium reduction (p <.001), moderation in alcohol consumption (p <.001), and weight control or reduction (p <.001).
NP Primary Care	Tsiachristas (2015)	[52]	Patient information (4 studies) was found to be higher in 3/4 studies.
NP Primary Care	Martin-Misener (2015)*	[69]	Patient education (1 study) Patients had been told the cause of their illness (relative risk (RR) 1.12; 95% CI 1.06 to 1.19; p=0.0001), how to relieve their symptoms (RR 1.27; 95% CI 1.19 to 1.34; p<0.00001) , and what to do if the problem persisted (RR 1.06; 95% CI 1.02 to 1.09; p=0.002)

			Mortality (21 reviews)
APN Acute	Allsop (2021)	[10]	No significant differences in 9/12 studies, significant reductions reported in 2/12 studies and 1/12 reported increased mortality at 30 days with no significant differences at 9 and 12 months
APN Acute	Audet (2021)	[7]	Mortality during hospitalization: No significant difference was identified between groups.
APN Acute	Edkins (2014)	[91]	No significant difference was found between the 2 groups for mortality, ventilator days, or final disposition.
APN Primary Care	Kennedy (2012)	[92]	Reduced mortality/morbidity found in 1 qualitative study found in 2 quantitative studies.
APN Primary Care	Lawton (2018)	[84]	No difference in mortality with one patient died in each group
APN Primary Care	Massimi (2017)	[64]	Total mortality: Two APN studies.<br>Total number of deaths in the experimental groups was lower than in the control groups, reaching statistical significance in 1/2 APN study ; these studies were based on interventions lasting 12 months or longer on patients with coronary heart disease or chronic heart failure led APNs.
APN Acute	Medeiros (2011)	[94]	No differences in mortality in 2/2 studies.
APN Acute	Ordonez-Piedra (2021)	[95]	Trend toward reductions to statistically significant reductions noted in 4/4 studies
APN Acute	Schneider (2021)	[80]	Survival time; reported in two studies with trend towards improved to statistically improved results in 2/2 studies
APN Acute	Woo (2017)	[96]	Mortality in the Emergency Department was not examined. Mortality in critical care was examined in five studies with equal to statistically significant reductions in the NP group for Intensive care unit mortality rates in 5/5 studies and significant reductions in hospital mortality noted in 1/1 study.
NP Primary care	Barker (2018)	[89]	Mortality (4 studies) 3/4: NS difference in mortality between the groups. 1/4: One of the control groups has a lower risk of mortality than the intervention group (p = 0.013).
NP Primary care	Carranza (2021)	[61]	Mortality: Sig reduced with NP group (1/1). NP vs. MD: 2 (0.3%) vs. 32 (1.5%); HR 0.2, 95% CI [0.04, 0.8]
NP Primary care	Driscoll (2015)	[90]	Deaths: p = 0.05 : results favour NP-led care (1 NP study)
NP Primary care	Garner (2017)	[46]	Mortality: no difference between nurse-led care and rheumatologist-led care in 3/3 studies
NP Primary care	Leduc (2021)	[93]	Mortality: no sig difference in 4/4 studies; most studies (3/4) trended toward a slight but insignificant increase in mortality in the intervention groups, with the exception of one intervention (1/4) that had a slight but insignificant decrease in mortality
NP Primary care	Lovink (2017)	[34]	Mortality in LTC: no sig difference in deaths in 1/1 study
NP Primary Care	McParland (2022)	[35]	Mortality at 90 days (1 study): Hospital case-managers did not detect any reduction in mortality at 90 days Mortality at 12 and 24 months (1 study) At 12 months, a community-based nurse case-manager intervention (n = 3432 participants) found significant differences in the proportion of deaths favouring the intervention group at 1 year. At 24 months, trend towards reduction at 2 years was not significant
NP Primary care	Morilla-Herrera (2016)	[36]	Mortality 3/3 studies reported as non significant results with a trend to increased rate of survival (no p value)

APN Primary Care	Newhouse/ Stanik-Hutt (2013)	[39-40]	Mortality (1 study). No difference between groups, no p-value reported.
NP Primary Care	Schadewaldt (2011)	[67]	Coronary events, coronary mortality and total mortality (1 study) Total mortality significantly reduced in the intervention group at the 4-year follow up (P = 0.038) and the occurrence of coronary events revealed a borderline difference (P = 0.052) even though some patients of the control group had, by that stage, attended nurse-led clinics. Coronary events, coronary mortality and total mortality (1 study) Total mortality: No significant differences occurred between the groups concerning all cause mortality, coronary events or deaths due to coronary events at 10-year follow up. However, because of the cross-over effect all patients had the chance to attend the nurse-led clinics within 10 years and detecting differences between the groups was unlikely.
NP Primary Care	Donald (2015)* (transition)	[33]	Mortality: no differences and outcomes favoured usual care
			**Morbidity (1 review)**
APN Acute	Allsop (2021)	[10]	Morbidity was reported in six studies. There is an overall reduction in the complication rate. One study reported statistically significant decrease (p <0.001) and one study reported a slight non-significant increase, with overall event remaining low.
			**Patient Adherence (7 reviews)**
APN Acute	Allsop (2021)	[10]	Bone mineral density (BMD) testing, vitamin D, calcium, osteoporosis medication treatment was examined in 5 studies. One study noted a statistically significant difference in Osteoporosis medication adherence p < 0.001) and BMD testing at 6 months (p < 0.001).
APN Acute	Audet (2021)	[7]	Adherence to cardiac rehabilitation: Equal to statistically significant improvements in the intervention group in 2/2 studies. Dietary management: Equal to statistically significant improvements in the intervention group in 2/2 studies. Smoking Cessation: patients of the intervention group were associated with higher effect of nicotine replacement therapy (p=0.003) and higher smoke-free time (p=0.038) six months after hospital discharge. Lipid management: intervention group were associated with lower serum total cholesterol (TC), lipoprotein cholesterol (LDL-C) and triglyceride (TG) levels than patients in the controlled group (p=0.0001) after one year. Patient adherence) Equal to statistically significant improvements in the intervention group in 2/2 studies
APN Primary Care	Van Camp (2013)	[97]	The primary outcome, medication adherence, was measured by either method adherence as the mean percentage of adherence, in the short and long term. The pooled mean differences were +5.39 (1.70–9.07) in the short term and +9.46 (4.68–14.30) in the long term, in favour of the intervention groups. The forest plots in Fig. 3a and b cluster the five studies expressing adherence dichotomously (proportion of adherent patients). The pooled odd's ratios were 1.55 (1.04–2.29) in the short term and 1.87 (1.35–2.61) in the long term.
APN Acute AND Primary	Gielen (2014)	[48]	Patient enablement: Two studies report that patient enablement, i.e. the extent to which patients understand their illness and are able to cope with it, is similar.
NP Primary Care	Kuethe (2013)	[85]	Compliance with medication: No data presented for patients
NP Primary Care	Swan (2015)	[51]	Follow-up adherence (two studies): APNs more frequently requested a return visit and their patients were more likely to keep the appointment.
NP Primary Care	Donald (2015)* (transition)	[33]	Attendance at cardiac rehabilitation: Estimates favoured nurse practitioner care

			Quality of Life (28 reviews)
APN Primary Care	Chan (2018)	[42]	HRQoL was reported in 17 studies included in the review with equal to statistically significant improvements noted in 16 studies. One study reported a small but statistically significant reduction in overall physical component scores on the SF36 for patients with Type 2 diabetes).
APN Acute	Allsop (2021)	[10]	QOL was reported in 3 studies. Significant improvements were reported in two studies including SF 36 QOL (p <0.001) and the subscale mental aspects of social functioning (p = 0.028), vitality (p = 0.004), physical aspects of bodily pain (F = 7.410, p = 0.009) and general health perceptions (F = 5.043, p = 0.029) of SF-36. One study reported no significant changes in general health status. There was an overall significant improvement at three months, with scores in the experimental group significantly higher (60.77 vs. 51.25 p <0.001) in one study.
NP Acute	Alotaibi (2020)	[75]	ANP interventions improved QoL of patients in palliative care
APN Acute	Audet (2021)	[7]	QoL: no significant difference was found between groups in 2/2 studies
NP Primary care	Baker (2017)	[41]	Health related quality of life (QoL) was reported in one study with statistically significant improvements noted in 1/1 study.
CNS Acute	Belun- Vieira (2016)	[76]	Quality of Life was reported in six studies: The meta-analysis of results at 3-month follow-up suggests a significantly increased quality of life score for patients receiving NFU with a mean difference of 2.88 (95% CI: 0.71–5.05, P≤0.01). However, there is a high level of heterogeneity recorded (X^2=6.79, df = 2, P=0.03 and I2 =71%)
APN Acute AND Primary	Bohner (2012)	[98]	Quality of life reported in 5 studies with improvements noted in the intervention group in 5/5 studies during the study period lasting up to 12 months. Long-term improvements noted up to 12 weeks in 1/5 studies.
APN Primary Care	Cheng (2018)	[77]	QLQ-C30[17] consists of 2-item global quality of life scale; 5 multiitem function scales, including cognitive, emotional, physical, role, and social; 6 single-item symptom scales for appetite loss, constipation, diarrhea, dyspnea, financial impact, and insomnia; and 3 multi-item symptom scales for fatigue, pain, nausea, and vomiting. A meta-analysis was conducted.<br>Global quality of life was evaluated in 6 studies. Obvious heterogeneity was found across these 6 trials (Q value=20.19, df=5, P=.001; I 2=75.2%, Tau=0.0908) and thus a random effects model was performed. The pooled results suggested that there was no difference in the global quality of life (SMD=0.17, 95% CI=0.12 to 0.45; P=.255) between patients undergoing nurse-led management and not.<br>Six studies assessed the function status of the cognitive and social, but 7 for emotional and role. Obvious heterogeneity existed across these 6 trials for the cognitive (Q value=28.46, df=5, P=.000; I 2=82.4%), emotional (Q value=21.99, df=6, P=.001; I 2=72.7%), role (Q value=19.28, df=6, P=.004; I 2= 68.9%), and social (Q value=28.87, df=5, P=.000;I 2=82.7%) functions and thus a random-effects model was used for them. In contrast, no heterogeneity was present for physical function (Q value=8.22, df=5, P=.144; I 2=39.2%), which led to a fixed effects model used.
APN Acute	Kobleder (2017)	[78]	Quality of life was examined in two studies with a trend towards improvement to statistically significant improvements noted in the intervention groups.
APN Primary Care	Lawton (2018)	[84]	Quality of Life- fewer symptoms and less impact on daily life with nurse-led care, but data show no clinical or statistically significant differences between nurse led and doctor-led care Exercise capacity- No significant differences in distance walked between nurse-led and doctor-led care FeV1/FVC- Nil significant differences in percentage predicted FEV1 between nurse-led and doctor-led care
APN Acute	Monterosso (2019)	[60]	Quality of Life: Three studies investigated QoL in terms of change over time and found no significant differences between the experimental and control groups.<br>Cancer survivorship QOL was measured in 2 studies. Statistically significant improvements noted in 2/2 studies. A significant benefit of the intervention for overall QoL, and psychological and social domains in 2/2 studies.

APN Acute	Ordonez-Piedra (2021)	[95]	Quality of life reported in 3 papers with equal to statistically significant improvements noted in all studies.
APN Acute	Schneider (2021)	[80]	improvement in the quality of life of cancer patients noted in four studies with a trend towards improved to statistically significant improvements in 4/4 studies
NP Primary care	Kwok (2022)	[99]	quality of life For the RCTs, there was no significant difference between the intervention and control groups for improving QOL (SMD 0.10; 95% CI − 0.13, 0.34; 4 studies)
NP Primary care	Carranza (2021)	[61]	Quality of life: 4/4 no sig difference between NP and physician groups
NP Primary care	Fung (2014)	[45]	QOL: When comparing depressed women with cancer in high distress with those in the placebo group, achieved better results in the Short- Form Health Survey-12 mental (P = 0.0001) and physical (P < 0.0001) QOL over time (1/1)
NP Primary Care	HQO (2013)	[62]	HRQOL: SF-36 score: Model 1: sig improvement from baseline for all participants; no sig difference between groups on the physical and mental components (1/1) Model 2: no difference in the mental component for 2/2 studies and sig deterioration in the physical component for specialized nursing care (MD –3.1; P = 0.04) (1/2). HRQOL diabetes: Model 2: sig. improvement in diabetes (MD, 5.42; 95% CI, 4.3–10.41) (1/1). No sig. differences for other elements indicating higher QOL. (1/1) HRQOL CAD and CHF: Model 2: improvement in exertional capacity (MD, 5.25; P = 0.001) and angina frequency (MD, 2.37; P = 0.04) among the nurse-led clinic group (2/2), significant decrease in worsening chest pain (OR, 0.59; 95% CI, 0.37–0.94; P = 0.02). No differences in angina stability, treatment satisfaction, QOL.
NP Primary Care	Kuethe (2013)	[85]	Quality of life (3/3 studies) QOL improved over time. No statistically significant difference between the nurse-led groups and the physician-led groups After meta-analysis of 2 studies using the same instrument, no effect was found (SMD -0.03; 95% CI -0.23 to 0.17; Figure 3). Sensitivity analysis using a fixed-effect model yielded the same results (SMD -0.03; 95% CI -0.23 to 0.17).
NP Primary care	Lovink (2017)	[34]	QOL (1 study) No sig difference between groups<br>Quality adjusted life-years (1 study) No sig difference between groups
NP Primary care	Morilla-Herrera (2016)	[36]	Quality of life 2/4 studies indicated significant improvements in QoL; 1/4 showed a non significant result with a trend to improved QoL; ¼ showed no significant differences between groups.
NP Primary care	Norful (2019)	[66]	QOL: sig improvements in 1/3 studies (p = 0.04); non sig changes in 2/3 studies
NP Primary Care	Osakwe (2020)	[37]	NP home visits and QOL: 1/1 study: At recovery, the total COPD specific questionnaire to assess QOL score decreased for patients who received NP-home visits, it did not reach significance (p = 0.06). NP home visits and QOL: improvement in the activity domain was significant (p< 0.05). 1/1 study
NP Primary Care	Schadewaldt (2011)	[67]	Quality of life and general health perception: Data pooled for 3 of the 4 studies: attending a nurse-led clinic is beneficial for patients in the following domains: physical functioning and physical role limitation with P-values of 0.01 and 0.05, respectively, emotional role limitation (P = 0.03) and general health perception (P = 0.02). No difference between the groups was seen in bodily pain and mental health. In the 4th study: improvements for physical functioning, mental health and general health perception, achieving the most significant results for energy and vitality (P = 0.0001) and social functioning (P = 0.0002). Across studies, no stat difference at 18 months and 4 years
NP Primary Care	Smigorowsky (2020)	[50]	HRQOL: Effect of NP-led care on length of stay after cardiac surgery (2 studies): The mean difference for length of stay indicates no significant difference between NP-led care and usual care on length of stay in postoperative cardiac surgery (mean difference [MD] = −0.89, 95% CI:−2.44, 0.66, Z = 1.13, p = .26,) I2 statistic is 0%, therefore low risk of heterogeneity
CNS Acute	Kilpatrick (2014)*	[55]	There were no significant differences between groups in QALYs.

NP Primary care	Tsiachristas (2015)	[52]	Quality of life (4 studies) improved in 2/4 studies and not significantly different in 2/4 studies.
APN Acute	Kilpatrick (2015)-Inpatients*	[56]	No significant differences noted in 2/2 studies related to NPs for congenital anomaly, apnea, feeding tolerance, respiratory distress syndrome, transport practice, 30-day post discharge mortality, overall adverse event rate, change in daily functioning, symptom severity, and change in health status and functional ability up to 6 weeks post discharge, patient perceived problems at 6-weeks post discharge.
NP Primary Care	Donald (2015)* (transition)	[33]	Change in asthma-related quality of life (Asthma Questionnaire-20; MD: 0.8, 95%CI: 2.22 to 0.62, p = 0.28) Quality of life-respiratory-related (St. George Respiratory Questionnaire; MD: 1.08, 95%CI: 4.93 to 7.09, p = 0.72).
			**Satisfaction-Patient and Family (40 reviews)**
APN Primary Care	Chan (2018)	[42]	Satisfaction was examined in five studies and equal to statistically significant results noted in all studies.
APN Acute	Audet (2021)	[7]	Satisfaction-Patient: Equal to statistically significant improvements in 4/4 studies
NP Primary Care	Baker (2017)	[41]	Patient Satisfaction was examined in 2 studies with no differences noted between groups
CNS Acute	Belun- Vieira (2016)	[76]	Patient Satisfaction at 3 and 12 months was examined in six studies. Significant increase in satisfaction with CNS care, though heterogeneity is high at 71%. Satisfaction with accessibility by phone was examined in two studies and no differences noted between the groups.
CNS Acute	Cook (2017)	[71]	Dependable expertise: women with gynecological cancer express trust and reassurance in the experience and expertise of the specialist nurse
NP Primary Care	Dawson (2015)	[101]	Three studies reporting on NP roles noted increased satisfaction with access to care, NP time spent with patients, care quality, and timeliness of service delivery.
APN Acute	De Thurah (2017)	[43]	Patient satisfaction was examined in four studies with trends toward improved to statistically significant improvements noted in the intervention group in 4/4 studies
APN Acute AND Primary	Gielen (2014)	[48]	Patients were generally more or equally satisfied with the care provided by nurses compared to the traditional care provided by physicians -Patients were generally more satisfied or equally satisfied
NP Acute	Johnson (2015)	[105]	Favorable patient satisfaction surveys were obtained from 90% of the patients. Eighty-six percent strongly agreed or agreed that the survivorship care plan met their needs, and 88% reported that they strongly agreed or agreed that they understood the treatment. Both groups included in the study were satisfied with the interpersonal aspects
APN Acute	Joseph (2015)	[106]	Patient satisfaction of endoscopy procedures performed by nurse endoscopists/nurse practitioners. Two studies demonstrated greater patient satisfaction in nurse-led colonoscopies over physician-led colonoscopies
APN Primary Care	Kennedy (2012)	[92]	Quality of patient experience: Favorable views by patients found in 5 qualitative studies and in 7 quantitative studies
APN Acute	Kobleder (2017)	[80]	Patient Satisfaction with telephone follow up was assessed in two studies with no differences noted satisfaction with information among women with endometrial cancer, APN-based telephone follow-up or APN counselling in 2/2 studies
APN Acute	Manoj (2019)	[63]	Patient Satisfaction : No statistical difference in scores before or after the implementation of APN-led DCCVs (p = 0.929) in 1/1 study. The favourable experience in this study highlighted that such services with excellent patient outcome can maintain high patient satisfaction. However, the author failed to provide the comparative data.
APN Acute	Medeiros (2011)	[94]	Improved patient satisfaction when NP added to team providing ICU care, also satisfaction from shortened wait time in clinic in 1/1 study

NP Acute AND Primary	Niezen (2014)	[107]	Patients' perceptions on NP care relate to the need of legitimizing one's disease. The wish to be seen by a doctor since this legitimizes a patient's illness is a societal countermovement that should not be ignored.
APN Acute	Schneider (2021)	[80]	Satisfaction noted in 4 studies with a trend towards improved to statistically significant improvements in 4/4 studies. Improvement in pain control or other symptoms related to disease and/or treatment reported in 5 studies with trend toward improvement to statistically improved results in 5/5 studies
NP Acute	Thamm (2019)	[109]	Equal to statistically significant improvements noted in patient satisfaction in the four included studies.
APN Primary Care	Whiteford (2016)	[111]	Patient satisfaction reported in two studies with improvements noted in 2/2 studies
APN Acute	Woo (2017)	[96]	Patient satisfaction in the Emergency room was reported in two studies with equal to statistically significant improvements noted in 2/2 studies. Patient satisfaction in critical care was reported in one study with similar scores between groups.
APN Acute AND Primary	Arian (2017)	[88]	Patient satisfaction: patients are satisfied with nurse prescribing and the consultation with the nurse prescriber in the included studies
NP Primary Care	Ansell (2017)	[100]	Patient satisfaction with open access: 3 studies. 16% increase post-intervention implementation in 1/3; no change in 2/3 studies. No p- values reported.
NP Primary Care	Carranza (2021)	[61]	Patient satisfaction: sig. higher with NP group in 4/7 studies (p-values reported for ¾ studies); not sig difference in 3/7
APN Primary Care	Donald (2013)	[44]	Resident satisfaction (1 study) with medical services did not differ statistically. Family members satisfaction significantly improved with 3 items including: [resident] seen often enough to treat problems, physician/ NP spends enough time with patient and one person in charge (1/1). Family member dissatisfaction lower in the intervention group (1/1).
NP Primary Care	Galiana-Camacho (2018)	[87]	Patient satisfaction: 97.3% of the patients were satisfied with the time devoted to their care, to deal with everything related to their health process. 83.8% of patients would return to return to the APN for similar problems, 14.4% would not, and 96.4% would recommend this care to a friend. The results of the satisfaction in the 5 categories of the questionnaire reached the highest level of satisfaction in 82% of the cases. Regarding the overall evaluation of care received, 82% rated it as "excellent", 15.3% as "good" and 15.3% as "good", and 0.9% as "poor"(2/2)
NP Primary Care	HQO (2013)	[62]	Patient satisfaction: Model 1: no sig differences between groups (p = 0.87) (1/1) Model 2: increase in patient satisfaction for patients receiving specialized nursing care, one study assessed significance (2/2)
NP Primary Care	Ismail (2013)	[102]	Satisfaction: rates with the scheme from A&E doctors, health workers, and residential care staff was high.
NP Primary Care	Jennings (2015)	[103]	Satisfaction: higher satisfaction in 3/3 studies. After adjusting for waiting time the emergency NP service still maintained a 1.5 x higher mean total satisfaction score (beta coefficient = 1.5, p = 0.004, 95% CI, 0.48–2.5).Responses to 12 out of 16 questions in a survey demonstrated a significant between the two groups in favour of the emergency nurse practitioner services (p <0.05) 100% of patients seen by emergency nurse practitioner services in the fast-track area scored their care as good or excellent
NP Primary Care	Jeyaraman (2022)	[104]	Patient satisfaction (3 studies) 2/3 studies reported high level of patient satisfaction with NP-led triage model (p values not reported). 1/3 studies reported a slight decrease in patient satisfaction with NP led-triage without reaching statistical significance
NP Primary Care	McParland (2022)	[35]	Patient satisfaction (1 study) Significant improvement in communication 1/1 study, no changes in other dimensions of patient satisfaction

NP Primary Care	Morilla-Herrera (2016)	[36]	Patient satisfaction with ED discharge care 1/2 studies found increased satisfaction with ED discharge (3.41 versus 3.03; mean difference 0.37; 95% CI 0.13– 0.62). The intervention was more effective for high-risk than low risk aged people. ½ studies found no significant group differences in patient satisfaction.
APN Primary Care	Newhouse/ Stanik-Hutt (2013)	[39-40]	Patient satisfaction with provider/care: 4 studies; 1/4 studies reported findings favouring NP group, 3/4 reported no diff. between groups (no p-values reported)<br>CNS: Satisfaction. Three studies reported satisfaction. Studies were conducted inpatient oncology and community settings. Equal to a trend towards improved patient satisfaction noted when comparing CNS and non-CNS groups in 3/3 studies.
NP Primary Care	Patel (2019)	[108]	NP SoP and patient satisfaction with care: 2 studies. One study reported a smaller percentage of the population not seeking care due to costs in two states with less restrictive versus two other states' most restrictive NP SOP policies, contradictory findings from another study reported increased patient difficulties with cost in states with the least restrictive NP SOP policies. This study also found that patient satisfaction with usual source of care and wait times was worse in states with the least restrictive NP SOP policies.
NP Primary Care	Sun (2022)	[38]	Patient-family satisfaction (4 studies) More than 95% of patient participants were satisfied with the NP intervention in 2/2 studies. (p value not reported). Caregiver satisfaction was significantly higher in the intervention group (99.8 vs 88.8, $p < .05$) in ¼ studies. Caregiver satisfaction increased 0.2 points on a 10-point scale postintervention, although it was not statistically significant.
NP Primary Care	Swan (2015)	[51]	Patient satisfaction (4 studies) Three studies/4 demonstrated higher patient satisfaction among patients who received care from APNs; one/4 study reported higher satisfaction among patients who received care from APNs at three of their ten study sites.
NP Primary Care	Thomas (2019)	[110]	Patient satisfaction at three months: The CNP intervention may have improved participant satisfaction at three months (dissatisfaction: 13/109 (12%) in the treatment group versus 17/45 (38%) in the control group; RR 0.32, 95% CI 0.17 to 0.59
NP Primary Care	Tsiachristas (2015)	[52]	Patient satisfaction (8 studies) 5/8 studies showed positive results, no p values reported
CNS Acute	Kilpatrick (2014)*	[55]	Mixed findings noted for patient satisfaction with statistically lower scores noted for men with prostate cancer at the end of treatment in ½ studies.
APN Acute	Kilpatrick (2015)-Inpatients*	[56]	NP group: Parental satisfaction: No significant differences
NP Primary Care	Donald (2015)* (transition)	[33]	Patient satisfaction post hysterectomy (effect size: 14 (95%CI: 3.5 to 24.5) <0.01
NP Primary Care	Martin-Misener (2015)*	[69]	Patient satisfaction (2 studies) meta-analysis of two studies in which the nurse practitioners had at least 1 year experience, nurse practitioner care was associated with higher patient satisfaction (1515 patients; $I2=0\%$) (mean difference: 0.15 (95% CI 0.11 to 0.20); $p<0.0001$) and parent satisfaction (804 parents; $I2=0\%$) (mean difference: 0.23 (95% CI 0.16 to 0.30); $p<0.0001$). Patient satisfaction with newly established NP roles (1 study) nurse practitioner care was associated with higher patient satisfaction in the subgroup of patients with chronic disease (583 patients) (mean difference: 0.24 (95% CI 0.05 to 0.43); $p=0.02$) (11-point Likert scale; LQE).
			**Signs and Symptoms (18 reviews)**
APN Primary Care	Chan (2018)	[42]	Symptom burden was reported in 12 studies with equal to statistically significant improvements in symptom burden or resolution of symptoms between those who received care from advanced practice nurses and from physicians, a multidisciplinary team or standard care
NP Acute	Alotaibi (2020)	[75]	Five studies showed that ANPs provide support to elder patients which helps to alleviate stress. ANPs can aid patients in symptom management. Symptoms were addressed prior to next scheduled clinic appointments (timely care)

APN Primary Care	Cheng (2018)	[77]	Four RCT trials were included in the meta-analysis to evaluate the symptom scales of appetite loss, constipation, diarrhea, dyspnea, financial impact, and insomnia. No heterogeneity was detected across these 4 trials for appetite loss (Q value=2.21, df=3, P=.531; I 2=0.0%), diarrhea (Q value=2.13, df=3, P=.545; I 2=0.0%), but a significant heterogeneity was for constipation (Q value=9.46, df=3, P=.024; I 2=68.3%), dyspnea (Q value=10.40, df=3, P=.015; I 2=71.2%), financial impact (Q value=7.34, df=3, P=.062; I 2=59.1%), and insomnia (Q value=31.65, df=3, P=.000; I 2=90.5%). Therefore, a fixed or random-effects model was adopted, respectively. The pooled analysis showed there was no difference in appetite loss (SMD=0.07, 95% CI=0.11 to 0.25; P=.452), diarrhea (SMD=0.02, 95% CI=0.20 to 0.16; P=.838), and dyspnea (SMD=0.12, 95% CI=0.24 to 0.49; P=.509) between patients undergoing nurse-led management and not, but the nurse-led management strategy significantly decreased the occurrence of constipation (SMD=0.36, 95% CI=0.71 to 0.00; P=.001) (Fig. 2) and insomnia (SMD=0.33, 95% CI=0.99 to 0.32; P=.011) (Fig. 3), and reduced the financial difficulty (SMD=0.34, 95% CI=0.65 to 0.03; P=.033) (Fig 4) Six RCT trials were included in the meta-analysis to evaluate the symptom scales of fatigue, pain, nausea, and vomiting. A significant heterogeneity was observed for fatigue (Q value= 23.41, df=5, P=.000; I 2=78.6%) and pain (Q value=11.15, df=5, P=0.048; I 2=55.2%), but no heterogeneity for nausea and vomiting (Q value=7.47, df=5, P=.188; I 2=33.0%). Hereby, a random-effects or fixed model was utilized, respectively. The combined results indicated the nurse-led management strategy had no significant influence on the fatigue (SMD= 0.03, 95% CI=0.35 to 0.28; P=.841), pain (SMD=0.05, 95% CI=0.16 to 0.27; P=.624), nausea and vomiting (SMD= 0.08, 95% CI=0.22 to 0.06; P=.264) of patients with cancer.
APN Primary Care	Kennedy (2012)	[92]	Symptomatology improvement found in 2 qualitative studies and in 8 quantitative/survey studies
APN Acute	Kobleder (2017)	[78]	Symptom management was assessed in two studies with statistically significant reductions in symptom burden noted in the intervention group for women with ovarian cancer in ½ studies and no differences between the groups noted ½ studies for women with vulva neoplasia. Statistically significant reductions in uncertainty noted in the intervention group in 1/1 study. A trend toward improved scores for depressive symptoms in the control group noted in 1/1 study.
APN Acute	Monterosso (2019)	[60]	Symptom management, overall not significant. Appetite loss during the intervention (4–6 months) was significantly lower in the control group by 4.43 units on average (MD = 4.43, 95%CI [0.08, 8.78], 354 participants, p = 0.05; I2= 0%; Fig. 5) After the intervention, symptoms of fatigue were significantly reduced in the intervention group by 4.45 units on average (MD = −4.45, 95%CI [−7.93, −0.97], 647 participants, p = 0.01; I2= 15%; Fig. 6).
NP Primary Care	Kwok (2022)	[99]	Symptom severity-Pain was the only symptom with enough data to perform a meta-analysis and was significantly reduced by the telehealth interventions (SMD − 0.54; 95% CI − 0.88, − 0.19) in two CBA studies
NP Primary Care	Nikpour (2022)	[112]	Nonopioid pain management strategies and symptom management : NPs were more likely to than physicians to use non-opioid pain management strategies in 3/3 studies and less likely to order low value services (e.g., scans) in 2/2 studies.
NP Primary Care	Carranza (2021)	[61]	Symptom management examined in four studies. No sig difference between NP and physician group.<br>Symptom improvement: Significantly improved with NP group (1/1).
NP Primary Care	Garner (2017)	[46]	Pain assessed in 4/4 studies. Sig reduction in ¾ studies and no difference in ¼ studies at 12 and 24 months<br>Fatigue assessed in 3 studies. Sig. less fatigue in 2/3 studies and no difference noted at 12 months and 24 months in 1/3 studies.<br>Arthritis impact assessed in 3 studies. No differences noted in 3/3 between nurse-led care and rheumatologist-led care or trainee rheumatologist-led care.
NP Primary Care	Kuethe (2013)	[85]	Symptom-free days: No sig difference between the paediatrician-led group and the nurse-led group (p =0.54). (1/1 study,)
NP Primary Care	Morilla-Herrera (2016)	[36]	Symptoms of dementia (NPI score) 1/1 study showed significantly fewer behavioral and psychological symptoms of dementia as measured by the total NPI score at 12 months (mean difference, -5.6; P: 0.01) and at 18 months (mean difference, -5.4; P: 0.01)
NP Primary Care	Norful (2019)	[66]	Cognitive and behavioural changes: 2/2 studies: no significative cognitive and behavioral changes. Significant change when using different assessment tools (p=0.01).

NP Primary Care	Schadewaldt (2011)	[67]	Angina symptoms 1/2 studies: Statistically significant differences between the groups at 1-year follow up were found in exertional capacity (P = 0.0014) and angina frequency (P = 0.0452), where the intervention group scored higher and therefore had less angina symptoms. No differences between usual care and nurse-led clinics were identified in other domains of the questionnaire such as stability of angina symptoms, satisfaction with treatment and quality of life. ½ studies: No significant difference between the groups at 1-year and 4-year follow up, except that worsening chest pain was experienced significantly less frequently in the intervention group (P = 0.025) after attending a nurse-led clinic for 1 year Angina symptoms
NP Primary Care	Thomas (2019)	[110]	Number of participants continent after treatment: Structured assessment and management of incontinence probably made little or no difference to the number of people continent three months after treatment (risk ratio (RR) 1.28, 95% CI 0.81 to 2.02; 121 participants; equivalent to an increase from 354 to 453 per 1000, 95% CI 287 to 715). At six months, the CNP intervention may have made little or no difference to the number of people continent after treatment (16/91 (17.5%) in the treatment group versus 8/55 (14.6%) in the control group; RR 0.96, 95% CI 0.83 to 1.11).<br>Urinary symptoms (frequency, nocturia, urgency and urinary incontinence) at three months: number of participants cured of all four urinary symptoms was 24.7% in the treatment group versus 17.9% in the control group (147 participants). At six months, Brittain 2000b found a larger proportion of people were cured of all four urinary symptoms in the treatment group (41/89; 46.1%) compared with the control group (16/54; 29.6%) (RR 1.55, 95% CI 0.97 to 2.48).<br>Changes in daytime and night-time leakage: no data provided<br>Daytime severity of leakage at three months: (P = 0.038) possibly improved following the intervention: Total number of symptoms experienced at three months: may have slightly reduced after the intervention (P <0.01). Total number of overall symptoms at six months: little or no difference (P = 0.06).<br>Urinary frequency at three months: No evidence that the CNP intervention made any difference 98/120 (82%) in the treatment group versus 59/67 (88%) in the control group; RR 0.93, 95% CI 0.82 to 1.05; 187 participants. Urinary frequency at six months: The CNP intervention may have made little or no difference to urinary frequency (73/89 (82%) in the treatment group versus 47/54 (87%) in the control group; RR 0.94, 95% CI 0.82 to 1.09).<br>Urinary urgency at three months: no difference. The number of people reporting urgency was 95/121 (79%) in the treatment group compared with 50/67 (75%) in the control group (RR 1.05, 95% CI 0.89 to 1.24; 188 participants). Urinary urgency at six months: the CNP intervention may have made little or no difference to (65/91 (71.4%) in the treatment group versus 40/54 (74%) in the control group; RR 0.96, 95% CI 0.79 to 1.18).<br>Nocturia at three months: the CNP intervention may have made little or no difference to nocturia (Brittain 2000b). The number of people reporting nocturia at three months was 102/119 (86%) in the treatment group versus 60/67 (90%) in the control group (RR 0.96, 95% CI 0.86 to 1.07). Nocturia at six months: At six months, the CNP intervention may also have made little or no difference to nocturia (77/89 (87%) in the treatment group versus 46/53 (87%) in the control group; RR 1.00, 95% CI 0.87 to 1.14).
NP Primary Care	Wu (2020)	[113]	Management of asymptomatic urinary tract infection: improvement in following supportive strategies: increased fluids (p <.001), frequent toileting (p <.001), and cranberry juice (p <.05).
APN Acute	Kilpatrick (2015)-Inpatients*	[56]	NP group: Quality of documentation: statistically significant improvements<br>Symptom management: Jaundice: statistically significant improvements (neonate)
NP Primary Care	Donald (2015)* (transition)	[33]	Urinary tract infections: outcomes favoured usual care

* Systematic review published as constellation papers, with additional methodological data extracted from: 154. Marshall D, Donald F, Lacny S, Reid K, Bryant-Lukosius D, Carter N, et al. Assessing the quality of economic evaluations of clinical nurse specialists and nurse practitioners: A systematic review of cost-effectiveness. NursingPlus Open. 2015;1(2015):11-7.doi:10.1016/j.npls.2015.07.001; and 155. Donald F, Kilpatrick K, Reid K, Carter N, Martin-Misener R, Bryant-

Lukosius D, et al. A systematic review of the cost-effectiveness of nurse practitioners and clinical nurse specialists: What is the quality of the evidence? Nurs Res Pract. 2014;2014. doi:10.1155/2014/896587.

A&E, accident & emergency; ADL, activities of daily living; AMBRA, AMBulant behandling af Reumatoid Artrit; ANP, advanced nurse practitioner; APN, advanced practice nurse; BADL, basic activity of daily living; BDI, Beck Depression Inventory; BMD, bone mineral density; BMI, body mass index; BP, blood pressure; CAD, coronary artery disease; CBA, controlled before-after; CHF, congestive heart failure; CI, confidence interval; CNP, continence nurse practitioner; CNS, clinical nurse specialist; COPD, chronic obstructive pulmonary disease; CVD, cardiovascular disease; DBP, diastolic blood pressure; DCCV, direct current cardioversion; df, degrees of freedom; ED, emergency department; EF, ejection fraction; FENO, fraction of exhaled nitric oxide; FEV1, forced expiratory volume in 1 second; FVC, forced vital capacity; GAD, generalized anxiety disorder; HA1C, hemoglobin A1C; HbA1c, hemoglobin A1c; HDL, high-density lipoprotein; HF, heart failure; HIV, human immunodeficiency virus; HR, hazard ratio; HRQL, health-related quality of life; HRQoL, health-related quality of life; IADL, instrumental activity of daily living; ICU, intensive care unit; IQR, interquartile range; LDL, low-density lipoprotein; LDL-C, lipoprotein cholesterol; LQE, low quality evidence; LTC, long-term care; MD, mean difference; MD, medical doctor; MUIS, Mishel Uncertainty in Illness Scale; NFU, nurse-led follow-up; NP, nurse practitioner; NPI, Neuropsychiatric Inventory; NS, non significant; NTproBNP, N-terminal pro-brain natriuretic peptide; OR, odds ratio; PD20, provocative dose of methacholine causing a 20% fall in forced expiratory volume in one second; PEF, peak expiratory flow rate; PMHNP, psychiatric mental health nurse practitioner; QALY, quality-adjusted life year; QLQ-C30, Quality of Life Questionnaire C30; QoL, quality of life; RCT, randomized controlled trial; RR, relative risk; RR, risk ratio; SBP, systolic blood pressure; SCSI, Structured Catastrophic Stress Intervention; SD, standard deviation; SDS, Symptom Distress Scale; SF-12, Short Form 12; SF-36, Short Form 36; sig, significant; SMD, standard mean difference; SoP, scope of practice; STAI, State-Trait Anxiety Inventory; SUD, substance use disorder; TC, total cholesterol; TG, triglyceride; UAER, urinary albumin excretion rate; WMD, weighted mean difference.
