# Supplementary material for: A global perspective of advanced practice nursing research: A review of systematic reviews
Source: PLoS One. 2024 Jul 2;19(7):e0305008. doi: 10.1371/journal.pone.0305008 (PMC11218965; doi:10.1371/journal.pone.0305008)
Supplement: S4 Table — (PDF) [file pone.0305008.s008.pdf]

S4 Table. Extraction of review results by indicator category at the Provider level.

			PROVIDER
**Role**	**Author (year)**	**Ref**	**Results**
			**Adherence to Best Practice-Provider (10 reviews)**
APN Primary Care	Emrich-Mills (2019)	[114]	Nurses and physicians in memory services have reported that nurse prescribers have improved continuity of care and rapport-building for service users.
APN Acute AND Primary	Gielen (2014)	[48]	Medication adherence: Only one study reported on medication adherence; it found no significant difference
APN Acute AND Primary	Hyde (2020)	[115]	Case management was examined in three studies with no differences found between the groups.
APN Primary Care	Kennedy (2012)	[92]	Able to better meet targets: found in 1 qualitative study
NP Primary Care	Lovink (2017)	[34]	Renin–angiotensin system blockade (1 study) 68 patients on treatment with renin–angiotensin system blockade in the control group compared with 79 in the intervention group (P = 0.002). Percentage mean dosage of renin–angiotensin system blockade of the optimal dosage was 94% compared with 69% in the control group (P <0.001).<br>Adherence and compliance to guidelines, protocols and quality of healthcare: reported in 4 studies. 2 studies: not stat. sig. and 2 studies: stat. significant. In LTC: No significant difference was found in the number of annual mandatory histories and physical examinations performed. 1/1 study Assessing Care of Vulnerable Elders-3 (ACOVE-3) quality indicators in favour of the intervention, (P <0.001), 1/1 study Overall performance rate on secondary prevention performance: In primary care, sig improvement in 1/1 study, (p <0.001).
NP Primary Care	Norful (2019)	[66]	Adherence to recommended care: Indicators including completed guidelines for patients with dementia (p <.001), falls (p = .00), incontinence (p = .01), and all diagnoses (p <.001). 4/4 studies found sig results for adherence to care and co-management<br>Medication compliance: no sig differences between the groups (1/1)
NP Primary Care	Schadewaldt (2011)	[67]	Compliance: No sig difference between groups in ½. Sig difference in ½ studies: compliance with aspirin intake at 1-year follow up adherence to correct aspirin intake was significantly higher in the intervention group (P < 0.001).
NP Primary Care	Swan (2015)	[51]	Clinician guideline adherence (3 studies) One/3 found that APNs had higher rates of providing disease-appropriate care across five of six indicators examined (p value not reported). There were no differences regarding the proportion reporting that they were advised of the likely duration of their illness and how to reduce the chances of recurrence. Patients assigned to the physician group were less likely to report having been told the cause of their illness (odds ratio [OR] 0.58, 95% confidence interval [CI] 0.44–0.76), how to relieve symptoms (OR 0.32, 95% CI 0.24–0.43) and what to do if the problem persisted (OR 0.61, 95% CI 0.41–0.90) in 1 study
NP Primary Care	Turi (2023)	[81]	Mental Health Guideline-Recommended Care (4 studies): No significant differences in the care provided by NPs related to adherence to guidelines for medications, counselling, cognitive behavioral and problem-solving therapy to older adults, patient monitoring and motivational interviewing in 4/4 studies
NP Primary Care	Donald (2015)* (transition)	[33]	Compliance with aspirin: Outcomes favoured usual care. Compliance with clopidogrel: outcomes favoured usual care<br>Compliance with beta-blockers: estimates favoured nurse practitioner care.<br>Compliance with statins: estimates favoured nurse practitioner care.<br>Compliance with angiotensin-converting enzyme inhibitors: estimates favoured nurse practitioner care

			Education-Provider (17 reviews)
APN Primary Care	Emrich-Mills (2019)	[114]	Activity logs of independent NPs in Scotland suggested they took increased opportunities to offer advice on issues such as side effects, correct administration and cost implications compared to medical prescribers. Several studies describe views that NPs require training in pharmacology, assessment and diagnosis, life sciences and clinical decision-making among other, less frequently repeated topics. Preferences include e-learning courses, discussion groups or workshops, local and national conferences, journals, clinical guidelines and the British National Formulary
APN Acute AND Primary	Hyde (2020)	[115]	Clinical Education was reported in two studies. Trends towards improved knowledge noted for patients, families, nurses and junior residents.
APN Primary Care	Kennedy (2012)	[92]	Raised opportunities for the nurse consultant found in 3 qualitative studies and in 1 quantitative study. Raised feeling of support for the nurse consultant: found in 1 quantitative Influencing training/education needs of others found in 2 qualitative studies and in 1 quantitative study.
NP Education	McQuilkin (2020)	[59]	The transition from clinician to the NP role can be very difficult. New faculty experience culture shock and concerns about maintenance of clinical practice. Orientation, peer support, and mentoring can mitigate the challenges and support the transition. We identified five key themes in the literature: orientation; culture shock and role strain; mentoring; peer support; and, concern about maintenance of clinical practice
NP Acute AND Primary	Niezen (2014)	[107]	NP Knowledge and capabilities Four studies described facilitators and barriers related to NPs' knowledge and capabilities. The NPs' knowledge and capabilities theme is divided in two subcategories: (1) self-knowledge, and (2) interpersonal skills.<br>Professional boundaries: The debate on NPs' qualification affects task reallocation, often discussed in relation to the standardisation, quality and adequacy of their education. It is the accessibility and uniformity of qualification and authorisation of the NP workforce that form an important factor regarding the NP's status.
NP Acute	Rutherford-Hemming (2016)	[118]	Thirteen studies evaluated increases in knowledge and/or competency with simulation with equal to statistically improvements noted in all studies, learner satisfaction and/or confidence in simulation were always positive in the six studies reporting this outcome.
APN Primary Care	Schallmo (2019)	[119]	Provider-Education: Areas for improvement reported in 5 studies and include additional advanced diagnostics and procedure skills (ADPS) training needed on multiple procedures after initial training. Program directors reported numerous ADPS as important but not included in curriculums 50% or more of the time ; program directors reported numerous ADPS as very important but not included in curriculums 50% or more of the time ; graduates relied on on-the-job training for clinical practice ; only 10.5 % of respondents felt well-prepared in ordering diagnostic imaging and only 6% felt prepared in ordering contrast ; 94% would like continuing education in diagnostic imaging ; many FNP graduates practice in emergency care where they need to be competent in ADPS; of FNP programs surveyed 80.7% had no specific program from procedures.
NP Primary Care	Speight (2019)	[120]	Both NPs and their mentors rated competencies as improving overtime in seven competency domains: clinical, leadership, interprofessional team collaboration, patient-centered care, shared decision-making, sustained relationships, and performance improvement. Competency tool analysis indicated that the NPs were prepared to practice independently by the end of the fellowship program, suggesting the intervention as a whole contributed to NP competency development. Respondents' mean NPRTS scores were not statistically significantly different after the intervention, suggesting that the intervention did not contribute to the improved professional transition of the NPs.
NP Acute	Veenema (2021)	[122]	Educational preparedness for ED practice was found in six studies. NPs reported high-priority learning needs that were met through continuing education and on-the-job training.

NP Acute AND Primary	Warren (2016)	[123]	Four studies reported on student satisfaction with high fidelity simulation. NP students noted that high fidelity simulation improved their critical thinking skills, evidence based practice and ability to function in the clinical setting. Knowledge was measured in eight studies with equal to statistically significant improvements noted.<br>Attitudes were measured in three studies with statistically significant improvements in students' level of confidence, recognition of status change, ability to identify cardiac rhythms that were shockable or non- shockable, defibrillator use. Instructor modeled vs self directed leaning influenced self-efficacy scores for NPs vs NP students, who scored significantly higher regardless of the method.<br>Skill performance was identified in three studies. Equal to statistically significant improvements noted in the time to start administration of medication, decision to do a lumbar puncture, history taking and clinical management of NP students, and critical thinking.
NP Acute AND Primary	Chua (2023)	[116]	Positive learner experience reported in relation to competency in knowledge and clinical skills, effective communication and satisfaction.<br>Standardized patient experience allowed for reflection, safe and realistic learning environment, and opportunities for intra-professional collaboration Student preference and way forward included the need to effective feedback, performance by standardized patients and preparation before standardized patient experience.
NP Primary Care	Galiana-Camacho (2018)	[87]	Professional competencies were assessed in nine studies using the Australasian Triage Scale (ATS) and found that NPs primarily saw patients with a non-urgent status with scores of between 4-5 (minimum of 5 and maximum 1) (9/9)
NP Primary Care	Hyer (2019)	[117]	Patient counseling on obesity: 65% of primary care physicians, obstetricians, and NPs surveyed believe this is a shared responsibility between the patient and the provider). (1/1)
NP Primary Care	Loescher (2018)	[72]	Current knowledge Five studies reported on NPs' knowledge of skin cancer detection). Correct responses on overall knowledge tests ranged between 26% and 85%.<br>NP attitudes towards the early detection of skin cancer. (3 studies) 41% reported No to mild confidence, with participants feeling a lack of confidence with basic dermatology examinations. 84% agreed that "dermatology training prepared me for practice." In one study, skin lesion identification by NPs and Knowledge of clinical examination were low but improved with training. Accuracy of skin lesion identification, errors tended to occur with benign skin lesions. Skin cancer detection training: Didactic training in 3/5 studies and informal training in 2/5 Didactic educational program improved skin lesion recognition and resulted in a 223% increase in participants' proper documentation of skin cancer screening and patient education in one study (Bradley, 2012)
NP Primary Care	Lovink (2017)	[34]	Orientation in LTC: significant improvement in 1/1 study (p = 0.02),
NP Primary Care	Stratton (2020)	[121]	Intervention components and activities for NPs performing clinical skin examination: (10 studies) 10/10 studies reported a didactic and a clinical portion or clinical apprenticeship with a dermatologist, with 8/10 8 focused on head-to-toe skin examination 6/10 studies did not specify the strategy for conducting skin lesion assessment Feedback on the NPs prior dermatology referrals to guide her education 1/1 study Intervention dosing The length of their didactic sessions (1 session of 14 min up to 6 months), timing and frequency were not specified, most sessions occurred one time (4 studies) Not mentioned in 3 studies. Mode of delivery of the intervention: 7/10 reported using face to face and observations by experts.
APN Primary Care	Donald (2013)	[44]	Following staff educational sessions provided by CNSs in 2 studies:<br>Urinary incontinence: Similar improvements noted in the control and intervention groups in ½ studies and significant improvements noted in ½ studies. no differences in the number of pressure ulcers or aggressions.<br>Restraint prevalence 56% relative reduction in INT2, 23% relative reduction in INT1, 11% relative reduction in CTR; within group comparison over time INT2 had significant reduction Chi2 = 25.5, df = 2, P < 0.001, trends towards reductions in restraint use in the CNS group (non sig.).<br>Restraint intensity: intervention group was 1.26 times more likely to decrease restraint use compared with INT1 and 1.35 times more likely to reduce restraint use compared with control group; continued physical restraint use more likely in the control group.

			Fall rates and minor injuries were significantly higher in the control group (P < 0.01).<br>Psychoactive drug use no increase in psychoactive drug use across 3 sites; decreased use of benzodiazepine use across all 3 sites (P<0.001); Mean staff hours unchanged
			**Illness Prevention (9 reviews)**
APN Acute	Bryant-Lukosius (2015)	[70]	Prevention: No studies were identified that compared advanced practice nurses with other healthcare providers in the delivery of cancer prevention services.
APN Acute AND Primary	Gielen (2014)	[48]	Follow-up consultations: Patients cared for by nurses make more return visits than patients cared for by doctors.
NP Primary Care	Carranza (2021)	[61]	Overall improvement in metabolic outcomes, but no p-values reported. NP vs. MD, within-group decrease (between- group difference): -HA1C: 2.5% vs 0.2% (2.3%) -BP: No difference within and between groups -Weight loss: 8.3 lbs. vs. 7.4 lbs. (0.9 lbs.) - Glucose: 83.7 mg/dl vs. 27.4 mg/dl (56.3 mg/dl)
NP Primary Care	Fung (2014)	[45]	Feasibility of screening of women with depression (1 study) Increase in the mean efficacy self-esteem score post-intervention and a significant difference between pre- and post-intervention BDI scores (t = 8.765, d.f. = 29, P = 0.0005) in a paired samples t-test. (1/1)
NP Primary Care	Garner (2017)	[46]	Health assessment assessed in 4/4 studies. Equal to superior care indicated at 12 and 24 months
NP Primary Care	HQO (2013)	[62]	Process indicator: height Model 1: (p < 0.01) Model 2: (no data) Clinical examination: Model 2: Patients in the specialized nursing group received significantly more assessments of smoking status (P < 0.0001) among CAD patients (3/3 studies).
NP Primary Care	Schadewaldt (2011)	[67]	Smoking: No sig results in 3/3 studies Smoking cessation in the long term for up to 1 year ½ studies sig for smoking cessation (p = 0.03) In 1/3 studies showed sig results at 12 months (p = 0.01) Bodyweight No sig difference in 3/3 studies. Compliance: No sig difference between groups in ½. Sig difference in ½ studies: Significant benefits in continuing physical exercise (P = 0.001).<br>Compliance: No sig difference between groups in ½. Sig difference in ½ studies: Significant benefits in diet (P = 0.009) after 1 year in nurse-led group.
NP Primary Care	Smith (2014)	[73]	Cervical cancer screening (5 studies) 3/4 studies evaluating Pap tests, most showed that APRN/PAs provide or recommend Pap tests to patients (72% to 98%) and that physicians who currently work with APRN/PAs are amenable to APRN/PAs conducting Pap tests 1/1 intervention study compared NPs recommending and performing cervical cancer screening during routine visits to a provider reminder system. At follow-up there was a significant increase in the annual rate of women screened for cervical cancer by a NP at the intervention location (from 17.8% to 56.9%), while the annual rate of screening by physicians at the control location improved less (from 11.8% to 18.2%) during the study time period, p = 0.006, after adjusting for patient characteristics.<br><br>Breast cancer screening (3 studies) 2/3 studies showed that a majority of patients who see NPs receive mammograms (69% to 91%) (p < 0.01) and that NPs recommend a similar amount of mammograms as physicians. 1/3 not sig. 1/1 a majority of patients who see NPs receive mammograms (69% to 91%) and that NPs recommend a similar amount of mammograms as physicians, p values not reported.<br><br>Colorectal cancer screening (4 studies) ¾ studies showed a range of reported colorectal cancer screening provided or recommended by APRN/PAs (19% to 95%) (p < 0.001). Large variation due to differences in reporting between studies (e.g., chart audit, self report) 2/4 studies showed that physicians reported more colorectal cancer screening than APRN/PAs; p < 0.001 in one study; not reported in other study.<br>HPV vaccination: No study of HPV vaccination identified.
NP Primary Care	Donald (2015)* (transition)	[33]	Smoking cessation: Outcomes favoured usual care

			Interprofessional Team Functioning (5 reviews)
APN Acute AND Primary	Arian (2017)	[88]	Interprofessional team functioning: Communication with care team and physicians helped build a trusting relationship and reduce hierarchy.
NP Primary Care	Hyer (2019)	[117]	Provider perceptions of high functioning interdisciplinary team approach: (2/2) positively affected the patient provider experience
NP Primary Care	Lovink (2017)	[34]	No outcomes related to providers were reported
NP Primary Care	McParland (2022)	[35]	Team Functioning (1 study): Staff perceived that patient care had improved following the intervention, also identified gaps particularly in communicating with secondary care in 1/1 study
NP Primary Care	Turi (2023)	[81]	Collaborative Care (6 studies): NP-led collaborative care where the PCP was an NP and psychiatric support was provided by a PMHNP. NP-led collaborative care where PCP NPs screened patients who screened positive; high-acuity patients were referred from the PCP NP to the PMHNP. After one year, the number of case reviews per month were higher (5 to 15) and the number of referrals to the PMHNP were lower (19 to 5). (no p value, effect measured using depressive symptoms). NP-led collaborative care (5 studies) NP-led collaborative care led to clinical or statistical improvements in depressive symptoms in 5/5 studies
			**Prescribing (24 reviews)**
APN Primary Care	Chan (2018)	[42]	Medication management: Medication management described in four reviews. Improvements noted in medication management of low complexity diseases in the community, patients with chronic kidney disease, and dyspepsia, follow-up visits with the advanced practice nurse and time on anti-depressant medications, less "full dose protein pump inhibitors" use, more "no protein pump inhibitor treatment.
APN Primary Care	Emrich-Mills (2019)	[114]	Earlier prescriptions reported for 81% of patients. People often valued the prescribing qualification regardless of the profession.
APN Acute AND Primary	Gielen (2014)	[48]	Number of patients prescribed medication: Nurses appear to prescribe for just as many patients as physicians do. Number of medicines prescribed per patient visit nurses prescribe comparable numbers of medicines per patient visit. Type of medication and dose prescribed there appear to be few differences between nurses and physicians in the type and dose of medication prescribed and in clinical outcomes. Investigations Results concerning differences in the number of investigations by nurses and physicians are mixed
APN Acute AND Primary	Hyde (2020)	[115]	Guideline adherence and prescribing practice was examined in two studies. No differences were found in guideline compliance in the studies.
APN Primary Care	Kennedy (2012)	[92]	Improved medication rates: found in 1 quantitative study
CNS Acute	Salamanca-Balen (2018)	[126]	Statistical significant changes in 4/5 studies. 1/5 study found that CNS requested significant more blood samples compared to surgeons when following up colorectal cancer patients. Medication prescriptions were significantly increased in the intervention group in two studies and medication uptake/use by patients was raised in another three studies. A study of a CNS-led supportive care intervention for patients with head and neck cancer found that in the intervention group, there was a statistically significant chemotherapy treatment completion and dose reduction compared to the control group. Four studies either did not find or did not report significant levels on this outcome.
APN Acute	Barrott (2023)	[124]	Prescribing of systemic anti-cancer therapy (SACT) treatments was identified in 4 studies with approximately half of nurses reported engaging in chemotherapy prescribing with physician and policy restrictions on prescribing noted in 4/4 studies. One study reported in two reviews noted nurses involvement in managing chemotherapy treatment-related toxicities.

NP Primary Care	Nikpour (2022)	[112]	Increasing opioid prescribing rates for NPs and PAs over time, potentially attributable to an increase in legislation allowing independent scope of practice for NPs and Pas in 2/2 studies. In contrast, states with expanded scope of practice, opioid prescriptions increased for both NPs and physicians relative to restricted practice states, suggesting that scope of practice had the same effect regardless of provider type. Theme 2: Similarities and differences in opioid prescribing by provider type. No difference in opioid prescribing rates between physicians and NPs noted in 3/3 studies with similar findings for NPs and PAs in 5/5 studies. NPs appear to be less likely to prescribe an opioid or be acute users at baseline to Medicare beneficiaries but they are more likely to prescribe higher doses of morphine milligram equivalents (MME) in 2/2 studies.<br>NPs and PAs were not always reported as separate groups in administrative databases. NPs and PAs were more likely than physicians to be high-volume opioid prescribers in 3/17 studies. The authors suggested that NPs may be less likely to write opioid prescriptions but more likely to prescribe higher levels of MMEs when pain becomes chronic
APN Acute AND Primary	Arian (2017)	[88]	Prescribing is influenced by care team support and time in 4 studies. It is not sufficient to have a regulatory framework in place, physicians must also trust that nurses can act as prescribers. Physician recognition of advantages of nurse prescribing can support development of these activities. Some physicians believe that nurse prescribing goes against a top-down approach. Increasing nurses' knowledge and role clarity can support expansion of the prescriber role. No significant differences were noted in physician and nurse prescribing.
NP Primary care	Barker (2018)	[89]	Prescribing (unchanged 2/3 (one p value not reported); 1/3 reduction)
NP Primary care	Carranza (2021)	[61]	Treatment success: 4/4 no sig difference between NP and physician groups
NP Primary Care	Driscoll (2015)	[90]	Patients initiated or up-titrated on beta-blockers: (1 NP study) Proportion of patients with stable CHF in outpatient settings who were initiated or up-titrated and maintained on beta-blockers, was achieved in 67% (36 of 54) of patients in the nurse facilitator group compared with 16% (10 of 64) in the provider/patient notification and 27% (14 of 51) in the control groups ($P < 0.001$) for the comparisons between the nurse facilitator group and both other groups). (1/1) Patients initiated on beta-blockers: (1 NP study) Proportion of patients with stable CHF in outpatient settings who were initiated on beta-blockers (beta-blocker-naïve at the start of the study) was highest in the nurse facilitator group at 61% (22/54), compared with control (29%) and provider/patient notification groups (12%) (P<;0.001). (1/1) Patients at target beta-blocker doses at end of study: (1 NP study) Proportion of patients with stable CHF in outpatient settings on target beta-blocker doses at the study end (median follow-up, 12 months) was also highest in the nurse facilitator group (43%) compared with the control (10%) and provider/patient notification groups (2%) ($P <0.001$). (1/1)
NP Primary care	Garner (2017)	[46]	Prescribing laboratory investigations: trainee rheumatologist-led care ordered more laboratory investigations (relative risk 0.46, 95% CI 0.2–0.9; $p = 0.03$)
NP Primary Care	HQO (2013)	[62]	Medication Prescribing Model 2: sig. differences in prescription to intensify glucose-lowering therapy ($p = 0.0005$) or blood pressure medication ($p = 0.01$), number of referrals to internists were higher ($p < 0.001$), and influenza vaccination ($p< 0.0001$), significant increase in the primary outcome of the appropriate prescribing of beta-blockers among individuals with a prior myocardial infarction ($P = 0.03$) Diverse results for Aspirin use ½ study with sig results. No sig differences in lipid-lowering agents ($p= 0.07$), no significant difference in the prescribing of an angiotensin converting enzyme (ACE) inhibitor among patients with confirmed LVSD ($P = 0.05$). However, it was not stated how many patients were already on insulin or if this increase reflected more appropriate referrals in comparison to physicians ($P = 0.03$) (4/4). Two studies reported on Aspirin use, with Khunti et al finding No significant difference in the proportion of patients receiving aspirin ($P = 0.55$) in one study, and a significant increase in use ($P < 0.001$) in one study
NP Primary Care	Hyer (2019)	[117]	Prescribing: NP prescribe from rarely to half of study participants reporting the practice of using weight-loss pharmaceuticals (3 studies); the reported thresholds for weight-loss pharmaceuticals, that prompt the prescription were not aligned with national guidelines of pharmacotherapy in 2 studies. Referrals for bariatric surgery varied greatly from rarely making a referral to 70% of participants referring out for consultation.

NP Primary Care	Kuethe (2013)	[85]	Use of rescue medication No sig difference in 2/2 studies in unstable patient group (Mean number of exacerbations requiring emergency treatment was 0.59 in nurse-led group vs 0.43 in the physician-led group).<br>Evidence of stepping down therapy (1/1): No sig differences between the groups
NP Primary care	Lovink (2017)	[34]	Beta-blockers: No sig. difference in patients receiving this treatment (1/1). Beta-blockers optimal dose: No sig difference (1/1) In LTC: Average number of medications: no sig difference in 1/1 study
NP Primary Care	Morilla-Herrera (2016)	[36]	Prescriptions-cholinesterase inhibitors 1/1 study showed that patients were more likely to receive cholinesterase inhibitors (p = 0.002) Prescriptions-antidepressants 1/1 study showed that patients were more likely to receive antidepressants (p = 0.03)
NP Primary Care	Ness (2016)	[125]	Totals and percentages of the type of antimicrobial (AM) selected, demonstrated that nurse practitioners in this study chose traditional and often low-cost antimicrobial medications and that these choices were made because the drugs were first-line choices (1/1) Prescribing decisions based on: Efficacy (94,2%) and tolerability (77,1%) were most frequently identified as very important influences on their prescribing decisions with guideline recommendation coming third (74%). (2/2) Prescribing decisions in pediatric settings are: always (3,4%) or very often (51,6%) based on watchful waiting instead of prescribing an antibiotic as the first strategy for managing otitis media. (1/1) Prescribing decisions when no culture is available: majority of respondents (60,3%) stated that they would start with a broad spectrum antimicrobial and then tailor upon culture results (1/1) Predictors of prescribing an anti-microbial: strongest negative predicator (i.e. would not be prescribed an AM) within the nurse practitioners was Medicaid insurance (p = 0,012). Patients with Medicaid insurance were 75% less likely to be prescribed an AM. The strongest positive predictors were that NPs were significantly more likely to prescribe an antimicrobial were geographic region (p = 0,001), black non-Hispanic race (p = 0,047) and when both pharyngitis and bronchitis were diagnosed as compared to patients with a nonspecific viral upper respiratory tract infection (p = 0,001). (1/1)
NP Primary Care	Swan (2015)	[51]	Prescription patterns for medications (three studies); one study reported: no differences in APN medication prescriptive practices. Prescription of diagnostic tests (three studies); one study reported a trend that APNs more frequently ordered diagnostic tests (not sig.).
NP Primary Care	Turi (2023)	[81]	Prescribing (3 studies) NPs in community health centers prescribed more antidepressants (70.4% vs. 61.6%, p = 0.03) and the same number of anxiolytics (24% vs. 33%, p>0.05) in 1 study. NPs prescribed more anti-anxiety and antidepressants in rural areas vs. urban areas (p < 0.001) in 1 study. No difference in NP-attributed patients in the odds of receiving alcohol use disorder pharmacotherapy compared to physician-attributed patients (primary care NP: AOR = 1.00, 95% CI = 0.69 – 1.46; psychiatry NP: AOR = 1.33, 95% CI = 0.67 – 2.65).
NP Primary care	Wu (2020)	[113]	Antibiotic prescribing rates: No significant differences
NP Primary Care	Zhang (2020)	[127]	Meta-analysis (26 studies) Stage of pre-exposure prophylaxis (PrEP) implementation cascade between NPs and physicians: being aware of pre-exposure prophylaxis (PrEP) among NPs were 37% (OR = 0.63, 95% CI = 0.46–0.87) less than that among physicians. Meta-analysis (26 studies) Prescribing pre-exposure prophylaxis (PrEP): Odds of being willing to prescribe pre-exposure prophylaxis (PrEP) among NPs was not different from that among physicians (OR = 1.00, 0.98, 1.02). The odds of prescribing pre-exposure prophylaxis 1.40 (95% CI = 1.02. 1.92) times higher among NPs than that among physicians. No significant difference for location and study design. The odds ratio of prescription between NPs and physicians was highest in 2015 among all available studies (OR = 1.94, 95% CI = 1.12, 3.36) Low to moderate between- study heterogeneity was detected across studies evaluating PrEP awareness (I2 = 0.00%), willingness (I2 = 54.00%), and prescription (I2 = 55.50%). Sensitivity analyses, including or excluding studies with extreme weights, showed no significant difference.

NP Primary Care	Martin-Misener (2015)*	[69]	Number of prescriptions. Nurse practitioner and general practitioner care were equivalent. Non sig. Number of investigations ordered or carried out. Nurse practitioner and general practitioner care were equivalent. Non sig.
			**Satisfaction-Provider (9 reviews)**
APN Primary Care	Chan (2018)	[42]	One non-randomized controlled trial reported that a nurse practitioner-led dementia outreach service in residential aged care facilities resulted in significantly higher level of staff satisfaction with regards to dementia care.
APN Primary Care	Emrich-Mills (2019)	[114]	Mainly positive feedback from staff. Nurse prescribers' views of their roles, several reported greater confidence, respect, autonomy, and job satisfaction following qualification, in memory services and further. Some mental health nurse prescribers expressed regarding the role of nurse prescribers. Some mental health nurse prescribers have expressed anxieties over conflict of their own roles. Specifically, their nursing and prescribing roles were perceived to create a "care versus cure" dichotomy
APN Primary Care	Han (2018)	[129]	Participants were only minimally satisfied with their jobs. Professional growth and intrapractice partnership and collegiality were cited as making the highest contribution toward job dissatisfaction. Half of the studies found reasonable to high NP satisfaction.
APN Acute AND Primary	Hyde (2020)	[115]	Staff experience of care provision: three studies explored staff experience of care provision by APNs, Staff reported that APNs effectively managed asthma care, APNs decreased physician workload, and nurses found that APNs increased care continuity and timely access to care. Contradictory reporting of findings noted in an older study by Martin (1999).
NP Acute	Johnson (2015)	[105]	The majority of responses for the program were that primary care providers were "very satisfied" (46%) or "satisfied" (29%) with the nurse navigator role. Reliability and validity testing was not addressed in the article. More information is needed to determine the reliability and validity of the instrument.
APN Primary Care	Kennedy (2012)	[92]	Favorable view by other health care providers found in 6 qualitative studies and in 4 quantitative studies
APN Acute	Lyness (2021)	[130]	Improved confidence: Lack of confidence was commonly reported by NMPs, leading to anxiety and inefficiencies in the service. Nurses conducting telephone triage reported offering GP appointments to some patients who didn't need them, because they were worried about missing serious illness, and lacked the confidence to reassure patients and their parents over the phone. This led to both slower triage (as the nurses felt the need to document the history in detail) and excessive numbers of GP appointments
NP Acute	Rutherford-Hemming (2016)	[118]	Communication was the topic of research in five studies and improvements were noted.
APN Acute	Geese (2022)	[128]	Provider-Job Satisfaction in cancer care was identified in four studies using survey methods. The studies identified that good interpersonal relationships with patients, physicians and other providers, increasing years of experience, increasing role confidence and level of independent practice were associated with higher job satisfaction. Higher overtime hours were associated with minimal job satisfaction in 1/1 study.

* Systematic review published as constellation papers, with additional methodological data extracted from: 154. Marshall D, Donald F, Lacny S, Reid K, Bryant-Lukosius D, Carter N, et al. Assessing the quality of economic evaluations of clinical nurse specialists and nurse practitioners: A systematic review of cost-effectiveness. NursingPlus Open. 2015;1(2015):11-7.doi:10.1016/j.npls.2015.07.001; and 155. Donald F, Kilpatrick K, Reid K, Carter N, Martin-Misener R, Bryant-Lukosius D, et al. A systematic review of the cost-effectiveness of nurse practitioners and clinical nurse specialists: What is the quality of the evidence? Nurs Res Pract. 2014;2014. doi:10.1155/2014/896587.

ACE, angiotensin converting enzyme; ACOVE-3, Assessing Care of Vulnerable Elders-3; ADPS, advanced diagnostic and procedure skills; AM, antimicrobial; AOR, adjusted odds ratio; APN, advanced practice nurse; APRN, advanced practice registered nurse; ATS, Australasian Triage Scale; BDI, Beck Depression Inventory;

BP, blood pressure; CAD, coronary artery disease; CHF, congestive heart failure; CI, confidence interval; CNS, clinical nurse specialist; CTR, control group; ED, emergency department; FNP, family nurse practitioner; GP, general practitioner; HA1C, hemoglobin A1C; HPV, human papillomavirus; INT1, intervention group 1; INT2, intervention group 2; LTC, long-term care; LVSD, left ventricular systolic dysfunction; MD, medical doctor; MME, morphine milligram equivalents; NMP, non-medical practitioner; NP, nurse practitioner; NPRTS, Nurse Practitioner Role Transition Scale; OR, odds ratio; PA, physician assistant; PCP, primary care provider; PMHNP, psychiatric mental health nurse practitioner; PrEP, pre-exposure prophylaxis; SACT, systemic anti-cancer therapy; sig, significant.
