# Supplementary material for: A global perspective of advanced practice nursing research: A review of systematic reviews
Source: PLoS One. 2024 Jul 2;19(7):e0305008. doi: 10.1371/journal.pone.0305008 (PMC11218965; doi:10.1371/journal.pone.0305008)
Supplement: S5 Table — (PDF) [file pone.0305008.s009.pdf]

S5 Table. Extraction of review results by indicator category at the Health System level.

			HEALTH SYSTEM
**Role**	**Author (year)**	**Ref**	**Results**
			**Access to Care (8 reviews)**
CNS Acute	Cook (2017)	[71]	Accessible care: specialist nurses guide women with gynecological cancer along the continuum of care and are an easily accessed source of knowledge and support
APN Acute AND Primary	Hyde (2020)	[115]	Access to care: only study which examined the concept of access to care. They did not report a statistically significant result
CNS Acute	Salamanca-Balen (2018)	[126]	Effect of general practitioners or specialist/consultant and CNS visits in any setting concluded that COPD patients in the intervention group visited GPs significantly less (but CNSs more) than those in the control group. Women in the control group visited their primary care providers less often compared to those in the intervention group. However, another study found opposing results, and others were in conclusive or reported non-significant results.
NP Primary care	Ansell (2017)	[100]	Open access scheduling: Increases access to care in 11/11 studies. No p-values reported.
NP Primary care	Carranza (2021)	[61]	Access to care reported 2 studies. Pediatric NP saw greater percent urgent appointments (12.5% vs. 6.2%, p <.001) (1/1); NPs rated higher: Access between visits (93.1% vs. 63.2%), no p-values reported (1/1).
NP Primary care	Mileski (2020)	[49]	Increased access to healthcare in 10.3 % of theme occurrences
NP Primary Care	Stratton (2020)	[121]	Treatment for precancerous lesions: 18.3% of the participating NP's patients (n = 828) had treatment and 6.2% were treated for nonmelanoma skin cancer in NP led surveillance clinic during the first two years of the clinic's implementation. No treatment available before clinic implementation. (1 study)
NP Primary care	Yang (2021)	[54]	Access to care (4 studies) ¾ studies reported significantly greater primary care access (no p-values reported); little difference in 1/4 studies (difference not statistically tested)
			**Consultations (20 reviews)**
APN Primary Care	Chan (2018)	[42]	Equal to statistically significant improvements noted for measures to prevent diabetic foot symptoms, referrals to insulin therapy, referrals to an ophthalmologist, a dietician, diabetes educator, and to receive mental health visits. Statistically significant increases were noted in the number of visits to diabetes-related healthcare professionals in one study and a statistically significant reduction in the number of physicians referrals in another study.
APN Acute	Audet (2021)	[7]	No significant difference between groups identified in ¾ studies with statistically significant improvements noted in attendance to their primary care provider appointment within a week after discharge for patients in the intervention group (1/1 study), No significant difference between groups was identified in three randomized controlled trials
APN Acute AND Primary	Gielen (2014)	[48]	Consultation time: Nurses appear to spend more time with patients than physicians do. Referrals: While there appear to be no differences between nurses and physicians in referrals to secondary care, patients cared for by nurses seem to make more return visits than patients cared for by physicians.

NP Acute	Johnson (2015)	[105]	Most frequent outcome measured in the review articles was timeliness of care and that the rate increased to 53% after the initiation of the NP navigation process. One study) (N = 352) used the mean interval (measured in days) from the time to abnormal image to treatment initiation (—X = 105, SD = 104, median = 78, range = 1–757), initial suspicious image to diagnosis (—X = 65, SD = 92,median = 37, range = 0–757), and diagnosis to treatment (—X =40, SD = 48, median = 28, range = 0–265) as study metrics.<br>No evidence-based guidelines exist for timeliness of lung cancer care but that their measurements are in line with the RAND Corporation, which recommends no more than two months from suspicion to diagnosis, and six weeks from diagnosis to treatment .<br>After the establishment of the navigation process, the barriers of depression and alcoholism or drugs were not found. A multidisciplinary team was established, which included the hiring of a lung cancer care coordinator to track patients and facilitate process improvements. A mean reduction of 25 days occurred from the initial abnormal image to the initiation of treatment. timeliness of care as an outcome. I n 2003, the mean number of days from suspicion to treatment was 117; the number dropped to 64.5 days in 2007 and to 54.2 days in 2010. In 2007, the mean number of days from suspicion to CT imaging was 11.8, and it dropped to 7.3 days in 2010. -<br>The mean number of days from suspicion to PET-CT imaging was 16.4 in 2007 and dropped to 10.8 days in 2010. -The mean number of days from suspicion to pulmonary consultation was 20.9 days in 2007 and dropped to 13.4 days in 2010
APN Primary Care	Kennedy (2012)	[92]	Improved/altered circumstances for the nurse consultant: found in 4 qualitative studies
CNS Acute	Salamanca-Balen (2018)	[126]	Referrals (e.g. to other health care professionals, health services or clinics): Equal to statistically significant reductions noted in 7 studies in the referrals to healthcare professionals, health services and clinics with the exception of one study, where the number of referrals to primary care and cardiology clinics increased significantly.
NP Acute	Thamm (2019)	[109]	Length of stay in Emergency room: Equal to significantly shorter wait times noted in 4/4 studies. Equal to significantly longer consultations noted in 3/5 studies
NP Acute	Veenema (2021)	[122]	In one study, access to a specialist physician for virtual consultation did not change the volume of consultations but improved diagnosis of acute abdominal pain when service providers worked together
NP Primary care	Carranza (2021)	[61]	Length of consultation times: no reported measure in supplemental table
NP Primary Care	Garner (2017)	[46]	Efficiency: referrals, conferrals (consultations). Three studies examined the relative risk of a conferral in the Nurse-led arm was 1.45 (95% CI 1.0–2.1; p = 0.04 and was 3.22 (95% CI 2.1–5.0; p < 0.001) for patients attending nurse-led care. Higher number of visits to a rheumatologist in the nurse-led care arm than in the rheumatologist-led care arm (mean 1.63 and 1.53, respectively), which was attributed to the restriction in a nurse's ability to prescribe and perform joint injection, and more rheumatology nursing visits (mean 2.28 and 1.5, respectively) .<br>Nurse referrals to other providers (4 studies) relative risk 5.3, 95% CI 3.4–8.2; p <0.0001 (Hill 1994); relative risk 2.8, 95% CI 1.8–4.2; p <0.0001 Referrals: no statistically significant difference between nurse-led care and rheumatologist-led care in 1/1 study. The number of referrals by nurse-led care and rheumatologist-led care to rheumatologists or family physicians was similar in one study.<br>Consultation time (2 studies): Longer in the intervention group 2/2 (no p-values reported).
NP Primary Care	HQO (2013)	[62]	Clinical examination (3 studies) Model 2: Patients in the specialized nursing group received significantly more referrals for echocardiographs among patients with presumed CHF (P <0.01) among CAD patients (3/3). Length of visits Sig. Increase of 11 minutes in average time in one study and (MD 95 minutes in another study) (p <0.001) (2 /2)
NP Primary Care	Kuethe (2013)	[85]	Referrals from primary care hospital No data presented in any of the included studies Duration of consultation with the asthma nurse and the physician: Duration of the first nurse-led follow-up visit was 29.0 (5.2) minutes. The second and third follow-up visits lasted 19.4 (7.2) and 18.3 (6.3) minutes, respectively. Subsequent nurse-led follow-up visits lasted approximately 15 minutes.<br>Duration of consultation with the physician examined in 2 studies. No data about duration of consultation were presented for the paediatrician-led group. Eight per cent of the children had problems that required more frequent input from the paediatrician.

NP Primary care	Loescher (2018)	[72]	Decreased the number of unnecessary referrals to dermatologists in 2/2 studies
NP Primary care	Lovink (2017)	[34]	In LTC: Unplanned consultations for acute conditions increased sig. in intervention group, 3.0 vs 1.2 per year (P <0.0001), 1/1 study
NP Primary care	Swan (2015)	[51]	Number of referrals (3 studies) Two/ 3 investigated the number of specialty care visits; both found no differences between APNs and physicians.
NP Primary Care	Van Vliet (2020)	[53]	Referral (1 study) PAs refer 50% of their patients to another health care professional (e.g., a GP or an emergency department (ED)) while nurses referred 73% (p value not reported). Consultation (1 study) PAs consulted other health care professionals (e.g., a GP, an emergency physician, or a medical specialist) significantly more often compared to nurses (p value not reported).
NP Primary care	Yang (2021)	[54]	Referral pattern (1 study) 1.8 times higher odds of physician referral in states with full practice authority versus those in restricted practice states.
CNS Acute	Kilpatrick (2014)*	[55]	CNSs in alternative provider roles were assessed in four studies with equal to statistically significant improvements in outcomes related to asthma management (e.g., attendance of ER follow-up visits and at 12 months, fewer asthma related ER visits). For patients with type 1 or 2 diabetes, CNSs had significantly more consultations (i.e. patient visits) and consultation times were longer. No significant differences in health care consultations (at most low-quality evidence). CNSs used significantly more resources because they made more referrals to mental health specialists and intervention patients had more general medicine and mental health clinic visits.
NP Primary Care	Donald (2015)* (transition)	[33]	Number of rehabilitation patient-to-staff consultation calls ($p < 0.05$): significant reduction Duration of rehabilitation patient-to-staff consultation calls ($p < 0.05$): significant reduction Total number of consultation calls: Rehabilitation 1 versus 7 calls $p < 0.05$; reported 1/1 Total duration of consultation calls: Rehabilitation 5 versus 48.5 min $p < 0.05$; reported 1/1
NP Primary Care	Martin-Misener (2015)*	[69]	Consultation times (3 studies) Nurse practitioners had longer consultation times than general practitioners.3/3 studies Meta-analysis of two studies with over 2500 patients, the mean total consultation time in the nurse practitioner group was 4.1 min longer per patient (95% CI 3.7 to 4.5; p<0.0001). Heterogeneity was high (I2=97%). Nurse practitioner consultations were significantly longer in 8 of 10 practices; the ratio of general practitioner to nurse practitioner consultation times varied from 0.57 (95% CI 0.49 to 0.67) to 0.92 (95% CI 0.7 to 1.21). 1 study Number of patients who were referred: Nurse practitioner and general practitioner care were equivalent. Non sig.
			**Costs (40 reviews)**

APN Primary Care	Abraham (2019)	[131]	Costs for Laboratory, Diagnostics Procedures, or Medications (Table 2): The cost for requested lab tests was significantly lower (-$44.06; p=0.001) in the APN group in one study). Costs of cardiovascular disease care provided by APNs compared to physicians for adult patients with atrial fibrillation and found the costs for blood tests and diagnostic procedures such as remote cardiac monitoring (Holter monitoring) and echocardiograms were higher by $41.36, $9.80, and $0.32, respectively in the APN group compared to the physician group in another study. Costs for diagnostic procedures such as stress tests, chest x-rays, and electrocardiography were lower by $2.13, $2.39, and $21.83, respectively in the APN group.<br>Three studies reported lower medication costs in patients cared for by APNs compared to patients cared for by physicians. The average cost per adult patient per month for hypoglycemic medications, antihypertensive medications, and cholesterol-lowering medications prescribed to patients with type 2 diabetes mellitus was $21.03 lower in the APN group compared to the physician group, although the difference was not statistically significant researchers from the United Kingdom compared differences in cost for ulcer-healing medications provided to 175 patients by either APNs or physicians in an outpatient clinic (Chan et al., 2009). The mean cost per patient per week for these medications was $86.93 less for patients treated by APNs compared to physicians (p<0.001) (Chan et al., 2009).<br>The total mean cost for cardiovascular medications (acenocoumarol, amiodarone, Ascal, beta-blockers, digoxin, fenprocoumon, sotalol, and verapamil) was $64.97 lower in the APN group compared to the physician group.<br>Costs for Patient Care Visits (Table 3): differences in costs for clinic, telephone, and emergency visit consultations were lower by $10.55, $6.79, and $7.92, respectively, for patients cared for by the APN compared to the physician. In a randomized controlled trial involving 181 patients with rheumatoid arthritis, the average cost per patient for clinic consultations in the United Kingdom was $62.20 less in the APN group compared to the physician group. The mean costs for outpatient facilities was $5,469 (p<0.05) less for patients receiving exclusive diabetes care from 1,536 APNs compared to 78,030 physicians (Lutfiyya et al., 2016). • After stratifying the sample by age (<65 years), the mean consultation cost (facility resources, follow-up, length of consultations, and salary) was $13.30 less (p<0.001) for patients cared for by APNs (Dierick-van Daele et al., 2010). • two randomized controlled trials comparing differences in costs of urinary incontinence care found physicians to be cost-effective by $385.30 in the Netherlands (Albers-Heitner et al., 2012) and $362.82 in the United Kingdom (Williams et al., 2005).<br>Savings of $178.39 for primary care consultations in a sample of patients with diabetes in the APN group, but a loss of $81.39 and $332.27 for consultations to specialists and for outpatient facilities, respectively
APN Primary Care	Chan (2018)	[42]	Economic outcomes: Costs were examined in five studies with no significant changes noted in four studies. One/5 study reported higher costs for inpatient care (favour the control group).
APN Acute	Allsop (2021)	[10]	Cost were examined in 4 studies. All studies reported a reduction in costs (p values not consistently reported
APN Acute	Audet (2021)	[7]	Cost-efficiency: Statistically significant reductions in costs noted in one study and statistically significant increase in program costs favouring the control group noted after 12 months in one study.
APN Acute AND Primary	Bohner (2012)	[98]	Costs were examined in three studies with reductions in hospital costs, intervention costs, and costs per patients noted in all studies. In 1/3 studies, additional home visits did not offset overall cost savings
APN Acute	Edkins (2014)	[91]	Patients managed by the acute care NPs were hospitalized 2306 fewer days than the baseline population cared for by fellows, resulting in an overall $2,467,328 cost savings. When compared to other units, the Burn ICU evidences the lowest provider costs per year ($495,835) and per patient day ($66). These costs in light of the highest utilization (97.5%) among all of University of North Carolina ’s ICUs reflect a unit that has high potential for provider burnout in 1/1 study
NP Primary care	Fichadiya (2021)	[57]	Comparison of cost of NP vs MD led HF care no significant differences in one study
APN Acute AND Primary	Hyde (2020)	[115]	Direct care cost: A total of three studies examined direct care costs with no difference noted in the three studies.

APN Acute	Joseph (2015)	[106]	Cost-effectiveness of endoscopy procedures performed by nurse endoscopists/nurse practitioners compared to medical endoscopists was examined in 2 studies with reduced costs in 2/2 studies
APN Primary Care	Kennedy (2012)	[92]	Reduction of costly health system issues found in 5 quantitative studies (ventilator days-; length of stays-; admission hospital rates—; A&E attendance-; number of appointments-, number of specialties attended. Improved cost effectiveness of clinic found in 1 qualitative study
APN Primary Care	Lawton (2018)	[84]	Cost-effective: Nurse-led care resulted in significantly higher costs per patient compared with doctor-led care, largely owing to differences in the number of hospital admissions and increased use of intravenous and nebulised antibiotics. Total cost of nurse-led care per patient in the first year was £5202, and total cost was £3262 in the second year. Costs of doctor-led care per patient in the first year were £2577 and in the second year £2851
APN Acute	Manoj (2019)	[63]	Wait times and Costs were not reported in retained studies with APNs
APN Acute	Medeiros (2011)	[94]	Two of the NP studies found significant cost savings.
APN Acute	Monterosso (2019)	[60]	Nurse-led care: Two studies found cost reductions. Costs of nurse-led visits were significantly less than standard visits (€234 versus €503; p < 0.001). The average cost of nurse-led follow-up was lower than standard care (€2592 versus €3798; p = 0.11) even though more patients in the nurse-led group attended all five protocol visits (82% vs 60%; p = 0.002). Cost effectiveness assessed in 2 studies with statistically significant reductions noted in 2/2 studies Nurse- led telephone follow-up with group education was most cost-effective for mean annual costs (€3 971, 95%CI, 2975–5186) and had the second highest mean quality-adjusted life years (0.772, 95%CI, 0.745–0.797; highest 0.776, 95%CI, 0.753–0.799) of the strategies tested.<br>Significantly fewer patients who received the nursing intervention had one or more primary care visits (mean =2.75 (S.D. = 2.03) vs 3.59 (S.D. = 4.66)) during 6 months post-surgery found in one study. However, the related costs were not reported
APN Acute	Ordonez-Piedra (2021)	[95]	Cost effectiveness identified in seven studies with significant improvements noted in five studies of cost effectiveness.
CNS Acute	Salamanca-Balen (2018)	[126]	Equal to statistically significant reductions in costs in 40/46 studies with statistically significant increases in costs in 6/46 studies.
NP Acute	Thamm (2019)	[109]	Cost effectiveness or utility Diverse findings noted in two studies including cost reductions primarily related to reduced salaries of NP and increased costs related to additional training for nurses that were incurred by the healthcare organizations.
APN Primary Care	Whiteford (2016)	[111]	Costs reported in two studies with reductions noted in 2/2 studies
APN Acute	Woo (2017)	[96]	Costs in critical care were reported in three studies with equal to reduced costs noted in 3/3 studies.
NP Primary Care	McMenamin (2023)	[47]	Costs measured in four studies with equal to statistically significant reductions noted in 4/4 studies for the intervention group. Although not significant, 1/4 study noted a trend toward higher costs in the intervention group related to long-term care and skilled nursing facility payments.
NP Primary care	Carranza (2021)	[61]	Costs: sig. higher for NP group vs physicians in (1/1) for patients with lung disease. Mean difference £1497 (95% CI [688, 2674], p <.001)
APN Primary Care	Donald (2013)	[44]	Cost-per-patient the sum of the costs associated with primary-care encounters, non-hospital, hospital, and nursing home care was nearly the same for both nursing home sub-groups (1/1)

NP Primary Care	Fraser (2018)	[132]	Fees for service: Annual total savings if APRNs and physician assistants assume all the primary care visits in one state (Florida), total savings are estimated at $339 million Healthcare costs to organization: in the first 6 months of introducing an onsite nurse practitioner program. While the program cost $82,716, the organization realized $1.3 million in healthcare cost savings. Chenoweth and associates (2005) reported a benefit-to-cost ratio of 15:1 for healthcare costs and 2.4:1 in major diagnostic categories. Healthcare costs to health insurer and patient: Using a sample of 9,503 patients, Spetz, Parente, Town, and Bazarko (2013) used costs paid by the health insurer and costs paid by patients in their CMA. Looking at 10 commonly treated conditions, they compared costs accrued over a 14-day period after receiving treatment from an APRN practicing independently, an APRN with a limited scope of practice, or a PCP. Using regression models, estimated mean cost of care was less with APRNs providing the treatment. Spetz and associates estimated a savings of $810 million if states allowed APRNs to practice independently. The Perryman Group estimated Texas would see an annual impact of $24 billion in expenditures, $12 billion in gross product, and 122,735 permanent jobs by 2020; $34.8 billion in expenditures and $17.5 billion in gross product and 151,462 permanent jobs by 2030; and $46.9 billion in expenditures, $23.6 billion in gross product, and 177,220 permanent jobs by 2040, education costs were not included in the estimates
NP Primary care	Garner (2017)	[46]	Costs (4 studies): Non sig. differences in 3/4 ; trend to increased cost in ¼ studies related to initial hospital in-patient and day-patient hospitalizations.
NP Primary care	Jennings (2015)	[103]	Cost of soft tissue injury management was equal between medical, nurse practitioner and extended scope physiotherapist services (1/1)
NP Primary Care	Kuethe (2013)	[85]	Healthcare costs, direct and indirect Costs of outpatient visits were lower in the nurse-led group (outpatient visits costs per patient per year; €156 in the nurse-led group versus €189 in the physician-led group; P< 0.001), not statistically significantly to lower total costs in the healthcare sector (total health costs €343 in nurse led group versus €357 in physician-led group; P = 0.62).
NP Primary care	Leduc (2021)	[93]	Cost savings assessed in 5 studies; Three studies found lower costs per patient in the intervention group while two studies noted higher costs in the intervention group. stat sig not indicated
NP Primary Care	Lovink (2017)	[34]	Costs: sig. reduction in total costs in 1/1 study. The costs were EUR 6638 in the control group and EUR 4471 in the intervention group (P = 0.01) In LTC: Costs: no significant difference in emergency department costs and hospital admission costs in 1/1 study
NP Primary care	McParland (2022)	[35]	Costs (3 studies): Non-significant reductions in costs noted in 2/3 studies. A significant reduction in health care costs in 1/3 studies (no p value reported)
NP Primary care	Mileski (2020)	[49]	Reduced healthcare costs mentioned in 9 of 136 occurrences of facilitator themes, or 6.6%
NP Primary care	Morilla-Herrera (2016)	[36]	Cost At 24 weeks after discharge, total Medicare reimbursements for health services were $1,238,928 in the control group vs $642,595 in the intervention group (P<.001)
APN Primary Care	Newhouse/ Stanik-Hutt (2013)	[39-40]	CNS: Four studies reported on costs. Studies were conducted in postpartum care, patients receiving end-of-life care, and guideline implementation for patients with radical prostatectomy. Equal to statistically significant reductions noted in costs when comparing CNS and non-CNS groups.
NP Primary Care	Sun (2022)	[38]	Costs in home-based primary care (5 studies): Trends to cost savings noted in 2/5 studies with no p value reported. 1/5 studies reported significant reductions in mean costs per patients in the post intervention year, No significant reductions at 2 years or mean day cost differences in 1/5 studies. One study reported on program costs. Cost savings of $200,000 for 18 participants but researchers did not report how they calculated savings in 1 study. Mean costs per patient in the postintervention year were significantly less in patients with high risk of hospitalization in the intervention arm than in those in the control arm ($5,088 vs. $6,575, p <.001) in 1 study. Total cost: No differences noted in at 2 years or per intervention year in 1 study, mean day-cost differences in 1 study Cost of NP intervention ($24,000 per 100 persons) and of preventing one day of stay in a nursing home ($35) estimated in one study published in 1995.

NP Primary Care	Swan (2015)	[51]	Cost of care (4 studies) 3/4 studies estimated cost using provider salary; of these, 2/3 found that APN care was less expensive compared with physician provided care. One study/4 examined annual laboratory and monthly medication costs; while APN care was less expensive for laboratory services (64.9 ± 34.5 versus 91.5 ± 36.7 euros, P = 0.001), there were no differences in monthly medication costs. Spitzer et al., 1976, examined cost of care by developing a Utilization and Financial Index in which provider salary was aggregated with laboratory, radiology, hospital costs and out of pocket expenditures; no differences were observed between care provided by APNs and physicians.
NP Primary Care	Tsiachristas (2015)	[52]	Cost of ANPs (5 studies): 2/5 showed reduced costs; 1/5 showed costs not sig. reduced; 2/5 showed that cost were increased
NP Primary Care	Yang (2021)	[54]	Wages (1 study): Change in NP hourly earnings compared with physicians before and after the expansion of NP scope of prescribing practice between 2005 and 2010. The researchers indicated that expanding prescribing authority increased NP hourly earnings yet decreased physician earnings Cost (price) of care (6 studies) Fewer restrictions of NP practice authority was associated with lower costs, more prescriptions filled in 5/6 studies
CNS Acute	Kilpatrick (2014)*	[55]	The incremental cost effectiveness ratio (ICER) was €3.61 less in direct costs (i.e. costs directly related to actions and decisions made by the CNS) but €20.34 more in overall costs (i.e. outpatient and inpatient costs for patient resource utilization in all related specialties) per quality adjusted life year (QALY) gained for the CNS intervention compared with usual care. Costs: the clinic visits were significantly higher than usual care; however, total health care costs did not differ significantly between groups. Fewer CNS patients received home help than inpatient controls and the CNS group had significantly lower treatment, health care and societal costs than both control groups.
CNS Acute	Bryant-Lukosius (2015)*	[83]	Costs were examined in 13 studies. There is no instance when resource use or costs were higher with CNS care but often instances when the CNS reduced resource use and costs, despite the fact CNSs represented an ‘add-on’ cost in these studies.
NP Primary Care	Donald (2015)* (transition)	[33]	Cost effectiveness: no significant differences between groups for any patient outcomes or any health system outcomes (1/1) Total costs Hysterectomy: 6% savings sig not reported Hospital costs (30 days) Frail elderly, -$134 (95%CI:-$644 to $376), p = 0.61 Hospital costs (90 days) Frail elderly -$497 (95%CI:-$1216 to $222), p = 0.18 Hospital costs (180 days) Frail elderly -$488 (95%CI:-$1290 to $314), p = 0.23
NP Primary Care	Martin-Misener (2015)*	[69]	Alternative provider nurse practitioner role in ambulatory primary care (4 non-inferiority trials): Nurse practitioners in alternative provider primary care roles could function at least at the level of physician comparators, with equal or lower costs in 4/4 studies Costs (2 studies) meta-analysis of the only two studies of this role that reported costs (2689 patients) with minimal heterogeneity and high-quality evidence, nurse practitioner care compared to general practitioner care resulted in lower mean health services costs per consultation (mean difference: −€6.41; 95% CI −€9.28 to −€3.55; p<0.0001) (2006 euros). All patient/provider outcomes in these studies were equivalent or better for the nurse practitioner.
			**Emergency Room Visits (18 reviews)**
APN Primary Care	Lawton (2018)	[84]	Emergency department attendance: no report
CNS Acute	Salamanca-Balen (2018)	[126]	Lower days in the ICU or E room visits showed non-significant changes between groups
APN Primary Care	Searle (2023)	[133]	ED visits: There were no significant difference for Emergency room transfers in 1/1 study.
NP Primary Care	McMenamin (2023)	[47]	Emergency room visits was examined in six studies with equal to statistically significant reductions noted in 6/6 studies.
NP Primary Care	Driscoll (2015)	[90]	Emergency Room visits: p = 0.81. (1 NP study)

NP Primary Care	Elder (2015)	[86]	Unexpected representations to Emergency Room: Positive impact in one study (1/1)
NP Primary Care	Fung (2014)	[45]	Visits to the emergency room for post-surgical women with ovarian cancers: increase in (1/1) but sig not specified
NP Primary Care	HQO (2013)	[62]	Emergency room visits: Model 1: Fewer visits at 12 months than 6 months for the NP group; no differences at 12 months (1/1) Visits Model 2 sig increase in visits (In one study: 6.1 vs 2.8, (p = 0.001)) (2/2)
NP Primary Care	Ismail (2013)	[102]	A&E attendance from older care home residents (17%): Statistically significant reduction after controlling for seasonal variation.
NP Primary Care	Jennings (2015)	[103]	Unscheduled returns for emergency NP (2 studies): Sig. lower rate in 1/2 studies (2.3% compared with 4.2% for the medical patients (p <0.001). 2% for the emergency NP group vs 1% (not sig.)
NP Primary Care	Jeyaraman (2022)	[104]	Number of Emergency room visits with NP led triage (3 studies) Trends towards a decrease in Emergency room visits were noted in 2/3 studies (no p value reported). The number of patients visiting Emergency room increased by 51 visits per month compared to the traditional nurse-led triage mode in 1/3 studies. (no p value reported) A 5% decrease in Emergency room visits in the NP team triage group noted in 1/3 studies (no p value reported). The number of Emergency room visits preintervention dropped from 2194 Emergency room visits over 6 weeks to 1699 patient visits over one month during the postintervention period in 1/3 studies. (p value not reported).
NP Primary Care	Leduc (2021)	[93]	Emergency room Transports: Reduction in transport to hospital found in 10/10 studies with stat sig in 4/10.
NP Primary Care	Lovink (2017)	[34]	Emergency room visits not leading to hospitalization: Sig reduction in the intervention group in 1/1 study (P = 0.001).In LTC: Emergency room visit: sig decrease in 1/2 studies, (p = 0.006), no sig differences in ½ studies Primary HC: Number of visits to the emergency room : incidence rate ratio of 1.5 for the intervention group compared with the control group (P = 0.02) in 1/1
APN Primary Care	Newhouse/ Stanik-Hutt (2013)	[39-40]	Number of unexpected Emergency room or urgent care visits (5 studies); 1/5 studies reported results favouring NP group and 4/5 reported no difference between groups (no p-values reported).
NP Primary Care	Osakwe (2020)	[37]	NP-home visits on Emergency room visits: sig 2/2. Significant reductions in the Emergency room visits by 35.56% and 23.7% after implementation of the home based primary care (HBPC) program after with 6 months (p = 0.001) and 12 months (p = 0.001) and home care patients who received NP-home visits had less Emergency room visits at 2 weeks (p = 0.0005) and 4 weeks (p = 0.0055) compared to those receiving usual care. No significant difference in the number of Emergency room visits between the 2 groups at the 8 week period (p = 0.800).
NP Primary Care	Sun (2022)	[38]	Emergency room visits (8 studies) 5/8 reported significantly less Emergency room visits among intervention participants; no group difference in 3/8 studies
NP Primary Care	Van Vliet (2020)	[53]	Non-conveyance (ambulance transport) (n=3 studies) non-conveyance rates ranging from 20% –50% for PAs. Non-conveyance rates for the NP were not described.
NP Primary Care	Martin-Misener (2015)*	[69]	Number of patients who had at least one emergency room or urgent care visit Nurse practitioner and general practitioner care were equivalent. Non sig.
			**Health Care Service Delivery (25 reviews)**
NP Primary Care	Baker (2017)	[41]	Use of health care resources examined in one study and low numbers of admissions in the three-month follow-up period made analysis unfeasible
APN Primary Care	Kennedy (2012)	[92]	Improved adherence to appointments: found in 1 qualitative study (Tough, 2006)

APN Acute	Kobleder (2017)	[78]	Health service utilization was examined in one study with no group differences found between the intervention and control groups in relation to hospitalizations and office-based visits to oncologists. The same study found significantly more primary care visits for the control group and a trend towards more visits to the Emergency department in the intervention group.
NP Primary Care	Ansell (2017)	[100]	No Show rates: 4 studies. Unchanged ¼; reduction ranging from 1.44% to 5% in ¾ studies). No p-values reported.
NP Primary Care	Carranza (2021)	[61]	Continuity of care: reported in 1 study. NPs rated higher. Same Provider (62.1% vs.36.8%); Patient inclusion in plan of care) (79.3% vs.47.4%). No p-values reported.
NP Primary Care	Fung (2014)	[45]	Feasibility of transitional care model for patients with schizophrenia, not sig. (1/1) Home-based intervention for individuals with SMI/HIV, a significant improvement in depression (P = 0.012) and in the physical component of health-related quality of life (QOL) (P = 0.03) from baseline to 12 months. fewer primary care visits (β = −0.95 ± 0.16, P = 0.0003) for post-surgical women with ovarian cancers
NP Primary Care	Garner (2017)	[46]	Acceptability of nurse-led care assessed in 4 studies. Acceptability of nurse-led care superior in ¾ studies and unchanged in ¼ studies
NP Primary Care	HQO (2013)	[62]	Specialist visits: Model 1: more specialty visits at 12 months compared with 6 months in both groups; no differences at 12 months 1/1 Model 2: (no data) Primary care visits Model 1: mean number of visits 3.1 SD = 2.38, no sig differences between groups Model 2: (no data)
NP Primary Care	Jennings (2015)	[103]	Collaborative model of care: (1/1 study) increased patient throughput with larger numbers of patient presentations being seen
NP Primary Care	Kwok (2022)	[99]	Health services utilization: A meta-analysis was not performed due to significant heterogeneity in reported outcome measures including composite outcomes and differences in how outcomes were measured. Overall, there is no significant for reducing health service utilization.
NP Primary Care	Leduc (2021)	[93]	End of life care in 3 different ways between 5 studies One study implemented a palliative care framework and set of tools in addition to a palliative care nurse providing and modeling good palliative care. They report a 7% reduction in hospital admission in the last eight weeks of life. One author contributed three retrospective cohort studies to this review, one of which was a subgroup analysis of patients with moderate to severe advanced dementia. These studies evaluate palliative care consults provided by nurse practitioners that address goals of care and symptom management. All three studies showed a reduction in hospital admission in patients with palliative care consults, and both studies measuring ED visits found a reduction in these as well. Patients who had earlier consults had a hospital admission rate that was 13.2% less than those without consults (p=.003).
NP Primary Care	Lovink (2017)	[34]	Number of primary healthcare contacts: no sig differences. Difference is significant once outpatients contacts and primary care contacts are combined: 16.3 per patient in the intervention group vs. 24.3 per patient in the control group (P = 0.04) Process evaluation: no study was identified where implementation was an outcome measure in its own right
NP Primary Care	Morilla-Herrera (2016)	[36]	Service use 1/2 studies At 180-days of follow-up, significant differences in the minutes/month spent by the community nurse between groups [34.5(102.0) vs 96.1 (352.2); P: 0.05]; 1/2 studies the intervention had no effect on overall service use rates at 30 or 120 days
NP Primary Care	Osakwe (2020)	[37]	Transition care and case management: half as many Emergency room visits compared with the usual care group (mean=0.50, SD=1.2 versus mean =0.99, SD=2.5; P = 0.096) (1 study).
NP Primary Care	Swan (2015)	[51]	Healthcare resource utilization (4 studies): Consultation length: Four studies examined consultation length. Three studies found that APN consultations were 3.0 [29] to 4.3 [33] minutes longer than those provided by physicians. Two RCTs and one follow-up study examined total number of primary care visits with conflicting findings at 1 year but fewer visits among APN patients at 2 years. One RCT and its follow-up study examined hospitalization and emergency room or urgent care visits with no significant differences between groups.

NP Primary Care	Tsiachristas (2015)	[52]	Health care utilization: Three studies 2/3 found no differences and one/3 study found an increase.
NP Primary Care	Van Vliet (2020)	[53]	Follow-up contacts (n= 1 study) Follow-up contact after the completion of prehospital EMS care also indicated no significant differences between PAs and nurses Resource use (n=1 study) One study found in 107 cases other EMS resources were released from the scene and put back in service while the NP attended the patient, (by default, two units respond to a call). Eighteen high utilizers of 911 were connected with a social work organization, and 12 of 18 (66.7%) decreased their use of EMS in the 90-days following.
NP Acute	Veenema (2021)	[122]	Higher crude (uadjusted) resource use was noted in one study and one study showed similar resource use with the addition of the NPs.
NP Primary Care	Yang (2021)	[54]	NP growth over time (3 studies) 1 study reported that restricted practice regulations reduced NP growth rates by 25% over a 7 year period. 1 study showed no sig difference in practice authority and growth in rural communities over a 4-year period. 1 study showed that NP growth occurred across most states but that growth was significantly higher in states with reduced practice authority (prescribing) Overall health service utilization (n = 11) 10/11 studies reported higher services utilization under Full Practice Authority Primary care utilization included number of NP visits in primary care, routine check ups, rates of cancer screening, chronic disease management and preventable hospitalization, education, counselling, and medication-related visits, reduced use of the emergency, increase in number of psychotropic and opioid prescriptions with no decrease in mental health outcomes, increase in number of opioid misuse treatment admissions. No sig difference in State -level Opioid and benzodiazepine prescription rates with full prescriptive authority.
NP Primary Care	Zhang (2020)	[127]	Perceived barriers of pre-exposure prophylaxis implementation (26 studies) among health providers the lack of such a request from patients (56, 95% CI = 48–64%), concerns about toxicity and resistance (55, 95% CI = 34, 74%), lack of knowledge about PrEP (47, 95% CI = 27, 66%), concern about the cost to patients (46, 95% CI = 31, 62%), lack of clear guidance (41, 95% CI = 36, 47%), concerns about patients' adherence (38, 95% CI = 26, 50%), concerns about no supporting evidence (33, 95% CI = 9, 63%), time management (29, 95% CI = 13, 49%), risk compensation (28, 95% CI = 17, 40%), lack of comfort regarding PrEP (24, 95% CI = 5, 50%) (Fig. 2). Ideal location to provide pre-exposure prophylaxis (26 studies) Health providers reported that the ideal locations for PrEP care included HIV clinics (p = 71, 95% CI = 67, 74%), primary care settings (p = 42, 95% CI = 27, 58%), sexually transmitted disease clinics (p = 40, 95% CI = 8, 78%), and public health departments (p = 21, 95% CI = 16, 27%).
CNS Acute	Bryant-Lukosius (2015)*	[83]	Following surgery for cancer, CNS transitional care was reported in 2 studies and assessed as superior in reducing mortality 2 years post-surgery by half, improving uncertainty in illness at 6 months, and reducing primary care visits.<br>Usual care was superior in improving functional dependence, physical quality of life, depressive symptoms and symptom distress for patients with cancer. Health care delivery: Post-discharge care of patients with heart failure was reported in 3 studies, There was no instance where usual care performed significantly better than CNS care. CNS care reduced time to death or re-hospitalization and also reduced the combined end points of death or re-hospitalization, improved adherence to treatment recommendations and patient satisfaction and reduced costs and length of re-hospitalization stay. Healthcare delivery: Post-discharge care of elderly patients was reported in five studies. CNS transitional care was superior to usual care for time to re-hospitalization; total and multiple re-hospitalizations; short, moderate and long-term re-hospitalizations and re-hospitalization length of stay as well as a number of cost outcomes (daily hospital costs, health services charges, total re-hospitalization costs and total reimbursement costs).<br>Health system outcomes were examined in two studies Of the 93 health system outcomes, CNS care was superior for 25 outcomes and equivalent for 68 outcomes. Post-discharge care of high-risk pregnant women and infants Three studies evaluated CNS transitional care for high-risk pregnant women and very low birthweight infants. CNS care performed better that usual care in all instances. CNS care was superior for two patient outcomes related to immunization of infants at 8 weeks and maternal satisfaction.
NP Primary Care	Donald (2015)* (transition)	[33]	Additional interventions (RR: 1.02, 95%CI: 0.66–1.56, p = 0.93)

CNS Acute	Kilpatrick (2014)*	[55]	Resource use was examined in five studies for CNSs in Complementary provider outpatient roles with equal resource use noted in 3/5 studies. Statistically significant increases in resource use noted in 2/5 studies in the intervention group where CNSs used significantly more resources because they made more referrals to mental health specialists and pre-natal visits. Intervention patients had more general medicine and mental health clinic visits than patients in the control group in one study. At 24 months, there were no significant differences between groups in health resource use or charges
APN Acute	Kilpatrick (2015)-Inpatients*	[56]	NP group: No significant differences noted in 2/2 studies for hospital length of stay, diagnostic tests, surgical procedures, hospital costs, consultations per patients, referrals to dietetics, total hospital charges, ancillary costs, pharmacy and radiology costs. Significant increase noted in received home care services received for patients discharged home in 1/1 study.
NP Primary Care	Martin-Misener (2015)*	[69]	Return visits: (3 studies) nurse practitioners were more likely to ask patients to return than the general practitioners (2562 patients; I2=76% (RR 1.32, 95% CI 1.20 to 1.46); p<0.0001). The number of patients who made return visits within 2 weeks for the index reason was reported in three trials. A meta-analysis, including almost 3500 patients (I2=5%), indicated that more nurse practitioner patients than general practitioner patients made return visits for the same problem or within 2 weeks (RR 1.18; 95% CI 1.06 to 1.32; p=0.002). One study examined the number of return visits for any reason over 1 year and found equivalent results.
			**Hospitalization (29 reviews)**
APN Acute	Allsop (2021)	[10]	Readmissions examined in 4 studies. One study reported a significant reduction in readmissions (p = 0.02) while another study noted a significant increase at 2 weeks post discharge (p<0.015).
APN Acute	Audet (2021)	[7]	Rehospitalization Equal to statistically significant reductions of re-hospitalizations in 5/5 studies
APN Acute AND Primary	Bohner (2012)	[98]	Rehospitalizations at 3-month, 6-month and 12-month periods were examined in four studies with equal to significant reductions noted in 4/4 studies for the intervention group.
APN Acute	Edkins (2014)	[91]	No significant difference was found between the 2 groups for ICU length of stay (LOS), readmission rates,
NP Primary care	Fichadiya (2021)	[57]	Differences in HF readmission rates were only significantly reduced with NP-led care after one year compared to usual care (p <.001); no significant differences were noted after two years
APN Acute AND Primary	Hyde (2020)	[115]	Readmission, return, and referral rates was examined in two studies and no significant differences were identified.: Return rates were higher in one study related to a confounder (presence of wheeze). Higher return rates in the APN group in 1/1 study // ICU activation: one study that examined ICU activation rates. This was used as a measure of organizational impact following introduction of a new model of APN care. The authors reported ICU activation rates had reduced from 100% pre-model to 50% then 64% post implementation
APN Primary Care	Lawton (2018)	[84]	Hospital admission: Data show a statistically higher proportion of hospital admissions in nurse-led care over the trial period.
APN Acute	Ordonez-Piedra (2021)	[95]	hospital readmissions identified in nine studies with statistically significant reductions noted in all studies.
CNS Acute	Salamanca-Balen (2018)	[126]	Equal to statistically significant reductions in hospitalizations and/or readmissions
APN Primary Care	Searle (2023)	[133]	All nurse specialist/nurse practitioner studies reported a reduction in the rate of hospitalisations. Findings in only one study reached statistical significance, and this was unblinded and at high risk of bias in 3/3 studies
NP Primary care	McMenamin (2023)	[47]	Hospitalizations examined in six studies with equal to statistically significant reductions noted in 6/6 studies
NP Primary care	Barker (2018)	[89]	Unplanned hospital transfer: reduction 10/12; 7/12 reached statistical significance.

NP Primary care	Carranza (2021)	[61]	Hospital admission rates for patients with lung disease: sig. higher in NP group (1/1) 66 NP vs. 42 MD (relative rate of 1.52, p = .03); -43 vs. 23 bronchiectasis-related (relative rate 1.59, p = .22)
NP Primary care	Driscoll (2015)	[90]	Hospitalizations: p = 0.81. (1 NP study) Hospitalizations for CHF: p = 0.66 (1 NP study) Median hospitalizations or ER visits per patient: p =0.14 (1 NP study)
NP Primary care	HQO (2013)	[62]	Hospitalization Model 1: no sig differences in patients hospitalized at 6 months (2/2) and 12 months (1/1) Model 2: stat sig decrease in proportion of patients hospitalized after receiving nurse-led secondary prevention (1/1)
NP Primary care	Kuethe (2013)	[85]	Hospital admissions (4 studies) pooled data showed substantial heterogeneity (RD -0.02; 95% CI -0.06 to 0.02; I2= 59%). For patients with stable asthma, no sig difference between groups
NP Primary care	Leduc (2021)	[93]	Hospital Admission Reduction in 15/16 studies, effect ranged from 3% to almost 50% reduction with Evercare. Stat sig. reductions in 8/16 studies
NP Primary Care	Lovink (2017)	[34]	Hospital admissions: sig decrease in the intervention group (p = 0.03) (1/1) Hospital days: no sig differences (1/1) In LTC: Hospital admissions: 2/4 studies found sig reductions (p = 0.03 and p = 0.001); no sig differences in 2/4 studies In LTC: Number of hospital days: sig. decrease in 1/1 study, from 4170 per 1000 patient years in the control group to 1310 per 1000 patient years in the intervention (P <0.001). Primary HC: Number of hospital admissions: no significant difference in 1/1 study
NP Primary care	Mileski (2020)	[49]	Decreased Hospitalization identified in 27.2 % of theme occurrences
NP Primary Care	Morilla-Herrera (2016)	[36]	Hospital admission (readmission) 4 studies; 2/4 studies indicated sig differences (Number of hospitalizations reduced at 3 month of follow-up [IG:47 (23%) vs CG:68(33%); p = 0.003 (Imhof); By week 24 after the index hospital discharge, control group patients were more likely than intervention group patients to be readmitted at least once (37.1 % vs 20.3 %; P<.001)) and 1/4 studies showed no significant difference, 1/4 reported a reduction (no p-value reported)<br>Patient Institutionalization (admission to residential care) 3/5 studies indicated significant fewer transfers to residential care; 1/5 no difference; 1/5 trend in favour of intervention (no p-value reported)
APN Primary Care	Newhouse/ Stanik-Hutt (2013)	[39-40]	Hospitalization: Seven studies. 3/7 studies reported findings favouring the NP intervention and 4/7 reported no differences between groups (no p-values reported)
NP Primary Care	Osakwe (2020)	[37]	NP-home visits on hospitalizations: not sig. in 2/2 studies. The mean length of stay per hospitalization was 6.3 days in the intervention group and 5.1 days in the control group (p = 0.7) and p value in study 2: (p = 0.514). NP home-visits on readmission: 4 studies. 2/4 studies reported significant decreases in hospital readmissions: for patient following cardiac surgery with the addition of home visits (p = 0.023) in one A 59.42% decrease in readmissions at 6 months (p = 0.001) after enrollment in the home based primary care intervention led by a NP noted in another study, however the result was not sustained at the 12 month-interval (p = 0.087). Not sig findings in 2/4 studies.
NP Primary Care	Schadewaldt (2011)	[67]	No differences in hospital admissions at the 10 year follow up in one study
NP Primary Care	Smigorowsky (2020)	[50]	Effect of NP-led care on 30-day readmission rates for HF (2 studies) The meta-analysis using a model of random effects revealed NP-led care had no statistically difference (Risk Ratio: 0.74, 95% CI: 0.47, 1.17, Z = 1.27, p = .20) on 30-day readmission rates in HF. I2 statistic is 15% and indicates that at the risk of heterogeneity is low
NP Primary Care	Sun (2022)	[38]	Hospitalization (12 studies) 10 /12 studies found that NP home visits led to significantly fewer hospitalizations. A trend of decreased hospitalizations noted in 2/12 studies with non-significant results. Nursing home admissions (3 studies) Fewer nursing home admissions in intervention groups in 3/3 studies. P values not all reported.

CNS Acute	Kilpatrick (2014)*	[55]	Significantly shorter postpartum re-hospitalization length of stay, more prenatal visits, and lower prenatal hospital charges
CNS Acute	Bryant-Lukosius (2015)*	[83]	Data were combined from the two studies examining re-hospitalization at 6 months post-discharge and found no significant differences.
NP Primary Care	Donald (2015)* (transition)	[33]	Re-hospitalisations (RR: 0.60, 95%CI: 0.21–1.73, p = 0.34) Index re-hospitalisation up to 42 days: Meta-analyses (n = 766, pooled relative risk (RR): 0.69, 95%CI: 0.34–1.43, I2 = 0%). Not significant. Any re-hospitalisation up to 180 days: Meta-analysis: (n = 800, pooled RR: 0.87, 95%CI: 0.69–1.09, I2 = 32%) were inconclusive (low quality). Not significant. Index re-hospitalisation over 90 days (RR: 0.55, 95%CI: 0.32–0.94, p = 0.03) in complex care patients (both low quality): significant reduction for complementary provider nurse practitioners. Index re-hospitalisation over 180 days (RR: 0.62, 95%CI: 0.40–0.95, p = 0.03) in complex care patients (both low quality): significant reduction for complementary provider nurse practitioners.
NP Primary Care	Martin-Misener (2015)*	[69]	Number of patients who were hospitalized at least once Nurse practitioner and general practitioner care were equivalent. Non sig.
			**Length of Stay (22 reviews)**
APN Primary Care	Chan (2018)	[42]	Hospital admission, length of stay and emergency presentation: Hospital admission, length of stay and emergency presentation were reported in nine studies. Equal to statistically significant improvements were noted in all studies.
APN Acute	Allsop (2021)	[10]	Statistically significant improvements in LOS in 9/12 studies and improvements that were not statistically significant in 1/12 study
APN Acute	Audet (2021)	[7]	Hospital length of stay: Equal to statistically significant reductions in LOS n 2/2 studies
APN Acute	Curr (2015)	[134]	Emergency room length of stay was statistically shorter intervention groups in 3 studies and not significant in 1 study.
APN Acute	Edkins (2014)	[91]	No significant difference was found between the 2 groups for ICU length of stay, readmission rates,
APN Acute AND Primary	Hyde (2020)	[115]	Length of stay: no significant differences in length of stay in 2/3 studies, in the third study increased length of stay was attributed to higher acuity of patients seen by the APNs in the ICU. no significant differences in length of stay
APN Acute	Manoj (2019)	[63]	A statistically significant difference in length of stay results among three groups (MD group—4.6 hr, MD/NP group—4.2 hr, NP group—3.7 hr) with a significantly shorter length of stay in the NP group (p <0.001).
APN Acute	Medeiros (2011)	[94]	ICU length of stay: statistically significant reductions noted in 3/3 studies of NP care
CNS Acute	Salamanca-Balen (2018)	[126]	Equal to statistically significant reductions in LOS in 33 studies with two studies showing a statistically significant increase.
NP Acute	Thamm (2019)	[109]	Length of stay in Emergency room: Equal to significantly shorter wait times noted in 4/4 studies.
APN Acute	Woo (2017)	[96]	Length of stay in Emergency room was reported in 4 studies with equal to statistically significant reductions noted in the NP-directed care model in 3/3 studies and similar results noted in collaborative care model in 1/1 study. Length of stay in critical care was reported in 7 studies with equal to statistically significant reduction noted in the NP directed care model in 3/3 studies and similar LOS noted on the collaborative care model in 4/4 studies. One out of four studies noted statistically significant reductions in patient transfers to other services, patient discharges to rehabilitation services, and time to discharge older patients on intravenous antibiotics/wound therapy.
NP Primary Care	Elder (2015)	[86]	Nurse-initiated X-Rays showed little impact on ED LOS in two studies/ 3. ED LOS: 1/1 indicated that ED LOS was not impacted by nurse-initiated analgesia.

NP Primary Care	Galiana-Camacho (2018)	[87]	Average length of stay per patient of 180 minutes, together with 78.5% of patients seen in less than 4 hours.
NP Primary Care	HQO (2013)	[62]	Length of stay: Model 2: no sig. difference in median LOS at 1 year (6 days, p= 0.49) (1/1)
NP Primary Care	Jeyaraman (2022)	[104]	Emergency roomlength of stay (LOS) (9 studies) All nine studies in the NP team triage model showed a decrease (median = -28.50 minutes) in ED LOS favoring the intervention group. 5/9 studies showed significant decrease in LOS and 4/9 indicated a decrease without reaching statistical significance. Patient discharge from ED within benchmark times (4 studies) At 60 minutes, 41% of patients discharged from the ED in the NP team triage vs 16% in the traditional nurse-led triage group (1/1 study) At 90 minutes, 30% of low-acuity patients in the NP team triage group discharged vs 12% in the traditional nurse-led triage group. (1/1 study) At 4 hours, 98.1% of patients discharged under 4 hours in the in the NP team triage group compared to 94.7% in the traditional nurse-led triage group. (1/1 study) Under 6 hours, 85.7% of patients discharged in the NP team triage group vs 80.1% in the traditional nurse-led triage group. (1/1 study) Time to triage (2 studies) Statistically significant decrease (pre-intervention time to triage (Median: 4; IQR: (2, 10)); post-intervention time to triage (Median: 3; IQR: (1, 8)) favoring the intervention group noted in ½ studies. 98% of patients in the intervention group were triaged within 15 minutes vs 75% of patients in the comparison group. (p value not reported)
NP Primary Care	Leduc (2021)	[93]	Length of stay: decrease in 4/4 studies, ranging from 0.2 days fewer (43) to 1.2 days fewer in the intervention group (32). Stat sig not indicated
NP Primary Care	Morilla-Herrera (2016)	[36]	Length of Stay 2/2 studies: showed a significant decrease in length of stay in the intervention group (p < 0.002; Huang and Liang); at 24 weeks, mean LoS for readmitted patients in the control group (n=69) was higher than in the intervention group (n=36), (11.0 +/- 10.6 days vs. 7.5 +/- 4.8 days; p<0.001) (Naylor 1999).
APN Primary Care	Newhouse/ Stanik-Hutt (2013)	[39-40]	Hospital Length of stay in 2 studies. Care involving NPs was similar to care involving only MDs in terms of hospital LOS (2/2 studies; no differences between groups, no p-values reported) CNS: Length of stay. Seven studies reported length of stay. Studies were conducted with inpatient settings with patients following coronary bypass surgery, end-of-life care, undergoing radical prostatectomy, post total knee replacement and post partum settings. Equivalent to statistically significant reductions in LOS for patients cared for in the CNS group were noted when compared with the control group in 7/7 studies.
CNS Acute	Kilpatrick (2014)*	[55]	LOS was examined in 2 studies for CNSs in Complementary provider outpatient roles with statistically significant reductions noted in the intervention group in 2/2 studies. LOS: Psychiatric inpatient length of stay was significantly reduced in 1/1 study.
APN Acute	Kilpatrick (2015)-Inpatients*	[56]	CNS group: LOS: no significant differences between the groups
CNS Acute	Bryant-Lukosius (2015)*	[83]	CNS care was superior in reducing hospital length of stay for very low birthweight infants and post-partum mothers, maternal and infant initial hospitalization charges, infant physician charges, home caregiver costs and maternal and infant total costs and charges.
NP Primary Care	Smigorowsky (2020)	[50]	No significant difference between NP-led care and usual care on length of stay in postoperative cardiac surgery (mean I2 difference [MD] = -0.89, 95% CI: -2.44, 0.66, Z = 1.13, p = .26,) in 2/2 studies. I2 statistic is 0%
			**Patient Safety (19 reviews)**
APN Acute	Audet (2021)	[7]	Postoperative complications, including infection, respiratory complications, and thromboembolism were measured in two randomized controlled trials. No significant association was identified for both randomized controlled trials.
APN Acute	Edkins (2014)	[91]	Urinary tract infection (UTI): lower rates of urinary tract infections when compared to routine medical management the previous year (p < 0.05) Skin breakdown (p < 0.05) significant reduction in 1/1 study when compared to routine medical management the previous year Significant increase in DVT identification in the NP group (4% vs 2.5%, P = 0.02) in 1/1 study because of increased compliance with aggressive screening guidelines in the organization.

CNS Acute	Schoch (2014)	[135]	Time to occlusion of arteriovenous fistula (AVF): AVF access had the highest success rate in terms of survival in all three of their end point measurements in 1/1 study.
NP Acute	Thamm (2019)	[109]	Adverse events were reported in four studies. No significant differences were noted in the number of readmissions, unplanned follow-up the initial consultation, mortality, missed fractures.
NP Primary care	Carranza (2021)	[61]	Treatment complications: 4/4 no sig difference between NP and physician groups<br>Adverse effects 4/4 studies no sig difference between NP and physician groups
NP Primary care	Driscoll (2015)	[90]	Adverse events: (1 NP study) There were no differences in adverse events among groups. (1/1)
NP Primary Care	Galiana-Camacho (2018)	[87]	Medication and medical history: completed in 97.4% of patients encounters (1/1). Patient safety: Medication interaction: documented in 87.5% of cases (1/1). Sexual health: documented in 65.9% of cases (1/1). No unplanned readmissions by the Transitional Emergency Nurse Practitioner (TENP) were adequate (1/1). All diagnostic tests requested by the Transitional Emergency Nurse Practitioner (TENP) were adequate (1/1).
NP Primary Care	Garner (2017)	[46]	Adherence to lab tests: no stat. sig. difference between nurse-led care and rheumatologist-led care in 4/4 studies for: mandatory monitoring of laboratories for patients; out-of-range blood tests. Hospitalizations: the number of hospitalizations 5/5 no sig. difference in nurse-led care; number of unplanned family physician visits: no diff. at 12 months and 24 months (1/1) Safety: Adherence to lab tests: no stat. sig. difference between nurse-led care and rheumatologist-led care in 4/4 studies for: mandatory monitoring of laboratories for patients; out-of-range blood tests.
NP Primary Care	HQO (2013)	[62]	Risk factor management Model 2: CAD patients receiving care from specialized nurses were 5 times more likely to achieve appropriate blood pressure (P <0.001) management and 3 times more likely to have appropriate lipid management (P <0.001) (1/1)
NP Primary care	Jennings (2015)	[103]	Missed injuries and inappropriate management: no sig difference (1/1)
NP Primary care	Loescher (2018)	[72]	Decreased the number of unnecessary biopsies in 2/2 studies
APN Acute	Manoj (2019)	[63]	There were no major adverse events including stroke, myocardial infarction and death in any group.
NP Primary care	McParland (2022)	[35]	Falls (1 study): Case-finding for referral to other services did not reduce falls risk. (p value not reported)
NP Primary care	Morilla-Herrera (2016)	[36]	Falls rate: 1/2 studies showed significant reduction in falls (p = 0.003); ½ studies found no significant differences in the number of falls
APN Primary Care	Newhouse/ Stanik-Hutt (2013)	[39-40]	CNS: Complications. Three studies reported patient complications. Studies were conducted with patients discharged following a stroke or transient ischemic attack, receiving care in a surgical intensive care unit, post-operative cardiac surgery, and a pregnancy wellness program. Equal to statistically significant reduction in complications noted in 3/3 studies when comparing CNS and non-CNS groups.
NP Primary Care	Yang (2021)	[54]	Malpractice (1 study) Between 1999 and 2012: 31% lower malpractice payments per 1,000 physicians in states with FPA compared with those with restricted practice authority
CNS Acute	Kilpatrick (2014)*	[55]	Health system outcomes, restraint reduction did not increase staff hours.
APN Acute	Kilpatrick (2015)-Inpatients*	[56]	CNS group: Patient safety: no significant differences in the number of sitter walk-aways or adverse patient events between suicidal and non- suicidal patients

NP Primary Care	Donald (2015)* (transition)	[33]	Falls: estimates favoured nurse practitioner care
			**Quality of Care (11 reviews)**
APN Primary Care	Chan (2018)	[42]	One non-randomized controlled trial reported that a nurse practitioner-led dementia outreach service in residential aged care facilities resulted in significantly higher level of staff satisfaction with regards to dementia care.
APN Acute	Cooper (2019)	[136]	CNS+ANP: -Both roles bring benefits to patients; both roles are effective;Both the ANP and CNS roles are considered to add value, are favourably received and improved quality of care or provided a similar quality of care as doctors
APN Acute	Joseph (2015)	[106]	Quality and safety of nurse endoscopists/nurse practitioners compared to medical endoscopists was measured in five studies. No complications with either the nurse-led colonoscopies or with the physician-led colonoscopies in 5/5 studies
APN Primary Care	Kennedy (2012)	[92]	Improved recording of medical information found in 2 quantitative studies
CNS Acute	Schoch (2014)	[135]	Quality of care Incident rates of arteriovenous fistula (AVF): statistically significant increases in the use of AVF in the first 12 months following the addition of the renal access coordinator (RAC) in 2/2 studies arteriovenous graft (AVG: reduction in the creation of AVG in 3/3 studies, no p value indicated, central venous catheter (CVC) at start of dialysis (incidence) was identified in 4 studies with equal to statistically significant reductions of catheter use in 4/4 studies following the RAC addition. Similar trends noted for prevalence with higher rates attributed to late referrals to the RACs.
NP Primary care	Jennings (2015)	[103]	Quality of care (4/4) Emergency NP patient group rated their care as excellent compared with the medical care patient group (68% vs. 50%, Fisher's exact p <0.02) (1/1)
NP Primary Care	Kuethe (2013)	[85]	Quality of care: No sig difference in 2/3 studies Asthma-specific and child-specific quality the ratings for the paediatrician-led care and nurse-led were higher than for the general practitioner-led care (P <0.05).
NP Primary care	Lovink (2017)	[34]	Assessing Care of Vulnerable Elders-3 (ACOVE-3) quality indicators: Higher score in favour of the intervention, 54% compared with 34% in the control group (P <0.001).
NP Primary care	Mileski (2020)	[49]	NPs improved healthcare quality in 19.1% of theme occurences
NP Primary care	Tsiachristas (2015)	[52]	Quality of care: 1 study reported improvements in Quality of care
NP Primary Care	Yang (2021)	[54]	Care quality: 3/3 reported no sig differences in overall quality of care by state level of NP practice regulations
			**Scope of Practice (22 reviews)**
NP Primary Care	Andregard (2015)	[137]	The analysis revealed four themes: (i) a threat to professional boundaries, (ii) a resource for the team, (iii) the quest for autonomy and control, and (iv) necessary properties of a developing interprofessional collaboration. Based on these 'themes, the synthesis was created, made visible and presented as a metaphorical journey: a tortuous journey towards a partially unknown destination. Table 3 provides an overview of the synthesis and the themes.

APN Acute	Clavo-Hall (2018)	[138]	Job title: The most frequently reported role, faculty, refers to clinical nurse leaders (CNLs) who are formally working as professors, assistant professors, lecturers, program directors, and adjunct instructors. There were 59 (62%) NHA CNLs identified practicing in a faculty role. The second most frequently identified role was the clinical management role (12%), such as chief nursing officer, clinical executive director, and unit manager. Then came specialty clinical roles (11%) that included advanced practice nurses, clinical administrators, clinical educators, clinical coordinators, and informatics specialists. The traditional staff nurse role was the next most frequently reported role (9%), followed by a variety of other roles (N = 5, 5%). There were 13 NHA CNLs that were represented in more than one article. The majority described the same NHA role across articles, but 15% (N = 2) reported different roles across articles over time. Role activity: The most frequent activity was expert commentary (33%) on topics such as: potential for CNL practice in new settings or with unique patient populations; pain management outcomes for total knee replacement patients; and in- person peer review. Many NHA CNLs (22%) are actively involved in developing and implementing academic curriculum, including CNL immersion experiences and accelerated nursing programs. NHA CNLs are also involved in developing and/or reporting on CNL implementation in health systems across the nation (18%). Finally, NHA CNLs were also involved in conducting systematic literature reviews (10%) and research (14%) on topics such as CNL practice, dyspnea management for hospice patients, COPD and ventilator associated pneumonia, and the impact of depressive symptoms on quality of life Role activity by role type: Faculty showed the greatest diversity of activities being involved in CNL implementation, general research, literature reviews, writing expert commentaries on diverse topics alongside more expected activities such as curriculum development and implementation. NHA CNLs in specialty clinical roles were also diversely involved in activities such as CNL implementation, curriculum development and testing, expert commentary and research on the CNL. Even certified CNLs who identified as staff nurses were conducting research and writing expert commentaries and literature reviews. Overall, NHA CNLs were found to be conducting a diverse amount of academic and clinical scholarship
APN Acute	Cooper (2019)	[136]	Governance and regulation appear to be country specific. In the Republic of Ireland, the ANP roles undergo a registration process to accredit practitioners, whereas the CNSs undergo an 'approval process'. In Australia, the ANP role is protected, but the CNS role is not. In Canada, ANPs have an expanded role function and legislative authority that allows them to perform additional activities (eg to diagnose and pr escribe) whereas the CNS has the same privileges as a registered nurse. ANP: -One study found that ANPs were more likely to be involved with research. -In the Republic of Ireland and Australia, master's-level preparation is required for the ANP role and there is a move towards this in England -In Canada, master's level is typical for ANPs while there is no standard for CNS education - CNS: -One study found that CNSs were more likely to be involved in audit. -In Canada, master's level is typical for ANPs while there is no standard for CNS education. In New Zealand, there is no national certification for the CNS role
NP Primary Care	Dawson (2015)	[101]	Three studies examining legislation found that legislation allowing for autonomous or full scope of practice for NPs reduces hospitalizations among Medicare- Medicaid beneficiaries and improved health outcomes in their communities, increased access to care for injured workers in rural communities and timely accident report filing for their injuries.

NP Primary Care	Harkless (2018)	[139]	State-determined Medicaid reimbursement and scope of practice legislation shapes nurse practitioner clinical practice. With full scope of practice authority and Medicaid reimbursement at 100% of the physician's rate, more NPs work in primary care, a higher number of practices employing NPs accept Medicaid, and primary care practices with NPs are more likely to be located in rural and high poverty areas.<br>When Nurse practitioners are identified primary care providers there is discrepancy of payment by insurance providers : The factors may include local policies at the NP's employment site or their parent health system, state laws and regulations that do or do not require the recognition of NPs as primary care providers. Managed care organizations (MCOs) do not have consistent standards for who is or is not a recognized as a primary care provider contracted to provide care to a panel of patients. Instead, the provider credentialing process used by MCOs, along with subsequent contracting standards, vary between and within states, with the percent of MCOs credentialing NPs stable at 74% in 2012 and 75% in 2016. Reimbursement parity is not the same for NPs: Legislated policy barriers, as well as disadvantageous third- party insurer policies, create financial sustainability issues for NP-managed clinics. Reimbursement was singled out as a key consideration and barrier affecting sustainability of both nurse-managed clinics and NP private practice. Incident to billing – practices still bill under a physician rather than an NP to receive full payment. Very limited data are available on the process and outcome of credentialing and contracting for reimbursement of NPs by location, setting, or specialty.
CNS Primary	Hourahane (2012)	[140]	The 11 synthesized findings pertain to a list of factors that facilitate and inhibit consultant nurse (CN) role implementation and development: 1. Four role functions: The CN needs to engage in all four role functions to facilitate role development, working across different areas with patients and at a strategic level with appropriate time management and an integrated approach are also necessary. Not working with patients and at a strategic level also hinders the CN's role development. Difficulty in demonstrating their impact in changing culture, service and practice development also impede role development. 2. Ability to influence through leadership and vision. 3.Organisational structure with autonomy allowing the CN to make major decisions. 4. Good working relationships with key individuals, supported by supervision and regular meetings, facilitate the CN role. 5. CN receiving administration and managerial support from the organisation. 6.Role clarity Managers and other members of the team also need to have a clear understanding of the CN role and be able to differentiate it from other nursing roles. 7. Role development: The consultant nurse's previous experience of developing a role with clear business and succession planning which is supported by the organisation (particularly in providing secretarial support). Time in the post, confidence and workload management also facilitate the crafting of the role which moves in cycles rather than in a linear way over a period from 18 months up to 5 years. 8. Role preparation should include induction; education to master's level with a variety of educational experiences including a broad preparation and structured approach which may use a medical model. Ongoing engagement and support for continuous professional development and research is essential. 9. Personal attributes include the ability to lead change, empower others in exercising leadership using a determined yet collaborative approach. Other essential attributes are self-confidence, motivation, credibility and commitment. 10. Work overload: Achieving a good work life balance and managing increased workload each facilitate role development. 11.What the CN role gives the CN: The role labelled as the CN gives the CN higher status, facilitates clinical credibility and enables professional development. In addition, having a clinical caseload and involvement in clinical care maintains that credibility.
APN Acute AND Primary	Hutchison (2014)	[141]	Domains of APN practice with frequency effect size 1.Autonomous or nurse-led extended clinical practice (0.82)<br>2.Developing the practice of others (0.62)<br>3.Improving systems of care (0.62)<br>4.Developing and delivering educational programs and activities (0.60)<br>5.Nursing research and scholarship (0.54)<br>6.Leadership external to the organization (0.24)<br>7.Administering programs, budgets, and personnel (0.18)

APN Primary Care	Kennedy (2012)	[92]	Development of other staff because of work of nurse consultant found in 9 qualitative studies Detriment to other staff because of work of nurse consultant found in 2 qualitative studies and in 1 quantitative study (1 survey question on deskilling of doctor experience/role —added comment “for most part not an issue”). Delay in decision to wean found in 1 quantitative study. Development of networks, services, improved/activated policies found in 8 qualitative studies . Improved standards of care found in 5 qualitative studies. Clinical Social significance: was captured in outcomes related to reduced mortality, waiting times and service/appointment utilization. From a broad perspective this was also suggested qualitatively, for example developing services, improving care, reducing waiting times<br>Professional social significance was related to consultants in the professional social significance category, such as contributing to role extension, the development of new nursing roles recruitment and retention, reducing others’ workload and contributing to meeting the education needs of staff.<br>Professional social validity, several qualitative studies indicated that staff valued nurse consultants’ contribution and three surveys illustrated the usefulness of nurse consultant-led services amongst GPs and nursing staff
APN Acute	Lyness (2021)	[130]	A clearly defined skillset, scope of practice and role: Some out-of-hours services appeared to expect non-medical practitioners NMPs to function as GPs, although they do not have the same breadth and depth of knowledge and skills. Therefore, it is not surprising that they felt unprepared for certain patients and sometimes avoided them
NP Acute AND Primary	Niezen (2014)	[107]	The organizational environment imposes a set of factors, located outside the professional communities of physicians and NPs, which influences the successful implementation of NPs in a healthcare setting. In total, eight subcategories were defined: (1) organizational policy support, (2) complexity of cure and care provided, (3) facility arrangements, (4) employment arrangements, (5) institution’s familiarity with the (regulatory) environment (6) type of health setting, (7) experience in working with NPs, and (8) (inter)professional collegiality. The first factor, organizational policy support, was addressed in eight studies, and encompasses a demarcation of the NP’s role, that professional tensions are addressed, that protocols or formal procedures are available and that unwarranted restrictions, such as limited prescription authority, are removed. Four studies indicate that the complexity of the cure and care provided is an important factor in the acceptance of NPs as cure providers. The less complex the cure component (medicine), the more positive the attitude towards NPs fulfilling these tasks. Both facility and employment arrangements influence the ability of NPs to perform their role. The lack of proper facility arrangements, such as not having one’s own office/treatment space and computer, was experienced as a barrier to task reallocation. Like facility arrangements, employment arrangements can limit or enhance the full integration of NPs into a team or clinical practice. Other factors within the organizational environment that can facilitate/hinder task substitution are: the health setting’s familiarity with (governmental) regulations and rules , the type of health setting , the amount of (previous) experience with NPs , and (inter)professional collegiality . Institutions need to know how (the interaction between) regulations and rules can either facilitate or hinder the roles and functions of NPs. The type of health setting focuses on the difference between, for example, a community clinic and a hospital; the former being less supportive of expanding the scope of nursing practice roles to the domain of medicine than the latter, as it has different expectations and often less experience with NPs . (Inter)professional collegiality refers to the perceived support from within the nursing professions, the support, effort and trust from management and the enthusiasm from other people involved [1]. Institutional environment entails the influences of legal, political and societal institutions in shaping the healthcare system. These external factors can have a strong impact since they involve: (1) legislation, (2) socio-economic forces, (3) governmental (research) policy, and (4) patients’ perceptions. Legislation is referred to as a barrier by six studies. The socio-economic forces shaping NP care are reported in seven studies
NP Acute	Veenema (2021)	[122]	Fifteen studies attempted to determine the number of NPs in Emergency rooms. National surveys found a growing number of Emergency rooms staffed with NPs. One study noted that NPs account for 5% of independent billing in this setting. Workforce data often included physician assistant roles. Seven studies examined whether NPs could care for patients independently. In one study, patients were discharged by the NP more frequently directly following triage.

NP Primary Care	Wang- Romjue (2018)	[142]	1. Autonomy denotes the NPs' independent decision-making ability to choose the best patient care delivery method based on their knowledge, skills, critical thinking, and judgment within the full scope of practice as delineated by state regulations and organizational policies. However, 28 states have restrictive regulations limiting autonomy.<br>2. NPs' roles and responsibilities: Multidimensional; includes diagnosing, prescribing treatments, promoting health, preventing disease, providing holistic care, advocating for the patient, teaching patients, and being versed with computer/virtual technology; straddles medical curative model and nurse model which causes role friction due to patient care time constraints; organizational guidelines are unclear and compound the problems; NP practice not well understood by administrators, physicians, and patients.<br>3. Practice relationships NPs creating alliances and partnerships through honest and direct communication with ancillary staff, registered nurses, and nursing supervisors helps maximize collaboration and team work; evidence has indicated that ancillary staff, such as MAs, undermine the NPs' practices by not preparing their patients in advance for the NP-patient encounter like they would for the physician–patient encounter. Sometimes, this disparity of support is reinforced by clinic managers and/or administrators who tolerate these behaviors; regarding relationships with physicians challenges include having a supervising physician over their practice on record, having to seek physician signatures for certain aspects of care and record keeping, and lacking collegiality with physicians; regarding patient relationships this is viewed by NPs as the most enriching of all relationships.<br>4. Organizational work environment pressures Organizational constraints of time, financial constraints, and institutional metrics affect NP autonomy and practice
NP Acute AND Primary	Xue (2016)	[143]	Scope of Practice regulation on:<br>1) NP Workforce: Consistent evidence has shown that the number of NPs and growth of the NP workforce were highest in states with greater practice authority. Evidence from four studies indicates that states with more favorable NP practice environments have higher per capita NPs.<br>2) Care provision: Scope of practice: Five studies provide evidence indicating that states with expanded practice authority showed the greatest growth and advancement of NP primary care provision. Evidence further suggests that less restrictive SOP regulation was linked with promoting care provision by NPs in rural and medically underserved areas.<br>3)Scope of practice: Access to Care and Healthcare Utilization: One study found a significant impact of NP SOP regulations on health care utilization but no conclusive evidence of an impact on access to care.<br>4) Scope of practice: Healthcare Costs: Two studies that examined the effects of NP SOP regulation on health care providers' incomes arrived at partially inconsistent results related to NP prescribing controlled drugs and substances.
APN Acute AND Primary	Hako (2023)	[144]	APN capability consists of six dimensions: can apply competencies in familiar as well as novel situations, works well in teams, is creative, has a high level of self-efficacy, knows how to learn, and identifies the factors affecting the scope of practice.
NP Primary care	Elder (2015)	[86]	Nurse-Initiated analgesia was improved with the introduction of nurse-initiated analgesia protocols in three studies (3/3).
NP Primary care	Galiana-Camacho (2018)	[87]	Degree of autonomy for APNs at work ranging from a 27% to 84% of patients treated autonomously (3/3)
NP Primary Care	Hyer (2019)	[117]	NP Practice: NPs identify and assess weight status during an office visit in 7 studies out of 15, and they intervene or counsel patients regarding obesity in 5 studies out of 15. Provider perceived responsibility for managing obesity: 3/3 studies showed the positive impact
NP Primary care	Mileski (2020)	[49]	Unrestrictive or least restrictive scope of practice for NPs was mentioned in 10 of 136 occurrences of facilitator themes, 7.35%

NP Primary Care	Patel (2019)	[108]	NP SOP and characteristics of the health delivery system: 8 studies more growth in the number of NPs in states with the least restrictive SOP policies. Patients in states with the least restrictive NP SOP policies were more likely to have an NP as their PC provider.<br>The results of most studies showed a positive association between less restrictive NP SOP policy and NP workforce capacity. 1/8 study reported no significant association between NP SOP policy and number of NPs licensed to practice per 100,000 population. NP SOP and characteristics of the population-at-risk: 5 studies. 4/5 studies reported that NPs with less restrictive Scope of practice were more likely to work in PC, provide care in rural and high-poverty areas. Accept patients under Medicaid; sig difference in 2/5. One study found no sig difference NP SOP and utilization of health services: 4 studies greater use of preventive services and decreased rates of avoidable hospitalizations, hospital readmissions within 30 days discharge from rehabilitation, and hospitalizations of nursing home patients in states with the least restrictive NP SOP policies in ¾ studies One out of the four studies reported an increased likelihood of patients receiving a referral to a physician from an NP at Community Health Centers in states with SOP policies that allow NPs to practice without physician supervision. ¼ studies reported that a larger supply of NPs, without considering other state- and patient-level factors, did not significantly affect healthcare utilization.
NP Primary Care	Yang (2021)	[54]	NP Supply (6 studies): 5/6 studies showed a positive association between FPA and NP supply. 1/6 (nursing home study), reported an insignificant relationship (Intrator et al., 2015).<br>NP Workforce distribution. (4 studies) 1/3 studies indicate higher odds of NPs in rural areas in states with FPA, compared with those with restricted regulations, though the difference was not statistically significant 2/3 studies found significantly greater numbers of NPs in rural areas of states with FPA than those with restricted practice regulations 1/1 study evaluating the odds of NPs practicing primary versus specialty care by level of state NP practice regulations and found that NPs had 13% higher odds of practicing primary care in states with FPA than in states without it NP autonomy (2 studies). NPs had greater day-to-day practice autonomy when they had full independent prescriptive authority, yet having practice independence for diagnosis and treatment only showed little effect on autonomy. 2/2 studies: no difference in NP hospital admission privileges by level of state NP practice regulations and hospital admission privileges did not differ by prescriptive or practice independence.<br>Mobility (1 study) odds of moving from one state to another by state NP practice regulations between 1992 and 2004 and found that NPs were 46% more likely to move from a state with no controlled substance prescriptive authority to one that allows NPs to prescribe them. NP-provided health service use (5 studies) Full practice authority led to sig increases in care provided by NPs in 4/5 studies. Neg association in 1/5 studies (services provided in LTC)
APN Acute	Ramis (2013)	[145]	Advanced practice registered nurses working in roles within acute, hospital, or tertiary care centre, intensive care and critical care units as well as hospital emergency departments. Six themes were identified in the study.<br>**1) Expert knowledge:** Outcomes to patient, provider and healthcare system-APNs have expert knowledge that is required, utilized and is ongoing. Outcomes to education-APNs are an educational resource for their departments and spend a large amount of time educating peers, staff, patients and their families.<br>**2) Spectrum of work activities:** Outcomes to provider and healthcare system-APNs experience a certain amount of routine in their daily work; APNs work in diverse areas with varying management responsibilities and functions. They may work independently or interdependently<br>**3) Confidence and familiarity:** Outcomes to provider-APNs exhibit familiarity with tasks and have the ability to use their previous experience to deal with varied issues; APNs demonstrate intuitive knowledge in their practice**.** Outcomes to patient and healthcare system-confidence in their role allows APNs to make quick and effective decisions, time manage, prioritize and bypass hierarchy if required; APNs have a strong sense of responsibility and accountability<br>**4) Negative experiences:** Outcomes to patient, provider and healthcare system-having to do a large component of administrative work detracts APNs from patient care and contributes to overtime, work overload and frustration; organizational factors can affect APN experiences and service utilization**.** Outcomes to provider-negative experiences with staff relationships, organization and workload can impact greatly on the APN experience at a personal and professional level<br>**5) Relationships:** Outcomes to patient, provider and healthcare system-multi-disciplinary relationship building and maintaining

			positive relationships are essential components of the APN experience. Outcomes to patients, healthcare system and education-APNs demonstrate expert communication skills in their professional relationships as well as patient and staff interactions; consultancy and liaison can be extensive and may link the APNs unit to other internal departments and at times, areas external to the hospital environment**.** Outcomes to patient, provider, healthcare system and education-acting in a professional manner in all circumstances is paramount to the APN experience.<br>6) **Patient-centered experiences:** Outcomes to patients-the patient is central to the APN experience**.** Outcomes to patients, provider, healthcare system and education-APNs provide support to staff, patients, families and each other; APNs have positive and rewarding experiences which are usually related to positive patient care experiences
APN Acute AND Primary	Jokiniemi (2012)	[146]	The goal of the position is to help provide better outcomes for patients by improving services and quality, to strengthen leadership, and to provide a new career opportunity to help retain experienced and expert nurses in practice. Each position should be structured around four core functions of expert practice, professional leadership and consultancy; education, training, and development; and practice and service development, and research and evaluation. The position should involve working directly with patients, clients, or communities for at least 50% of the time available.<br>In the USA, the role of the CNS dates back to the early 1940s. The American Nurses Association defines the CNS as "an advanced practice nurse who integrates and applies a wide range of theoretical and evidence-based knowledge and is licensed, certified and/or approved to practice.
			**Wait Times (12 reviews)**
APN Acute	Allsop (2021)	[10]	Time to surgery was reported in seven studies. Trends toward reductions to statistically significant reductions reported for time to surgery and improvement in the number of patients having surgery within 24 hours
APN Acute	Manoj (2019)	[63]	Wait times were not reported in retained studies with APNs
NP Acute	Veenema (2021)	[122]	Wait times were reduced in two out of two studies.
APN Primary Care	Whiteford (2016)	[111]	Waiting time reported in one study with considerable reductions noted (no p value reported)
APN Acute	Woo (2017)	[96]	Wait time in the Emergency Department was examined in five studies with equal to statistically significant reductions noted in the intervention group for time to consultation in 4/4 studies. Statistically significant reductions in time to treatment were noted in 1/1 study where a greater proportion of patients (15.4%) managed by emergency NPs received analgesia within 30 min of arrival at the ED compared to patients managed by physicians (1.6%) (P <0.001). Wait time in critical care was examined in one study with statistically significant reductions in time to treatment for patients with acute ischemic stroke (P <0.001).
NP Primary Care	Ansell (2017)	[100]	Wait times for appointments: reduced wait times in 11/11 studies. Mean reduction of -11.3 days (SD +/- 8.3 days) for all included studies after implementation. Open access scheduling: Reduced wait times in 11/11 studies. No p-values reported. Wait times for appointments: Use of NPs reduced wait times for appointments in 2/2 studies. No p-values reported. Telephone follow-up consultations reduced wait times for appointments in 2/2 studies. No p-values reported. Measures to promote self care reduced wait times for appointments in 2/2 studies. No p-values reported. Email consultations reduced wait times for appointments in 2/2 studies. No p-values reported.
NP Primary care	Carranza (2021)	[61]	Wait times was reported in 2 studies. Decreased wait time from 46 to 42 days for non-urgent appointments in pediatric NP clinic; no p-values reported (2/2).
NP Primary Care	Elder (2015)	[86]	Did-Not-Wait rate was reported in one study with a 1% reduction in Did-Not-Wait (DNW) rates over a 12 month period following the introduction of the clinical initiative nurses (CIN) role (P <0.001). (1/1) Nurse-initiated X-Rays showed little impact on wait times in two studies/ 3. Wait times: Reduced in 5/6 studies

NP Primary Care	Jennings (2015)	[103]	Wait time: Reduction the number of patients' who did not wait for treatment during service delivered by the nurse practitioner service (1/1) Wait times: 8 studies. Significant reduction in 2/8 studies favors NP: significant reduction in waiting time for patients managed by the emergency nurse practitioners in comparison to the emergency department medical registrars. Time to be seen (median) NP group 14 min (range 5–27) vs. 50 min (range 21–78) in doctor group (p <0.0001). Significant reduction in waiting times for NP managed patients. Waiting times NP was 12 min (range 5.5–28 min), vs. 31 min (range 11.5–76 min), (p <0.001) for doctor group.<br>Reduction favors NP in 3/8, p value not reported or no significant difference in 3 studies. Patients managed by emergency nurse practitioners trended to shorter waiting times when compared with medical officers, with a difference of 7 min (p = 0.06). Fry et al. 2011: Median transitional emergency nurse practitioner waiting time 38 min compared with 59.7 min previous year. Did not wait 4.5% vs. 8.1% in previous year. Considine et al. 2010: waiting time reduction. Wait times: No significant difference in 3/8 studies Van der No significant difference in waiting time 19 mins nurse practitioners vs. 20 mins doctors No significant differences in overall median waiting times. Did not wait rate 11.9% vs. 13.7% (Intervention/Control). Considine et al. 2006: No significant differences in median waiting time between nurse practitioner candidate and doctor managed patients.
NP Primary Care	Jeyaraman (2022)	[104]	Provider initial assessment (9 studies): Significant reductions in mean difference in time to provider initial assessment in 8/9 studies (-3.00 min, 95%CI [-3.47, - 2.43] to -50 min, 95%CI [- 53,63, -46.37] Non-significant trend towards a reduction noted in 1/9 studies (-2.30 min [-5.93, 1.33].<br>Leave without being seen (LWBS) (6 studies) 5/6 studies reported a reduction in percentage of patients who LWBS in the NP team triage. 4/6 studies reported statistically significant decrease in the intervention group; 1/6 studies did not report p value). (p value not reported). 1/6 studies reported a non significant change in patients LWBS. Leave against medical advice (LAMA) (3 studies) Non-significant findings reported in 3/3 studies.
NP Primary care	Loescher (2018)	[72]	Numbers of patients seen in a timely manner: increase (no p value reported)
NP Primary Care	Van Vliet (2020)	[53]	On-scene time (n=2 studies) One/2 studies reported no significant difference between PAs and nurses regarding the length of on-scene treatment time. ½ study: an average length of treatment time on scene of 21.47min but made no comparison with other EMS professionals.

* Systematic review published as constellation papers, with additional methodological data extracted from: 154. Marshall D, Donald F, Lacny S, Reid K, Bryant-Lukosius D, Carter N, et al. Assessing the quality of economic evaluations of clinical nurse specialists and nurse practitioners: A systematic review of cost-effectiveness. NursingPlus Open. 2015;1(2015):11-7.doi:10.1016/j.npls.2015.07.001; and 155. Donald F, Kilpatrick K, Reid K, Carter N, Martin-Misener R, Bryant-Lukosius D, et al. A systematic review of the cost-effectiveness of nurse practitioners and clinical nurse specialists: What is the quality of the evidence? Nurs Res Pract. 2014;2014. doi:10.1155/2014/896587.

A&E, accident & emergency; ACOVE-3, Assessing Care of Vulnerable Elders-3; ANP, advanced nurse practitioner; APN, advanced practice nurse; APRN, advanced practice registered nurse; AVF, arteriovenous fistula; AVG, arteriovenous graft; CAD, coronary artery disease; CG, control group; CHF, congestive heart failure; CI, confidence interval; CIN, clinical initiative nurse; CMA, cost minimization analyses; CN, consultant nurse; CNL, clinical nurse leader; CNS, clinical nurse specialist; COPD, chronic obstructive pulmonary disease; CVC, central venous catheter; diff., difference; DNW, Did-Not-Wait; DVT, deep vein thrombosis; E room, emergency room; ED, emergency department; EMS, emergency medical service; ER, emergency room; FPA, full practice authority; GP, general practitioner; HBPC, home based primary care; HC, health care; HF, heart failure; HIV, human immunodeficiency virus; ICER, incremental cost effectiveness ratio; ICU, intensive care unit; IG, intervention group; IQR, interquartile range; LAMA, leave against medical advice; LOS, length of stay; LTC, long-term care; LWBS, leave without being seen; MA, medical assistant; MCO, managed care organization; MD, mean difference; MD, medical doctor; NHA, not hired as; NMP, non-medical practitioner; NP, nurse practitioner; PA, physician assistant; PC, primary care; PCP, primary care physician; PrEP, pre-exposure prophylaxis; QALY, quality adjusted life year; QoL, quality of life; RAC, renal access coordinator; RCT, randomized controlled trial; RD, risk difference; RR, relative risk; SD, standard

deviation; sig, significant; SMI, serious mental illness; SOP, scope of practice; stat sig, statistically significant; TENP, transitional emergency nurse practitioner; UTI, urinary tract infection.
