# Supplementary material for: A global perspective of advanced practice nursing research: A review of systematic reviews
Source: PLoS One. 2024 Jul 2;19(7):e0305008. doi: 10.1371/journal.pone.0305008 (PMC11218965; doi:10.1371/journal.pone.0305008)
Supplement: S6 Table — (PDF) [file pone.0305008.s010.pdf]

S6 Table. Extraction of review results by indicator category related to Artificial Intelligence.

			Artificial Intelligence
**Role**	**Author (year)**	**Ref**	**Results**
			**Artificial Intelligence-Health Technology (2 reviews)**
APN Acute	Borum (2018)	[147]	Barriers:<br>CDS alerts/nuisance alerts; Incorrect format; CDS systems algorithm: improperly defined/not current with evidence-based practice; Inaccurate patient information/lack of clinical patient information; Inappropriate timing of CDS11–14; Lack of training; Lack of knowledge; CDS inefficiencies; Reliance on manual input; Incorrect CDS content; Hardware issues; Usability; Lack of interoperability
NP Acute AND Primary	Raymond (2022)	[148]	NP involvement in AI enhanced health technology<br>NP involvement and experience with AI-based health technologies<br>Six studies were conducted in a primary care (e.g., family, geriatric and pediatric care), hospital (e.g., acute, post-acute and post-operative) care, and Emergency Department in which a Machine Learning- based clinical decision support systems (ML-CDSS) was developed and whose decision outputs (diagnostic and referral decisions) were compared with the clinical decisions of NPs in their role as diagnosis expert and/or therapeutic expert.<br><br>NP clinical activities and AI-based health technologies (AIHT)<br>NP clinical activities that are enabled by AIHT systems and address AIHT in support of the referral or triage decisions made by NPs were examined in 7/11 studies. Four studies present AIHT-based systems that enable the diagnosis of patient illnesses by NP. Four studies examined AI-based technologies meant to improve the follow-up and surveillance of patients by NPs, and a single study examined illness prevention.<br><br>All 11 studies focus on machine learning-based clinical decision support systems; however, one study is also knowledge-based. Four out of 11 studies also include natural language processing and computer vision as AI components of the system in addition to machine learning. AI-enhanced robotics has yet to figure among the AI-based technologies that have been studied in advanced nursing practice contexts.<br><br>Given the nascent approach, proposed impact of AI based health technology to provide clinical decision support to NPs in assessing patients' physical and mental status and reviewing laboratory test results was examined in seven studies. Six of the seven studies looked to machine learning-based clinical decision support systems in support of NPs' evaluation and classification of patients at risk (of epileptic seizure, admission to the ED, cardiorespiratory insufficiency, rehospitalization, pressure ulcers and cardiac arrhythmias. One study looked at machine learning and computer vision to assist NPs in interpreting clinical images. AI-based clinical decision support is meant to improve the diagnosis capability of NPs, that is, to increase their clinical effectiveness in terms of diagnostic accuracy, consistency, and timeliness.<br><br>Proposed AI based health technology outcomes for patients include AIHT-based support of NPs' patient referral and surveillance tasks is meant to reduce the occurrence of adverse patient outcomes (e.g., rehospitalization) or prevent adverse clinical events (e.g., failure to rescue).

AI, artificial intelligence; AIHT, AI-based health technologies; CDS, clinical decision support; ED, emergency department; ML-CDSS, machine learning-based clinical decision support systems; NP, nurse practitioner.
