# Supplementary material for: A global perspective of advanced practice nursing research: A review of systematic reviews
Source: PLoS One. 2024 Jul 2;19(7):e0305008. doi: 10.1371/journal.pone.0305008 (PMC11218965; doi:10.1371/journal.pone.0305008)
Supplement: Attachment — Submitted filename: Response to Reviewers.docx [file pone.0305008.s011.docx]

Dr Federica Canzan and Reviewers,

Thank you for your insightful comments. The suggested changes have been made in the manuscript entitled *A global perspective of advanced practice nursing research: A review of systematic reviews* (Manuscript ID PONE-D-23-42783) as requested. The changes are summarized in the table below to facilitate the review.

| **Comments** |  | Page |
| --- | --- | --- |
| Academic  Editor | 1. Please ensure that your manuscript meets PLOS ONE's style requirements, including those for file naming <https://journals.plos.org/plosone/s/file?id=wjVg/PLOSOne_formatting_sample_main_body.pdf> and  <https://journals.plos.org/plosone/s/file?id=ba62/PLOSOne_formatting_sample_title_authors_affiliations.pdf> | We adjusted our manuscript’s format to fit PLOS ONE’s style requirements as requested. |
|  | 2. We note that Figures 1 and 2 in your submission contain [map/satellite] images which may be copyrighted. All PLOS content is published under the Creative Commons Attribution License (CC BY 4.0), which means that the manuscript, images, and Supporting Information files will be freely available online, and any third party is permitted to access, download, copy, distribute, and use these materials in any way, even commercially, with proper attribution. For these reasons, we cannot publish previously copyrighted maps or satellite images created using proprietary data, such as Google software (Google Maps, Street View, and Earth). For more information, see our copyright guidelines: <http://journals.plos.org/plosone/s/licenses-and-copyright>.  We require you to either (1) present written permission from the copyright holder to publish these figures specifically under the CC BY 4.0 license, or (2) remove the figures from your submission:  “I request permission for the open-access journal PLOS ONE to publish XXX under the Creative Commons Attribution License (CCAL) CC BY 4.0 (<http://creativecommons.org/licenses/by/4.0/>). Please be aware that this license allows unrestricted use and distribution, even commercially, by third parties. Please reply and provide explicit written permission to publish XXX under a CC BY license and complete the attached form.”  Please upload the completed Content Permission Form or other proof of granted permissions as an "Other" file with your submission.  In the figure caption of the copyrighted figure, please include the following text: “Reprinted from [ref] under a CC BY license, with permission from [name of publisher], original copyright [original copyright year].”  b. If you are unable to obtain permission from the original copyright holder to publish these figures under the CC BY 4.0 license or if the copyright holder’s requirements are incompatible with the CC BY 4.0 license, please either i) remove the figure or ii) supply a replacement figure that complies with the CC BY 4.0 license. Please check copyright information on all replacement figures and update the figure caption with source information. If applicable, please specify in the figure caption text when a figure is similar but not identical to the original image and is therefore for illustrative purposes only. | **FIGURE 1 (PRISMA 2020 FLOW DIAGRAM)**  We reviewed the PRISMA Statement’s website section **Citing and using PRISMA** (<http://www.prisma-statement.org/PRISMAStatement/CitingAndUsingPRISMA.aspx>). Also, the Page et al. PRISMA 2020 Statement paper published in BMJ in 2021 mentions: *This is an Open Access article distributed in accordance with the terms of the Creative Commons Attribution (CC BY 4.0) license, which permits others to distribute, remix, adapt and build upon this work, for commercial use, provided the original work is properly cited. See:* [*http://creativecommons.org/licenses/by/4.0/*](http://creativecommons.org/licenses/by/4.0/)*.*  The template PRISMA 2020 Statement flow diagram provided on the website includes the caption:  *From:*  Page MJ, McKenzie JE, Bossuyt PM, Boutron I, Hoffmann TC, Mulrow CD, et al. The PRISMA 2020 statement: an updated guideline for reporting systematic reviews. BMJ 2021;372:n71. doi: 10.1136/bmj.n71. For more information, visit: <http://www.prisma-statement.org/>  We added this caption in the Fig 1 legend on lines 276-279, and adjusted the Page MJ et al. (ref. #18) citation details we already had in our reference list from Syst Rev. 2021;10:89 to BMJ. 2021;372:n71. doi: 10.1136/bmj.n71 to match the PRISMA 2020 flow diagram caption.  **FIGURE 2 AND 3 (MAPS)**  Fig. 2 and 3 maps were created with MapChart.net After review of MapChart’s webpage (<https://www.mapchart.net/terms.html#licensing-maps>) where they indicate under **Licensing for maps:** This work is licensed under a Creative Commons Attribution-ShareAlike 4.0 International License. *More info here: Creative Commons License* You are allowed to use, edit and modify any map (image) created with the Website or Mobile App for personal, non-commercial, or commercial use/publication freely, by adding any kind of reference to the Website or Mobile App in your work. Some examples of an appropriate reference:   - Keeping the "Created with [mapchart.net](https://mapchart.net/)" or "Created with [historicalmapchart.net](https://historicalmapchart.net/)" or "Created with MapChart" tag that each map has embedded. - A simple link to the front page or any other page of the Website or the Mobile App's store page. - A text attribution on the page or inside the article/ publication where the map is used (as a caption, in the credits, etc).   We additionally e-mailed the PLOS ONE editorial office (Mar. 21, 2024) this information to inquire whether it would it be sufficient to indicate in our paper: "We would like to acknowledge the use of MapChart ([https://www.mapchart.net](https://www.mapchart.net/)) used for creating Figures 1 and 2 in this article."  On Mar. 22, 2024, we received the following reply from Jacob Ulmschneider (PLOS ONE Publications Assistant): “The citation/acknowledgement you have provided would certainly be an acceptable attribution for a map published under a CC BY 4.0 license. Please include this attribution in your figure citations, and your manuscript will be in compliance with PLOS copyright requirements.”  Therefore, Fig. 2 and 3 have been revised to display their embedded "Created with [mapchart.net](https://mapchart.net/)" tag, and we added the attribution approved by the PLOS ONE office in our figure citations on lines 295-298. |
|  | 3. Please review your reference list to ensure that it is complete and correct. If you have cited papers that have been retracted, please include the rationale for doing so in the manuscript text, or remove these references and replace them with relevant current references. Any changes to the reference list should be mentioned in the rebuttal letter that accompanies your revised manuscript. If you need to cite a retracted article, indicate the article’s retracted status in the References list and also include a citation and full reference for the retraction notice. | We reviewed our reference list for completeness and correctness. Thank you for bringing this to our attention.  We changed the publication dates in some references in S1-S6 Tables for those references whose ePub publication year changed once they were attributed their final published citation.  We updated the CADTH reference (ref. #24) with the new link to its search filters database.  We have made also adjusted the Page MJ et al. PRISMA 2020 statement publication citation details (ref. #18) to match the article mentioned in the Fig 1 caption (PRISMA 2020 was simultaneously published in multiple journals). |
|  | While revising your submission, please upload your figure files to the Preflight Analysis and Conversion Engine (PACE) digital diagnostic tool, <https://pacev2.apexcovantage.com/>. | We uploaded Figs 1-3 to the PACE tool, and there were no image problems identified. |
| Comments to the Author |  |  |
| **Reviewer #1** | I would suggest minor improvements, namely:  i) Abstract:  I suggest that under results the sentence written in lines 652-657 expresses more clearly the results of the study "We found highly consistent evidence about the care provided by APNs, NPs and CNSs across 29 indicator categories at the patient, provider and health system levels. Care was assessed as equal or superior to the comparator (usual care, physicians) across a wide range of clinical settings, patient populations and acuity levels in almost all the reviews. Mixed findings were noted for quality of life, consultations, costs, emergency room visits and health care service delivery where some studies favoured the control groups." | As suggested, we have rewritten the abstract results on lines 69-73 such that they now read:  We found highly consistent evidence that APN, NP and CNS care was equal or superior to the comparator (e.g., physicians) for 29 indicator categories across a wide range of clinical settings, patient populations and acuity levels. Mixed findings were noted for quality of life, consultations, costs, emergency room visits, and health care service delivery where some studies favoured the control groups. |
|  | ii) Materials and Methods:  In Database search (line 186) it is not clear why you decide to include reviews published after 2011.  In the assessment of review quality it is not clear if the authors decided to include the reviews even the ones with low CASP scores. | We added on lines 191-193 the following explanation as to why we chose to include reviews published after 2011:  This time period was selected to capture the most recent trends in advanced practice nursing research and because evidence in approximately half of reviews is outdated five years after publication [16].  We clarified on lines 254-256 that we did not exclude reviews based on methodologic quality:  No study was excluded based on methodologic quality because our overview aimed to capture the countries where advanced practice nursing research had been undertaken. |
|  | iii) Results  In line 291 authors refer to Patient outcomes and in line 301 Patient Indicators. Are they synonyms? | We changed outcomes to indicators on line 303. |
|  |  |  |
| **Reviewer #2** | In the PRISMA statement, enter how many articles were found for each database queried. | We revised Fig 1 (PRISMA 2020 Flow diagram) to include how many articles were found for each database queried. |
|  | I would also insert a table with search strings for each database queried. | Search strings for each database queried are reported in S2 Appendix. Combining the approaches used by these articles published in PLOS One (<https://doi.org/10.1371/journal.pone.0277323>; <https://doi.org/10.1371/journal.pone.0299933>), we presented in tabular form and labeled each concept in the PubMed search strings in S2 Appendix. Unless already presented in tabular format, we did not apply this formatting to our search strings which are presented as numbered lines and an adaptation of the database search that was initially developed in PubMed. |
